# Conformational dependence of chemical shifts in the proline rich region of TAU protein†

**DOI:** 10.1039/d4cp02484b

**Published:** 2024-08-30

**Authors:** Johannes Stöckelmaier, Chris Oostenbrink

**Affiliations:** a Institute of Molecular Modeling and Simulation (MMS), University of Natural Resources and Life Sciences Vienna Austria chris.oostenbrink@boku.ac.at

## Abstract

Nuclear magnetic resonance (NMR) is an important method for structure elucidation of proteins, as it is an easily accessible and well understood method. To characterize intrinsically disordered proteins (IDPs) using computational models it is often necessary to analyze and integrate calculated observables with measurements derived from solution NMR experiments. In this case study, we investigate whether and which chemical shifts of the proline-rich region of Tau protein (residues 210–240) offer information about the conformational state to distinguish two different microscopic conformers. Using multiple computational methods, the chemical shifts of these two conformationally distinct structures are calculated. The different methods are compared regarding their ability to compute chemical shifts that are sensitive to conformational change. The analysis of the data shows significant differences between the available methods and gives suggestions for an improved pathway for ensemble reweighting. Nevertheless, the variation in the chemical shifts which are predicted for configurations that are commonly considered to belong to the same conformation is such that this obscures a comparison between distinct conformations. Conformational sensitivity is found for up to ∼26% of calculated chemical shifts. It is found to be unrelated to the atom element and has a minor relationship with the change in the corresponding *ϕ* dihedral angle.

## Abstract 2

Chemical shifts of a stretched and a globular conformer of one TAU-protein fragment are calculated. The conformational sensitivity of each chemical shift is evaluated.

## Graphical abstract

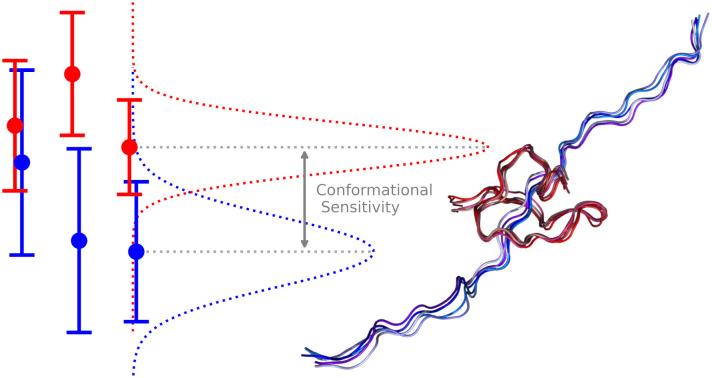

## 1 Introduction

For decades, the well-known structure–function paradigm had its firm place in the understanding of biochemistry.^1,2^ The discovery of much more flexible, disordered, proteins led to a rethinking of established theories in the early 2000s.^3^ Fully disordered proteins are named intrinsically disordered proteins (IDPs), while partly disordered proteins contain intrinsically disordered regions (IDRs).^4^

During the last two decades, IDPs were the subject of significant scientific interest as they can be physiologically active and are predicted to play a role in understanding diseases such as Alzheimer's and Parkinson's disease.^5–7^ It is estimated that more than one third of proteins in eukaryotic organisms feature intrinsically disordered regions.^8^

At room temperature, IDPs can access many different conformational states within less than one microsecond. Therefore, they need to be described by a set of structures, called the conformational ensemble.^9^ To calculate the conformational ensemble of an IDP, molecular dynamics simulation can be performed.^10,11^ Due to the flexible nature of IDPs, they usually feature flatter potential energy surface regions that may span multiple conformations,^12^ which makes computer simulations very sensitive to inaccuracies of the force field. To overcome these limitations, conformational ensembles can be optimized with reweighting algorithms by combining experimental and simulated data.^13^

A chemical shift is a measurement of the resonance frequency change of a nucleus in reference to a standard in an NMR experiment. This corresponds to a change in the magnetic shielding tensor of the atomic core in reference to a standard.^14^ In organic chemistry, it is well understood that the local geometry of a molecule has a strong influence on the chemical shift. In biochemistry, secondary chemical shifts are applied to differentiate between α-helix or β-strand regions in structured proteins.^15–17^ While empirical predictors are designed to handle such cases, it is a topic of discussion to which extent they can capture conformational dynamics.^18–20^ As measured chemical shifts do not represent single conformations, but only the ensemble average,^21,22^ it is observed that chemical shifts calculated from ensembles show increased agreement with experiments.^23,24^

The quality of a reweighted conformational ensemble is crucially dependent on the quality of the input data. To allow reweighting tools to yield the best results, the chosen experimental and simulated datasets should fulfill the following characteristics:

(1) The measured signal must be sensitive to the overall conformation of the sample.

(2) The expected error of measurement must be less than the expected conformational sensitivity.

(3) The measured data must not have assignment errors.

(4) The measured properties should be easily accessible both computationally and experimentally.

To study IDPs, it has become a common practice to use data from residual dipolar coupling, NOE, SAXS and FRET experiments.^13^ While chemical shifts are a property relatively easy to measure and understand, it is disputed if they are an appropriate observable for use with protein ensemble reweighting. Here, we aim to determine if predicted chemical shifts for individual configurations differentiate between distinct conformations. We analysed two distinct conformations of an intrinsically disordered TAU-protein fragment, by selecting five highly similar configurations for each of the conformations. We compare the variation in the predicted chemical shifts within a single conformation to the variation between conformations to determine the conformational sensitivity of the predictions. We subsequently ask the question whether conformationally sensitive chemical shifts can be predicted from the molecular properties, or if a specific algorithm is more suitable to obtain conformationally sensitive chemical shifts. While our work is not aimed to validate the predicted chemical shifts against experimental data, we do perform a comparison against the experimental data to determine if systematic errors in the predictions occur.

## 2 Materials and methods

### 2.1 Molecular dynamics simulation

A 31-amino-acid long fragment of TAU-protein (aa220–aa240) as described in Lasorsa *et al.*^25,26^ was chosen as a test case. The fragment was simulated with acetylated and *N*-methylated N- and C termini, respectively, at 310 K for 500 ns using the AMBER99SB-ILDN force field^27^ with PME calculation of nonbonded interactions using the OpenMM molecular dynamics engine.^28^ We have used the capped ends to reflect the fact that this is just a fragment from a larger protein. The fragment was solvated using 1.5 nm padding in explicit OPC water.^29^ The LangevinMiddle integrator using a 2 fs time-step was used together with SHAKE constraints on the bond lengths. A MonteCarlo barostat was used to keep the pressure at 1 atm, creating an NpT-ensemble.

**Fig. 1:**
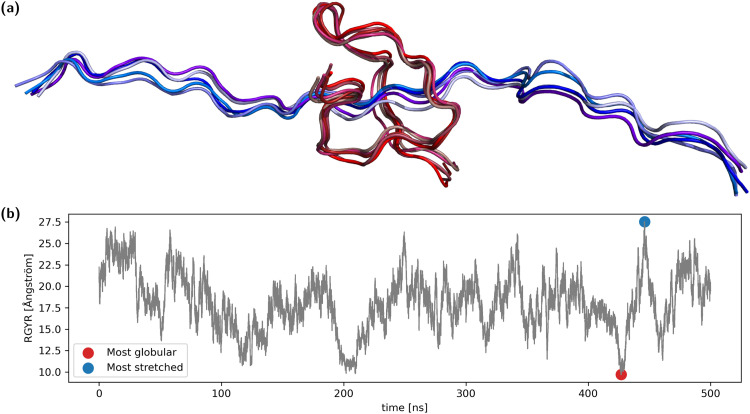
(a) A set of five structures representing the most stretched conformation (blue) and a set of five representing the most globular conformation (red). (b) The radius of gyration of the polypeptide shows reversible fluctuations between ∼9.7 Å and ∼27.5 Å.

Analysis of the radius of gyration of the backbone showed reversible collapse and extension during the entire time span (Fig. 1). From the trajectory, both the frame with the maximal radius of gyration (RGYR) as well as the frame with minimal RGYR were selected, representing the most stretched and the most globular conformation. We selected two of the most differing conformations from the entire simulation to increase the probability of observing large differences in structurally sensitive properties. For each of the two structures, the four most similar conformations were also selected, summing up to two groups of five conformations (Fig. 1). In reference to the corresponding central structure, the maximum RMSD of the globular ensemble is ∼1.4 Å, while it is ∼2.0 Å for the stretched ensemble.

### 2.2 Geometry optimization

As it is known that chemical shifts are very sensitive to changes in the local chemical environment, the selected frames obtained from molecular dynamics were geometry optimized using MOPAC v22.1.0 utilizing the MOZYME protocol and the PM7 semiempirical method.^30^ For each frame, the 31 amino acid long polypeptide plus the two residues of the N- and C-termini as well as the first solvation shell of water were selected. The geometry of all peptide atoms plus termini was optimized, while the position of the water molecules in the solvation shell was frozen to prevent the macroscopic conformation of the peptide from changing.

### 2.3 Chemical shift prediction

Many different methods to predict chemical shifts have been developed in the past decades. Some of those have been selected to be investigated in this work and are listed in Table 1. These methods can be separated into two groups – empirical models using statistical approaches and DFT-based calculations using first principles methods. The class of DFT-based methods applying the gauge-independent atomic orbital (GIAO) theory can be further split using pure quantum (QM) and combined quantum/molecular-mechanics (QM/MM) approaches. Further subdivision can be made into subgroups depending on the solvation method.

**Table 1:** Overview of the tested methods to predict chemical shifts from static protein structures. The column 'Solvation' describes how solvation effects are included into the calculation of the chemical shifts. Calculations that do not take solvation effects into account (solvation 'None') are called vacuum calculations in this article

Method	Type	Solvation	Annotations/settings	Ref.
DFT-based (NWChem)	QM	None	2 functional/basis-set combinations.	31 and 32
DFT-based (NWChem)	QM	Implicit	12 functional/basis-set combinations.	31 and 32
DFT-based (NWChem)	QM	Explicit	3 functional/basis-set combinations.	31 and 32
DFT-based (NWChem)	QM/MM	None	2 functional/basis-set combinations.	31 and 32
DFT-based (NWChem)	QM/MM	Implicit	12 functional/basis-set combinations.	31 and 32
SHIFTX2	Empirical	Empirical		33
SPARTA+	Empirical	Empirical	-first 2 -last 32	34
UCBShift-X	Empirical	Empirical	–shiftx_only –pH 6.5	35
PPM	Empirical	Empirical	-model ann	36

After the geometry optimization, all 2 × 5 conformations of the peptide were saved as PDB files and then converted into the corresponding input format of each method using a MDAnalysis software^37,38^ based custom toolchain.

For use with the DFT-based QM algorithm, the polypeptide had to be preprocessed as the system would otherwise be too large. The polypeptide was split into 33 smaller fragments. Each resulting fragment contains a central amino-acid as well as all other amino-acids and end-groups which feature at least one atom within 4 Å of the central amino-acid. At the cutoff, where the backbone of the protein was cut, the new fragments were missing atoms to replace the broken bonds. To fix the fragments, they were saturated with ACE/NME end-groups.

**Fig. 2:**
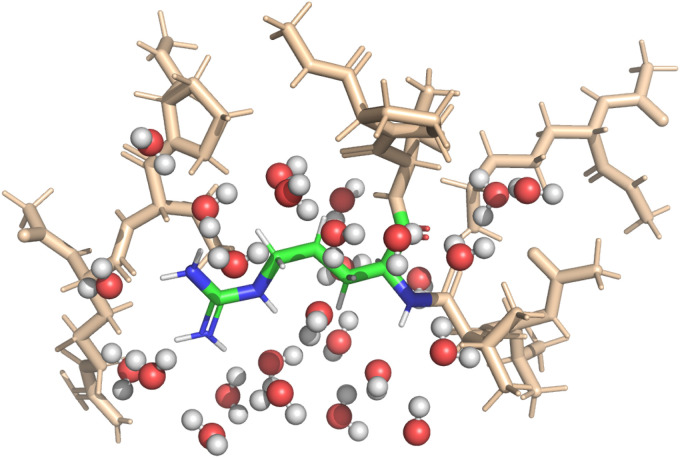
The QM approach splits the entire polypeptide into smaller fragments. One amino-acid is selected for property calculation which then constitutes the center of the created fragment (shown in color). The environment is modeled by all amino-acids with at least one atom closer than 4 Å to the central residue and, in the case of a calculation with explicit solvent, the first solvation shell of water molecules. The visualization shows the setup for calculation of the 22nd amino acid of the polypeptide in its globular form with explicit solvation.

Implicit solvent simulations applied the continuum solvation model COSMO (York–Karplus formulation^39^) as implemented in NWChem. The dielectric constant of the medium was set to 78.4 to represent water. Explicit solvent simulations were set-up to feature one solvation shell around the central amino-acid; itself embedded in implicit solvation. Fig. 2 shows a visualization of the setup for one of the DFT-based QM calculations. The micro-solvation contained all water molecules within 4 Å of the central fragment and was taken from the MD-simulation with preserved geometry and orientation of the water molecules.

**Fig. 3:**
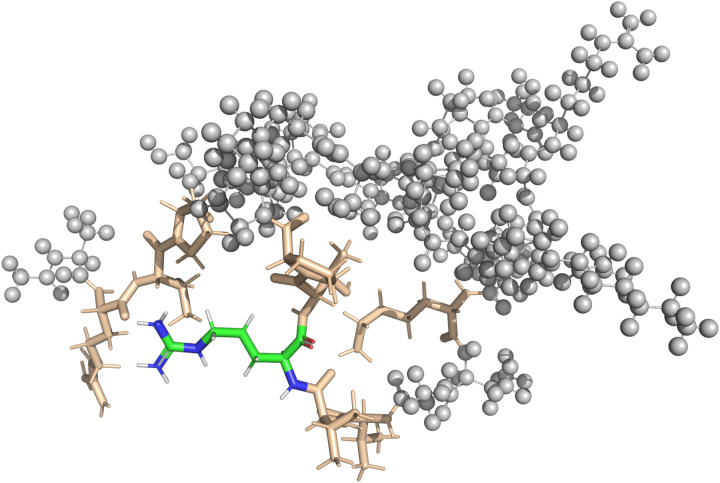
The QM/MM approach splits the molecule into two regions. The QM-region is composed of the central residue (shown in color) and all residues with at least one atom closer than 4 Å to the central residue (shown in brown). The rest of the protein is modeled as the MM-region, shown as gray spheres. Shielding properties are calculated for the atoms of the central residue. The visualization shows the setup for calculation of the 22nd amino acid of the polypeptide in its globular form in vacuum.

In the case of the DFT-based QM/MM approach, the fragmentation was handled by the NWChem software. Similar to the pure QM approach, all amino-acids close to the central amino-acid were included into the QM region of the QM/MM calculation. The QM-region includes the point charges of the MM-region according to the modified AMBER95 force field as implemented in NWChem. Broken bonds between the QM and the MM region were automatically repaired using hydrogen link atoms. In both vacuum and implicit solvent calculations, the interaction zone between the QM-region and the MM-region as well as the MM-cutoff was set to half the box-size. Fig. 3 shows a visualization of the setup for one of the QM/MM calculations.

To evaluate the influence of functional/basis-set combinations, three basis-sets (6-31G*,^40–42^ cc-pdvz,^43^ and pcSseg-1^44^) and four functionals (B3LYP,^45^ Becke97-2,^46^ Becke97-D^47^ and wb97x-d3^48^) were tested. Empirical GD3 dispersion^49^ has been applied where available. The resulting 12 combinations were used with implicit solvent both by the pure QM and with the QM/MM approach. Taking the observations from these calculations into account, a smaller sub-set of three combinations was chosen to test the influence of vacuum and explicit solvent on the results.

To obtain a full set of chemical shifts for the entire polypeptide, the chemical shifts of all 33 central residues were combined. While the empirical methods yield chemical shifts directly, the DFT-based methods yield isotropic nuclear magnetic shieldings. To obtain the chemical shifts, the magnetic shieldings were referenced against standards as recommended in the study by Pavlíková Pecechtlová *et al.*^50^ The chemical shifts of ^1^H and ^13^C were referenced against tetramethylsilane (TMS) (eqn (1)) while the ^15^N chemical shifts were referenced against methylamine (eqn (2)) using a secondary standard referencing scheme, where *δ*^calc^_X_ is the chemical shift of atom X and *σ*^calc^_X_ is the isotropic nuclear magnetic shielding of atom X. The values of 
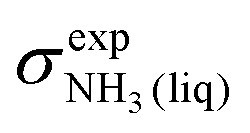
 (244.6 ppm,^51^) and 
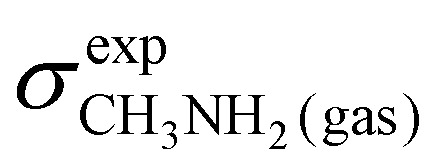
 (249.5 ppm,^52^) were taken from the literature. All DFT-based methods were referenced against standards using implicit solvent (TMS) and vacuum simulations (CH_3_NH_2_).

1 *δ*^calc^_X_ = *σ*^calc^_TMS_ − *σ*^calc^_X_

2
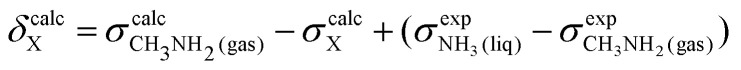


Empirical predictors, which have been trained to predict chemical shifts directly from a three-dimensional structure, are also available. As these models were trained on structured proteins it remains to be tested if they are able to calculate chemical shifts that show conformational sensitivity toward IDPs.

The four empirical predictors work in a straight forward way and take PDBs of the entire polypeptide as input. The water molecules and ions of the system were removed before calculation of the chemical shifts, which are calculated directly without need for a reference calculation. As these predictors are only trained for specific tasks, they are not able to reproduce the chemical shifts of all atoms. Most chemical shifts are calculated with SHIFTX2, which includes the backbone and most of the sidechains (401 atoms in total). The PPM software calculates the chemical shifts of the backbone, C_β_ atoms and most of the sidechain hydrogen atoms (317 atoms in total). Both SPARTA+ and UCBShiftX yield chemical shifts only for the backbone and C_β_ atoms (170 atoms).

### 2.4 Analysis

To analyze the sensitivity of chemical shifts with regards to conformational change, the five stretched and the five globular conformations of the polypeptide were considered to be equivalent in both cases. Thus, the simulation is regarded as five independent measurements of both the stretched and globular conformation.

**Fig. 4:**
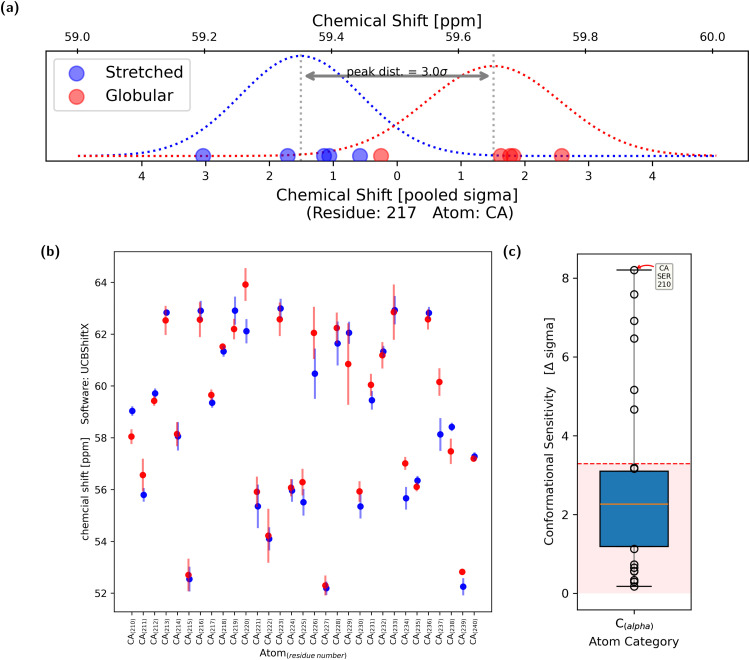
The central methodology of this work can be described using the three plots presented above. All calculations shown in these figures were calculated using the UCBShiftX method. (a) The chemical shift of the C_α_ atom of residue 217 was calculated for each of the 10 conformations. The result is displayed as blue and red dots, depending on whether the conformation was stretched or globular. Dots of the same color are part of the same probability distribution (PDF), shown as a dotted curve. The pooled standard distribution of the two PDFs is noted on the lower *x*-axis. The value zero is set to be in the middle of the two peaks. The distance between the two expectation values, in multiples of pooled *σ*, is used as a metric of the conformational sensitivity and quality criteria of the prediction methods. An overlap of 10% is equal to a distance of 3.29*σ*. The chemical shift yielded from C_α_, residue 217, has a conformational sensitivity of 3.0*σ* and thus is just not conformationally sensitive. (b) Average chemical shifts of both the stretched (blue) and globular (red) population of all 31 C_α_ atoms can be seen. The vertical lines display a confidence interval of ±2*σ*. (c) The conformational sensitivities of all C_α_ atoms are displayed as a box plot. The majority of C_α_ atoms show a conformational sensitivity of below 3.29*σ*. Some shifts are more sensitive, with the highest sensitivity at just over 8*σ*.

For each evaluated atom, five chemical-shift values for both the stretched and globular conformation were calculated, respectively. Using the two times five samples of the observable, two Gaussian shaped probability distributions were obtained (Fig. 4a). It was assumed that the standard deviations of the two distributions were similar, thus allowing the calculation of a pooled standard deviation. Each of the two distributions has one expectation value and the difference between them, in multiples of the pooled standard deviation, is regarded as the sensitivity of the chemical shift to changes in the overall conformation. The chemical shift is regarded as conformationally sensitive if the overlap of the two probability distributions is less than 10%, which is equal to a difference in expectation value of 3.29*σ*. This process was repeated with all tested atoms (Fig. 4b).

The conformational sensitivities were categorized – regarding their atom of origin – into seven groups: The backbone atoms C_α_, C_carbonyl_, H_amide_ and N_amide_ as well as atoms from side-chains C_β_, C_other_, H_other_ and N_other_. For each of the groups, the conformational sensitivity (difference in chemical shift expectation value counted in pooled *σ*) is displayed as a box plot in Fig. 4c. The cutoff of 3.29*σ* is presented as a dashed horizontal red line. Chemical shifts which originated from the capped end groups as well as from oxygen atoms were excluded from further analysis as it is uncommon for them to be measured experimentally and are thus rarely used for ensemble reweighting.

In addition to the chemical sensitivity, the agreement with experimental data was evaluated to assess if any systematic biases are observed. If chemical shifts predicted for any conformation are systematically under- or over-estimated with respect to the experiment, a reweighing of the ensemble becomes difficult. The experimental chemical shifts were obtained from the study by Lasorsa *et al.*^25^ in which they were measured at 600 MHz, 5 °C and pH 7.3. Backbone assignment was performed using triple-resonance solution state NMR experiments. Sidechains were assigned using additional 3D NOESY and TOCSY experiments. The mean relative error (eqn (3)) was chosen as a metric of compliance with the experiment.3
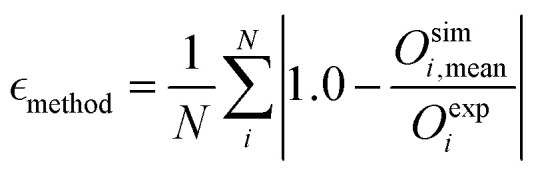
where, 
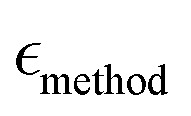
 is the mean relative error of the tested method, *O*^exp^_*i*_ is the experimental measurement of the observable *i*, and *O*^sim^_*i*,mean_ is the simulated mean of observable *i*.

To rule out a significant share of non-random coil secondary structure in the tested polypeptide, DSSP analysis^53^ using MDAnalysis as well as the secondary chemical shifts were analyzed. The secondary chemical shift is defined as the difference between the random coil chemical shift and the measured chemical shift of the same atom (Δ*δ* = *δ*_obs_ − *δ*_rc_).^54^ To follow the more modern convention used in key publications,^16,17,55^ the sign of the equation has been inverted compared to in the study by Dalgarno *et al.* Due to the contrasting behavior of C_α_ and C_β_ secondary chemical shifts with respect to stable secondary structures,^17^ they can be subtracted^56^ to create the secondary structure identifier ΔΔ*δ*_αβ_ = Δ*δ*_C_α__ − Δ*δ*_C_β__. A positive ΔΔ*δ*_αβ_ indicates an α-structure, while a negative ΔΔ*δ*_αβ_ indicates a β-structure.^17,26^ To calculate the random coil chemical shifts the POTENCI software^57^ was used. The settings were chosen as pH = 7.3, *T* = 293 K and ionic strength = 0.13 mol L^−1^ to represent the experimental conditions as closely as possible.

## 3 Results

### 3.1 Conformational sensitivity

**Fig. 5:**
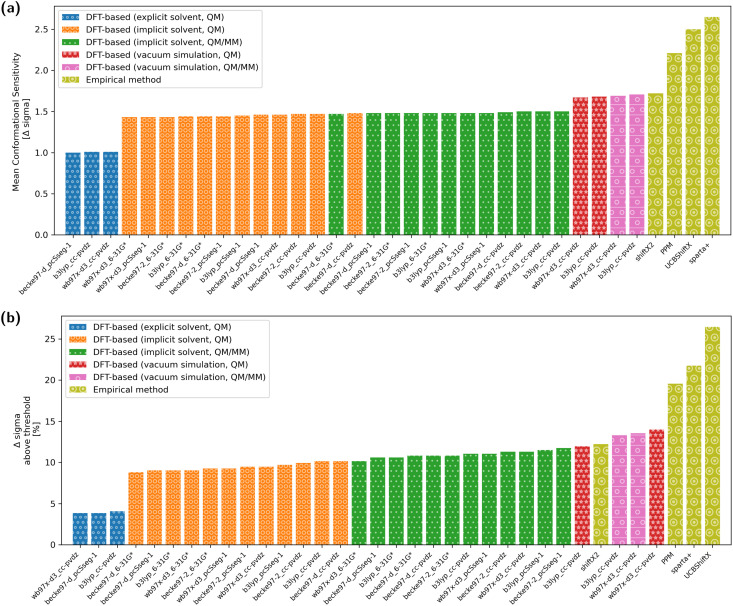
Measurements of the mean conformational sensitivity (a) as well as the share of chemical shifts above the threshold (b) show that three empirical methods (PPM, SPARTA+ and UCBShiftX) are more sensitive to conformational change compared to DFT-based methods. It has to be noted, that there is no experimental reference and the true value is unknown. It is expected, that a higher value represents a more sensitive model.

The overall conformational sensitivity of all approaches is summarized in Fig. 5. The sensitivity in terms of mean chemical shift difference between the stretched and globular conformations (in measures of Δ*σ*) is shown in Fig. 5a while Fig. 5b shows the percentage of chemical shifts that is regarded as conformationally sensitive. While both metrics confirm that the overall sensitivity of chemical shifts with respect to conformation is rather limited, the empirical predictors UCBShiftX, SPARTA+ and PPM were found to be the most sensitive. The SHIFTX2 predictor performed equally to the DFT-based methods calculated in vacuum. DFT-based calculations, both QM and QM/MM, with implicit solvation differentiated very little depending on the choice of functional and basis-set. The introduction of micro-solvation as an explicit solvent model removes most of the remaining conformational sensitivity.

**Fig. 6:**
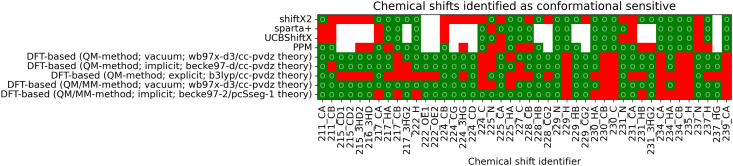
The matrix shows whether a chemical shift is predicted to be conformationally sensitive by each of the nine compared methods. A green mark (○) represents a conformational sensitive chemical shift while a red mark identifies a non-sensitive shift. If a chemical shift has not been calculated, it is marked as white. Chemical shifts with at least four out of the nine methods agreeing on conformational sensitivity are shown. A complete overview of all 497 shifts can be found in the ESI,† Chapter 1.2.

To reweight a conformational ensemble into agreement with experimental data, it is required that the observable of interest is sensitive to the overall molecular conformation. While reweighting algorithms may be able to disregard some insensitive data that does not give information about the conformation, we emphasize that a major share of chemical shifts in these calculations seems insensitive. It may, hence, be necessary to find a sub-set of chemical shifts that contains most of the information in regard to the conformational state. We investigated whether the four empirical methods and the DFT-based predictions with a selected functional/basis-set combination can find agreement regarding the conformational sensitivity of chemical shifts. Fig. 6 shows whether the methods regard individual chemical shifts as conformationally sensitive or not. A green mark represents a sensitive chemical shift, while a red one is expected not to be sensitive. Out of the total 497 chemical shifts, there are 42 cases with at least four of nine predictions agreeing on the chemical shift to be conformational sensitive. Another two sensitive cases are chemical shifts of oxygen atoms, which are not considered for further analysis.

#### 3.1.1 Relationship of atom category and conformational sensitivity

**Fig. 7:**
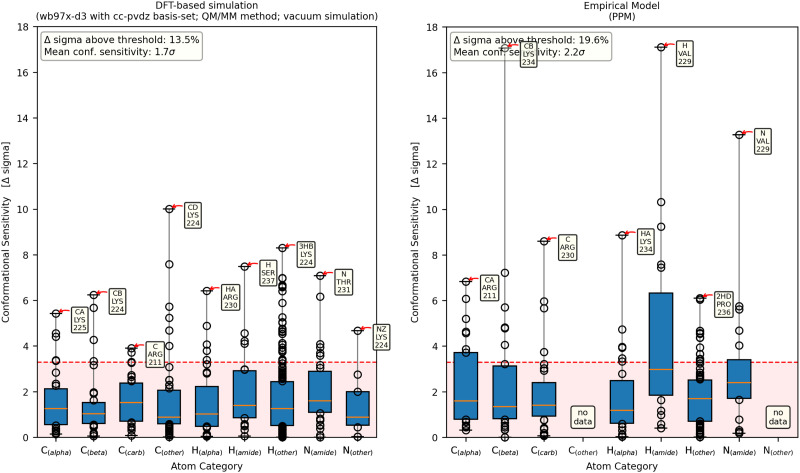
Each column shows the conformational sensitivity of an atom category. On the *x*-axis, seven atom categories can be viewed, to check whether some atoms are more prone to sensitive chemical shifts than others. The *y*-axis shows the conformational sensitivity as described in Fig. 4a. The orange line of each box represents the median value while the lower and upper edges represent the end of the first and third quartiles, respectively. Data points outside of that range are displayed as dots with the largest being annotated for each atom category. The left panel shows a representative result from a DFT-based calculation while the right panel shows results obtained with the empirical method PPM.

Fig. 7 shows the conformational sensitivity of the seven atom-categories. Visual assessment of the plots generated from DFT-based calculations with implicit solvent and from vacuum simulations, as well as half of the empirical predictors, seem to show minor increased median sensitivity on the amide-nitrogen and the accompanying proton compared to the other atom categories. Another common theme of the DFT-based calculation is increased maximum sensitivities of some side-chain atoms. The choice of functional and basis leads to only few noteworthy differences in conformational sensitivity (comparisons are given in the ESI,† Chapter 1.4).

The empirical models PPM, SPARTA+ and SHIFTX2 agree on the slightly increased sensitivity of the amide-hydrogen (Fig. 7 (right) and Fig. S4.3 and S4.4, ESI†). In contrast, UCBShiftX evaluates C_α_ and the amide-nitrogen atoms to contain the most conformational information (Fig. S4.2, ESI†). Most empirical chemical shift predictors show less data as they are not designed to calculate the chemical shifts of all side chain atoms. Therefore, the effect of the overall conformation on the chemical shifts of sidechains cannot be observed.

#### 3.1.2 Relationship of chemical shift and backbone torsion

**Fig. 8:**
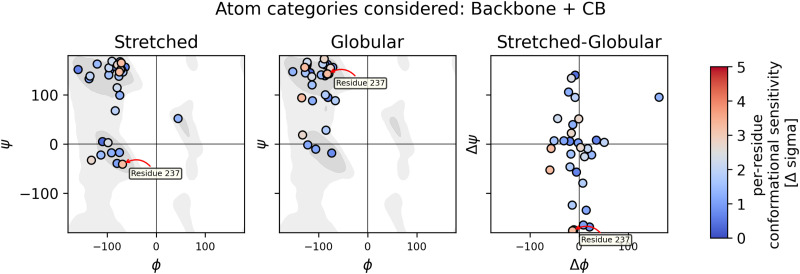
The left and middle columns show Ramachandran plots for both the stretched and globular conformation, respectively. The right column shows the change in *ψ* and *ϕ* angles when switching from the globular to the stretched conformation. The chemical sensitivity is represented in the color of the dots. Conformational sensitivities in this figure were obtained using the DFT-based QM/MM method in vacuum with the wb97x-d3/cc-pvdz theory. The Ramachandran background was plotted using data from ref. 58 and 59.

It can be hypothesized that the change in the chemical shift is related to changes in the *ψ* and *ϕ* angles of the protein backbone. To check this hypothesis, the conformational sensitivities of the residues were plotted against the change in backbone torsion Δ*ψ* and Δ*ϕ* in Fig. 8.

As the conformational sensitivity is a per-atom metric, it has to be transformed into a per-residue metric according to eqn (4). To calculate the average sensitivity of the residue, the sensitivities of all backbone atoms and C_β_ were averaged.4
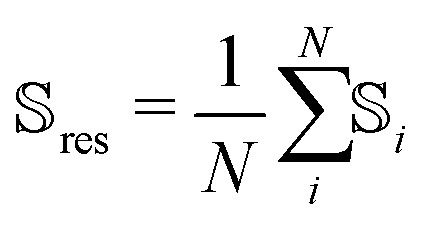
where 𝕊_res_ is the conformational sensitivity of the residue res, 𝕊_*i*_ is the conformational sensitivity of atom *i*, and *N* is the number of single sensitivities averaged into 𝕊_res_.

If, instead of mean sensitivities, the influence of Δ*ψ* and Δ*ϕ* on specific atoms is of interest, the residue sensitivity can be set equal to the sensitivity of that specific atom (𝕊_res_ = 𝕊_i_). Plots for C_carb_, C_α_, C_β_, H_amide_ and N_amide_ atoms as well as the remaining methods can be found in the ESI,† Chapter 1.5.

In the case of the DFT-based QM/MM method with wb97x-d3/cc-pvdz theory, residue 237 shows the highest sensitivity and has also a significant change in *ψ* angle. Two more residues show sensitivity and have a significant change in *ϕ* angle. On the other hand, there are plenty of residues that show changes in the angles but no sensitivity.

#### 3.1.3 Random forest analysis

In the previous sections the visual analysis of the influence of dihedral angles and atom category on the conformational sensitivity was assessed to be very minor with unclear statistical significance. To evaluate methodically if there is any feature that is related with a chemical shift being conformational sensitive or not, a permutation feature selection has been performed. Table 2 shows both geometrical and biochemical features that can be expected to influence the chemical sensitivity. As label, the conformational sensitivity was chosen.

**Table 2:** Overview of the features tested to evaluate their influence on the conformational sensitivity of chemical shifts. The atom of interest is the atom for which the conformational sensitivity is evaluated

Feature	Annotations
Atom category	Categorical value according to the groups in Fig. 7.
Atom name	Categorical value according to the name of the atom.
Atom element	Categorical value according to the element of the atom.
Residue name	Categorical value according to PDB residue name.
Residue number	Number of the associated residue in the peptide sequence.
Δ-distance atom (*)	Change in distance from the atom of interest to the next closest atom of type (*) associated with a residue at least three amino acids away from the selected atom. Atom (*) can either be oxygen (O), nitrogen (N) or the center of geometry of the residue (RES).
Δ*ψ* and Δ*ϕ* angle	Change in backbone dihedral angle.
ΔSASA	Change in the solvent accessible surface areas per atom between stretched and globular conformation. Calculated with FreeSASA.^60^
Is sidechain?	Categorical value if the atom is part of the backbone or sidechain.
Per-atom alignability	The five equal samples of both the stretched and globular conformation are aligned. The per-atom alignability measures the mean self distance of the atoms to their own copies.
Negative control (continuous)	Uniform random number (float) between 0.0 and 20.0.
Negative control (categorical)	Categorical random number (int) between 0 and 6.

To train the random forest regressor,^61^ categorical features like atom-category and residue-name had to be converted into representative, numerical dummies using one-hot or ordinal encoding. While one-hot-encoding is expected to yield higher quality results, it is difficult to reverse the encoding to obtain the importance of whole feature categories. To evaluate whole features, for example the importance of the amino acid type, ordinal encoding was applied. To evaluate the importance of single elements within a feature, to answer for example if proline or valine amino acids are related with conformational sensitivity, one-hot encoding was used.

The random forest was trained on the features, using a 80/20 split between training and testing data, 100 trees and squared error as a measurement of the split quality. To prevent overfitting on the training data, *min_samples_leaf* was set to 10, *min_samples_split* to 15 and *max_depth* to 10. To measure the quality of the regression, artificial control features were added in addition to the geometrical and biophysical features. The two negative controls were always unrelated to the conformational sensitivity and were added to confirm the validity of the method. For each regression, the *R*^2^-score was calculated. After building the model, single features were randomly shuffled to calculate a permutation feature importance. If a feature has influence on the model, shuffling it will reduce the *R*^2^-score significantly. The features that yield the biggest loss if shuffled, are regarded as the most important. The process was repeated 50 times with different seeding of the random number generator to create a set of models. Single trainings, which yielded a negative coefficient of determination (*R*^2^) on the testing data, were removed from the set. The remaining feature importance values and model scores of the set were then averaged. Overall, only weak relationships were found with testing *R*^2^-scores up to 0.12 in the case of DFT-based calculations (Fig. S6.23, ESI†).

**Fig. 9:**
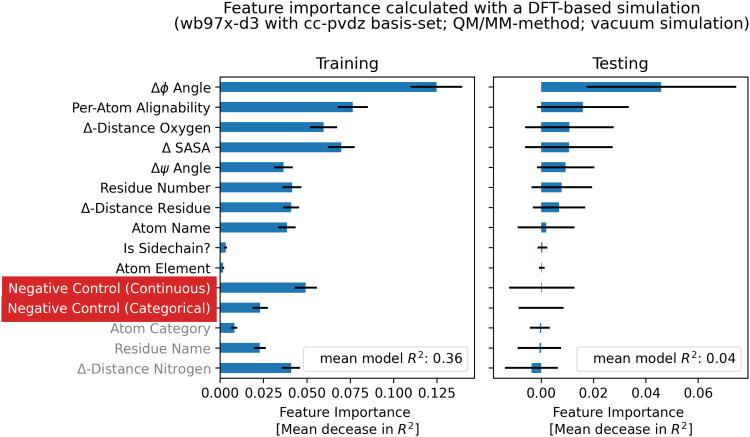
Permutation importance of geometrical and biophysical features in regard to the conformational sensitivity of chemical shifts show that the Δ*ϕ*-angle is of importance to predict whether a chemical shift is sensitive to conformational change or not. An error bar represents ± one standard deviation.

Feature importances obtained from DFT-based calculations with different functional/basis-set combinations show comparable results. Fig. 9 shows feature importances obtained with the wb97x-d3/cc-pvdz theory, demonstrating that the atom-category and element is not of importance whether a chemical shift is conformational sensitive or not. The most important feature that has an influence is the Δ*ϕ* angle, the per-atom alignability of the five replicas per conformation and the change in distance to an oxygen atom. The finding of increased importance of the Δ*ϕ* dihedral angle was also confirmed by feature importance calculations using the empirical methods (Fig. S6.1–S6.3, ESI†).

### 3.2 Agreement between simulation and experimental results

Not only is the sensitivity in regard to conformational change relevant, but so is the absolute agreement with experimentally obtained values. Due to the liquid nature of the NMR sample and the measurement time of the NMR methods used, it must be assumed that the experimental chemical shifts are averages of all accessible conformations. Therefore, the experimental chemical shifts have to be compared with simulated observables calculated from a reasonably complete conformational ensemble. A set of two conformational extreme cases, as discussed here, does not constitute a complete conformational ensemble so it is not expected that the average matches the experiment exactly. In order to properly validate the predicted chemical shifts against the experimental values, much more extensive simulations would be required, and the chemical shifts would need to be computed to all relevant conformations. Still, systematic over- or under-prediction of chemical shifts can be assessed using an incomplete ensemble and should be avoided.

**Fig. 10:**
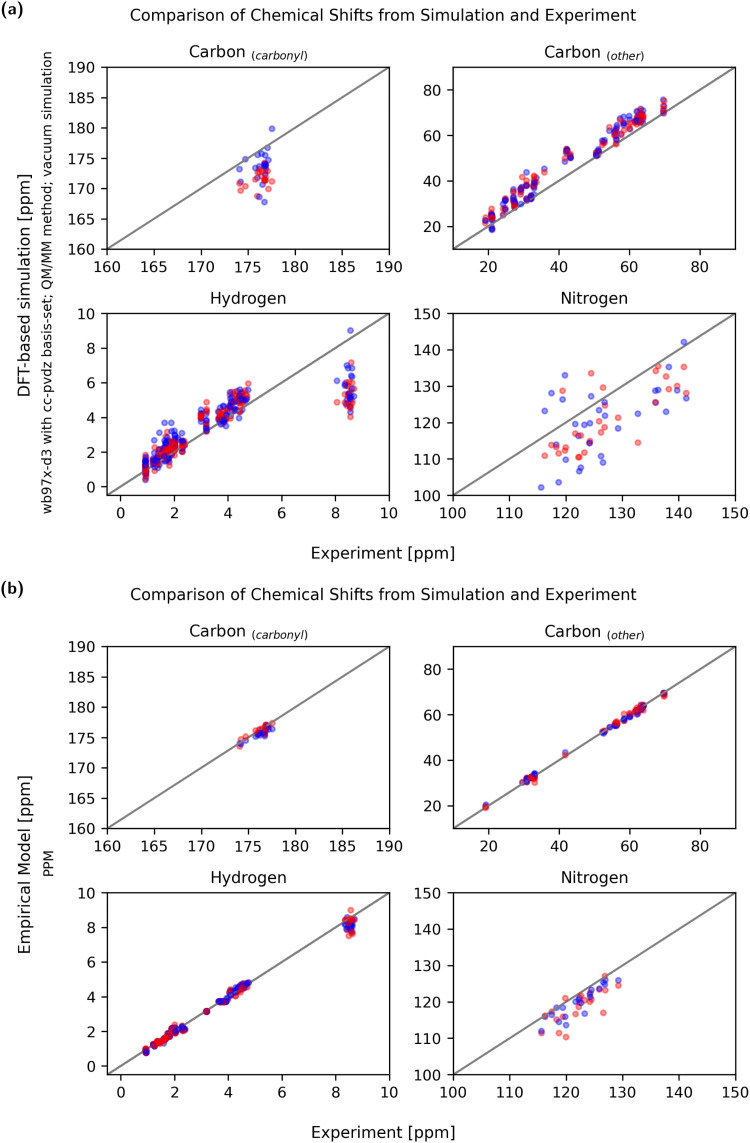
A comparison of the experimental chemical shifts (*x*-axis) and simulated chemical shifts (*y*-axis) allows an overview of the prediction. The diagonal gray line represents a perfect agreement between experiment and simulation, the blue dots indicate chemical shifts of the stretched conformation and the red dots indicate chemical shifts of the globular one. (a) The results obtained with DFT-calculations using the QM/MM method in vacuum using the wb97x-d3/cc-pdvz theory. Aliphatic carbons and hydrogen show a good quality of prediction, whereas the chemical shifts of amid protons is too low. (b) Chemical shifts calculated with the PPM software show very good agreement with the experiment both for the stretched as well as for the globular case.

**Fig. 11:**
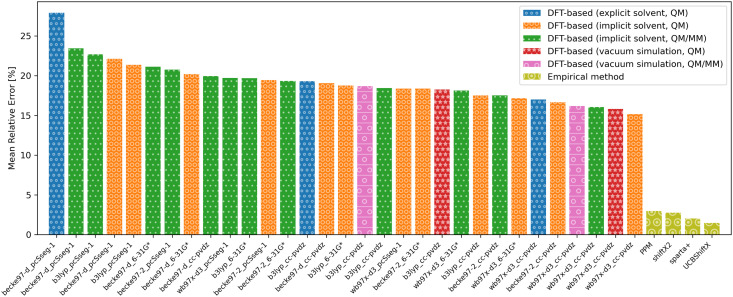
Overview of the mean relative error of the different approaches to predict chemical shifts. DFT-based methods achieved the best accuracies using the wb97x-d3 functional and cc-pdvz basis-set.

Fig. 10 shows a comparison between experiment and simulation for both a DFT-based QM/MM calculation (wb97x-d3/cc-pdvz) in vacuum and an empirical (PPM) prediction method. To compare the accuracy of the methods, the mean relative error of the simulation compared to the experiment was calculated using eqn (3) and is shown in Fig. 11.

Even though the empirical methods show the strongest conformational sensitivity, the accuracy is also remarkably good. While the conformational sensitivity showed little dependence on the choice of functional and basis set, the influence on accuracy is slightly greater. Using DFT-based methods, the best accuracies were achieved using the wb97x-d3 functional and cc-pdvz basis-set independent of the fragmentation and solvation method.

### 3.3 Secondary chemical shifts

**Fig. 12:**
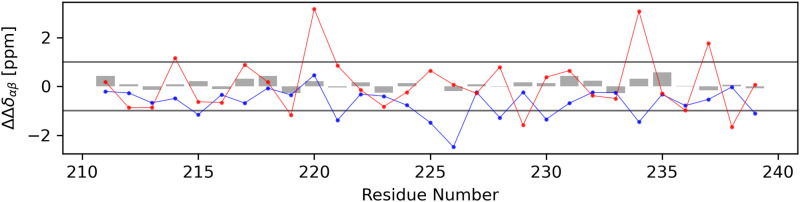
The calculation of a secondary structure identifier ΔΔ*δ*_αβ_ gives indications about the occurrence of secondary structures. To differentiate between random coil and non-random coil secondary structures, literature advises a secondary chemical shift of |Δ*δ*| > 0.7 which is more or less continuous for more than four residues.^55^ As ΔΔ*δ*_αβ_ is the difference between two secondary chemical shifts, which are expected to be additive in behavior, a threshold of 1.0 has been set arbitrarily and marked as a horizontal line. Gray bars represent data obtained from the experiment, blue from predictions for the stretched conformer and red from the globular conformer.

For each non-terminal residue, a secondary structure identifier ΔΔ*δ*_αβ_ was calculated using secondary chemical shift data. In Fig. 12, the calculated secondary structure identifier values for the experimental dataset (gray bars) and both the globular (red dots) and stretched (blue dots) conformations can be seen. The experimentally obtained data show no indication that either alpha- or beta-structured conformers make up a significant share of the ensemble, a finding supported by the DSSP analysis of the molecular dynamics trajectory (Fig. S3.1, ESI†). Applying the same secondary chemical shifts analysis to both the stretched and globular conformers with data obtained from the UCBShiftX method, stronger derivations from the random coil can be observed. The globular conformer shows mostly slightly higher secondary structure identifier values but the majority of data points for both conformers remain in the region attributed to the random coil. It should be mentioned that secondary chemical shift based analysis is very sensitive to systematic offsets of chemical shift prediction and measurement, as it is a comparison with tabulated random coil chemical shifts. Thus, it may be easy to wrongly declare parts of the peptide to be either alpha- or beta-structured. Therefore, only simulated data from the UCBShiftX method is discussed here, as the smallest mean relative error to the experiment was observed with this method.

## 4 Discussion

### 4.1 Conformational sensitivity

The results of the calculations show that the conformational sensitivity is mainly limited by the precision of the estimator. Even small differences between the five equally treated overall conformations (Fig. 1) yield different chemical shifts with the DFT-based methods. This results in wide probability distribution peaks and thus weak conformational sensitivities measured as multiples of pooled standard deviation. The mean probability density function width of all methods can be seen in the ESI,† Chapter 1.1 which shows a significant difference between DFT-based and empirical methods. The use of explicit solvent leads to an even stronger decrease in conformational sensitivity. With explicit solvent, all five replicas of the same overall conformation feature micro-solvation with water molecules at different positions and orientations, taken from the MD-trajectory. Wide probability distributions can be observed especially with side chain atoms and protons, thus weakening the distinction between the expectation values of the stretched and globular group.

Regarding DFT-based prediction methods, the tested QM/MM approach which models protein parts in greater distance as MM-region show slightly better conformational sensitivity compared to the QM approach but the difference is small. A slightly bigger improvement was made when replacing the implicit solvent with a vacuum simulation for both the QM and QM/MM cases. It has to be noted that the vacuum simulations showed weaker convergence behavior, with some functional/basis-set combinations (Becke97-2 and Becke97-D functionals with the pcSseg-1 basis-set) unable to yield converged shieldings tensors for some atoms.

Empirical predictors yield results that are much closer to the experimental average for both the stretched and globular conformation and the influence of conformation on the absolute value of the chemical shift is much smaller than with the DFT-based calculations. Nevertheless, the results are more capable of differentiating between the two conformations.

### 4.2 Influence of features

Visual interpretation of the influence of atom categories and backbone torsion on the conformational sensitivity could only partially be reproduced with random forest permutation feature selection. It was not possible to find a relationship between atom-category and conformational sensitivity but the chemical shift prediction models could find agreement that a change in an amino acid's *ϕ* angle has influence on the conformational sensitivity of chemical shifts. It has to be mentioned that all four empirical models have been parameterized using the *ϕ* angle or a derived property as input feature. Nevertheless, the influence of the Δ*ϕ* angle was also witnessed using *ab initio* methods in vacuum and with implicit solvent.

The *R*^2^-scores of the regression models were shown to be weak, and only slightly better than those of a constant model. Many evaluated biophysical features were shown to be unrelated to the predicted conformational sensitivity. Still, the results of the feature importance are consistent over the methods. It has to be noted, that the random forest had to be trained on a very small data-set while the feature space was wide. The weak regression scores also explain the difficulties with visual interpretation of the results and confirms the necessity to perform a similar study with a bigger set of proteins to conclude whether the findings can be generalized.

### 4.3 Agreement between experiment and simulation

When comparing simulated and experimental chemical shifts to assess the quality of prediction, it has to be taken into account that experimental chemical shifts are an averaged property of a molecular ensemble. Thus, simulated chemical shifts must be calculated using a reasonably complete conformational ensemble. As this is not the case when using only two conformational extreme cases, it is not unexpected that the predicted chemical shifts of single conformations can show deviations from the experimental means.

In the ideal case, chemical shifts calculated from single conformations can be found to be clustered around the experimental mean. In practice, most DFT-based calculations show either a systematic under- or over-prediction of values (example: Fig. S8.14, S8.22 and S8.34, ESI†). The error can not only be explained by faulty single-point referencing, as chemical shifts of aliphatic carbons are often predicted to be slightly too high while carbonyl carbon chemical shifts may be too low using the same referencing. As publications from Rablen *et al.* and the Tantillo group show,^62,63^ it may be necessary to reference chemical shifts calculated using the DFT-based method not just by one single reference point (intercept) but also to calculate a scaling factor. The choice of a fitting basis-set is key to minimizing these systematic offsets, so that predicted values are evenly clustered around the experimental means (Fig. 10a).

With DFT-based approaches using vacuum simulations or implicit solvent, the absolute chemical shift values of the *H*_amide_ atoms were constantly underpredicted, even when other shifts were systematically overpredicted. The addition of discrete water molecules as a micro solvent helped to improve these absolute values and the error due to underestimation could be considerably reduced.

Compared to the DFT-based methods, empirical predictors do an excellent job reproducing experimental chemical shifts. There are no obvious systematical errors and both chemical shifts of the stretched and globular conformation match the experimental average very well.

## 5 Conclusions

This study explored different methods to calculate chemical shifts of proteins and their sensitivity in regard to protein conformation. Judging by the results of the evaluated test system, the majority of the chemical shifts are expected not to be sensitive to changes in overall conformation. We find that many chemical shifts that are predicted for very similar configurations, which would generally be considered as being in the same conformation, actually differ in a similar amount as chemical shifts predicted for two really distinct conformations. It should be noted that the TAU-protein fragment evaluated in this case study is unlikely to feature a significant secondary structure, neither in the experimental data nor observed in the simulation or in the selected conformers. While it is very possible that the choice of this fragment makes conformational differentiation using chemical shifts more difficult, it has to be expected that such regions showing no major secondary structure propensities occur often in the context of ensemble reweighting.

There is likely no relationship between atom type, atom name and element with regard to the conformational sensitivity of the chemical shift. Up to ∼26% of the calculated chemical shifts (UCBShiftX software) show conformational sensitivity in the case of the tested peptide but it remains difficult to predict why exactly those are sensitive while others are not. Compared to established chemical shift-based structure elucidation methods targeting conformations with stable secondary structures, particular attention must be paid to only select data that offers information about the conformation with reasonable probability when working with IDPs.

When comparing empirical methods with DFT-based ones, the most obvious difference is the efficiency and compute-time needed to fulfill the task. Empirical methods remain orders of magnitude faster than DFT-based calculations. While most of the empirical chemical shift predictors were designed for and trained by globular proteins, they are still capable of including most conformational sensitivity in the predicted chemical shifts in this case study.

Taking efficiency, time spent and unmatched accuracy into account, empirical predictors will remain the method of choice for most researchers to calculate chemical shifts even if they can only be applied to a subset of atoms.

## Author contributions

JS conceptualized and managed the design and methodology of the project, implemented the necessary scripts, ran the calculations and wrote the manuscript. CO acted as the supervisor, organized the funding of the project, and edited and reviewed the manuscript. All authors have read and agreed to the published version of the manuscript.

## Data availability

Data supporting the findings of this study are available at: https://zenodo.org/doi/10.5281/zenodo.11086149. The archive contains the 2 × 5 evaluated structures of the tested TAU-fragment, scripts to set-up DFT-based and empirical calculations and data analysis for all tested methods.

## Conflicts of interest

The authors declare that there are no conflicts of interest.

## Supplementary Material

CP-026-D4CP02484B-s001
