# Supplementary material for: Conformational dependence of chemical shifts in the proline rich region of TAU protein
Source: Phys Chem Chem Phys. 2024 Aug 30;26(36):23856–70. doi: 10.1039/d4cp02484b (PMC11373535; doi:10.1039/d4cp02484b)
Supplement: CP-026-D4CP02484B-s001 [file CP-026-D4CP02484B-s001.pdf]

Supplementary Information (SI) for Physical Chemistry Chemical Physics.
This journal is © the Owner Societies 2024

Supplementary Materials

Conformational Dependence of Chemical Shifts in the Proline Rich Region of TAU Protein

Johannes STÖCKELMAIER and Chris OOSTENBRINK
Institute of Molecular Modeling and Simulation (MMS)
University of Natural Resources and Life Sciences, Vienna

July 29, 2024

Contents

1 Supplementary Materials **2**

1.1 Per-Method Mean Width of the Probability Density Functions 2

1.2 Agreement between Methods regarding Conformational Sensitivity of Chemical Shifts 4

1.3 DSSP Analysis of the Molecular Dynamics Trajectory 5

1.4 Influence of Atom Category on Conformational Sensitivity 6

1.5 Relationship of Chemical Shift and Backbone Torsion 43

1.6 Feature Importance using Ordinal Encoding 79

1.7 Feature Importance using OneHot Encoding 98

1.8 Comparison Simulation and Experiment 134

2 References **171**

1 Supplementary Materials

1.1 Per-Method Mean Width of the Probability Density Functions

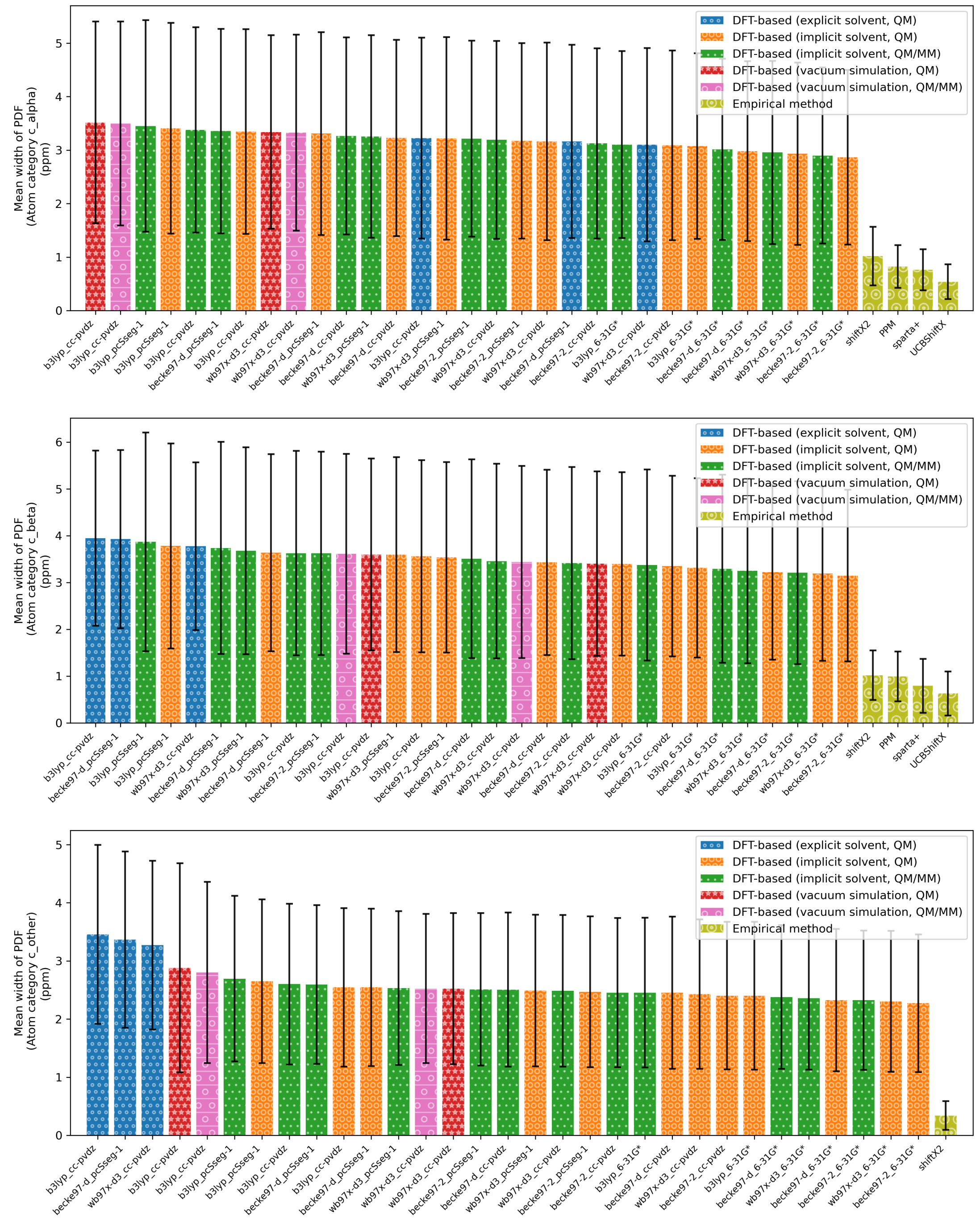

Figure S1.1: Each evaluated atom has two assigned chemical shift probability density functions; one from the five "measurements" of the stretched conformation and one from the five "measurements" of the globular conformation. The mean peak width (FWHM) of those probability distributions from C_α C_β and C_{other} atoms is shown for each calculation method.

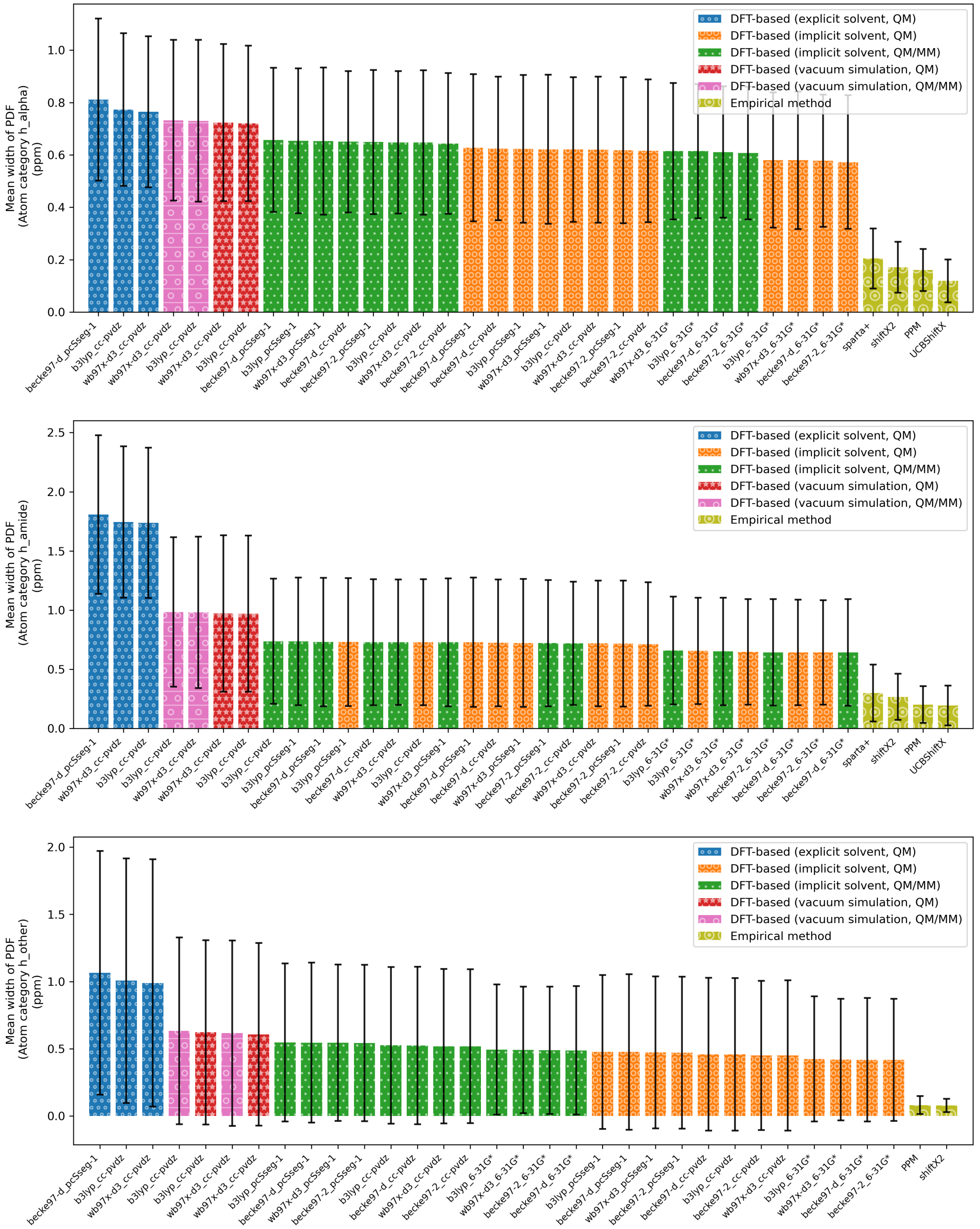

Figure S1.2: Each evaluated atom has two assigned chemical shift probability density functions; one from the five "measurements" of the stretched conformation and one from the five "measurements" of the globular conformation. The mean peak width (FWHM) of those probability distributions from H_α H_{amide} and H_{other} atoms is shown for each calculation method.

1.2 Agreement between Methods regarding Conformational Sensitivity of Chemical Shifts

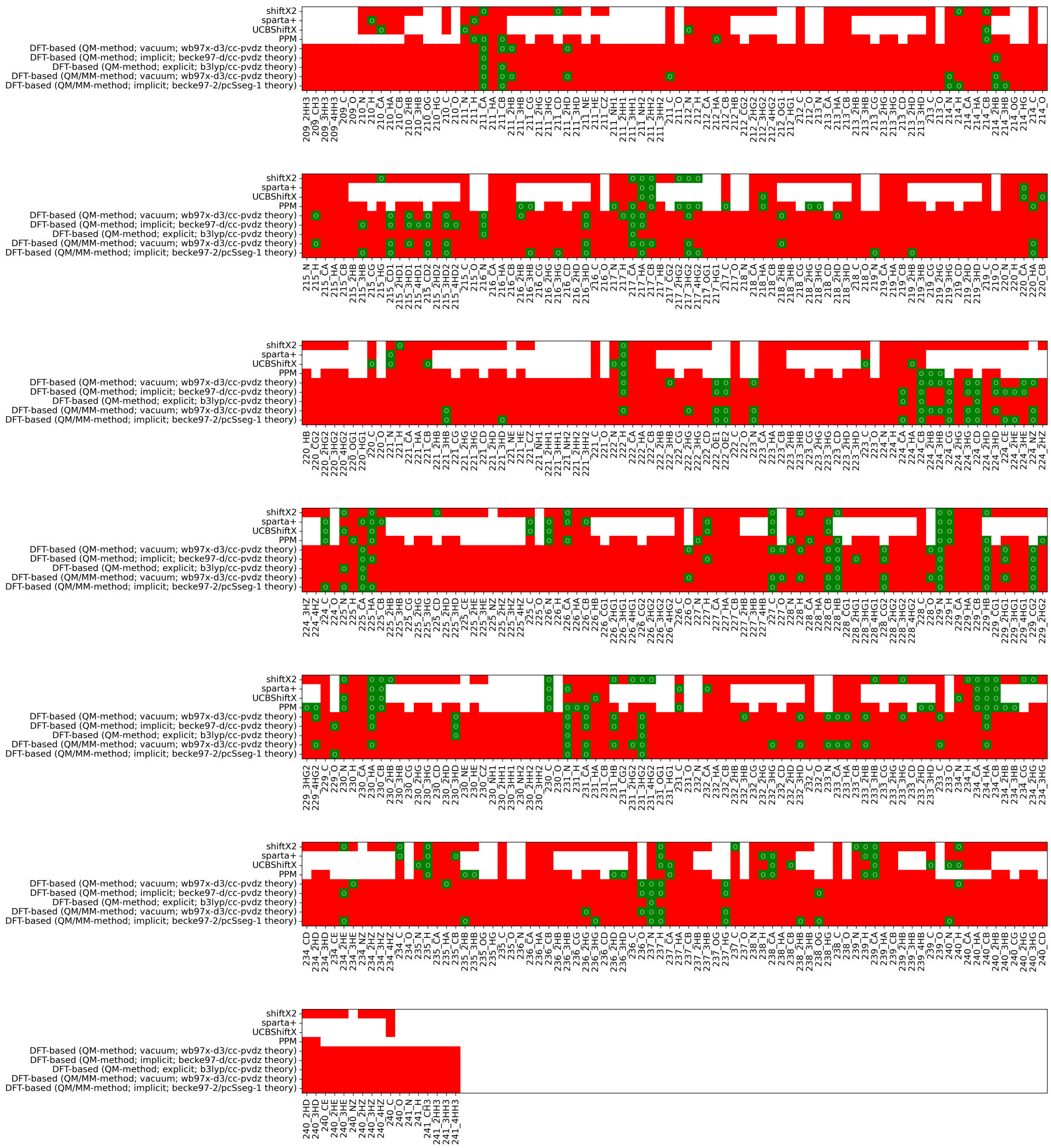

Figure S2.1: The matrix shows whether a chemical shift is predicted to be conformational sensitive by each of the five compared methods. A green mark (o) represents a conformational sensitive chemical shift while a red mark identifies a non-sensitive shift. If a chemical shift has not been calculated, it is marked as white.

1.3 DSSP Analysis of the Molecular Dynamics Trajectory

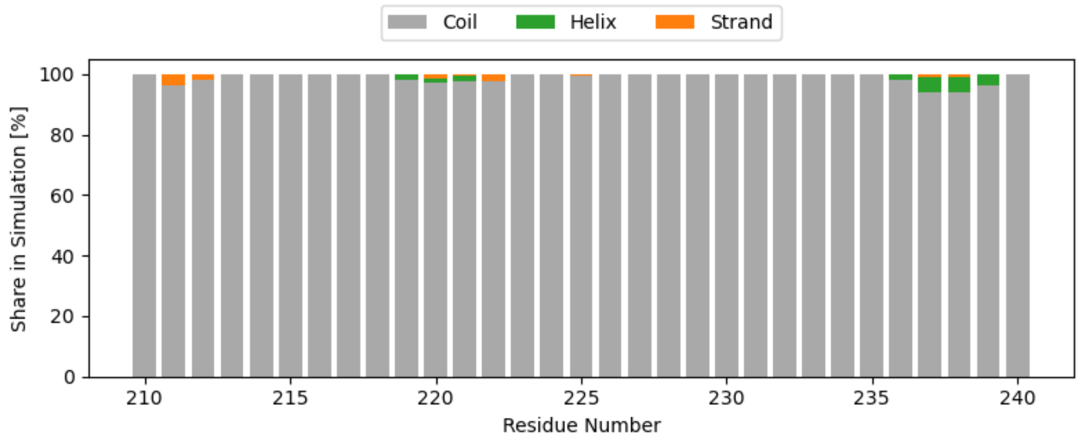

Figure S3.1: The DSSP analysis of the simulated 500 ns trajectory of the TAU-protein fragment shows that neither helix nor strand like secondary structures make up a significant share of the molecular ensemble.

1.4 Influence of Atom Category on Conformational Sensitivity

The conformational sensitivity is defined as the difference of chemical shift expectation value between the stretched and globular conformation. For each evaluated atom one sensitivity was calculated. All atoms were grouped into seven atom categories. If the sensitivity is higher than $3.29\,\sigma$, the chemical shift is considered as conformationally sensitive. The following figures show the conformational sensitivity of each method.

A table of content to find each graphic can be found in table 1.

Table 1: Table of content for the conformational sensitivity of the methods.

Name	Type	Solvation	Figure	Page
PPM	empirical	vacuum	S4.1	8
UCBShiftX	empirical	vacuum	S4.2	9
shiftX2	empirical	vacuum	S4.3	10
sparta+	empirical	vacuum	S4.4	11
b3lyp/6-31G*	QM/MM	implicit	S4.6	13
b3lyp/6-31G*	QM	implicit	S4.6	13
b3lyp/cc-pvdz	QM/MM	vacuum	S4.10	17
b3lyp/cc-pvdz	QM	explicit	S4.8	15
b3lyp/cc-pvdz	QM/MM	implicit	S4.11	18
b3lyp/cc-pvdz	QM	vacuum	S4.10	17
b3lyp/cc-pvdz	QM	implicit	S4.11	18
b3lyp/pcSseg-1	QM	implicit	S4.13	20
b3lyp/pcSseg-1	QM/MM	implicit	S4.13	20
becke97-2/6-31G*	QM/MM	implicit	S4.15	22
becke97-2/6-31G*	QM	implicit	S4.15	22
becke97-2/cc-pvdz	QM	implicit	S4.17	24
becke97-2/cc-pvdz	QM/MM	implicit	S4.17	24
becke97-2/pcSseg-1	QM	implicit	S4.19	26
becke97-2/pcSseg-1	QM/MM	implicit	S4.19	26
becke97-d/6-31G*	QM	implicit	S4.21	28
becke97-d/6-31G*	QM/MM	implicit	S4.21	28
becke97-d/cc-pvdz	QM/MM	implicit	S4.23	30
becke97-d/cc-pvdz	QM	implicit	S4.23	30
becke97-d/pcSseg-1	QM/MM	implicit	S4.26	33
becke97-d/pcSseg-1	QM	explicit	S4.25	32
becke97-d/pcSseg-1	QM	implicit	S4.26	33
wb97x-d3/6-31G*	QM	implicit	S4.28	35
wb97x-d3/6-31G*	QM/MM	implicit	S4.28	35
wb97x-d3/cc-pvdz	QM	explicit	S4.29	36
wb97x-d3/cc-pvdz	QM/MM	implicit	S4.31	38
wb97x-d3/cc-pvdz	QM	implicit	S4.31	38
wb97x-d3/cc-pvdz	QM	vacuum	S4.33	40
wb97x-d3/cc-pvdz	QM/MM	vacuum	S4.33	40
wb97x-d3/pcSseg-1	QM	implicit	S4.35	42
wb97x-d3/pcSseg-1	QM/MM	implicit	S4.35	42

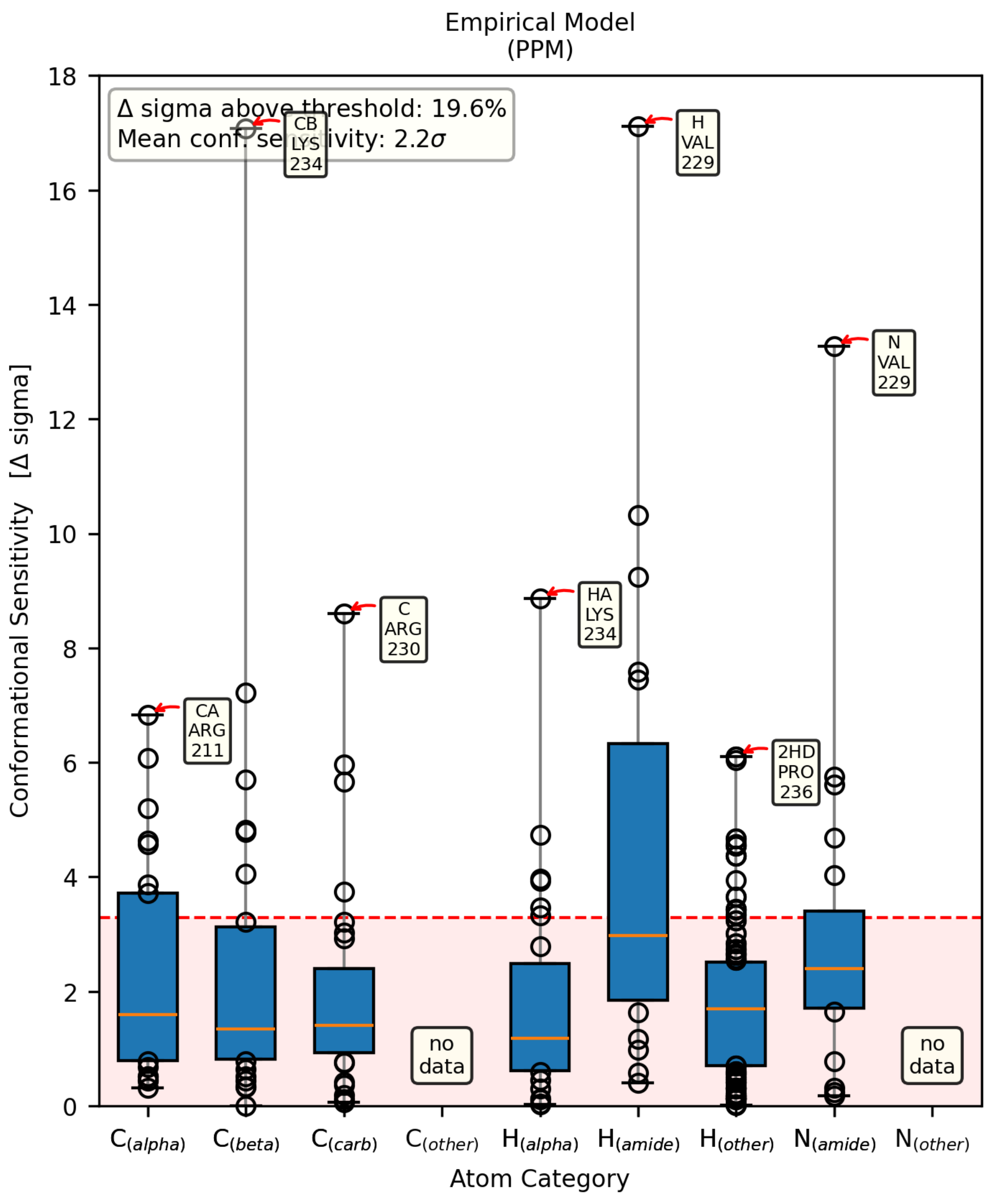

Figure S4.1: Conformational sensitivity calculated with the empirical method PPM.

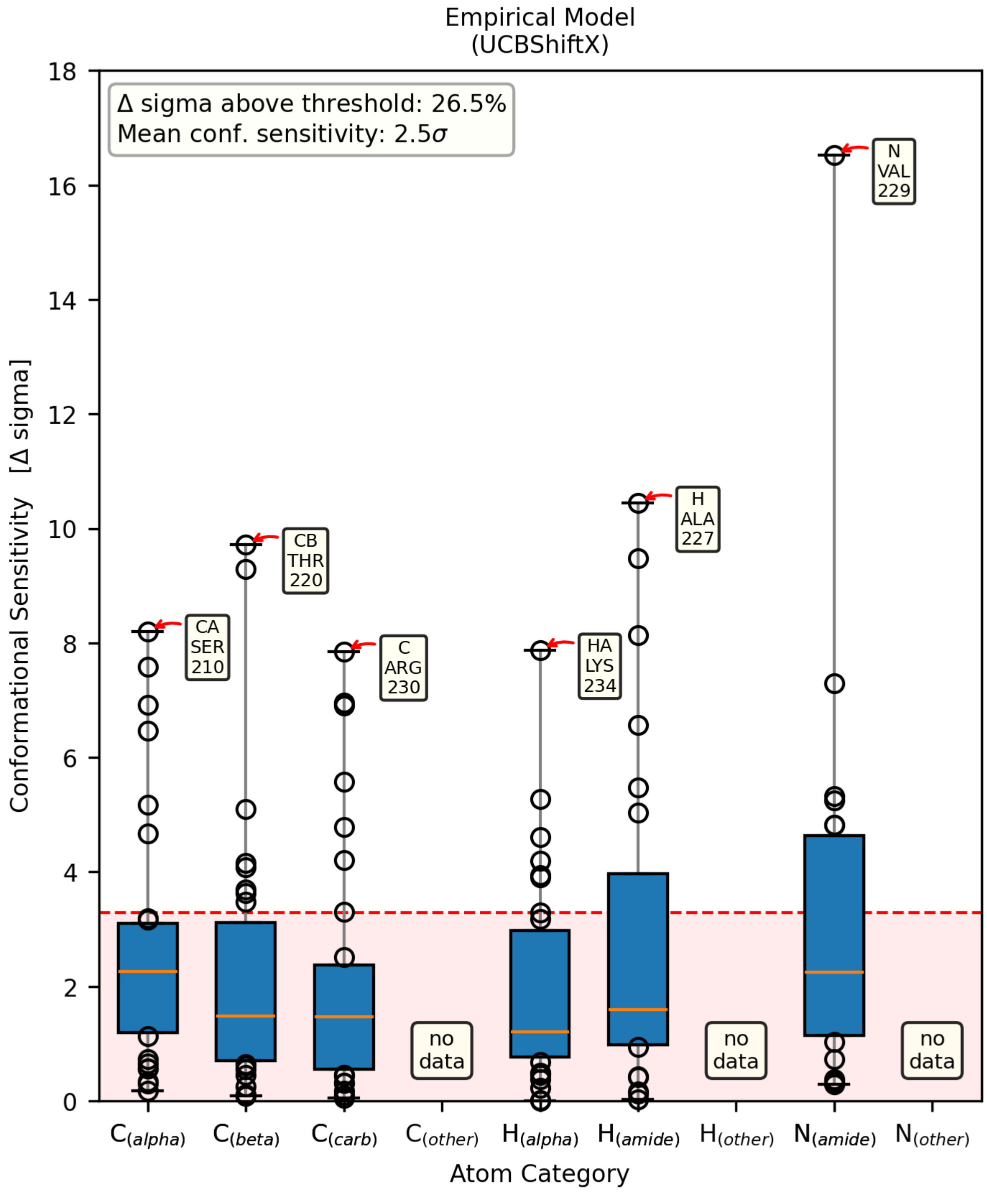

Figure S4.2: Conformational sensitivity calculated with the empirical method UCBShiftX.

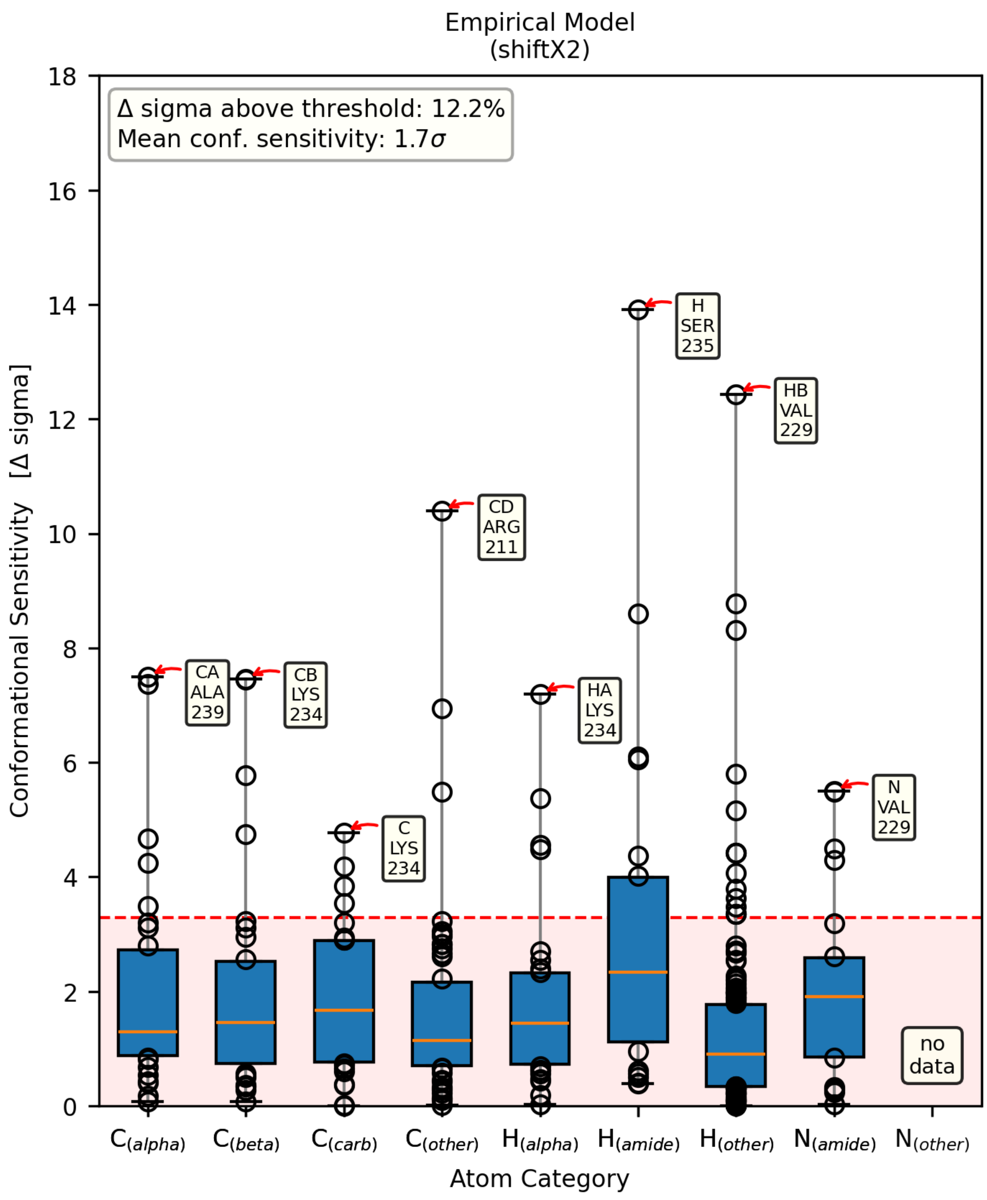

Figure S4.3: Conformational sensitivity calculated with the empirical method shiftX2.

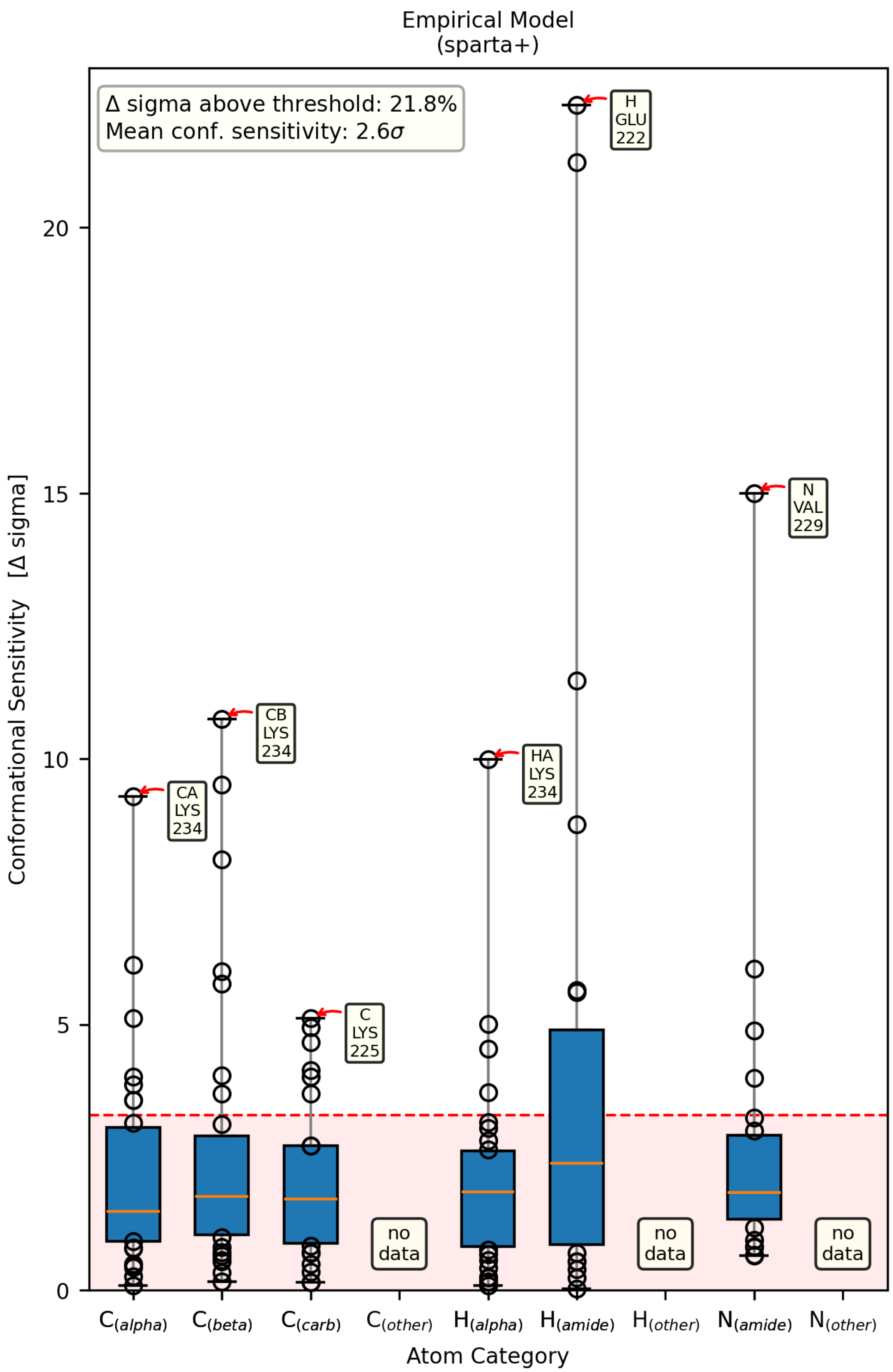

Figure S4.4: Conformational sensitivity calculated with the empirical method sparta+.

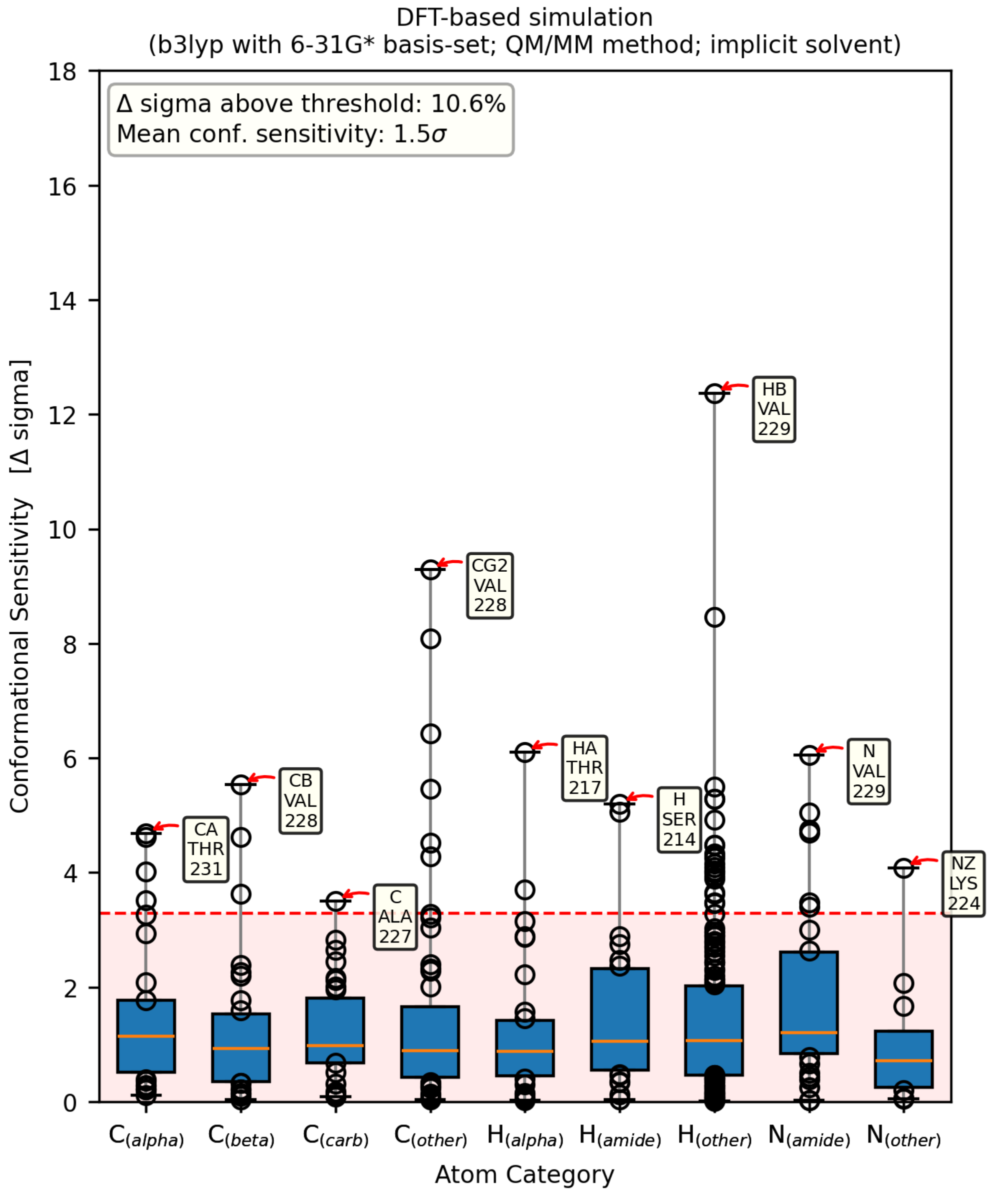

Figure S4.5: Conformational sensitivity calculated with the DFT-based QM/MM method using b3lyp/6-31G* theory with implicit solvent.

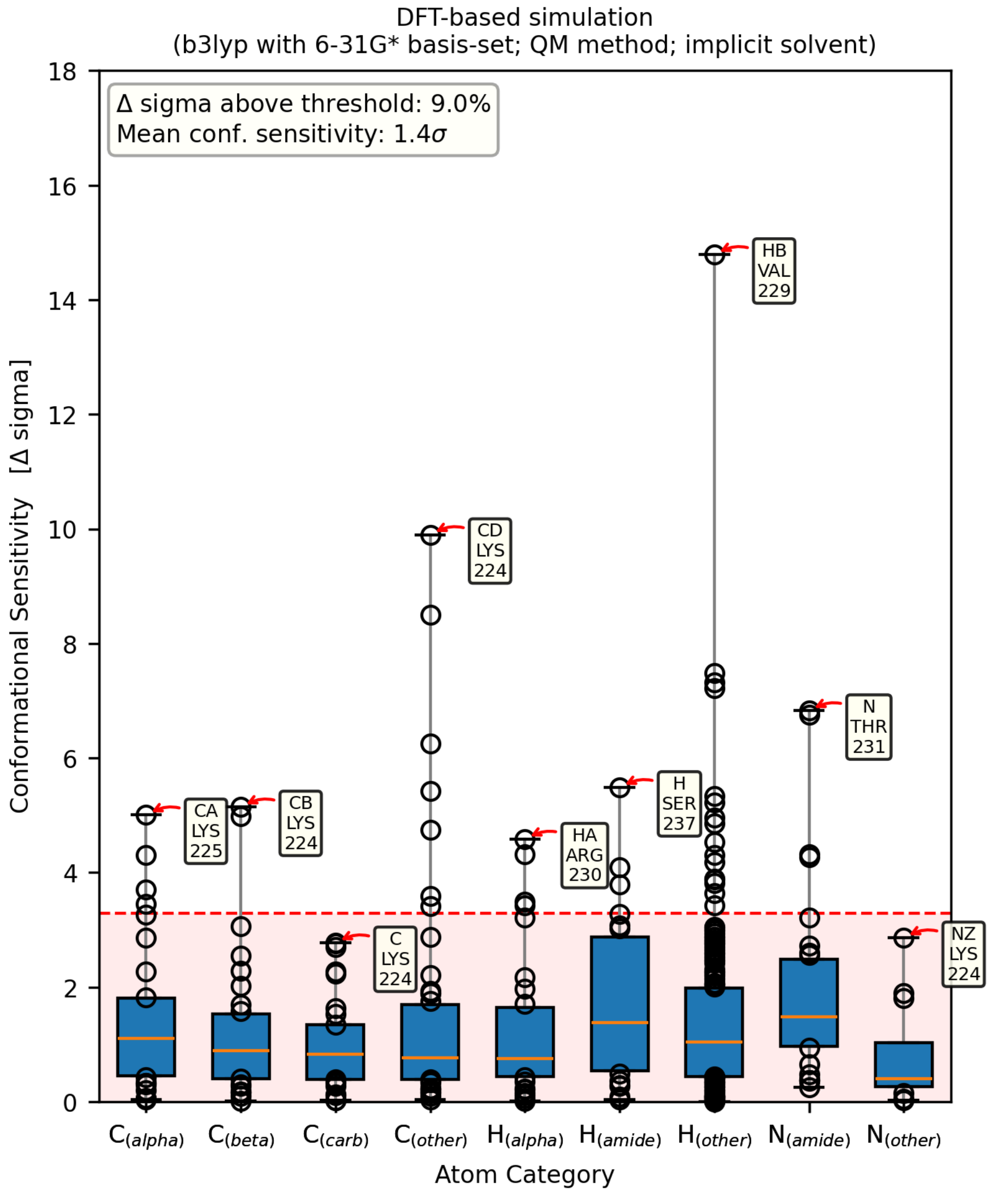

Figure S4.6: Conformational sensitivity calculated with the DFT-based QM method using b3lyp/6-31G* theory with implicit solvent.

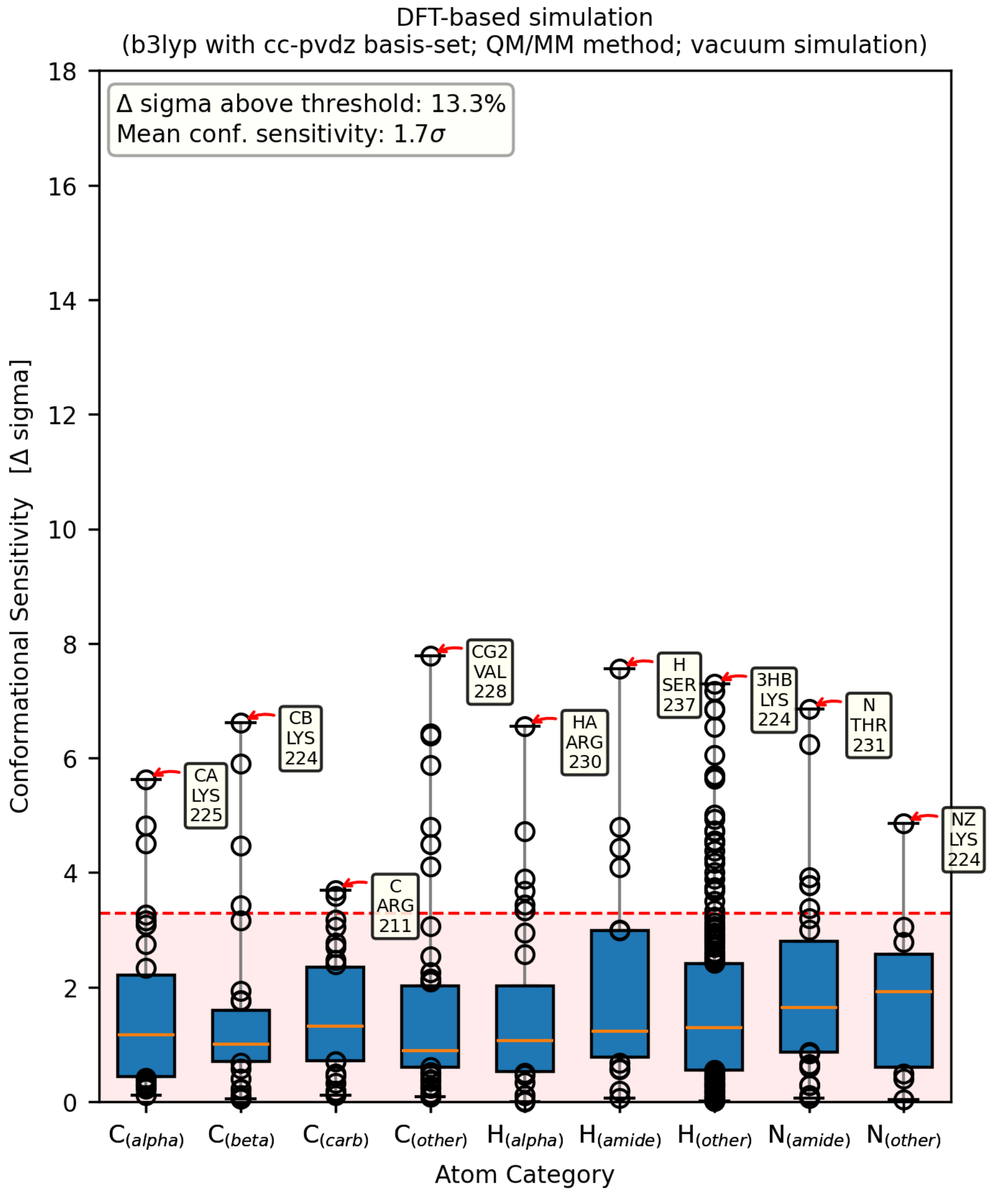

Figure S4.7: Conformational sensitivity calculated with the DFT-based QM/MM method using b3lyp/cc-pvdz theory in vacuum.

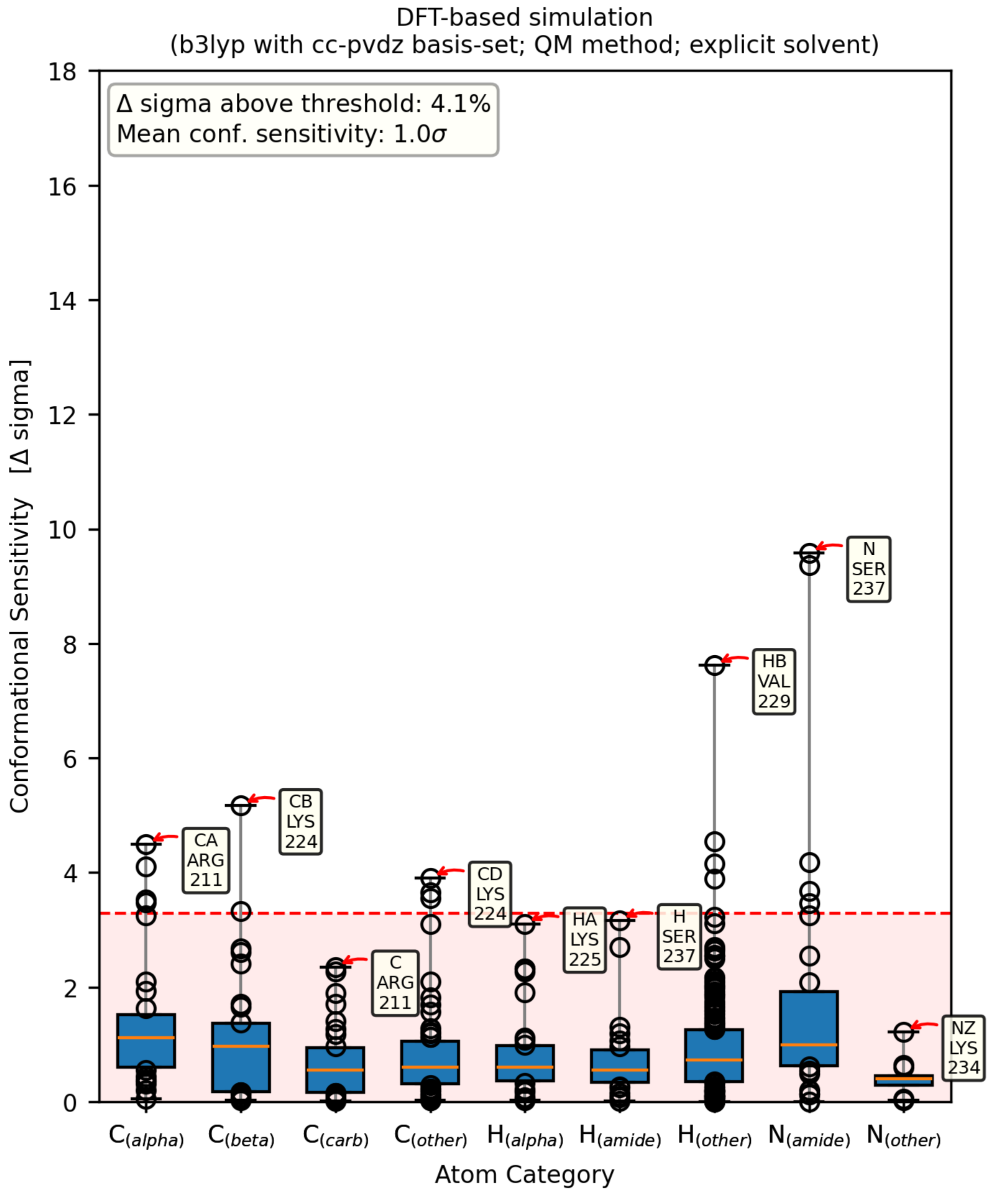

Figure S4.8: Conformational sensitivity calculated with the DFT-based QM method using b3lyp/cc-pvdz theory with explicit solvent.

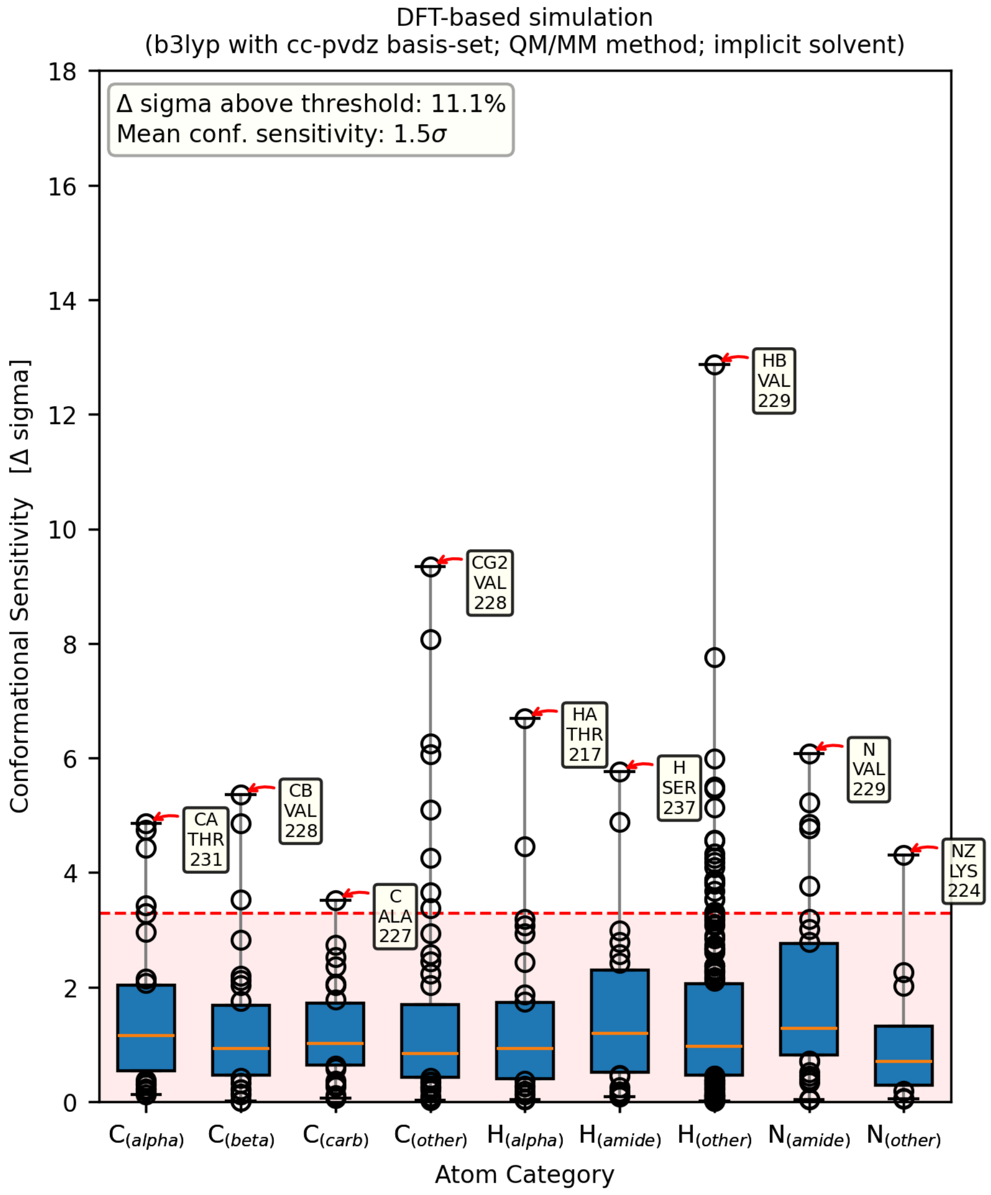

Figure S4.9: Conformational sensitivity calculated with the DFT-based QM/MM method using b3lyp/cc-pvdz theory with implicit solvent.

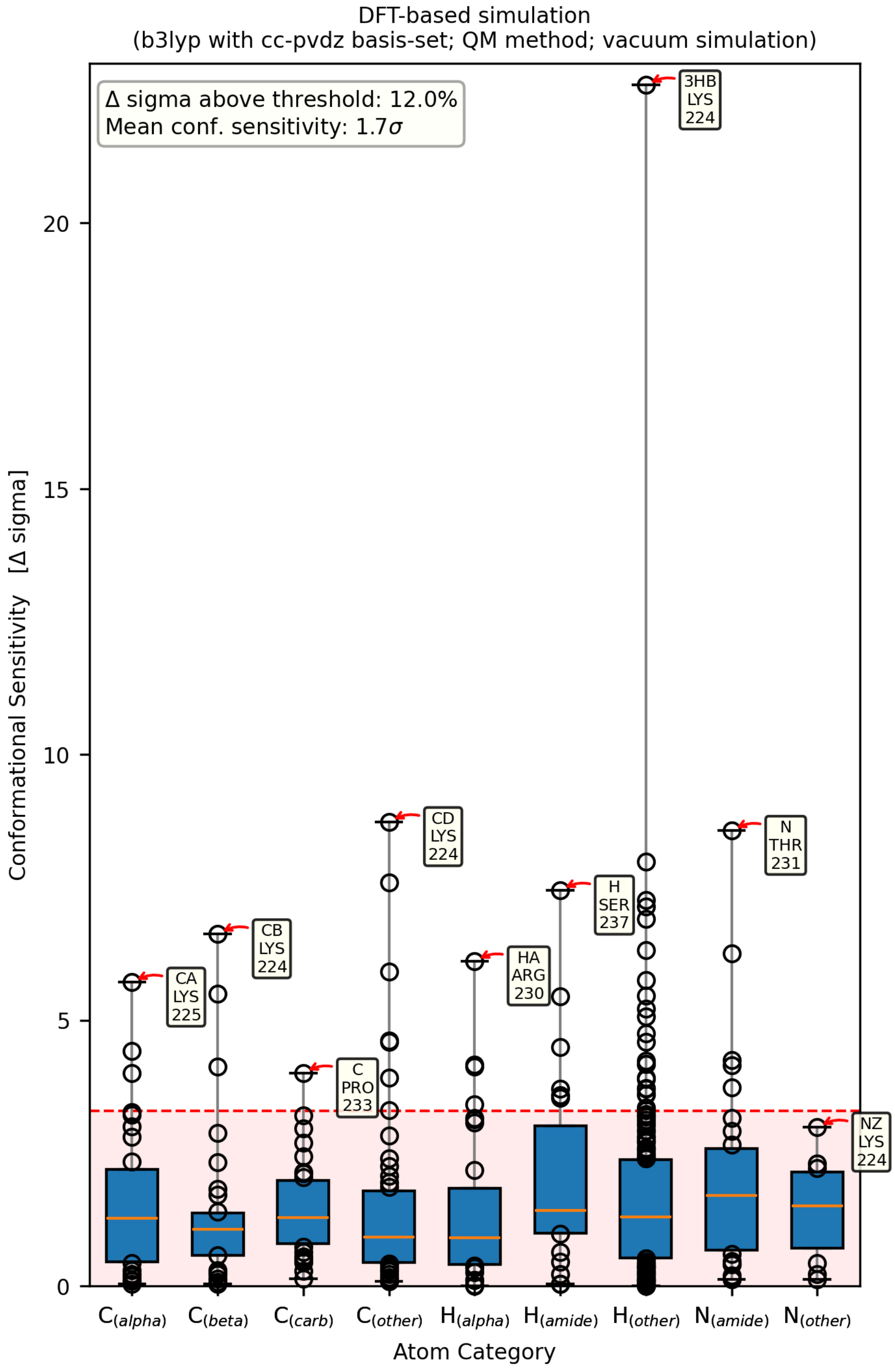

Figure S4.10: Conformational sensitivity calculated with the DFT-based QM method using b3lyp/cc-pvdz theory in vacuum.

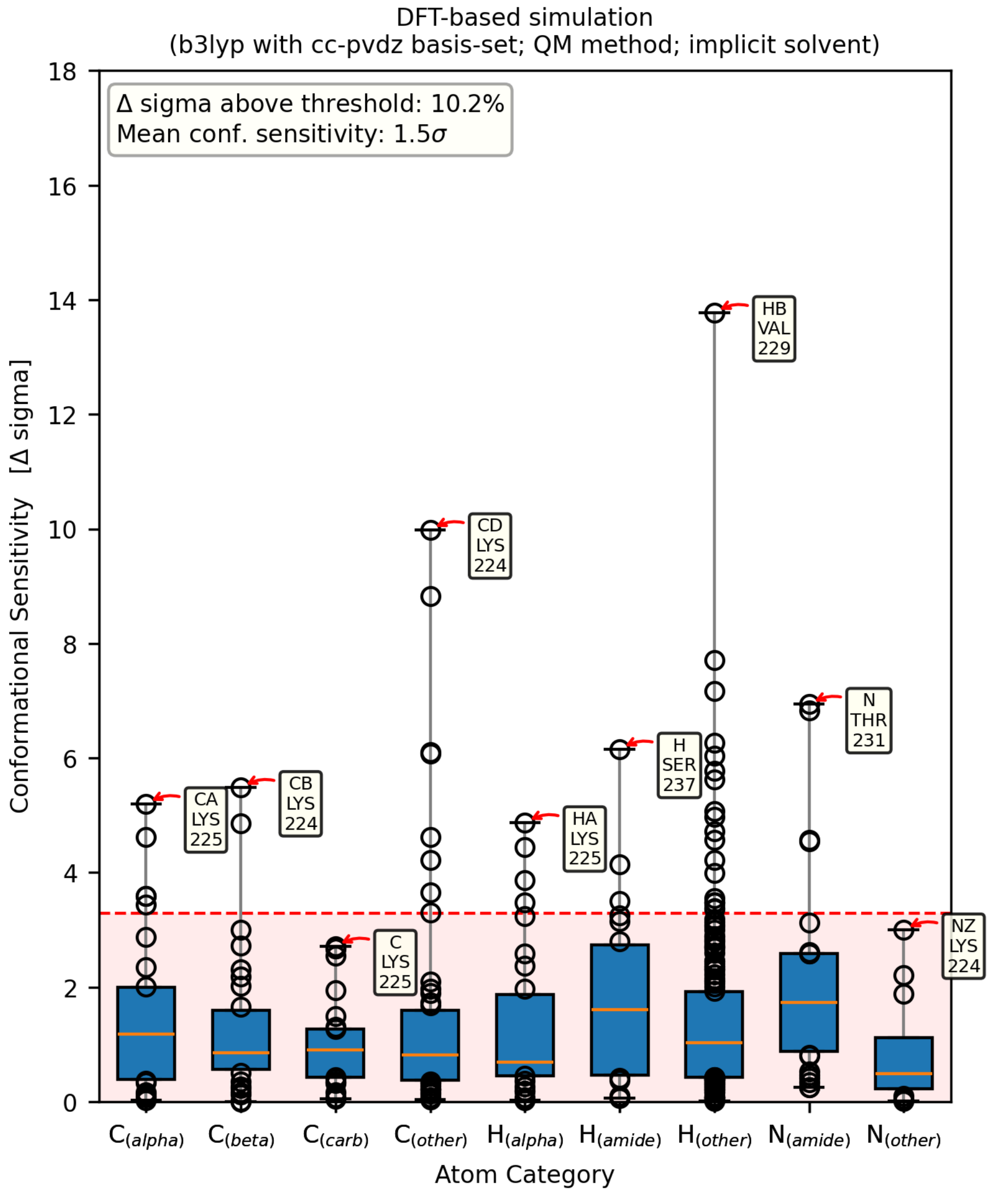

Figure S4.11: Conformational sensitivity calculated with the DFT-based QM method using b3lyp/cc-pvdz theory with implicit solvent.

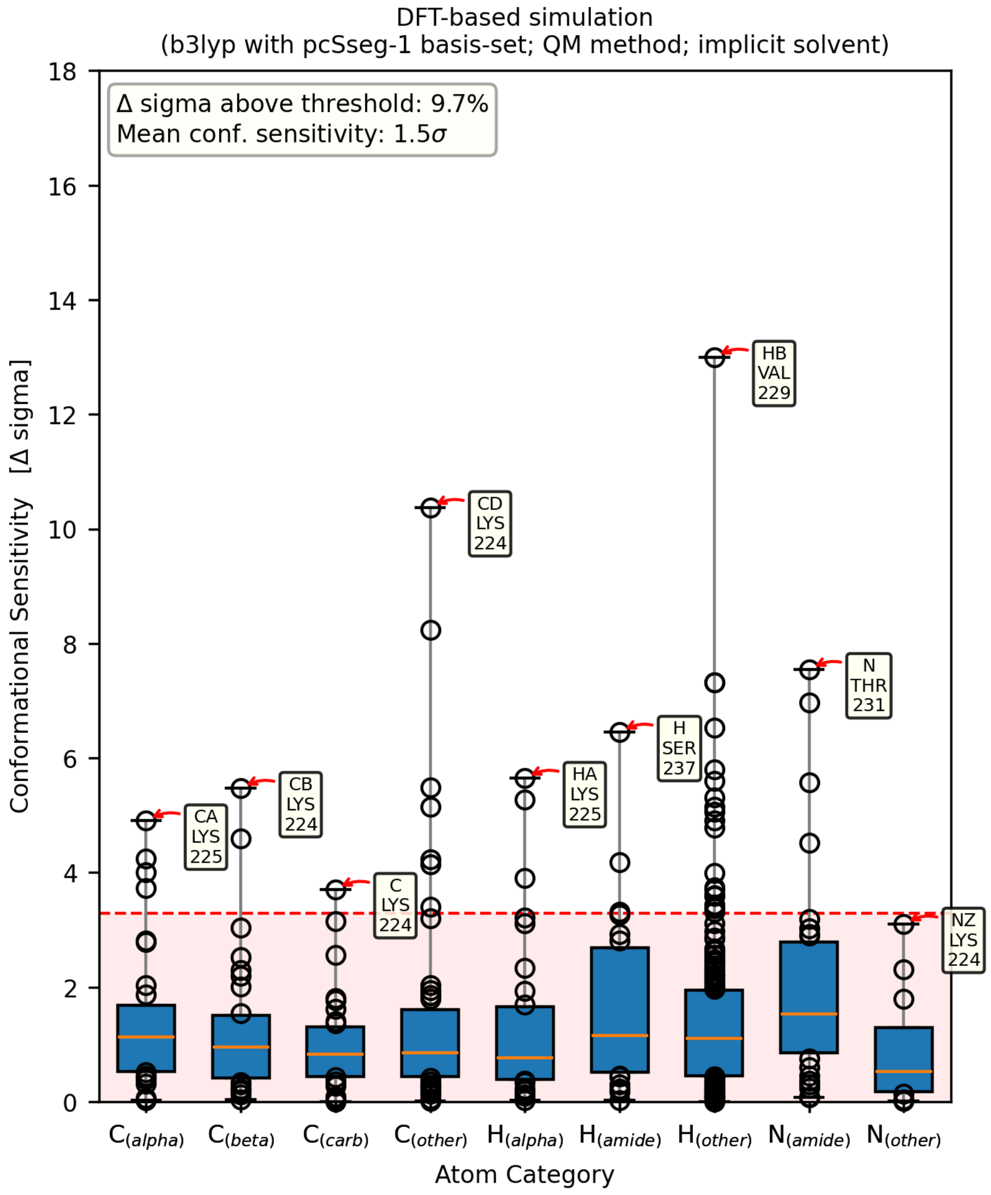

Figure S4.12: Conformational sensitivity calculated with the DFT-based QM method using b3lyp/pcSseg-1 theory with implicit solvent.

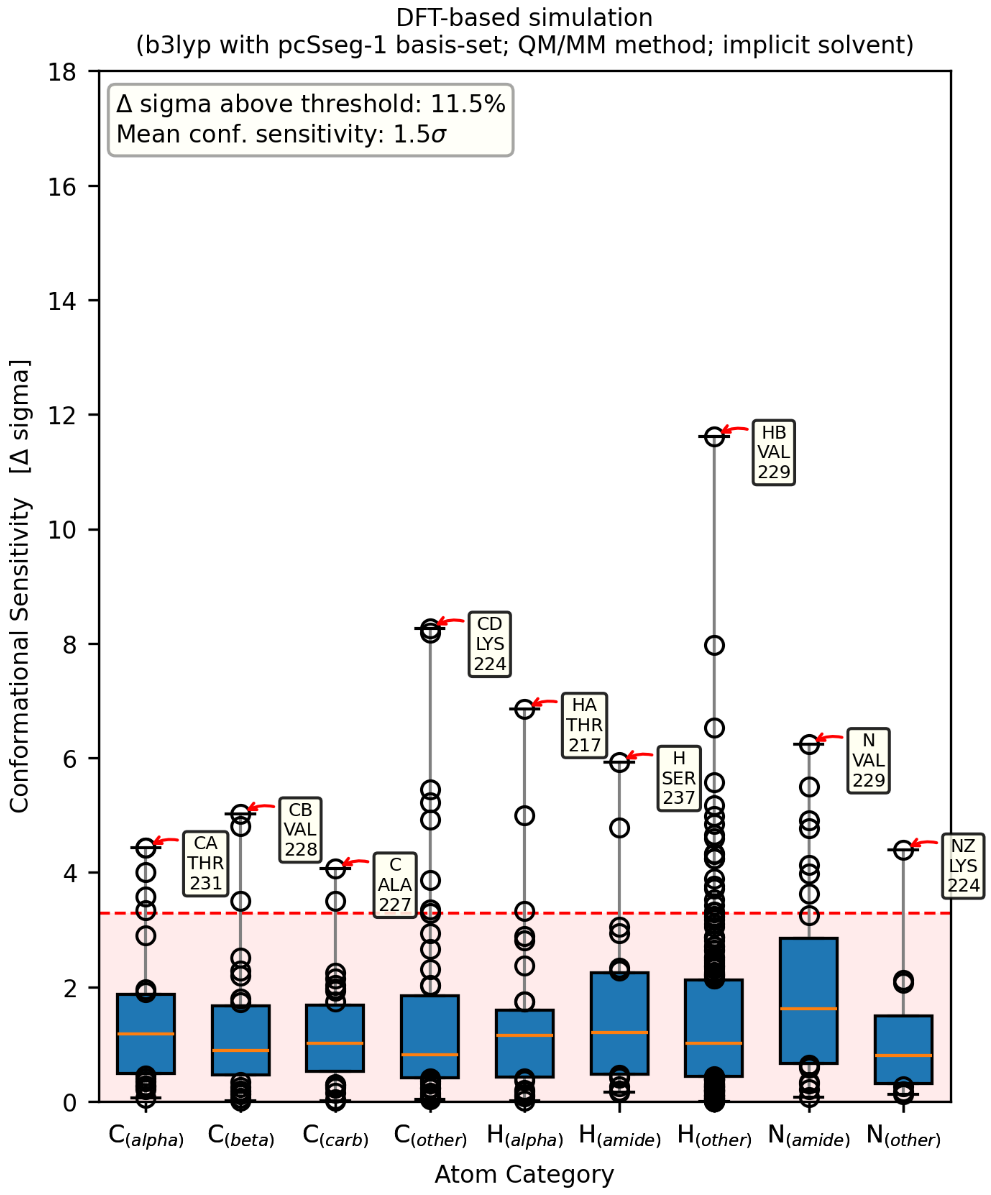

Figure S4.13: Conformational sensitivity calculated with the DFT-based QM/MM method using b3lyp/pcSseg-1 theory with implicit solvent.

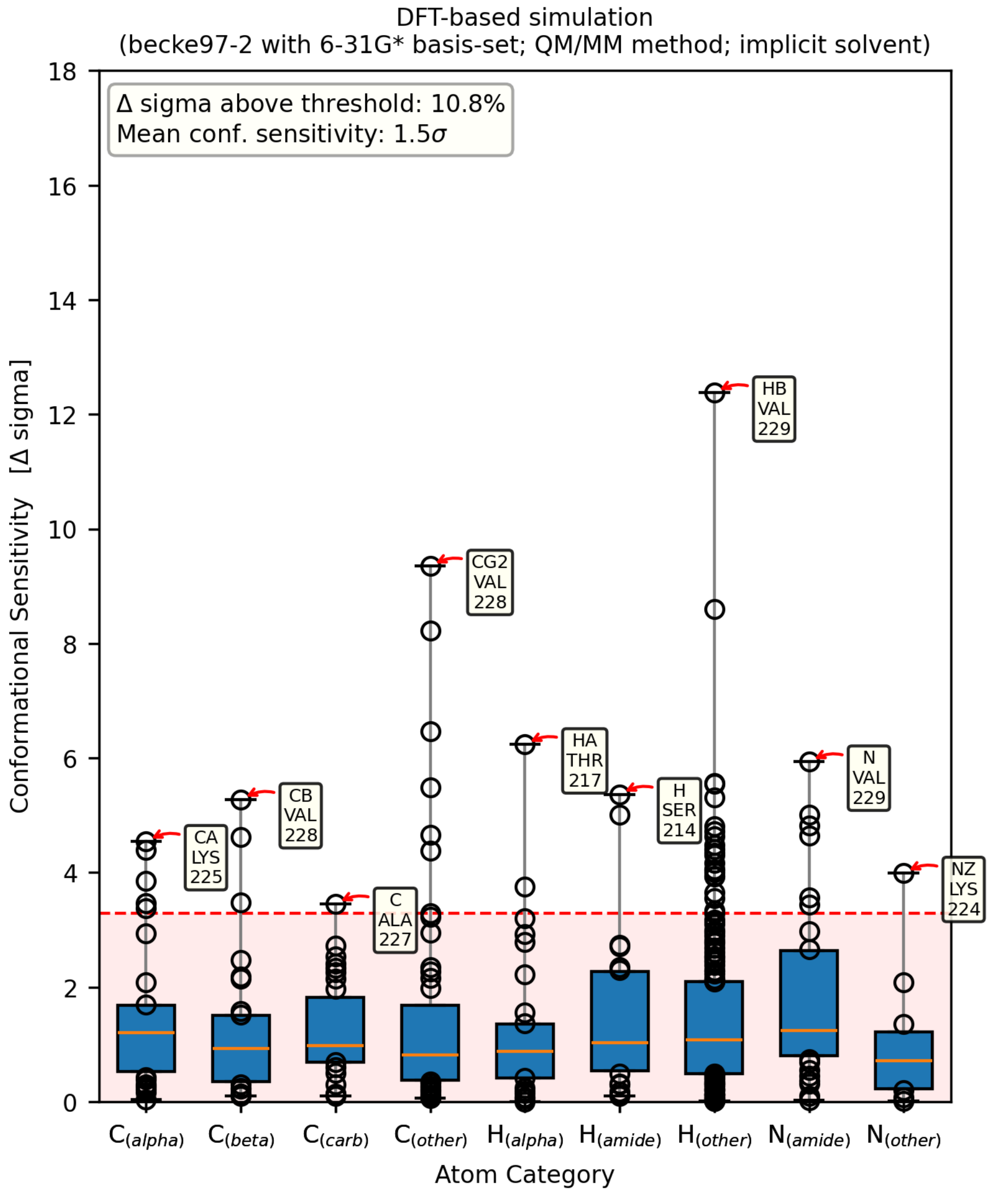

Figure S4.14: Conformational sensitivity calculated with the DFT-based QM/MM method using becke97-2/6-31G* theory with implicit solvent.

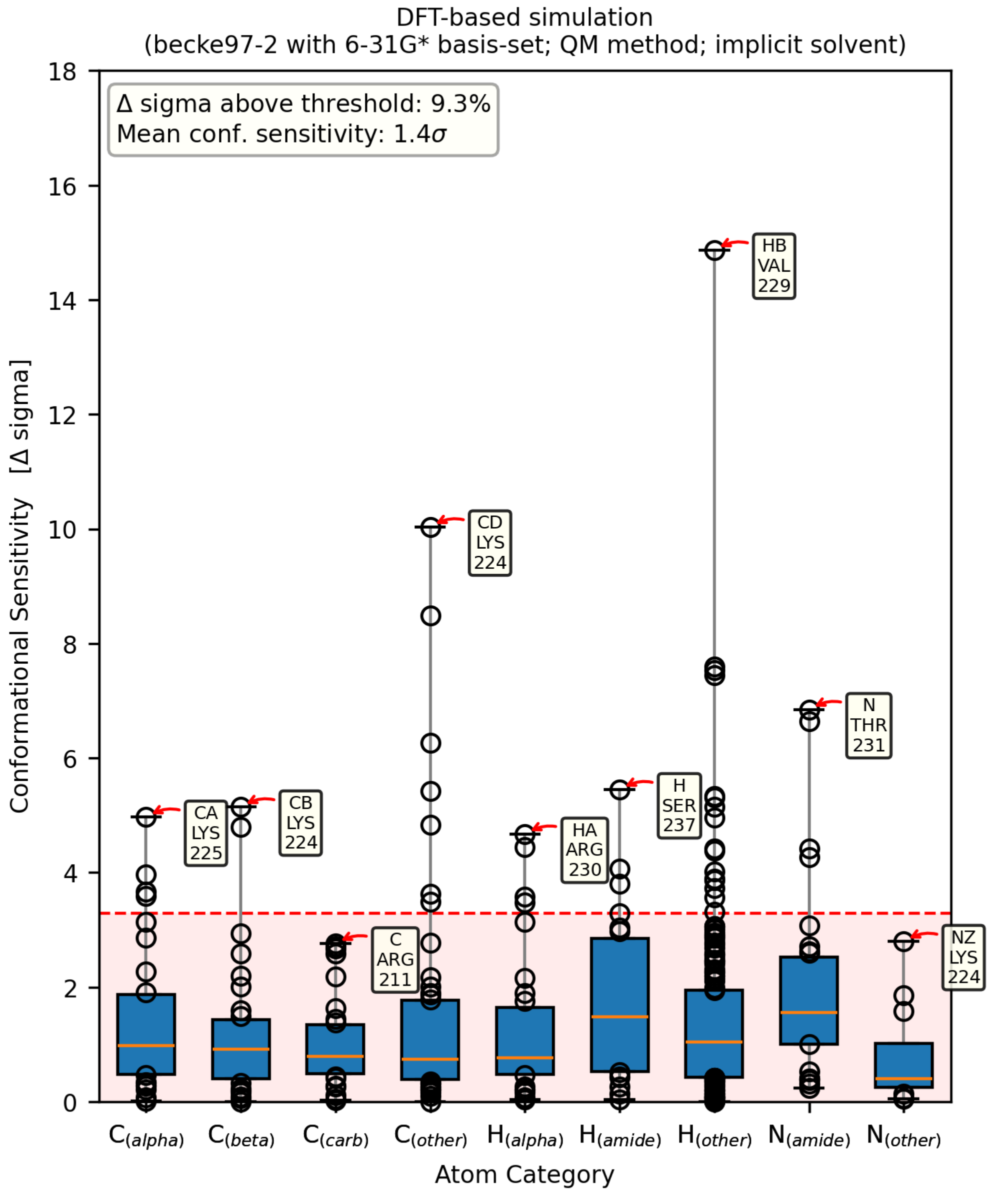

Figure S4.15: Conformational sensitivity calculated with the DFT-based QM method using becke97-2/6-31G* theory with implicit solvent.

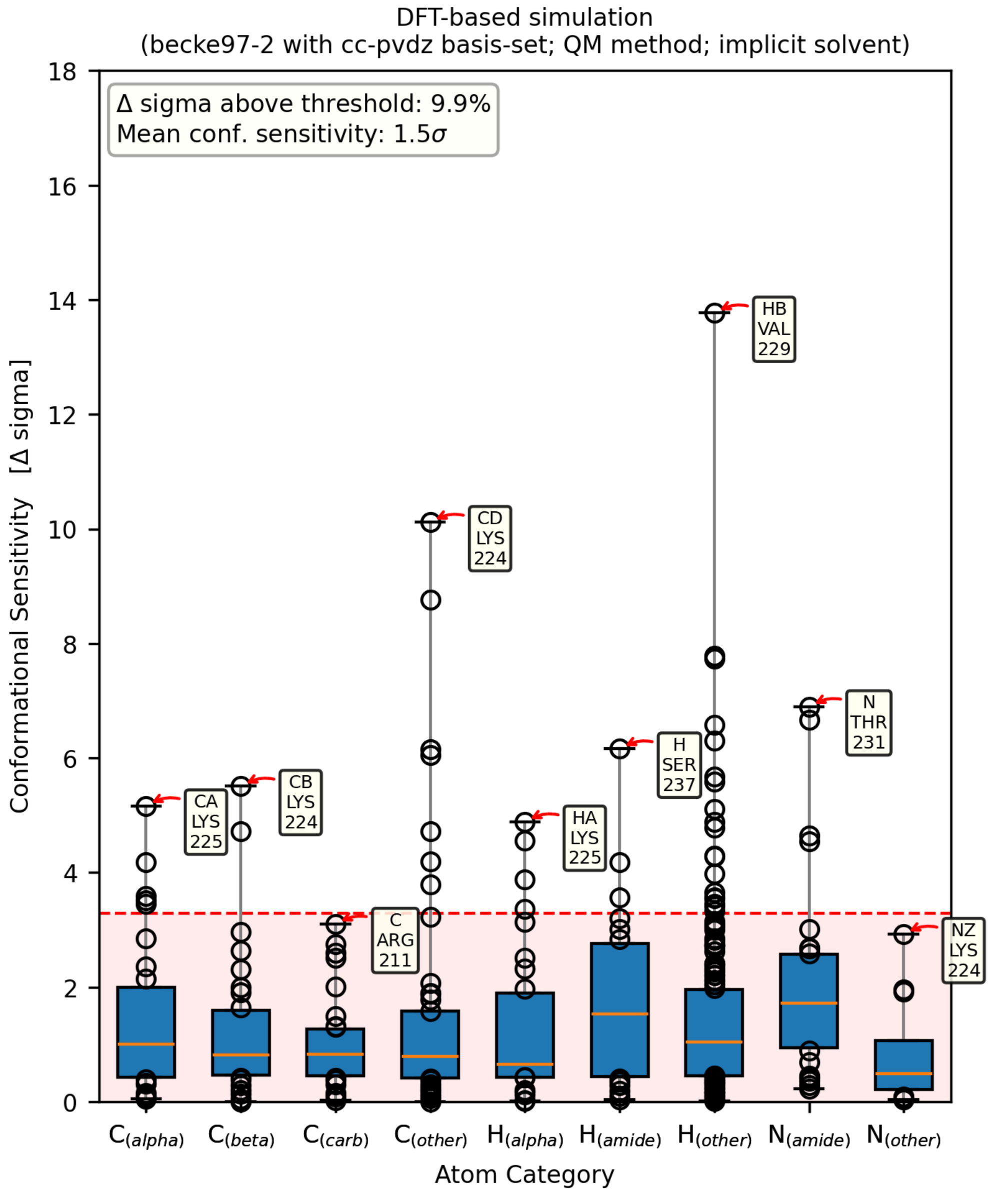

Figure S4.16: Conformational sensitivity calculated with the DFT-based QM method using becke97-2/cc-pvdz theory with implicit solvent.

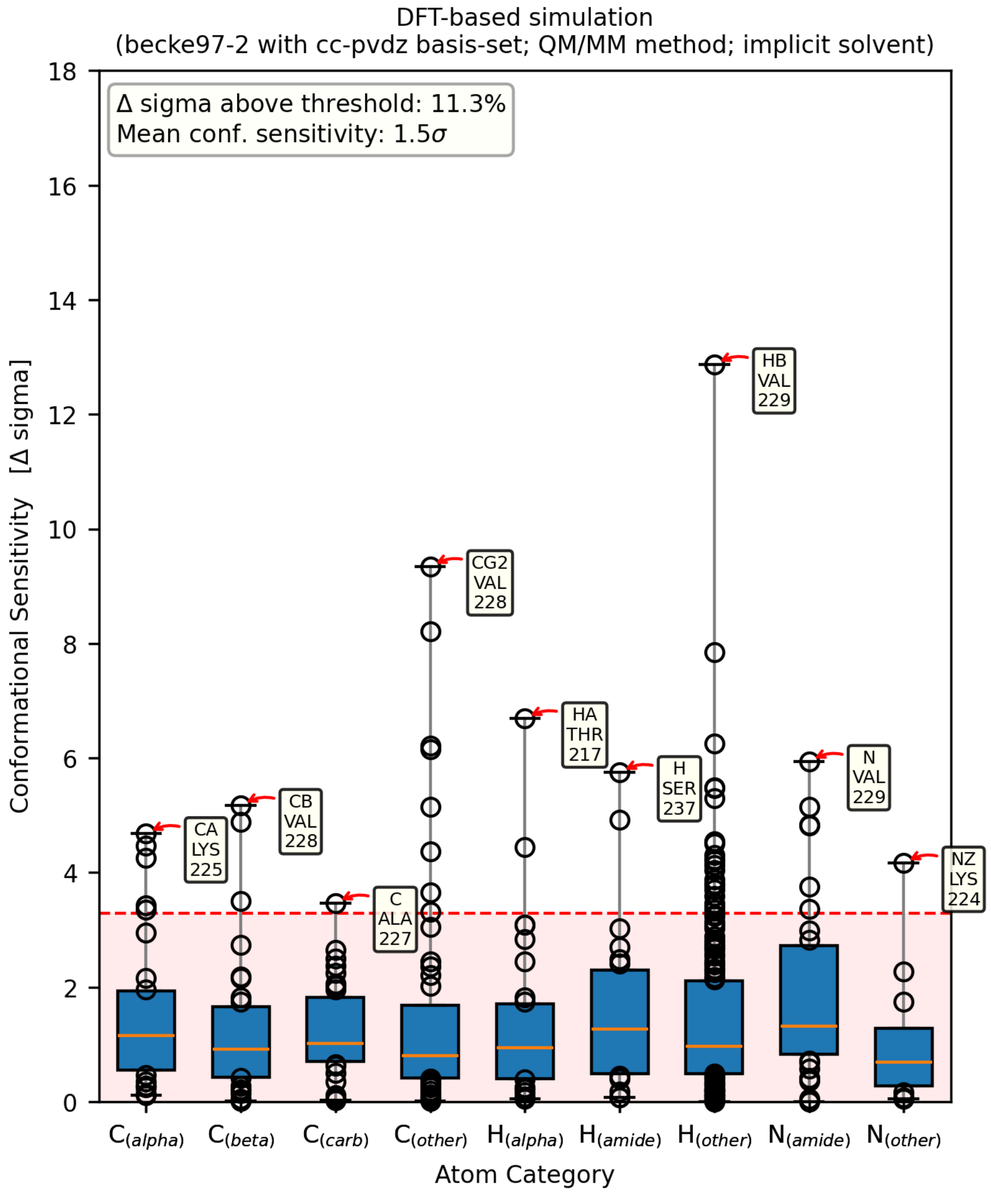

Figure S4.17: Conformational sensitivity calculated with the DFT-based QM/MM method using becke97-2/cc-pvdz theory with implicit solvent.

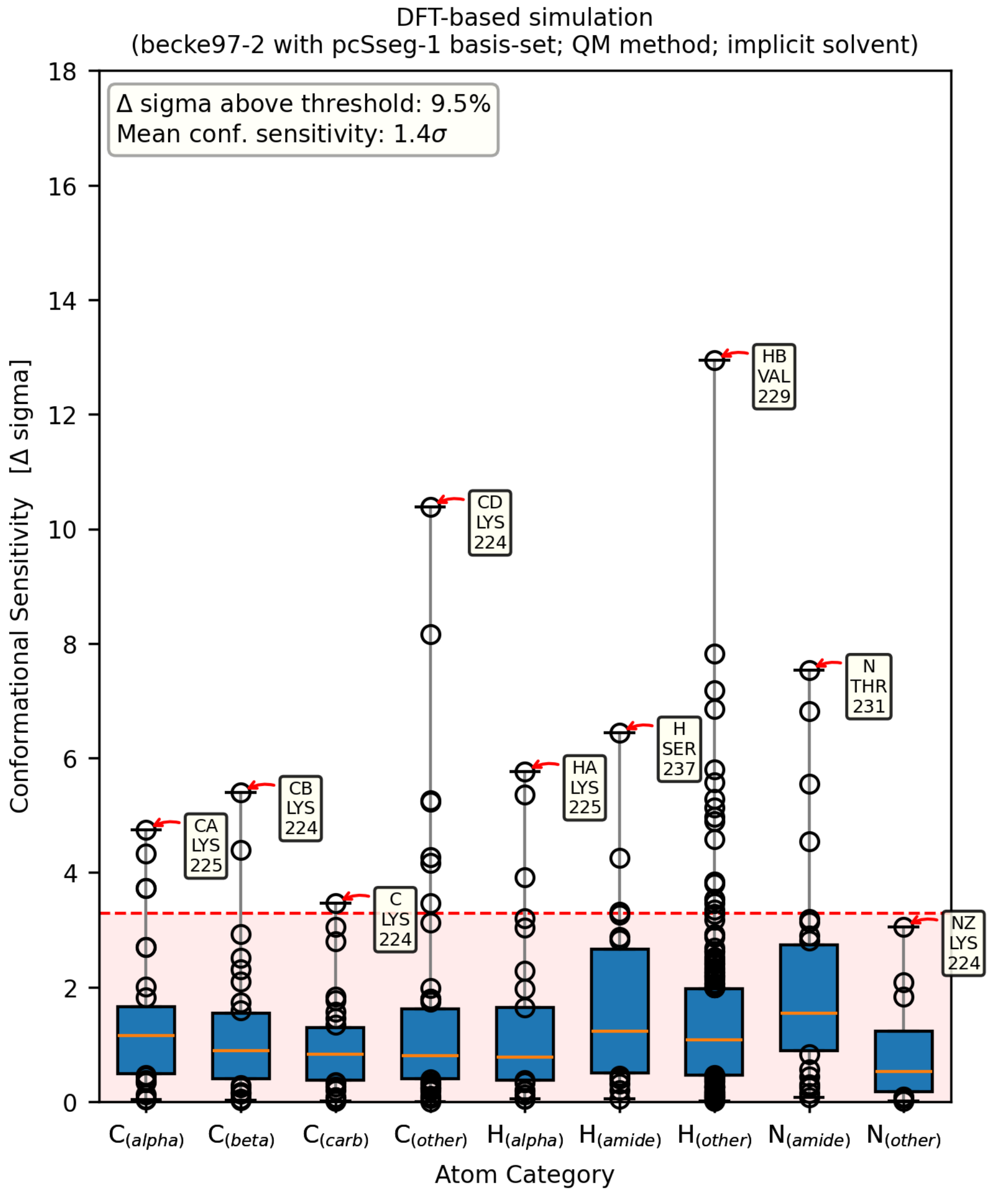

Figure S4.18: Conformational sensitivity calculated with the DFT-based QM method using becke97-2/pcSseg-1 theory with implicit solvent.

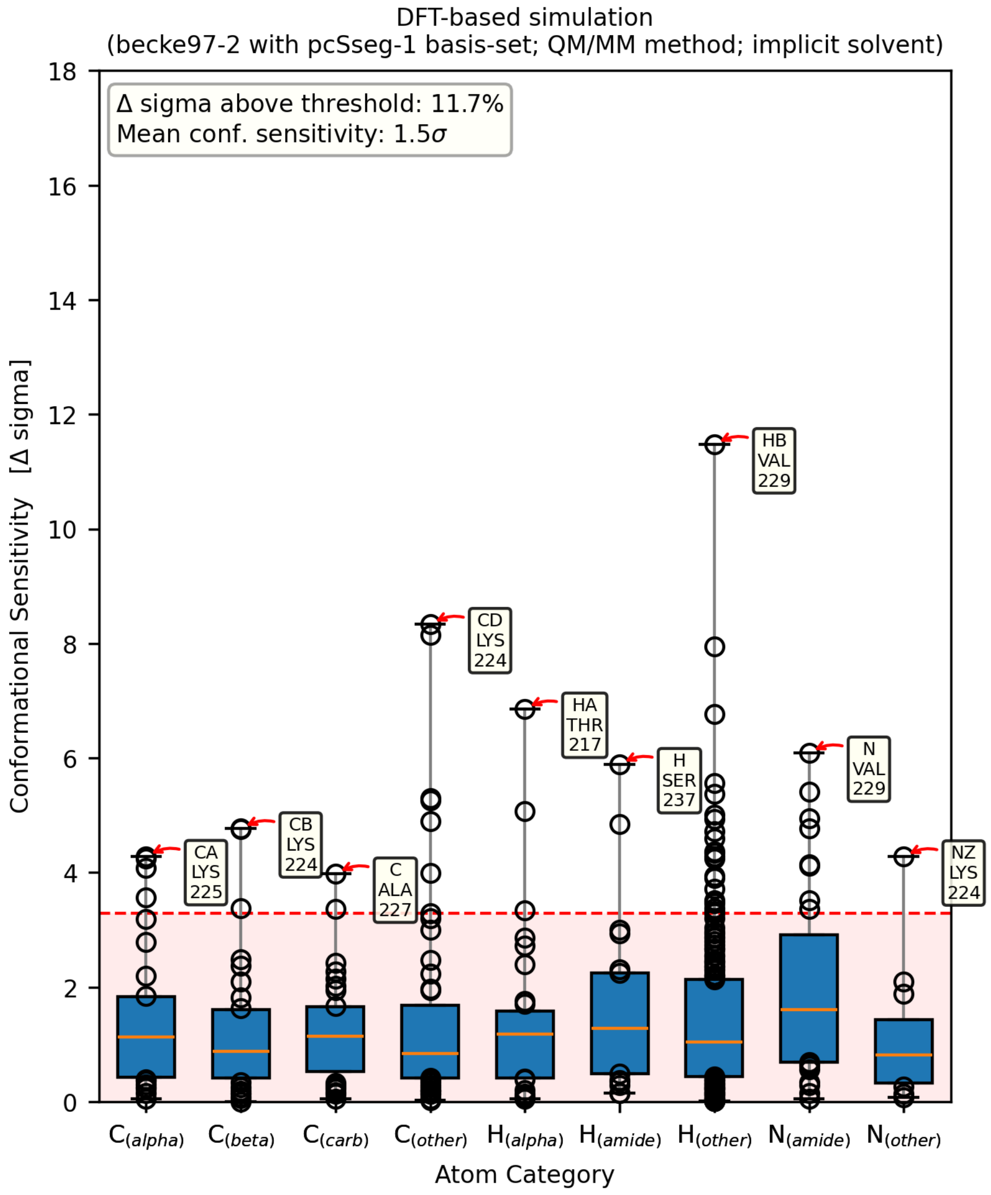

Figure S4.19: Conformational sensitivity calculated with the DFT-based QM/MM method using becke97-2/pcSseg-1 theory with implicit solvent.

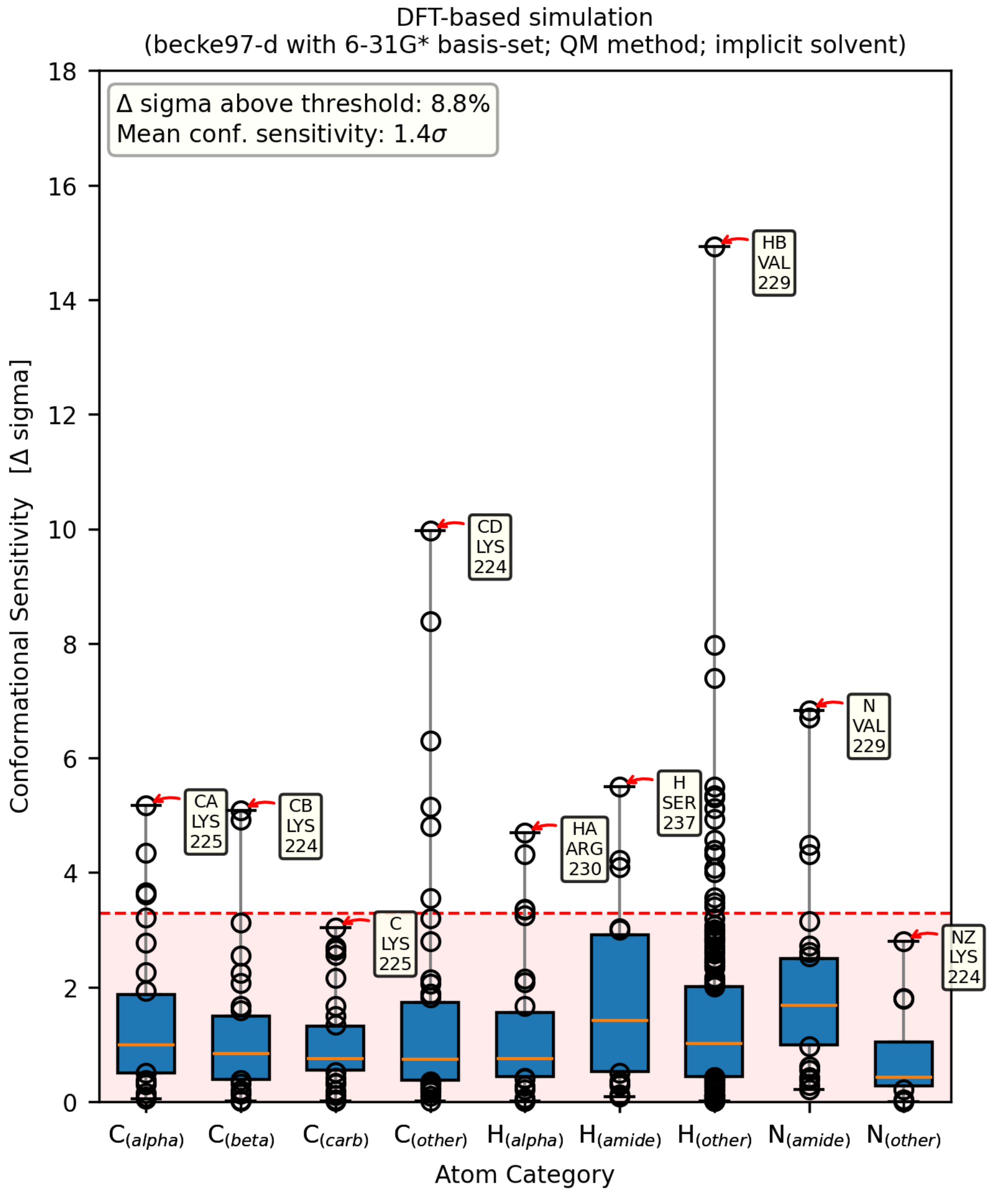

Figure S4.20: Conformational sensitivity calculated with the DFT-based QM method using becke97-d/6-31G* theory with implicit solvent.

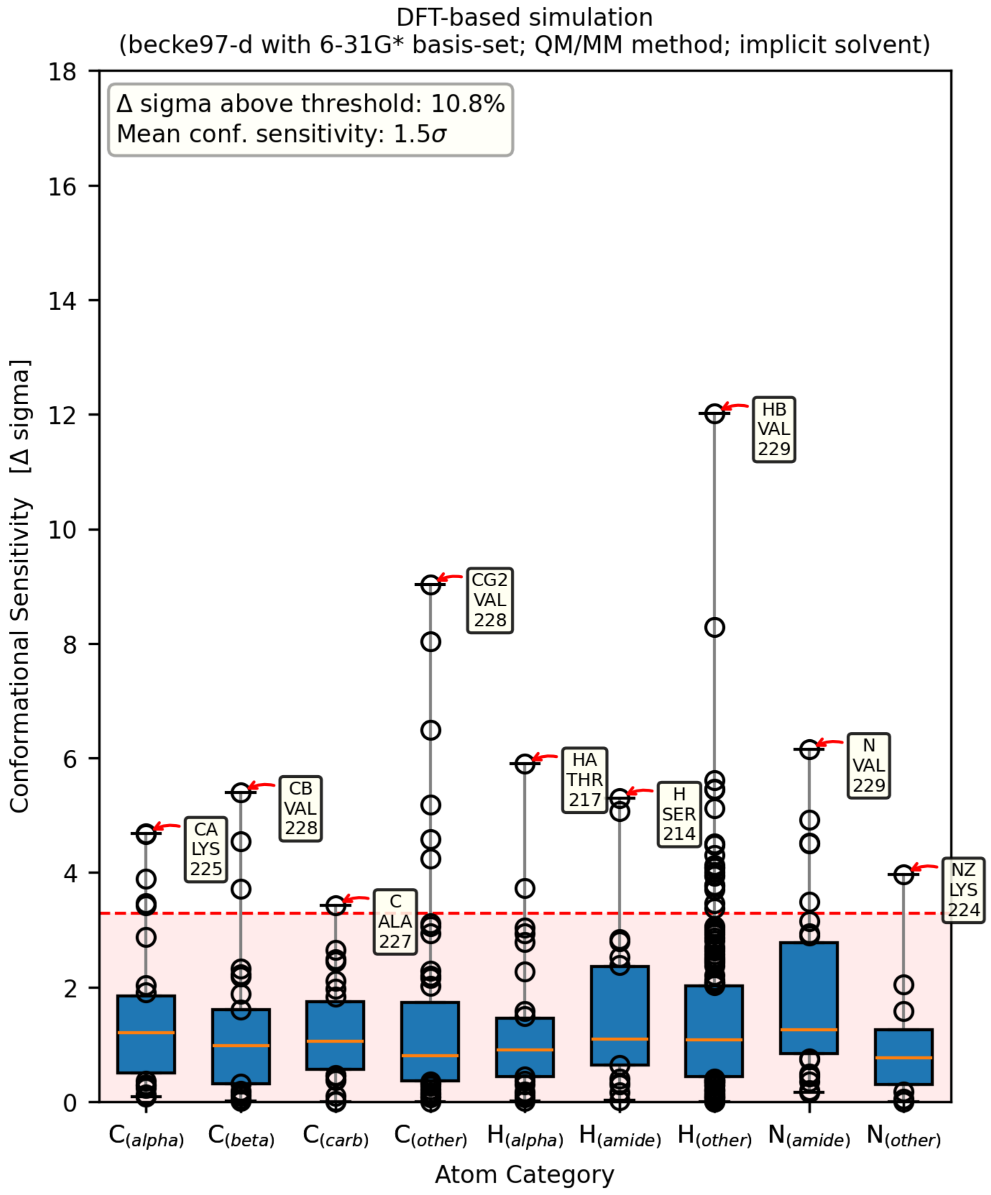

Figure S4.21: Conformational sensitivity calculated with the DFT-based QM/MM method using becke97-d/6-31G* theory with implicit solvent.

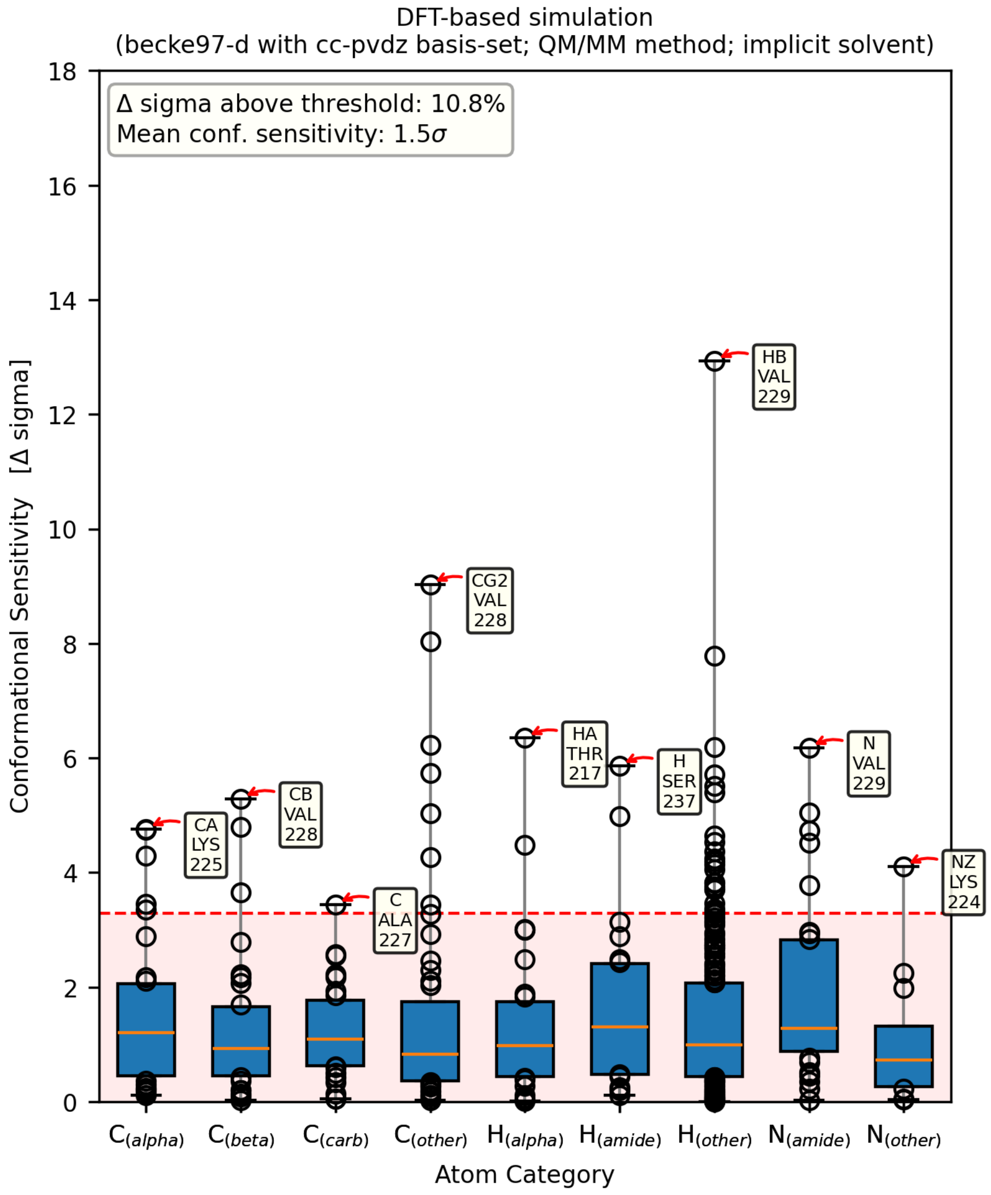

Figure S4.22: Conformational sensitivity calculated with the DFT-based QM/MM method using becke97-d/cc-pvdz theory with implicit solvent.

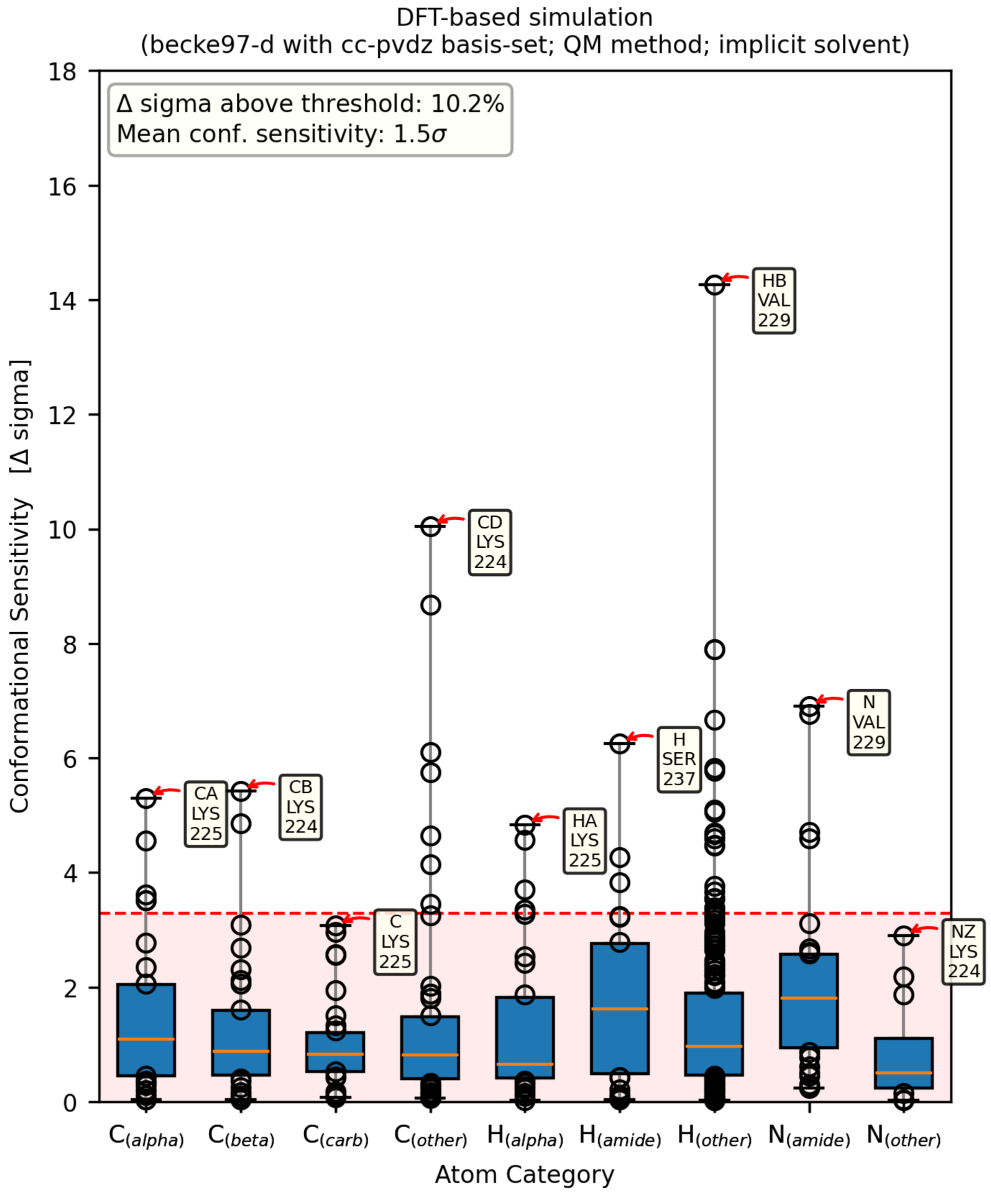

Figure S4.23: Conformational sensitivity calculated with the DFT-based QM method using becke97-d/cc-pvdz theory with implicit solvent.

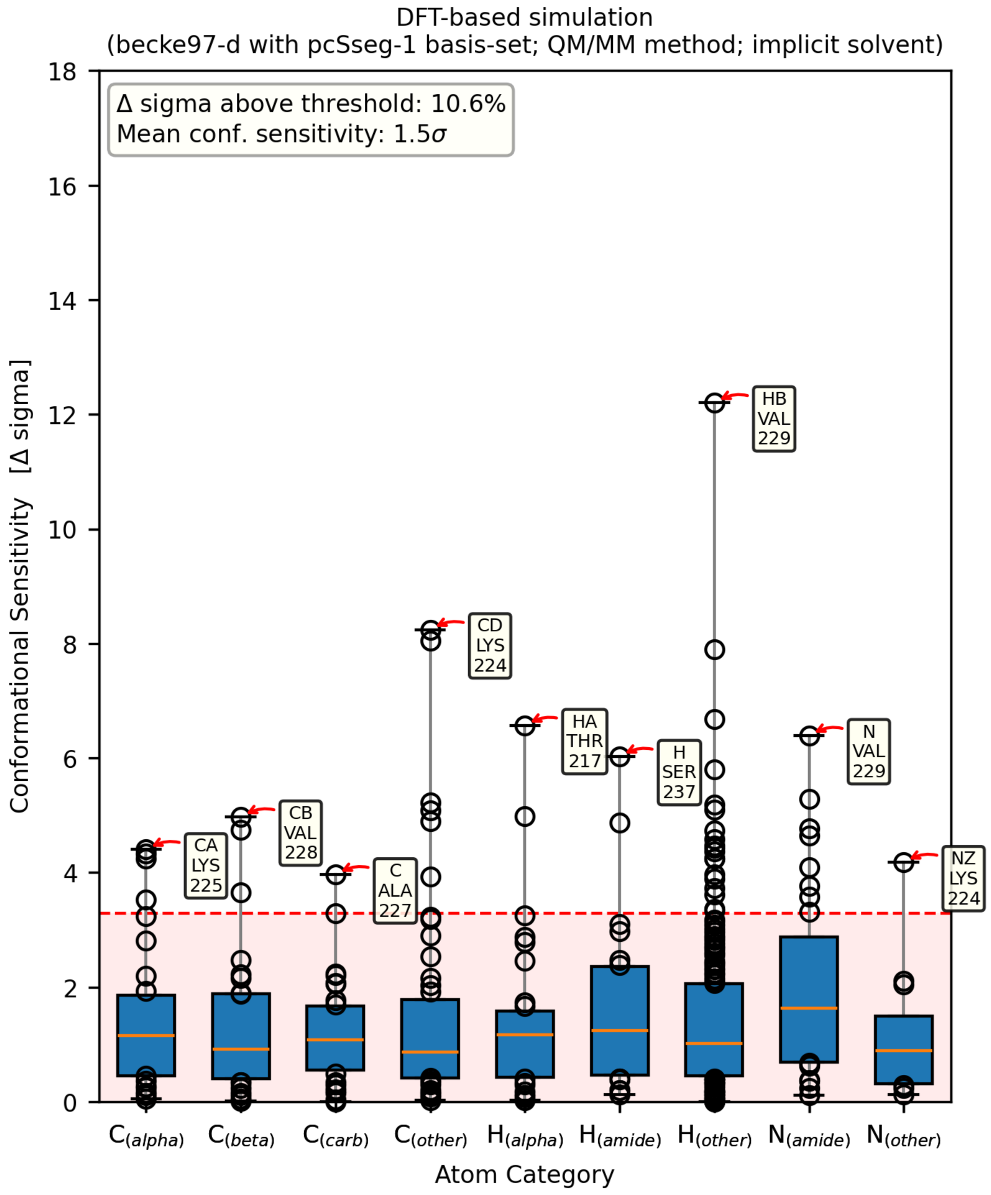

Figure S4.24: Conformational sensitivity calculated with the DFT-based QM/MM method using becke97-d/pcSseg-1 theory with implicit solvent.

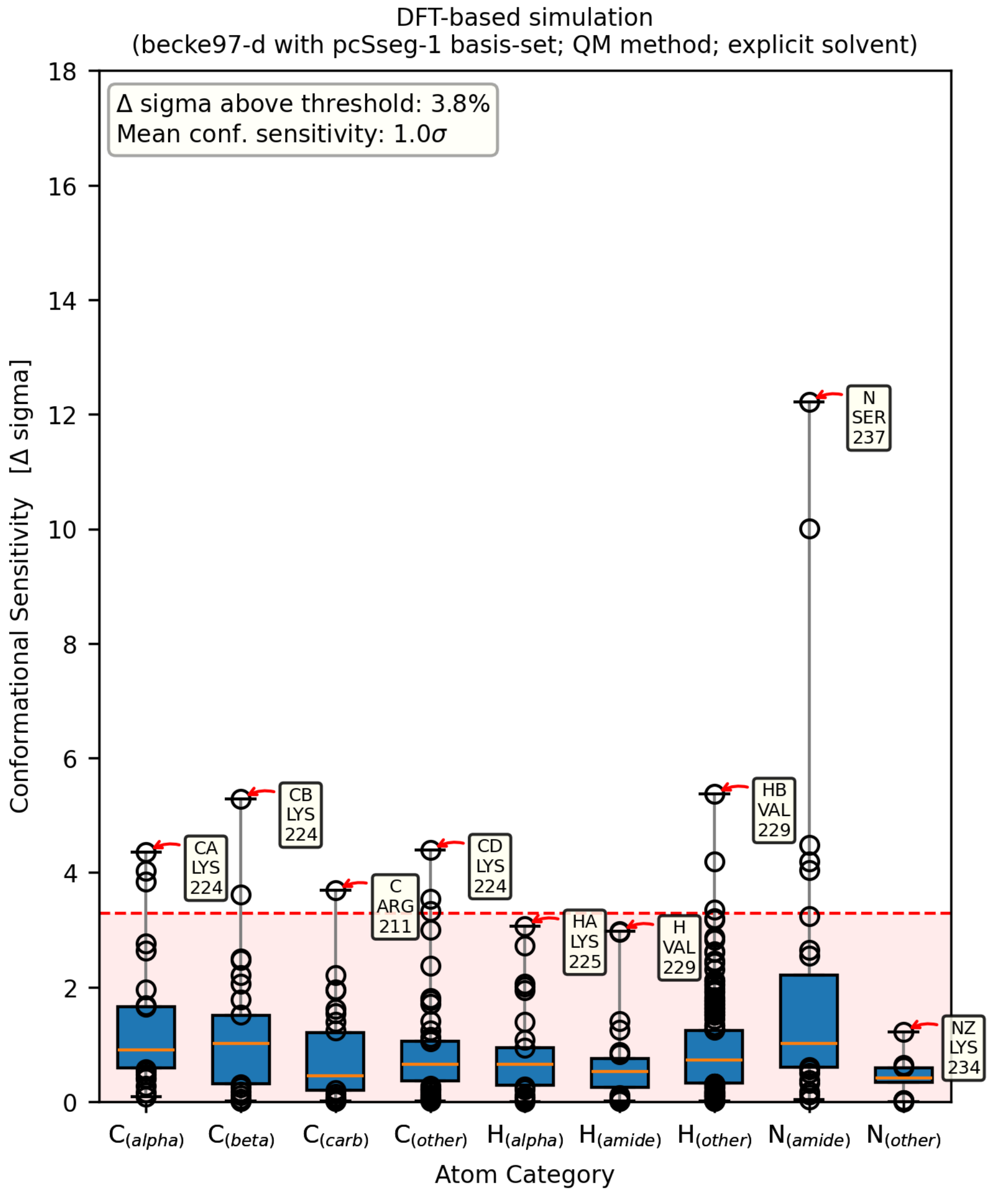

Figure S4.25: Conformational sensitivity calculated with the DFT-based QM method using becke97-d/pcSseg-1 theory with explicit solvent.

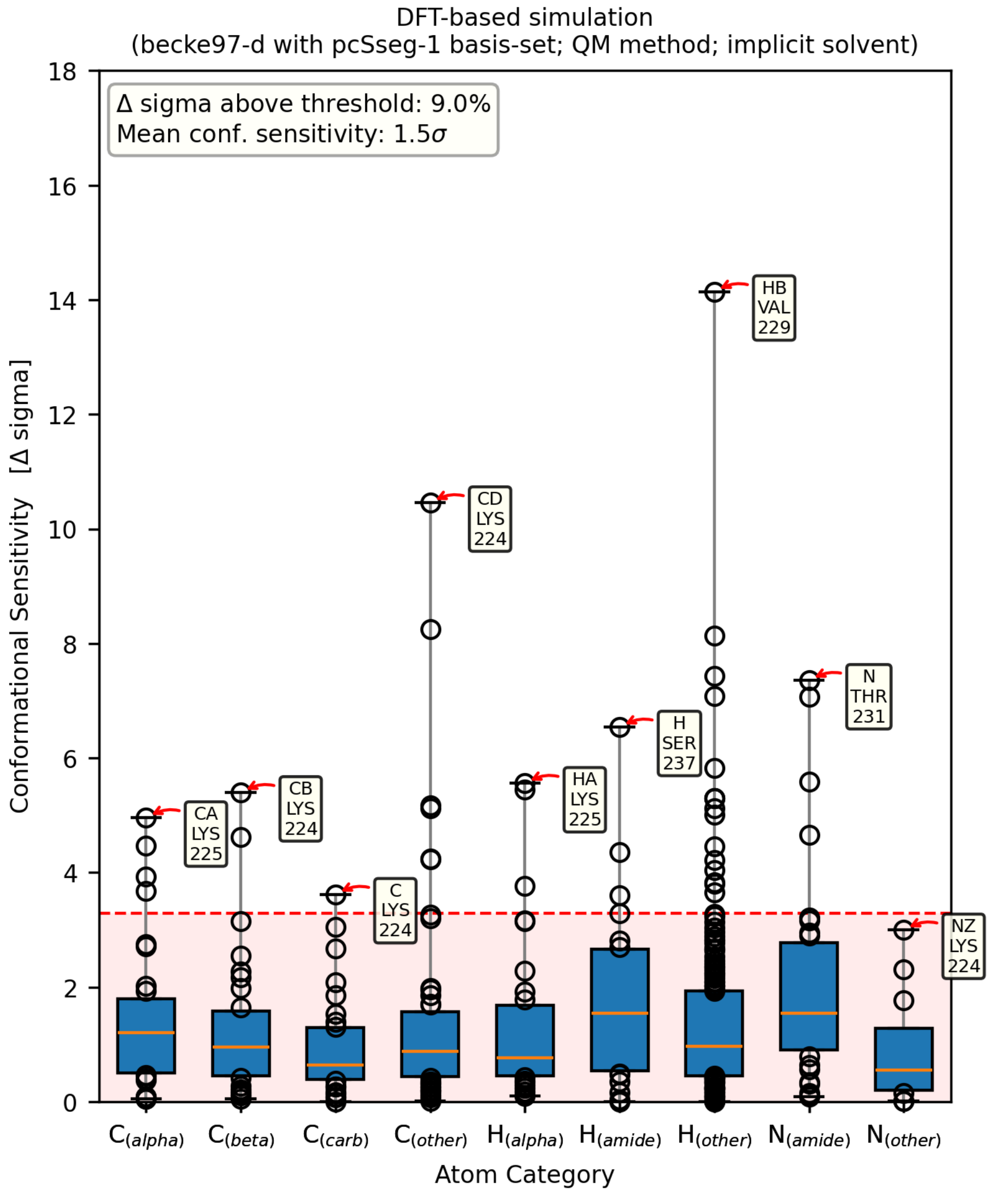

Figure S4.26: Conformational sensitivity calculated with the DFT-based QM method using becke97-d/pcSseg-1 theory with implicit solvent.

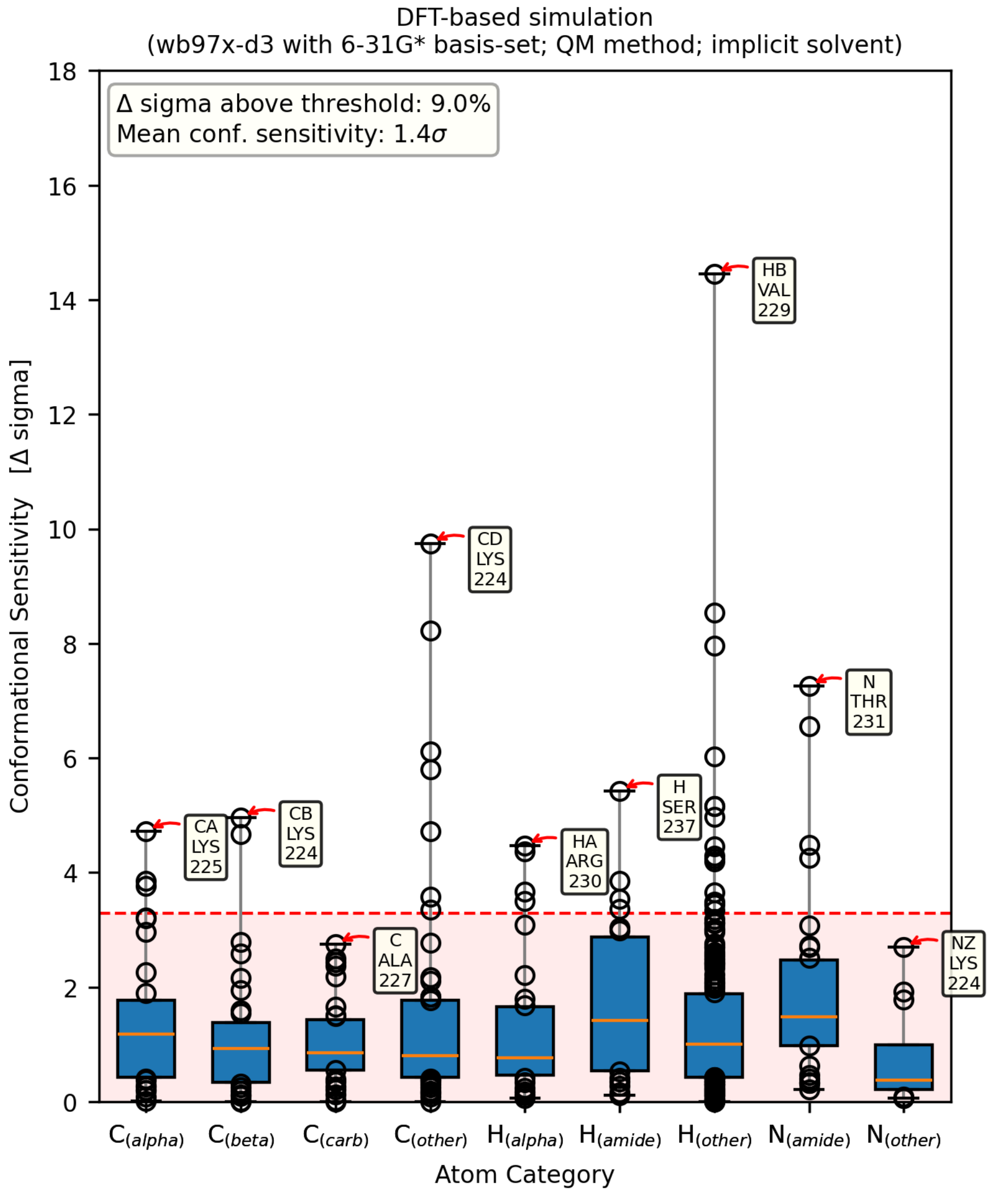

Figure S4.27: Conformational sensitivity calculated with the DFT-based QM method using wb97x-d3/6-31G* theory with implicit solvent.

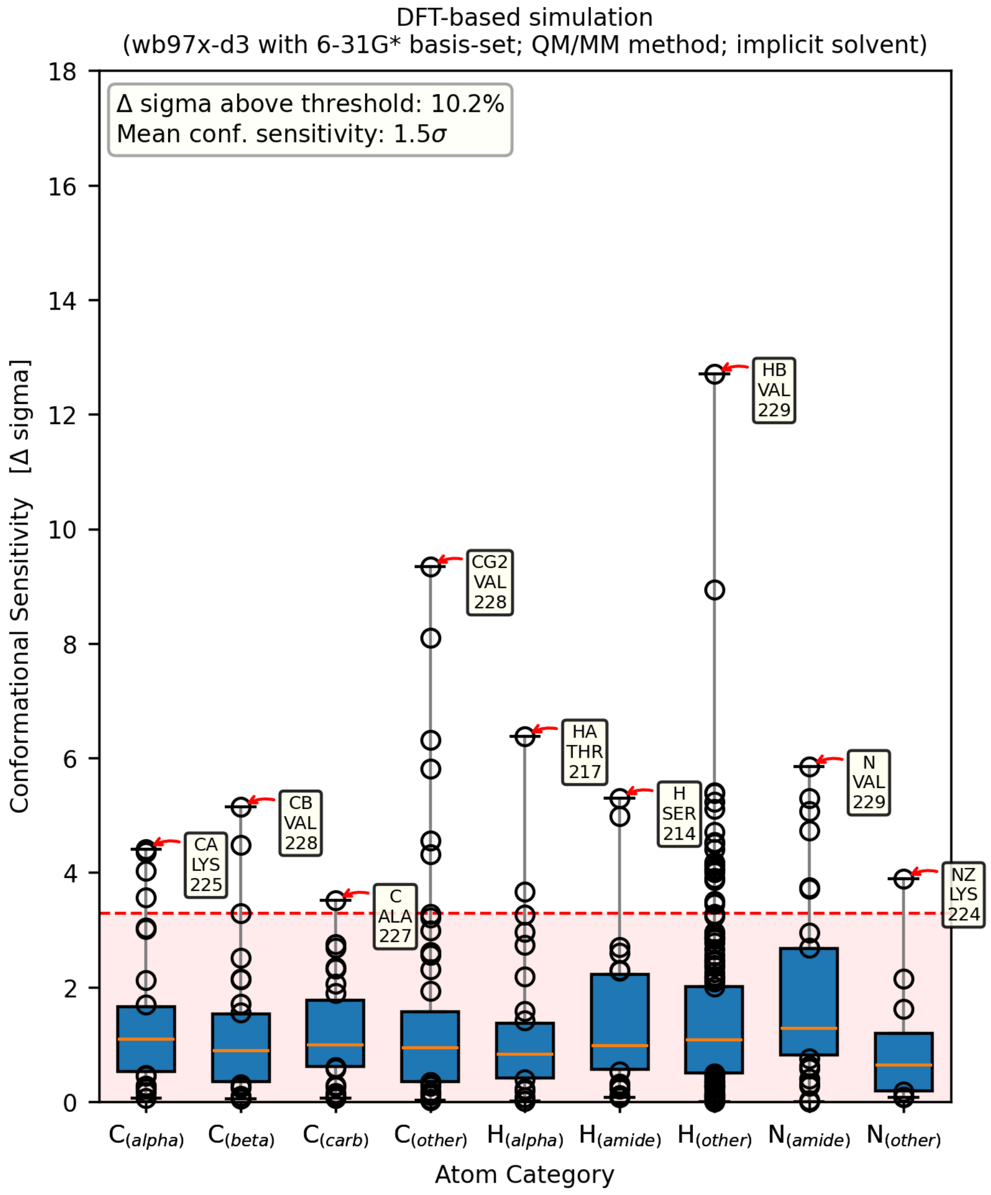

Figure S4.28: Conformational sensitivity calculated with the DFT-based QM/MM method using wb97x-d3/6-31G* theory with implicit solvent.

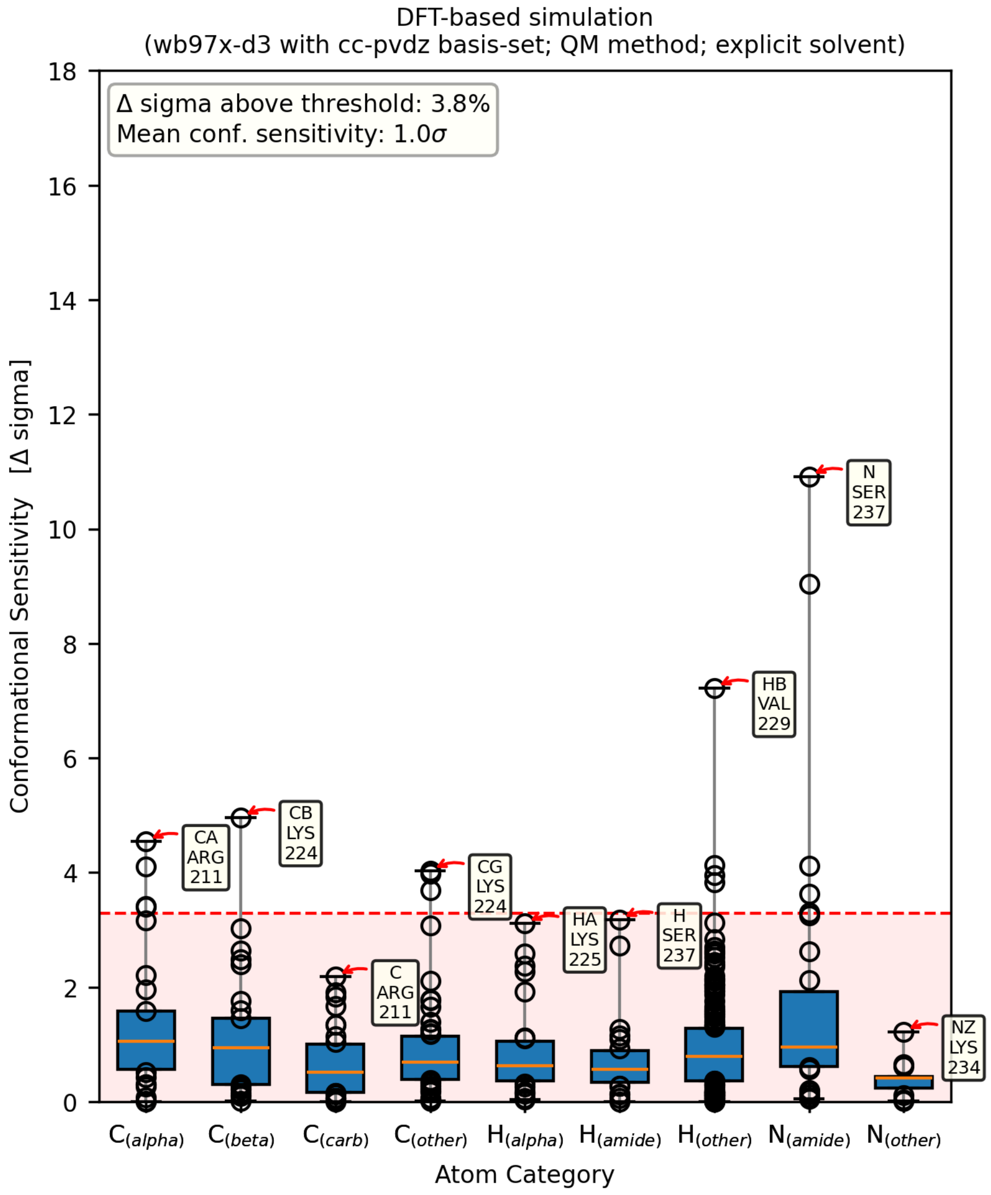

Figure S4.29: Conformational sensitivity calculated with the DFT-based QM method using wb97x-d3/cc-pvdz theory with explicit solvent.

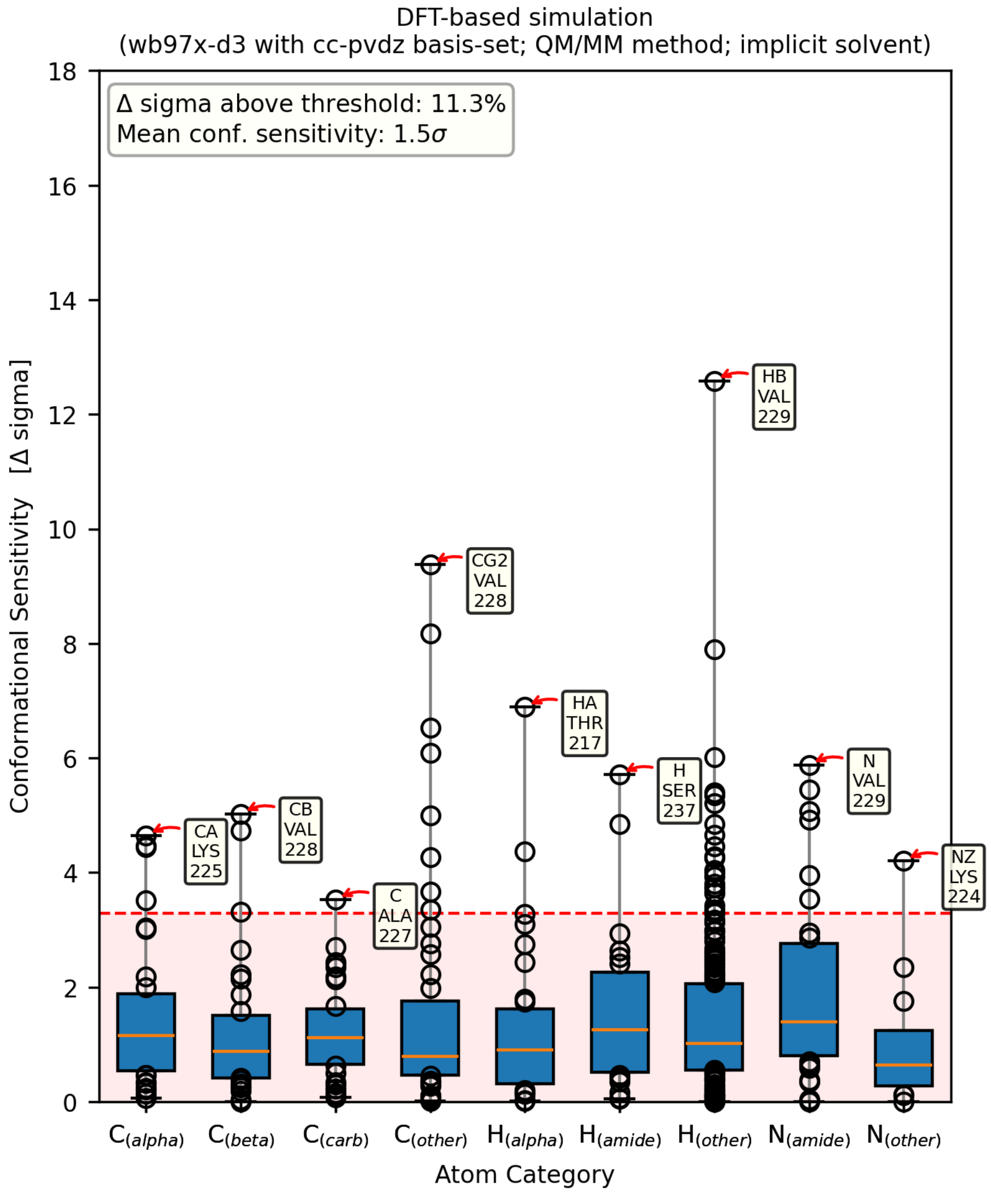

Figure S4.30: Conformational sensitivity calculated with the DFT-based QM/MM method using wb97x-d3/cc-pvdz theory with implicit solvent.

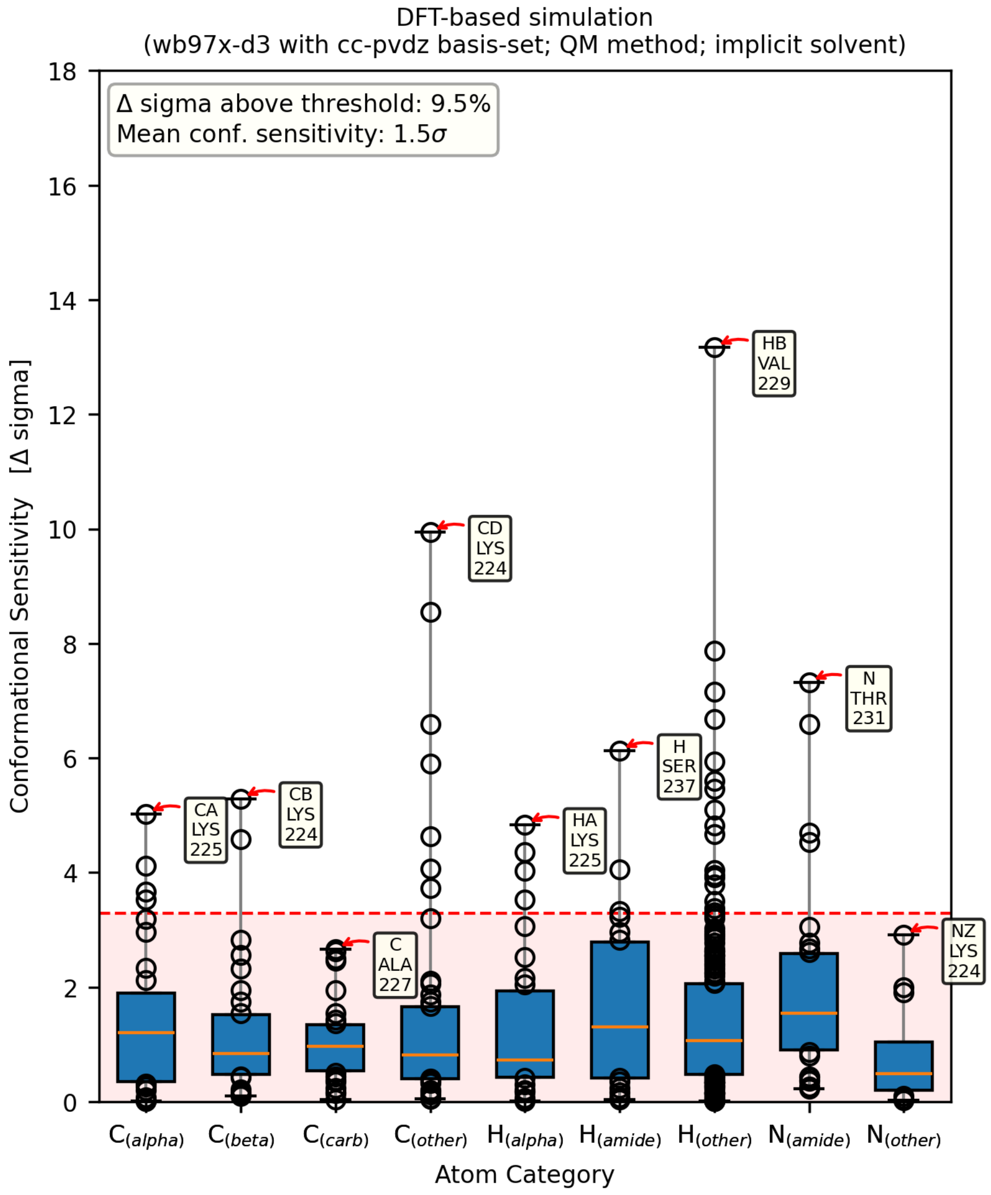

Figure S4.31: Conformational sensitivity calculated with the DFT-based QM method using wb97x-d3/cc-pvdz theory with implicit solvent.

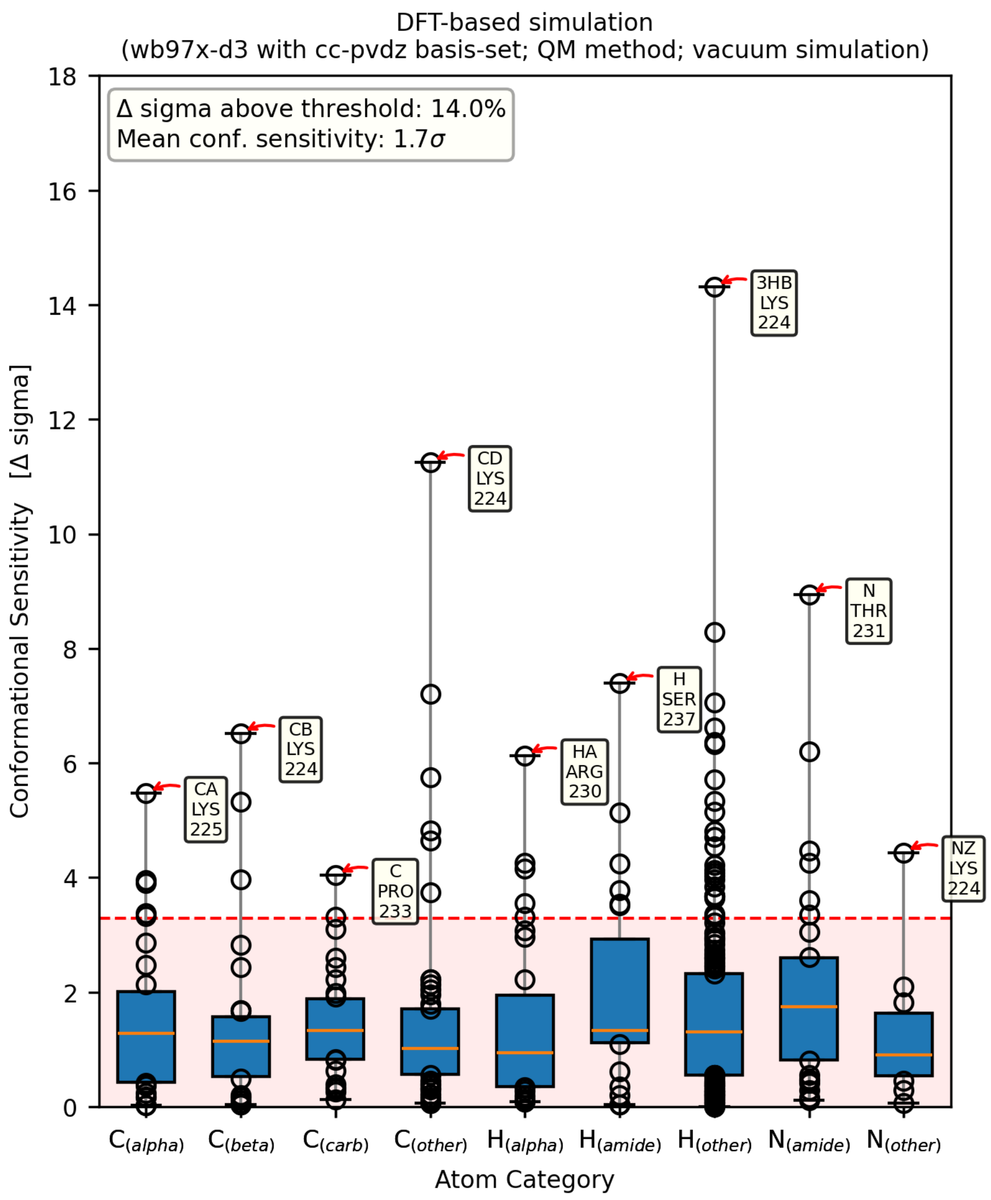

Figure S4.32: Conformational sensitivity calculated with the DFT-based QM method using wb97x-d3/cc-pvdz theory in vacuum.

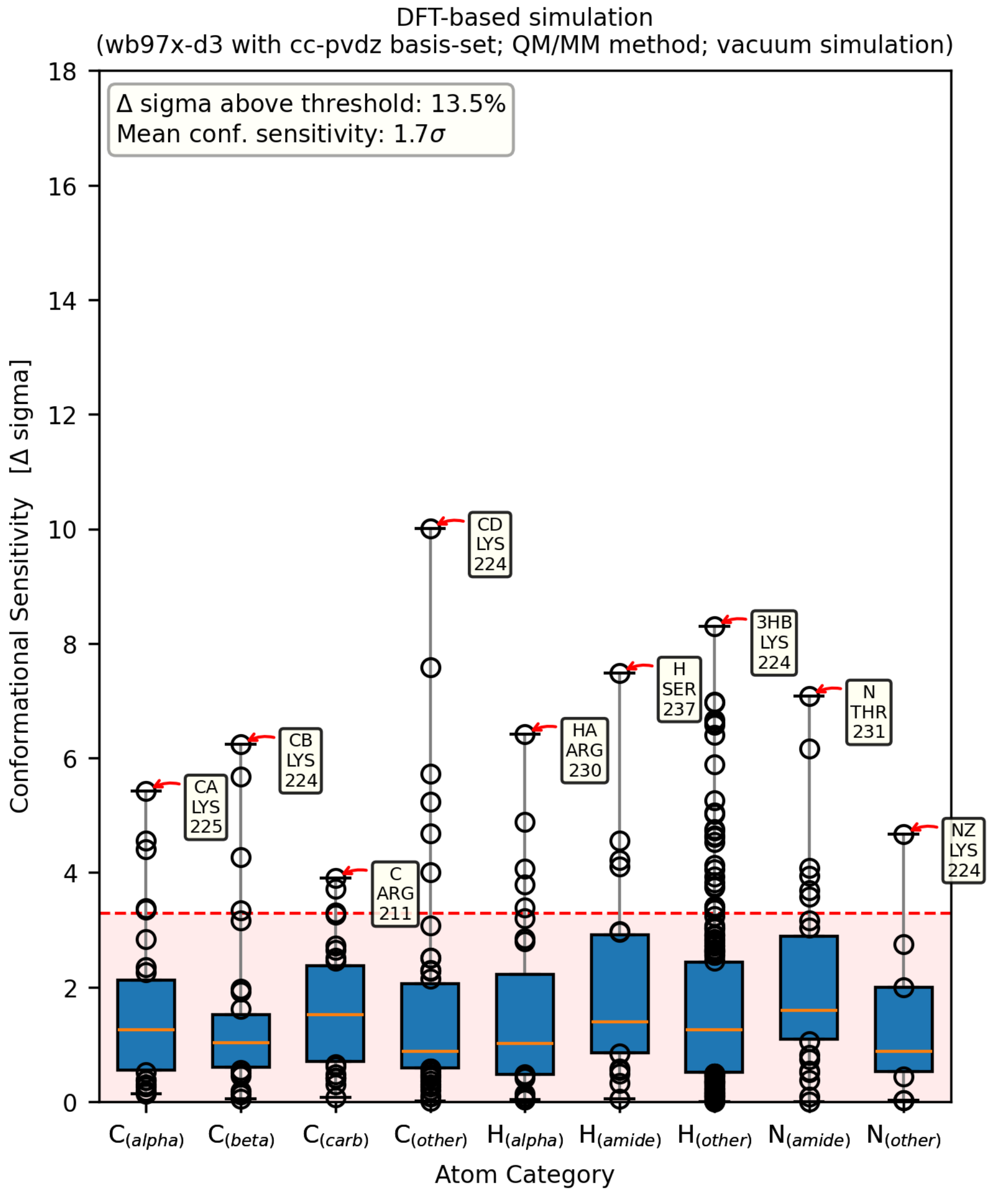

Figure S4.33: Conformational sensitivity calculated with the DFT-based QM/MM method using wb97x-d3/cc-pvdz theory in vacuum.

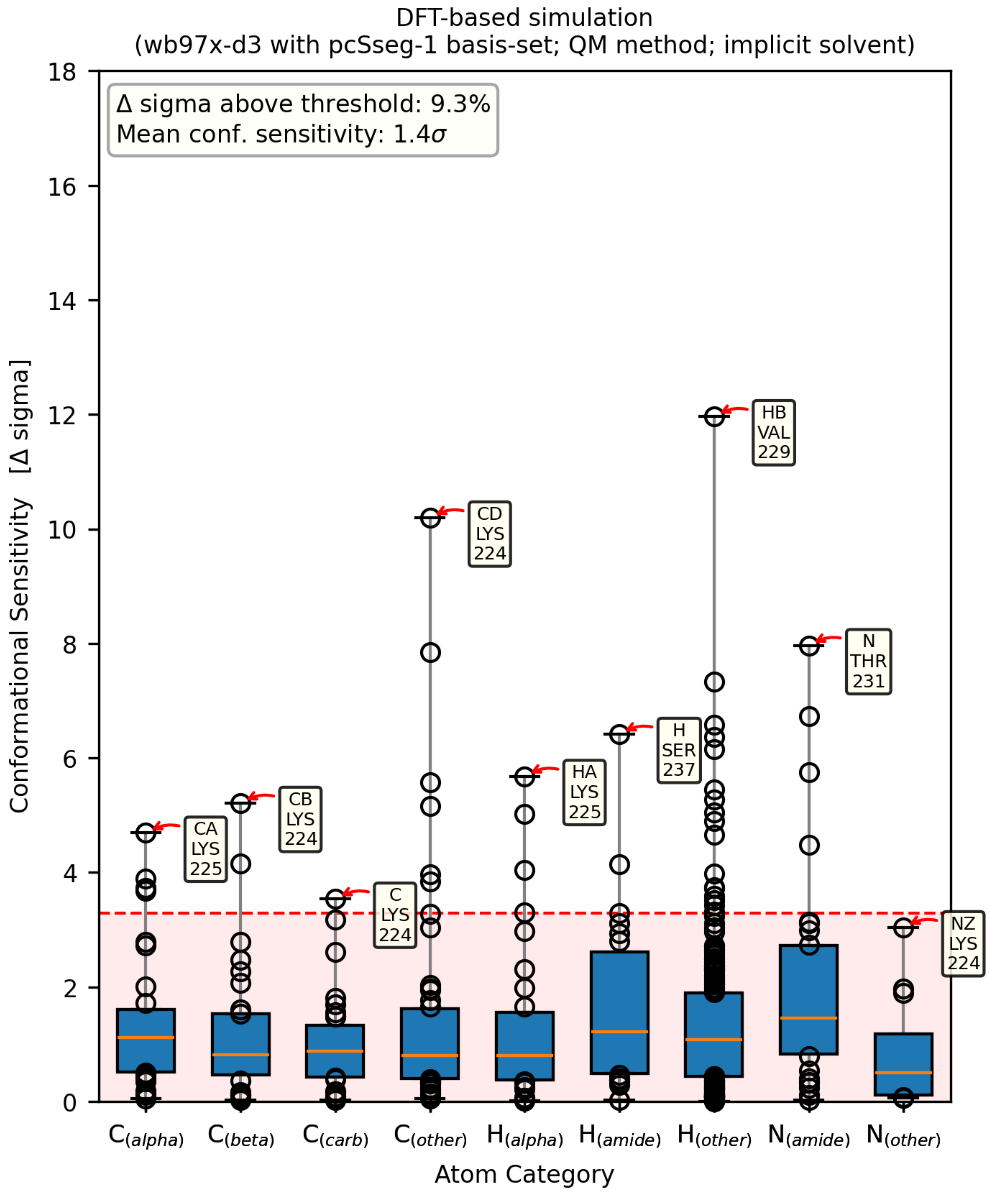

Figure S4.34: Conformational sensitivity calculated with the DFT-based QM method using wb97x-d3/pcSseg-1 theory with implicit solvent.

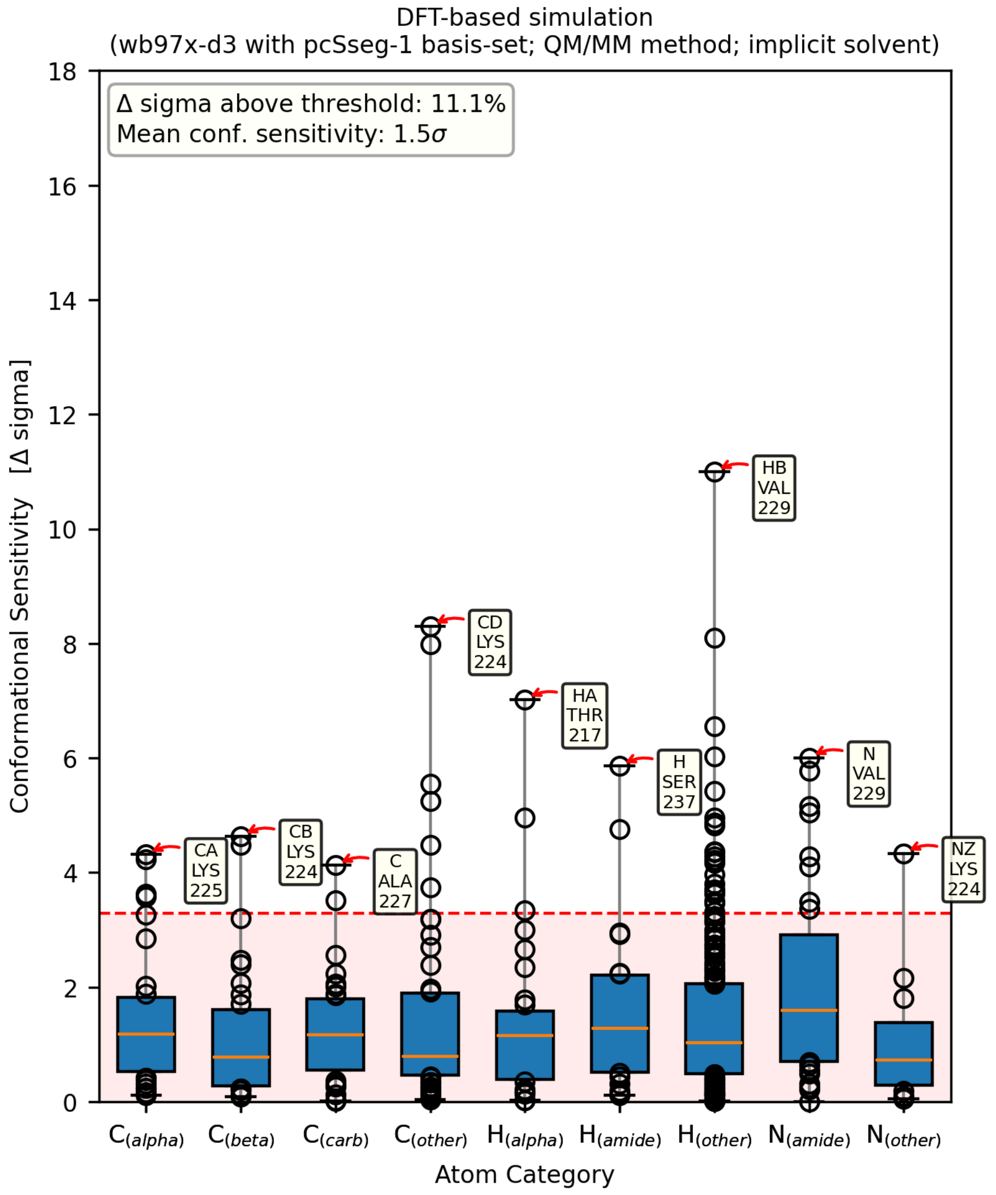

Figure S4.35: Conformational sensitivity calculated with the DFT-based QM/MM method using wb97x-d3/pcSseg-1 theory with implicit solvent.

1.5 Relationship of Chemical Shift and Backbone Torsion

The following figures show the influence of backbone torsion angle change $\Delta\phi$ and $\Delta\psi$ on the conformational sensitivity.

A table of content to find each graphic can be found in table 2.

Table 2: Table of content for the relationship of chemical shift and backbone torsion.

Name	Type	Solvation	Figure	Page
PPM	empirical	vacuum	S5.1	44
UCBShiftX	empirical	vacuum	S5.2	45
shiftX2	empirical	vacuum	S5.3	46
sparta+	empirical	vacuum	S5.4	47
b3lyp/6-31G*	QM/MM	implicit	S5.6	49
b3lyp/6-31G*	QM	implicit	S5.6	49
b3lyp/cc-pvdz	QM/MM	vacuum	S5.10	53
b3lyp/cc-pvdz	QM	explicit	S5.8	51
b3lyp/cc-pvdz	QM/MM	implicit	S5.11	54
b3lyp/cc-pvdz	QM	vacuum	S5.10	53
b3lyp/cc-pvdz	QM	implicit	S5.11	54
b3lyp/pcSseg-1	QM	implicit	S5.13	56
b3lyp/pcSseg-1	QM/MM	implicit	S5.13	56
becke97-2/6-31G*	QM/MM	implicit	S5.15	58
becke97-2/6-31G*	QM	implicit	S5.15	58
becke97-2/cc-pvdz	QM	implicit	S5.17	60
becke97-2/cc-pvdz	QM/MM	implicit	S5.17	60
becke97-2/pcSseg-1	QM	implicit	S5.19	62
becke97-2/pcSseg-1	QM/MM	implicit	S5.19	62
becke97-d/6-31G*	QM	implicit	S5.21	64
becke97-d/6-31G*	QM/MM	implicit	S5.21	64
becke97-d/cc-pvdz	QM/MM	implicit	S5.23	66
becke97-d/cc-pvdz	QM	implicit	S5.23	66
becke97-d/pcSseg-1	QM/MM	implicit	S5.26	69
becke97-d/pcSseg-1	QM	explicit	S5.25	68
becke97-d/pcSseg-1	QM	implicit	S5.26	69
wb97x-d3/6-31G*	QM	implicit	S5.28	71
wb97x-d3/6-31G*	QM/MM	implicit	S5.28	71
wb97x-d3/cc-pvdz	QM	explicit	S5.29	72
wb97x-d3/cc-pvdz	QM/MM	implicit	S5.31	74
wb97x-d3/cc-pvdz	QM	implicit	S5.31	74
wb97x-d3/cc-pvdz	QM	vacuum	S5.33	76
wb97x-d3/cc-pvdz	QM/MM	vacuum	S5.33	76
wb97x-d3/pcSseg-1	QM	implicit	S5.35	78
wb97x-d3/pcSseg-1	QM/MM	implicit	S5.35	78

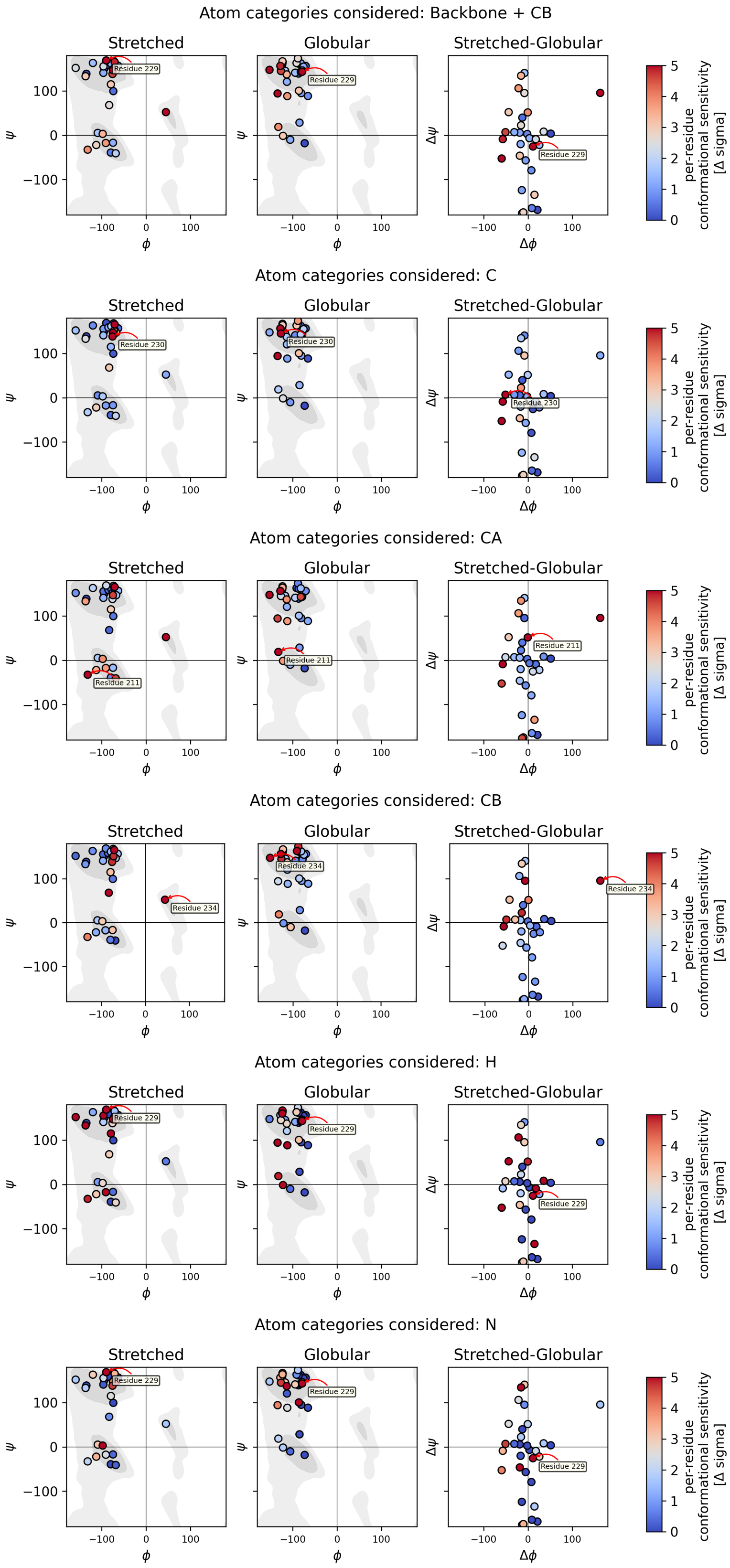

Figure S5.1: Influence of $\Delta\phi$ and $\Delta\psi$ on the conformational sensitivity calculated with the empirical method PPM.

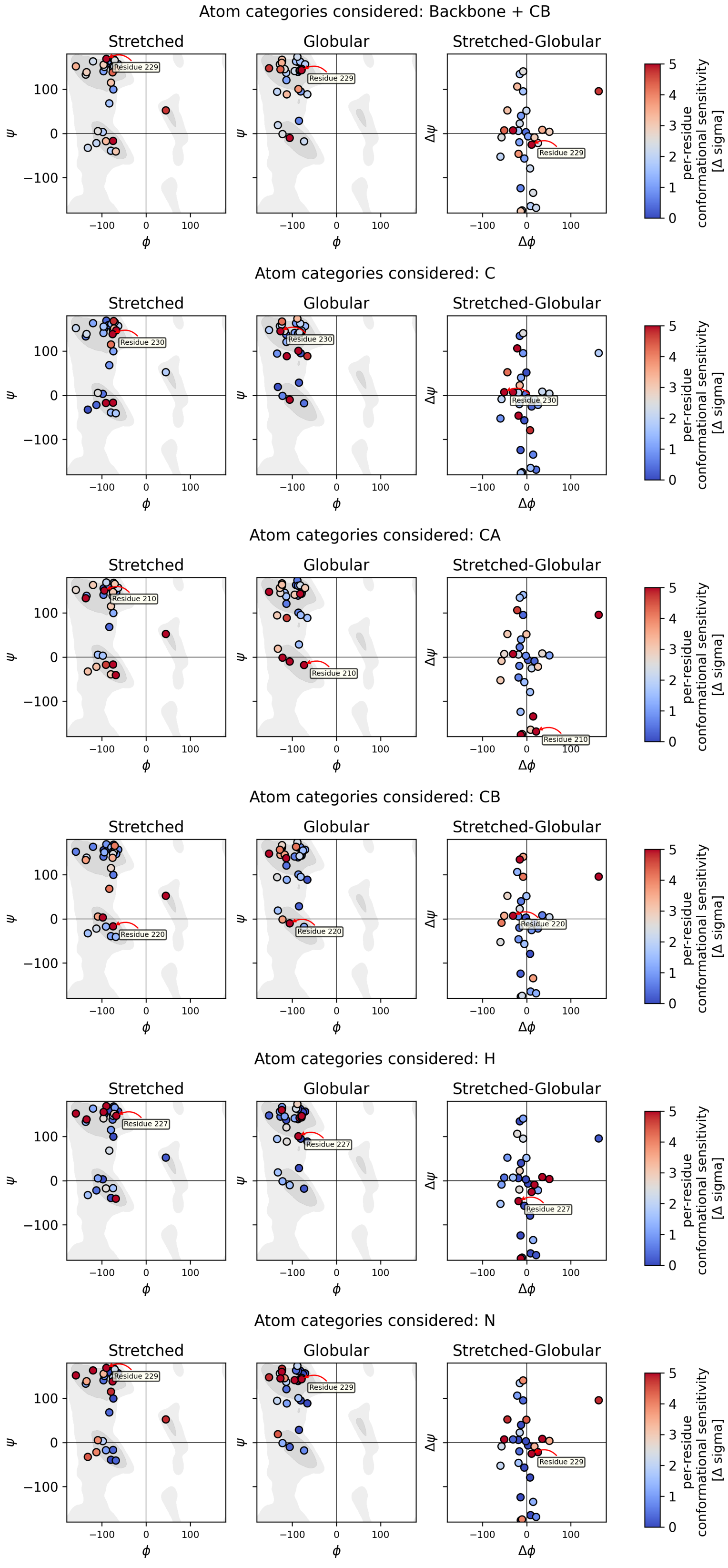

Figure S5.2: Influence of $\Delta\phi$ and $\Delta\psi$ on the conformational sensitivity calculated with the empirical method UCBShiftX.

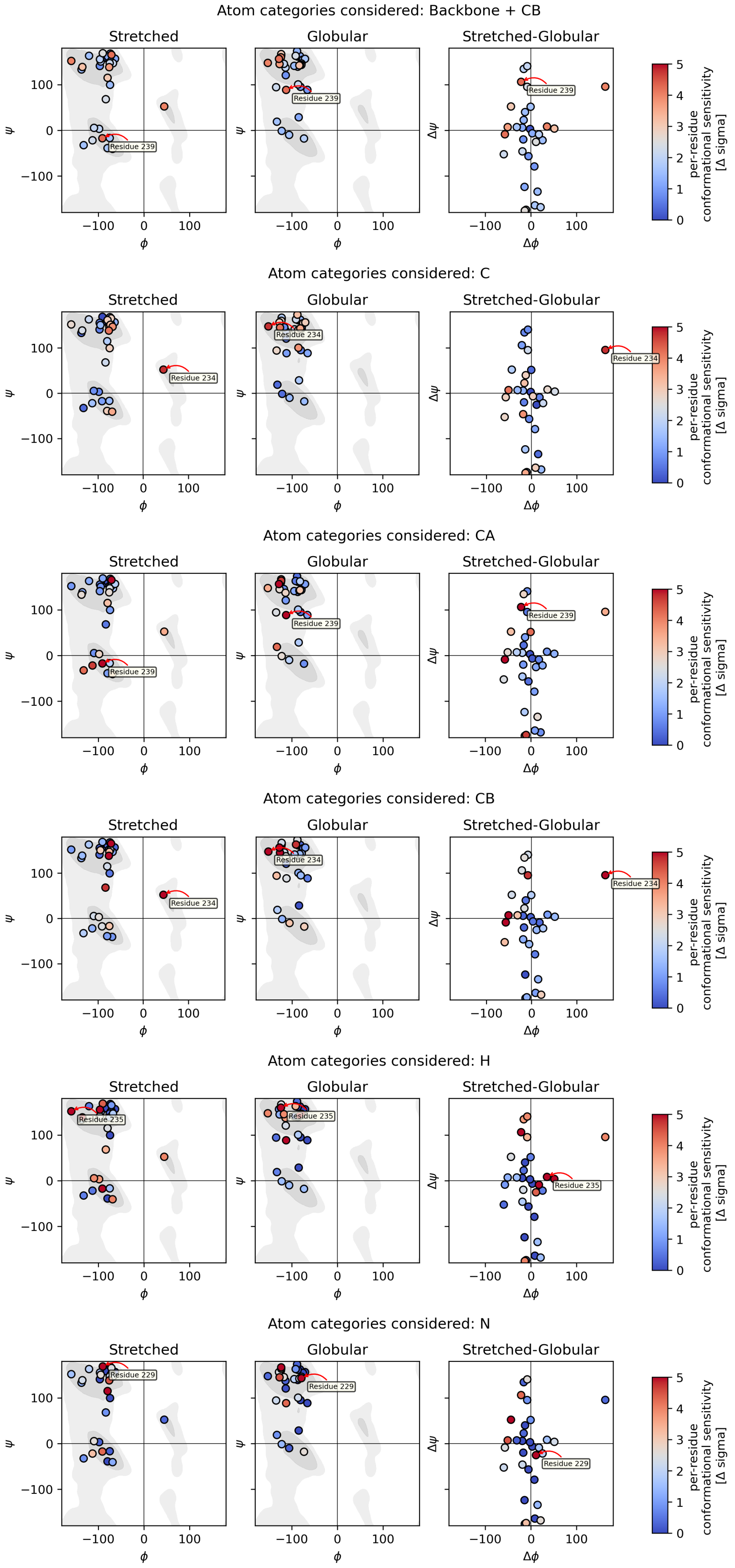

Figure S5.3: Influence of $\Delta\phi$ and $\Delta\psi$ on the conformational sensitivity calculated with the empirical method shiftX2.

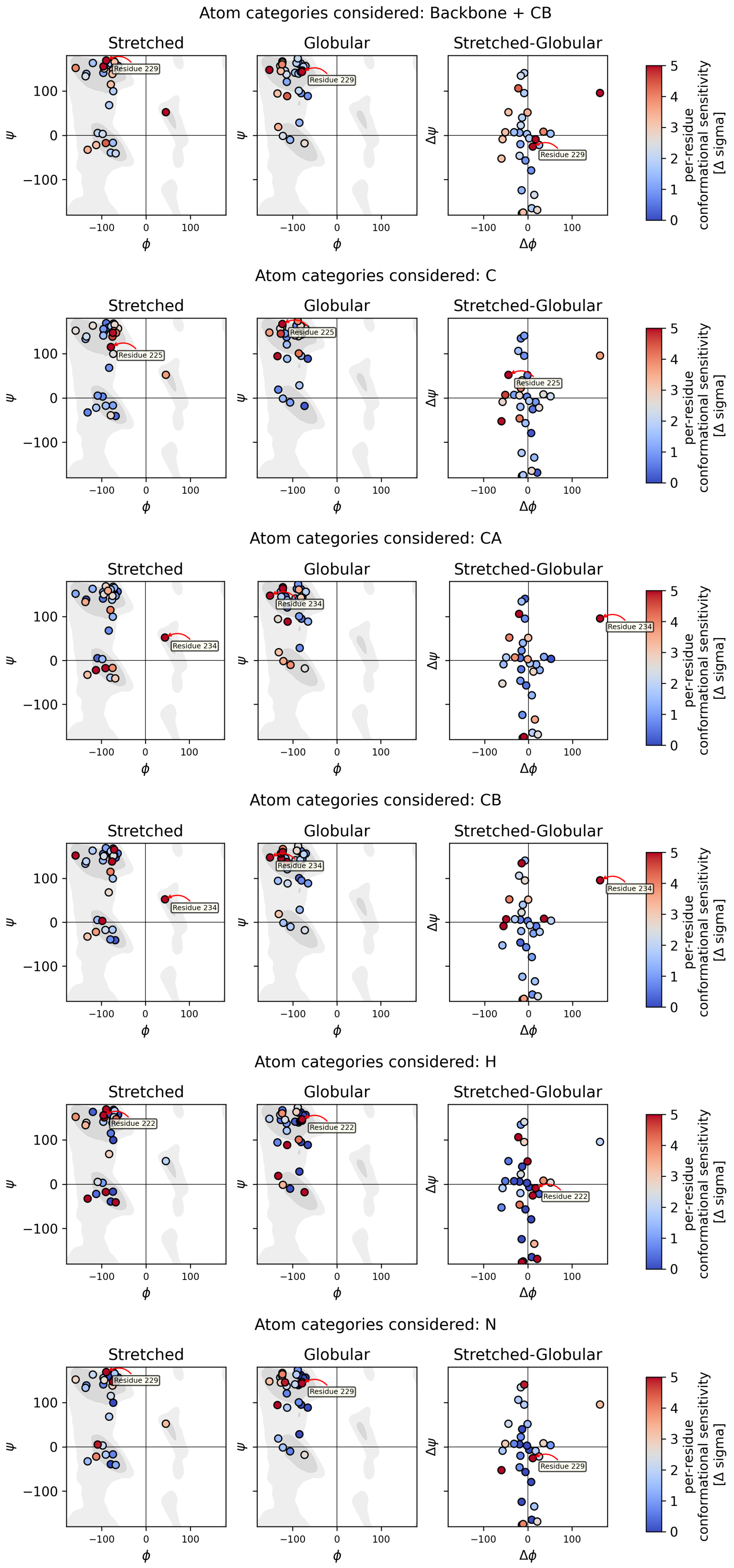

Figure S5.4: Influence of $\Delta\phi$ and $\Delta\psi$ on the conformational sensitivity calculated with the empirical method sparta+.

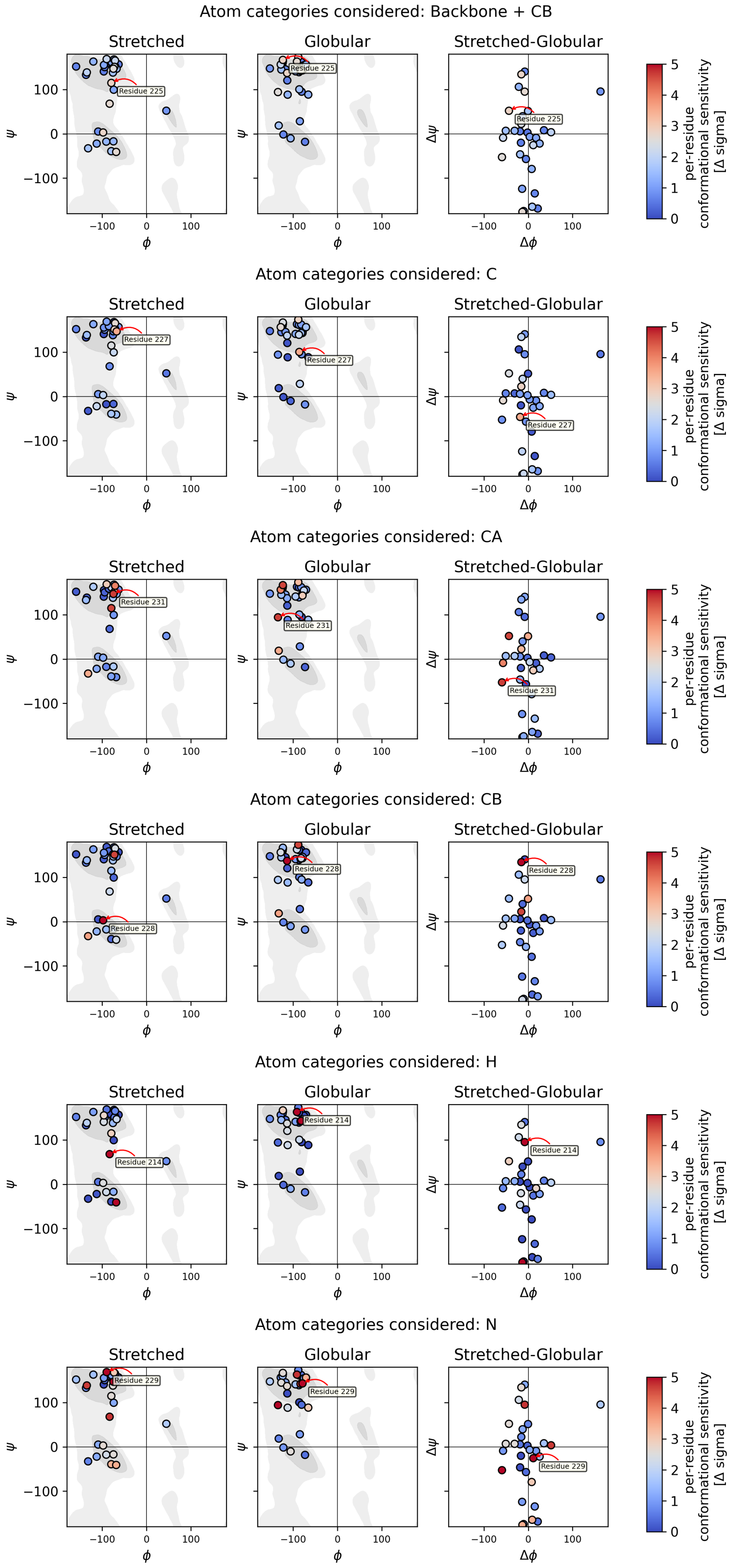

Figure S5.5: Influence of $\Delta\phi$ and $\Delta\psi$ on the conformational sensitivity calculated with the DFT-based QM/MM method using b3lyp/6-31G* theory with implicit solvent.

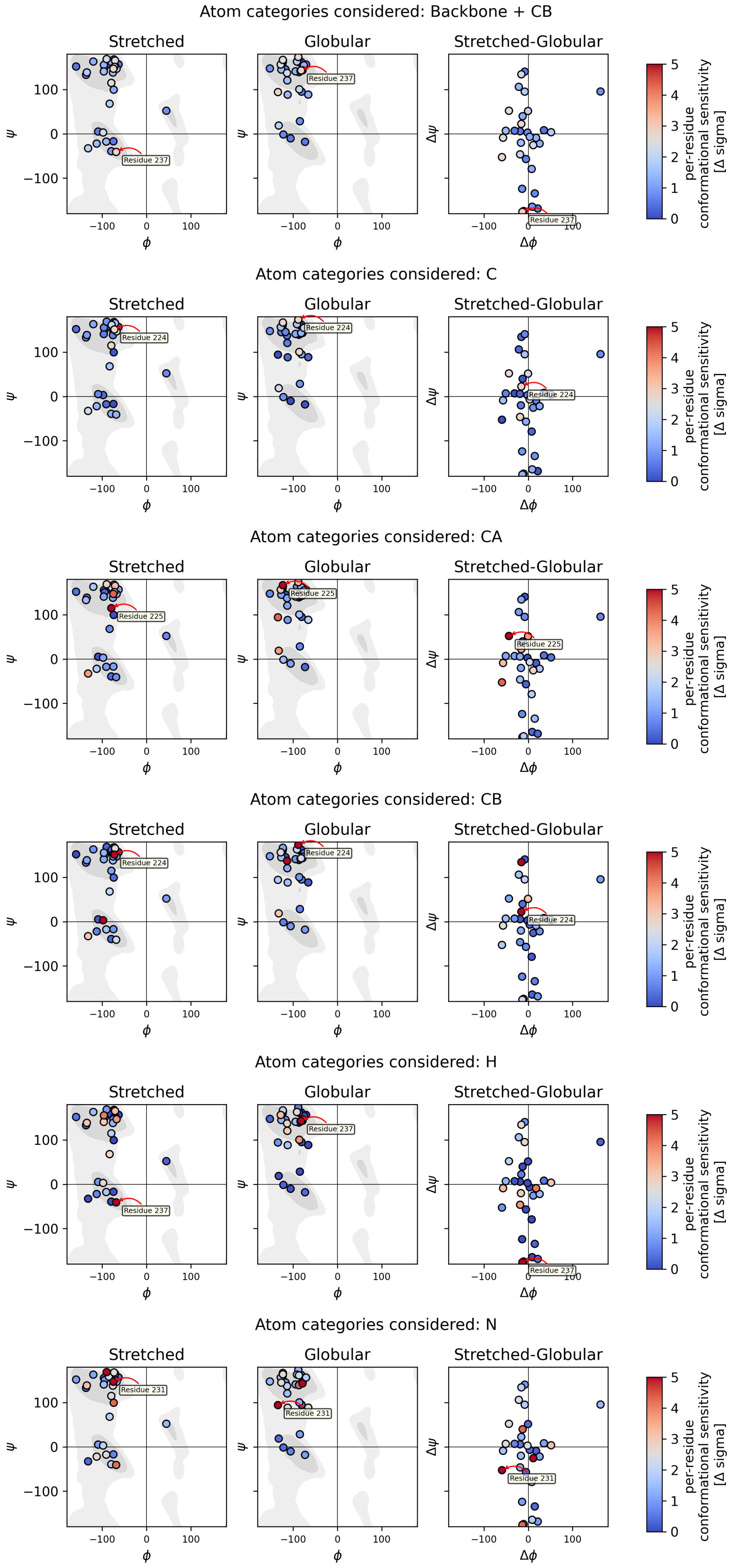

Figure S5.6: Influence of $\Delta\phi$ and $\Delta\psi$ on the conformational sensitivity calculated with the DFT-based QM method using b3lyp/6-31G* theory with implicit solvent.

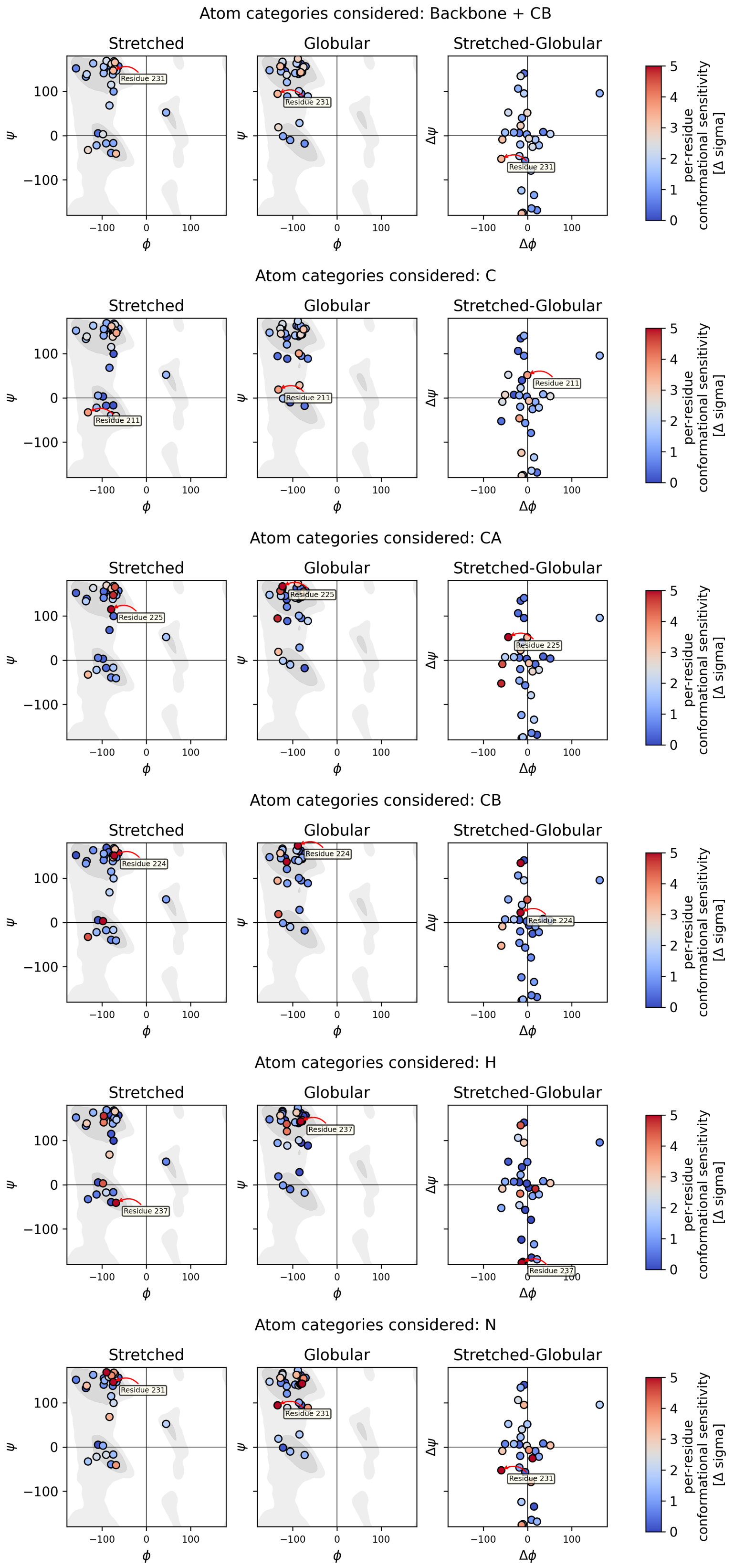

Figure S5.7: Influence of $\Delta\phi$ and $\Delta\psi$ on the conformational sensitivity calculated with the DFT-based QM/MM method using b3lyp/cc-pvdz theory in vacuum.

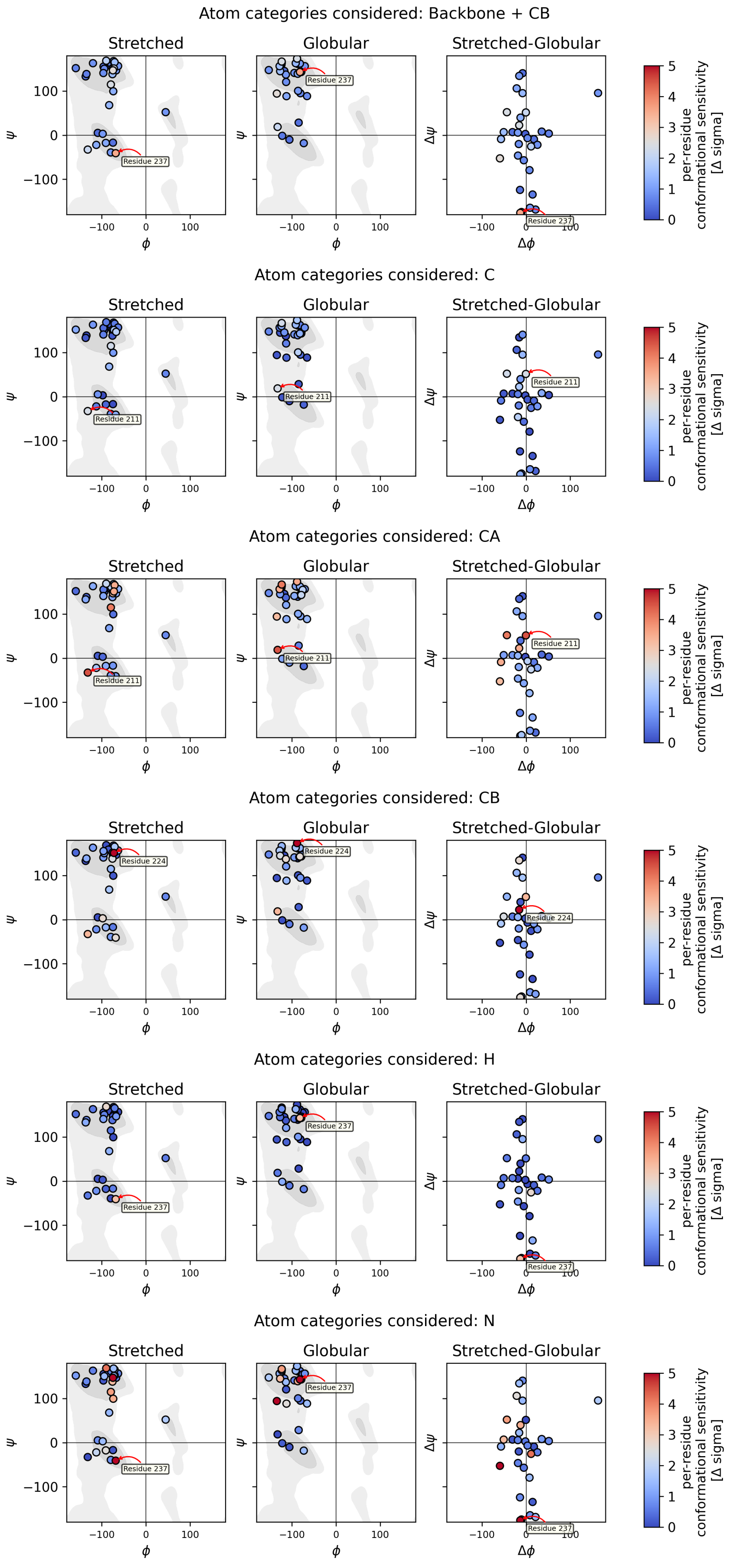

Figure S5.8: Influence of $\Delta\phi$ and $\Delta\psi$ on the conformational sensitivity calculated with the DFT-based QM method using b3lyp/cc-pvdz theory with explicit solvent.

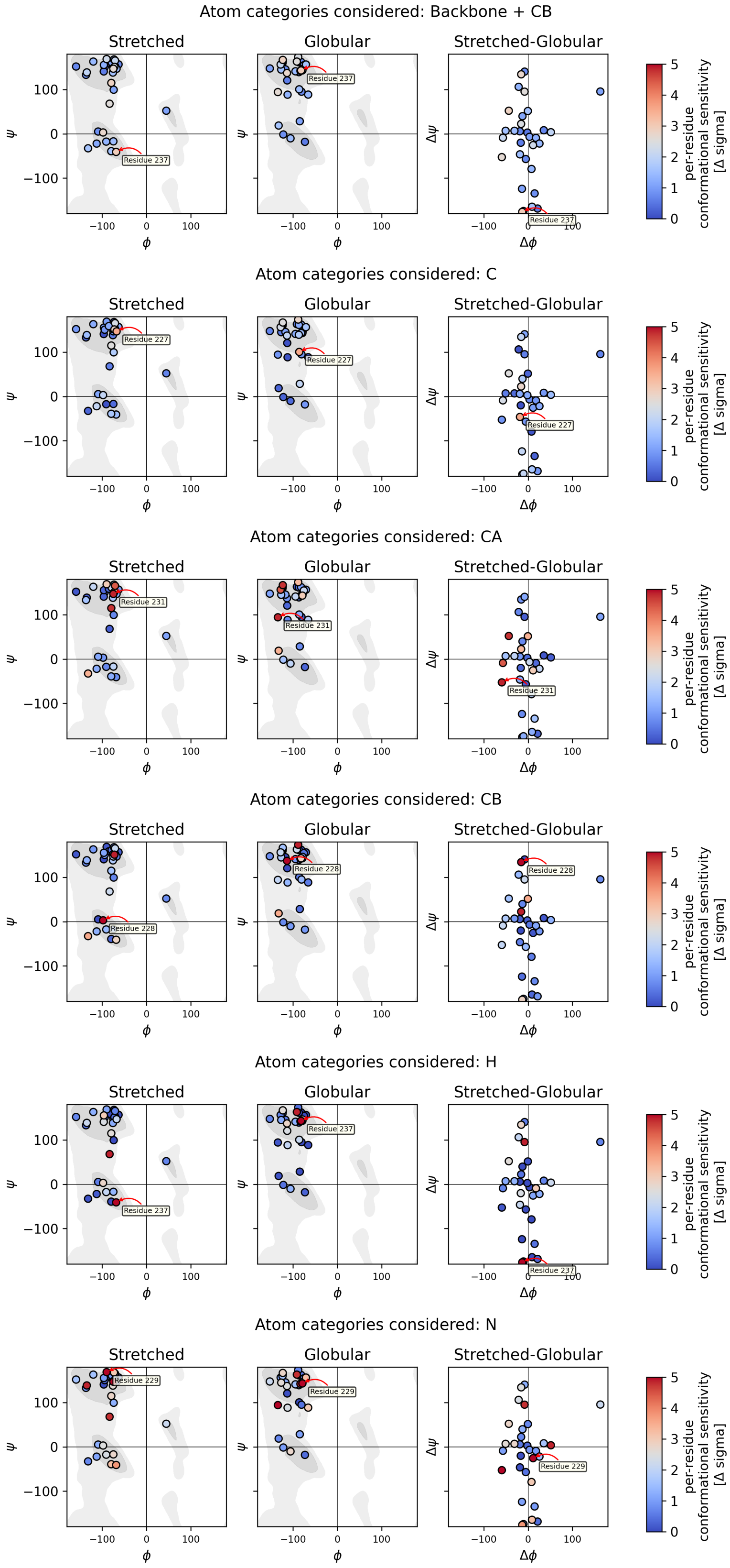

Figure S5.9: Influence of $\Delta\phi$ and $\Delta\psi$ on the conformational sensitivity calculated with the DFT-based QM/MM method using b3lyp/cc-pvdz theory with implicit solvent.

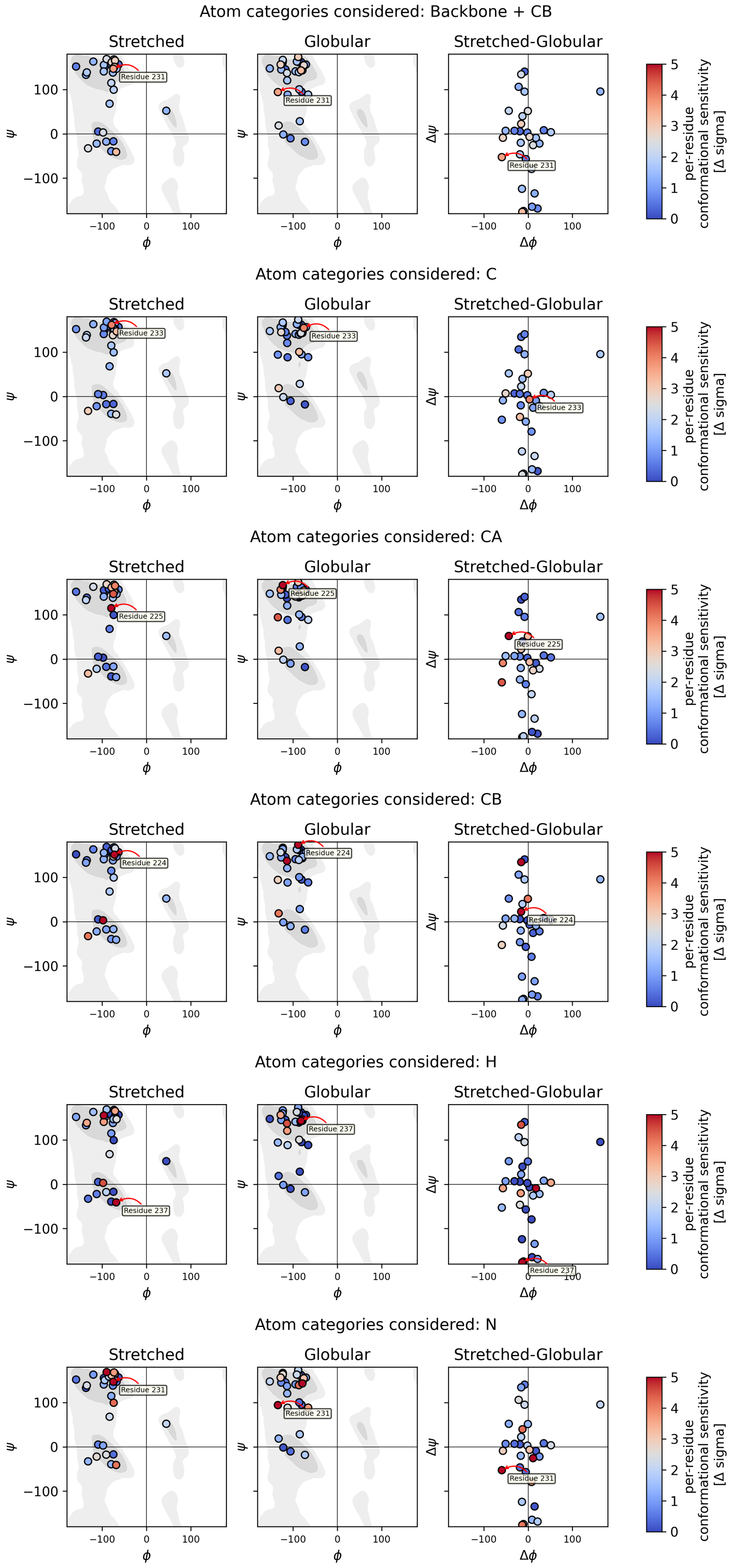

Figure S5.10: Influence of $\Delta\phi$ and $\Delta\psi$ on the conformational sensitivity calculated with the DFT-based QM method using b3lyp/cc-pvdz theory in vacuum.

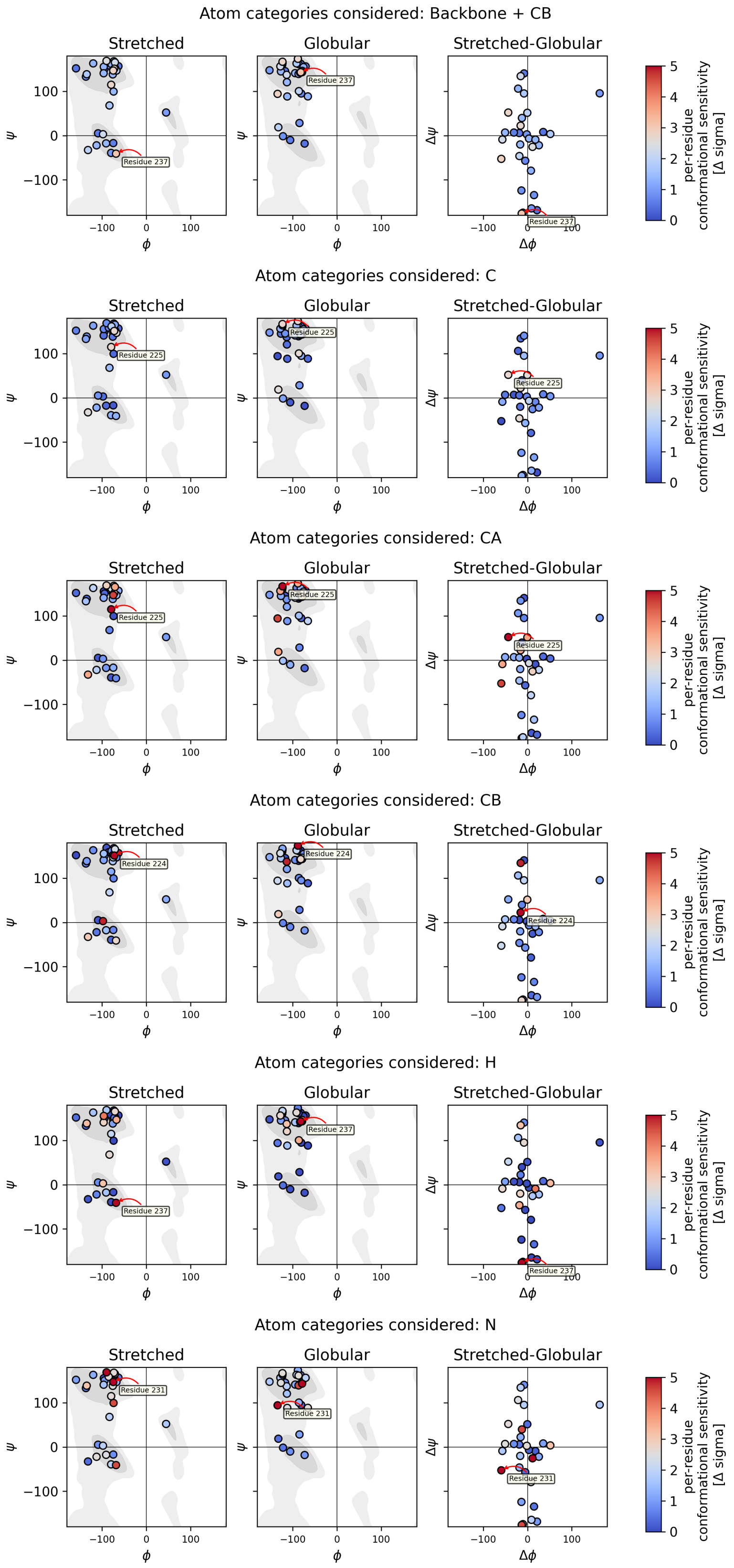

Figure S5.11: Influence of $\Delta\phi$ and $\Delta\psi$ on the conformational sensitivity calculated with the DFT-based QM method using b3lyp/cc-pvdz theory with implicit solvent.

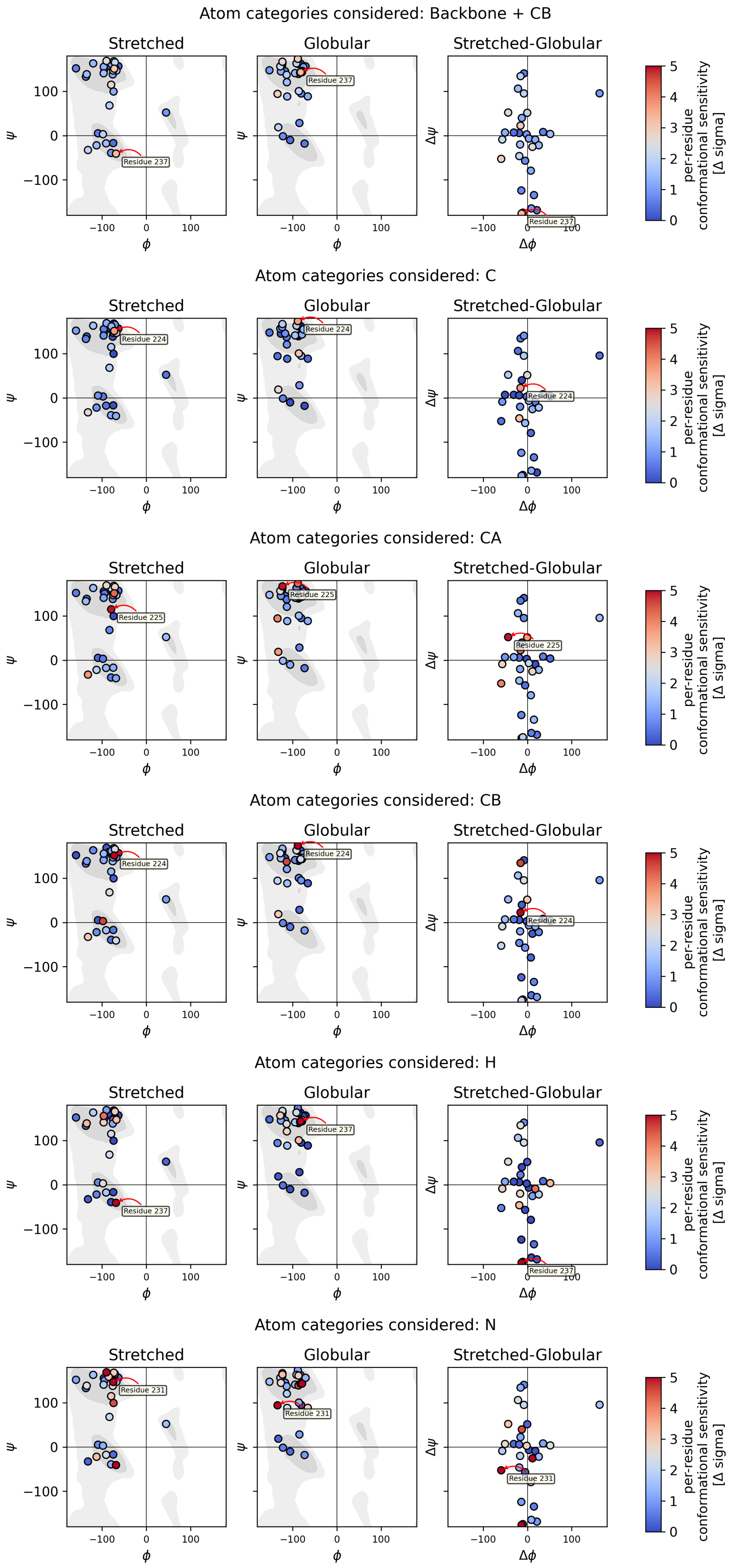

Figure S5.12: Influence of $\Delta\phi$ and $\Delta\psi$ on the conformational sensitivity calculated with the DFT-based QM method using b3lyp/pcSseg-1 theory with implicit solvent.

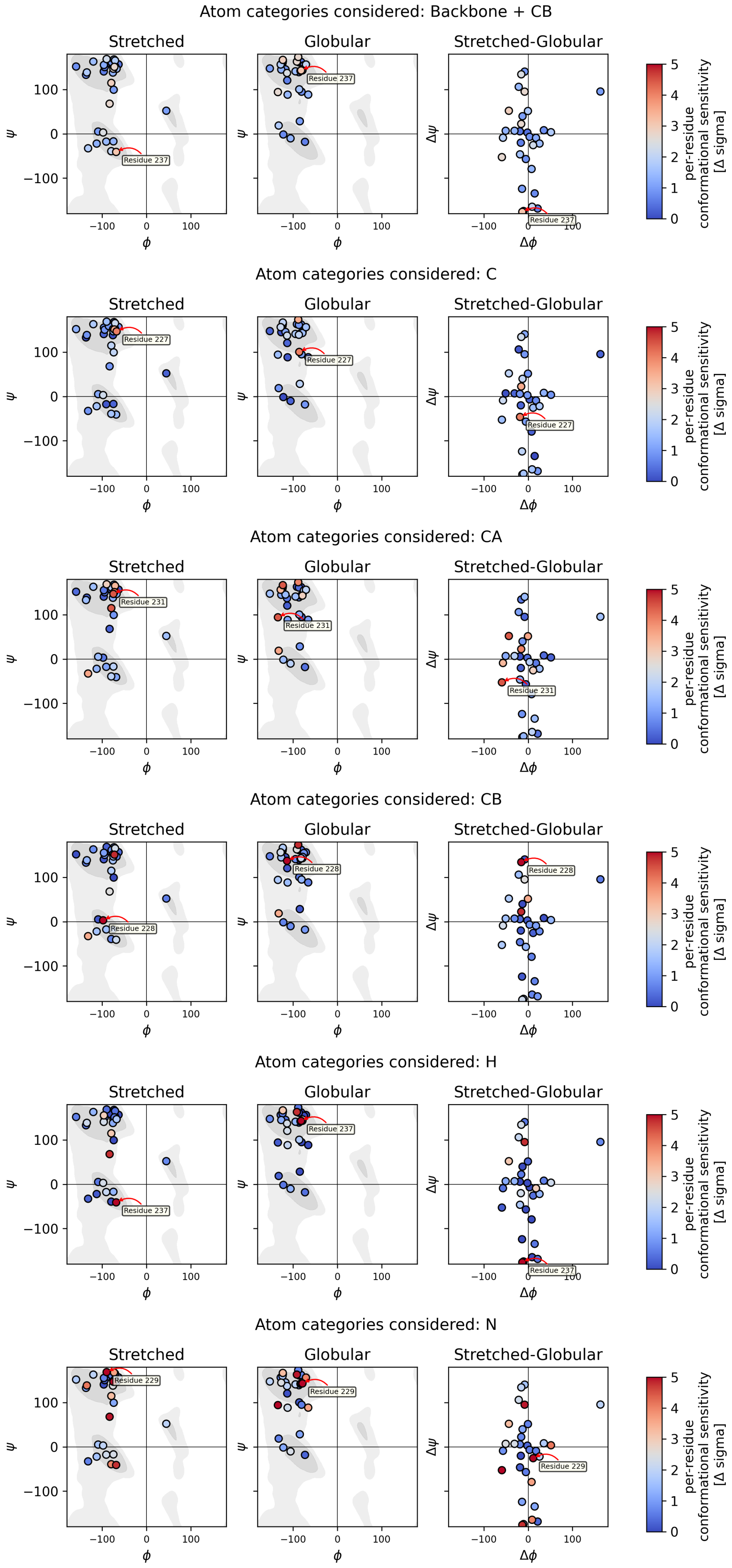

Figure S5.13: Influence of $\Delta\phi$ and $\Delta\psi$ on the conformational sensitivity calculated with the DFT-based QM/MM method using b3lyp/pcSseg-1 theory with implicit solvent.

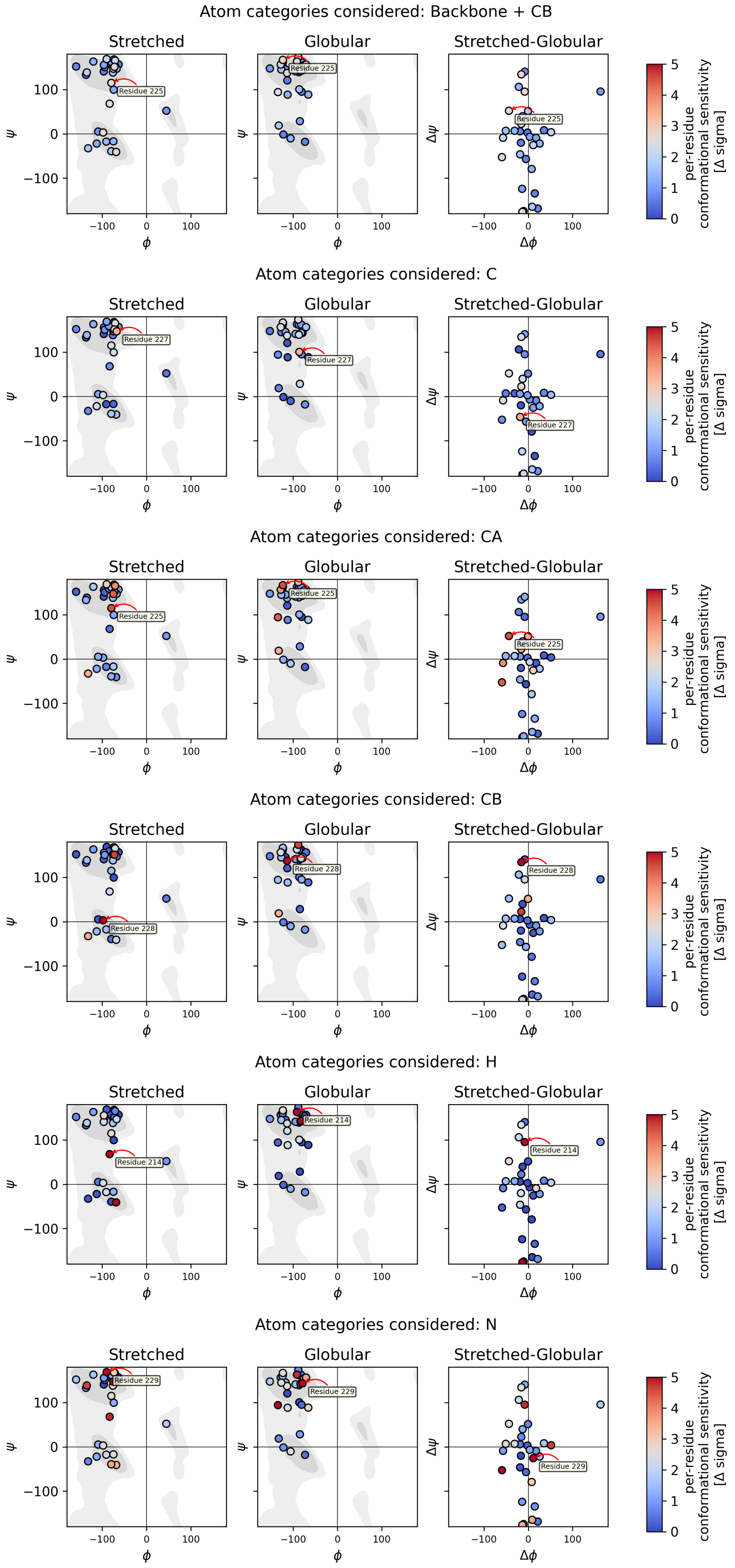

Figure S5.14: Influence of $\Delta\phi$ and $\Delta\psi$ on the conformational sensitivity calculated with the DFT-based QM/MM method using becke97-2/6-31G* theory with implicit solvent.

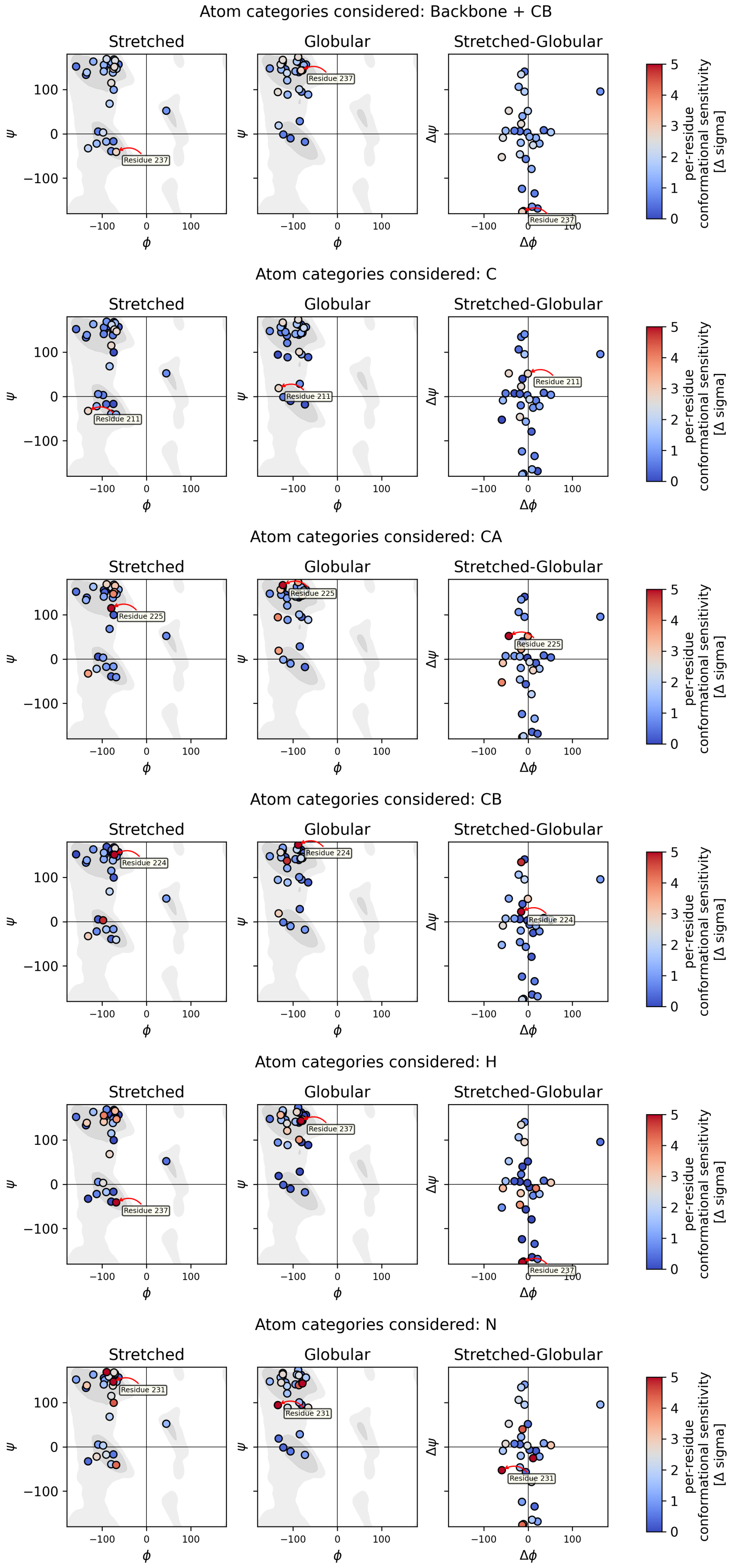

Figure S5.15: Influence of $\Delta\phi$ and $\Delta\psi$ on the conformational sensitivity calculated with the DFT-based QM method using becke97-2/6-31G* theory with implicit solvent.

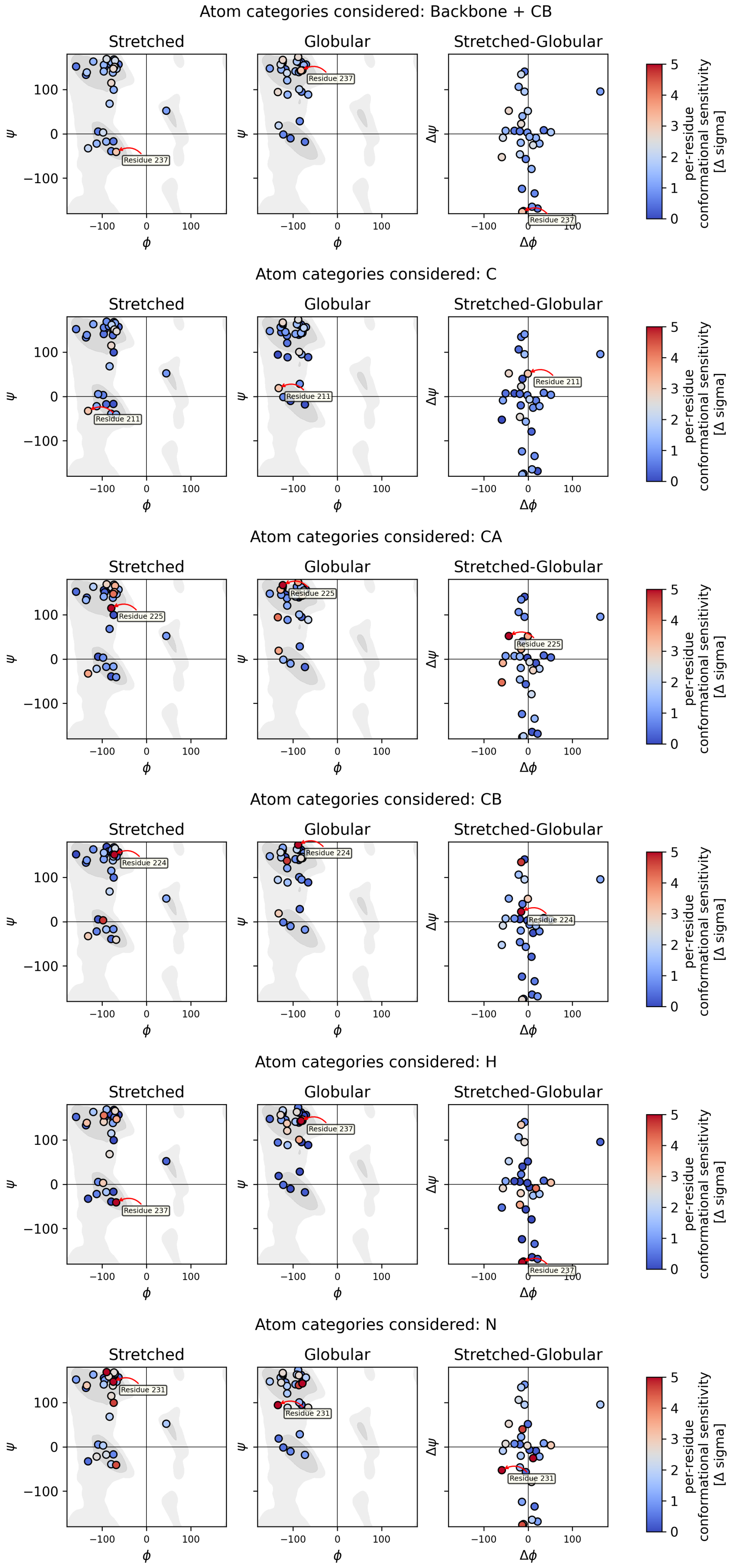

Figure S5.16: Influence of $\Delta\phi$ and $\Delta\psi$ on the conformational sensitivity calculated with the DFT-based QM method using becke97-2/cc-pvdz theory with implicit solvent.

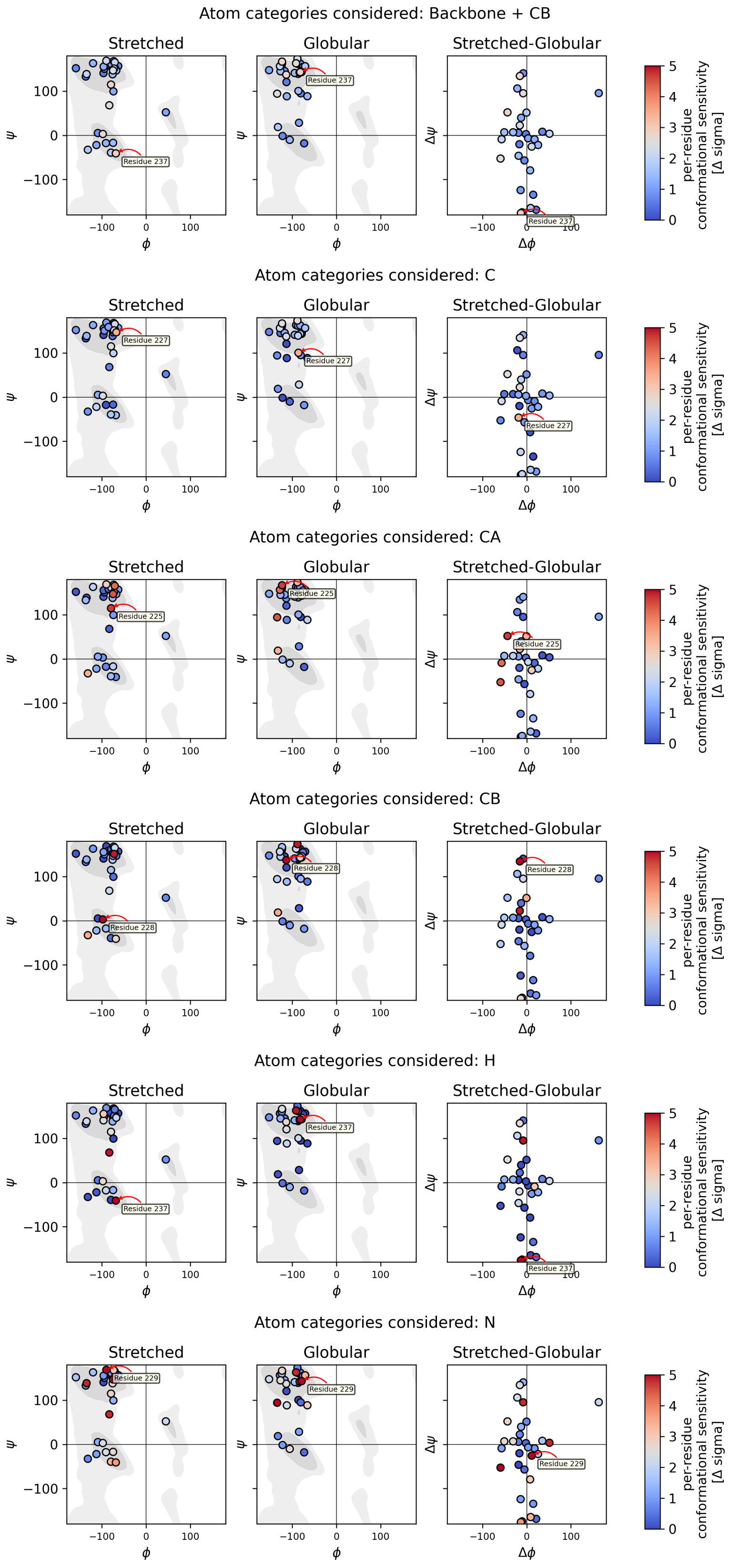

Figure S5.17: Influence of $\Delta\phi$ and $\Delta\psi$ on the conformational sensitivity calculated with the DFT-based QM/MM method using becke97-2/cc-pvdz theory with implicit solvent.

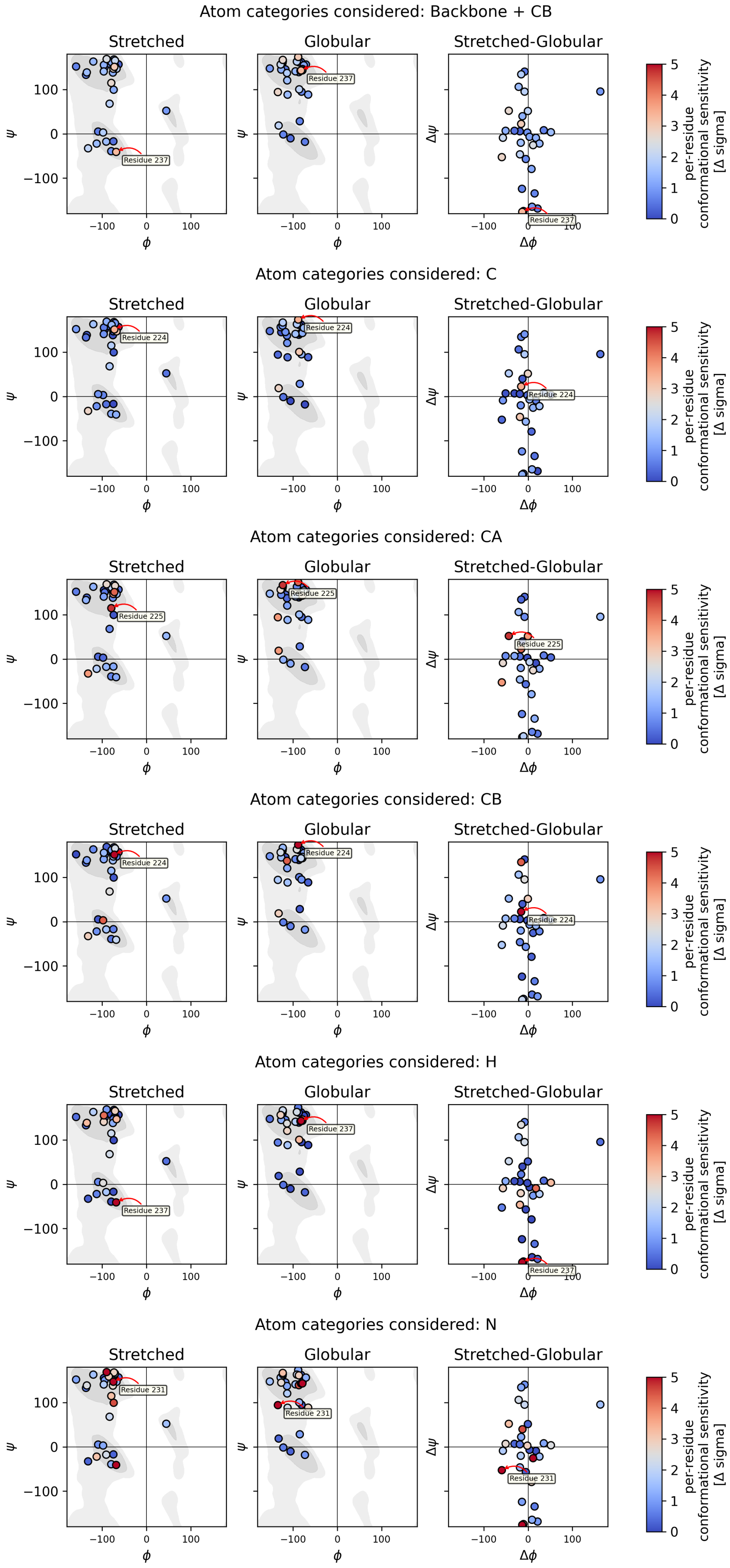

Figure S5.18: Influence of $\Delta\phi$ and $\Delta\psi$ on the conformational sensitivity calculated with the DFT-based QM method using becke97-2/pcSseg-1 theory with implicit solvent.

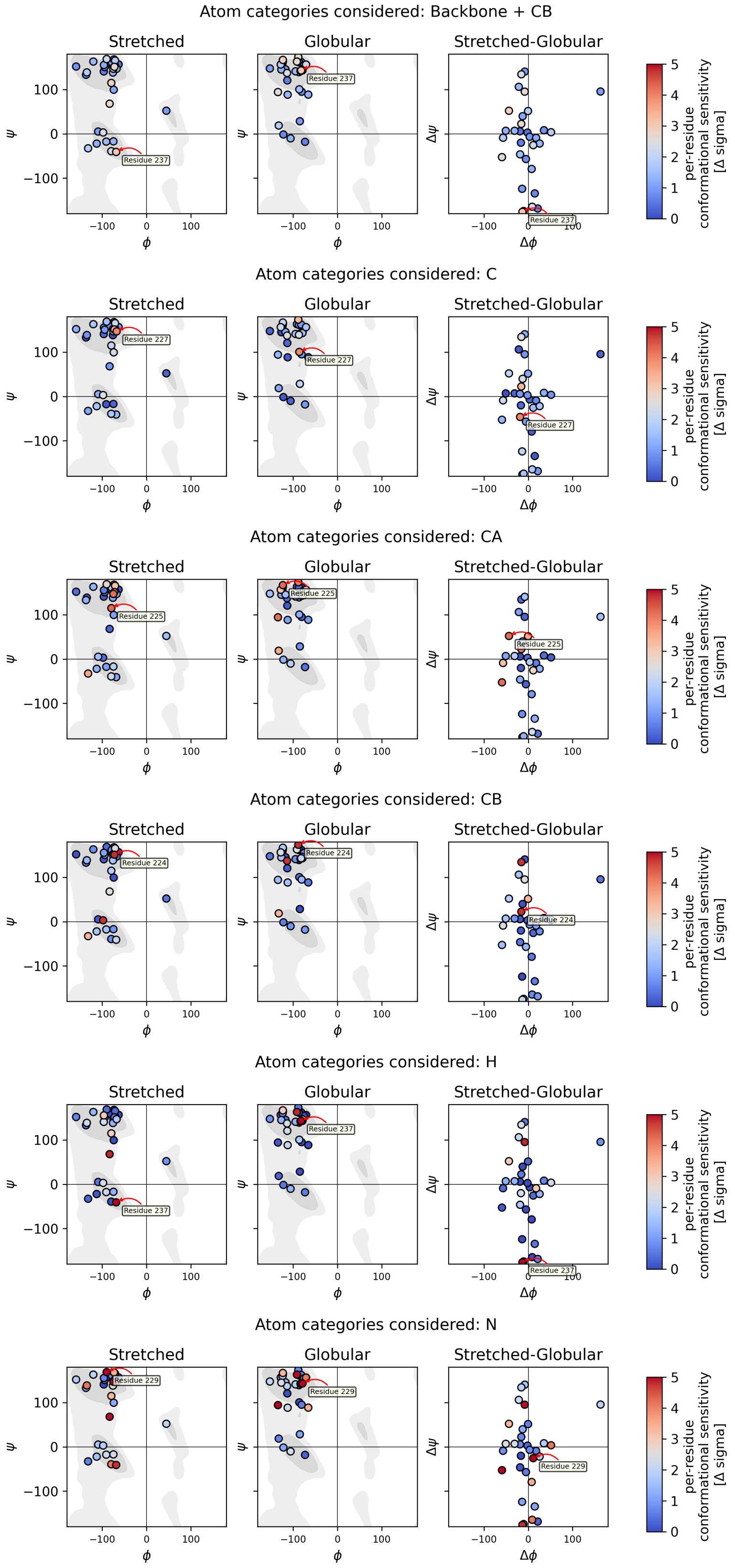

Figure S5.19: Influence of $\Delta\phi$ and $\Delta\psi$ on the conformational sensitivity calculated with the DFT-based QM/MM method using becke97-2/pcSseg-1 theory with implicit solvent.

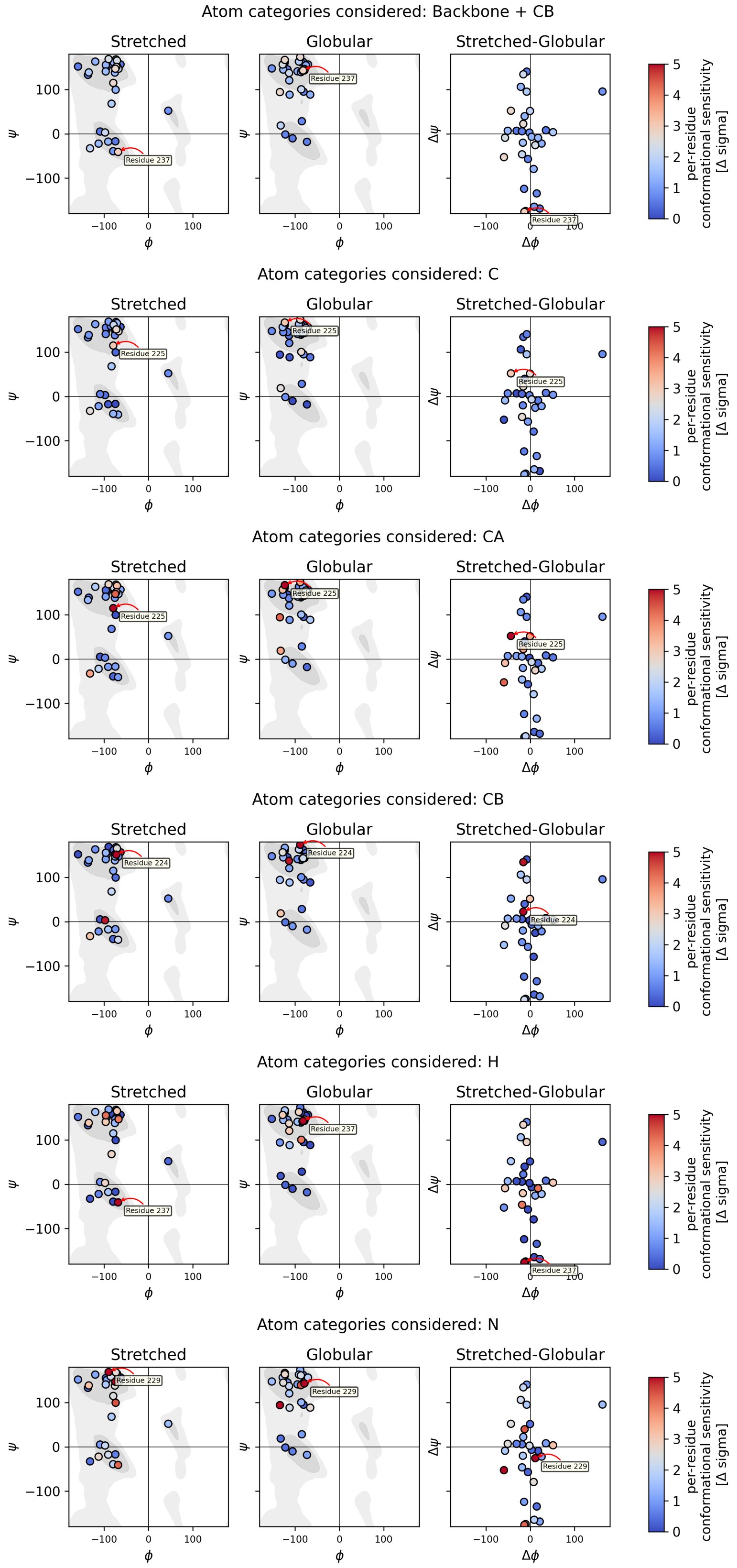

Figure S5.20: Influence of $\Delta\phi$ and $\Delta\psi$ on the conformational sensitivity calculated with the DFT-based QM method using becke97-d/6-31G* theory with implicit solvent.

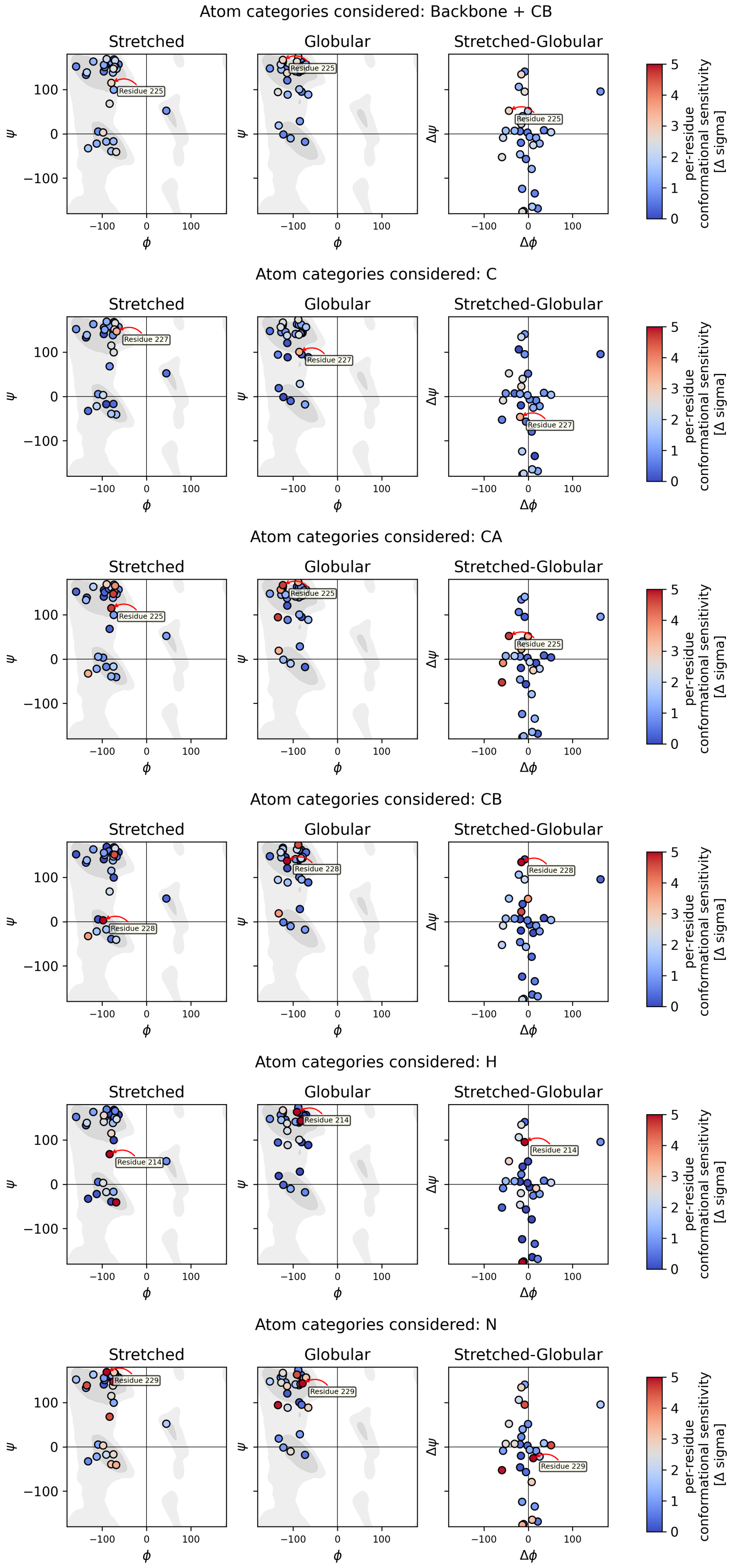

Figure S5.21: Influence of $\Delta\phi$ and $\Delta\psi$ on the conformational sensitivity calculated with the DFT-based QM/MM method using becke97-d/6-31G* theory with implicit solvent.

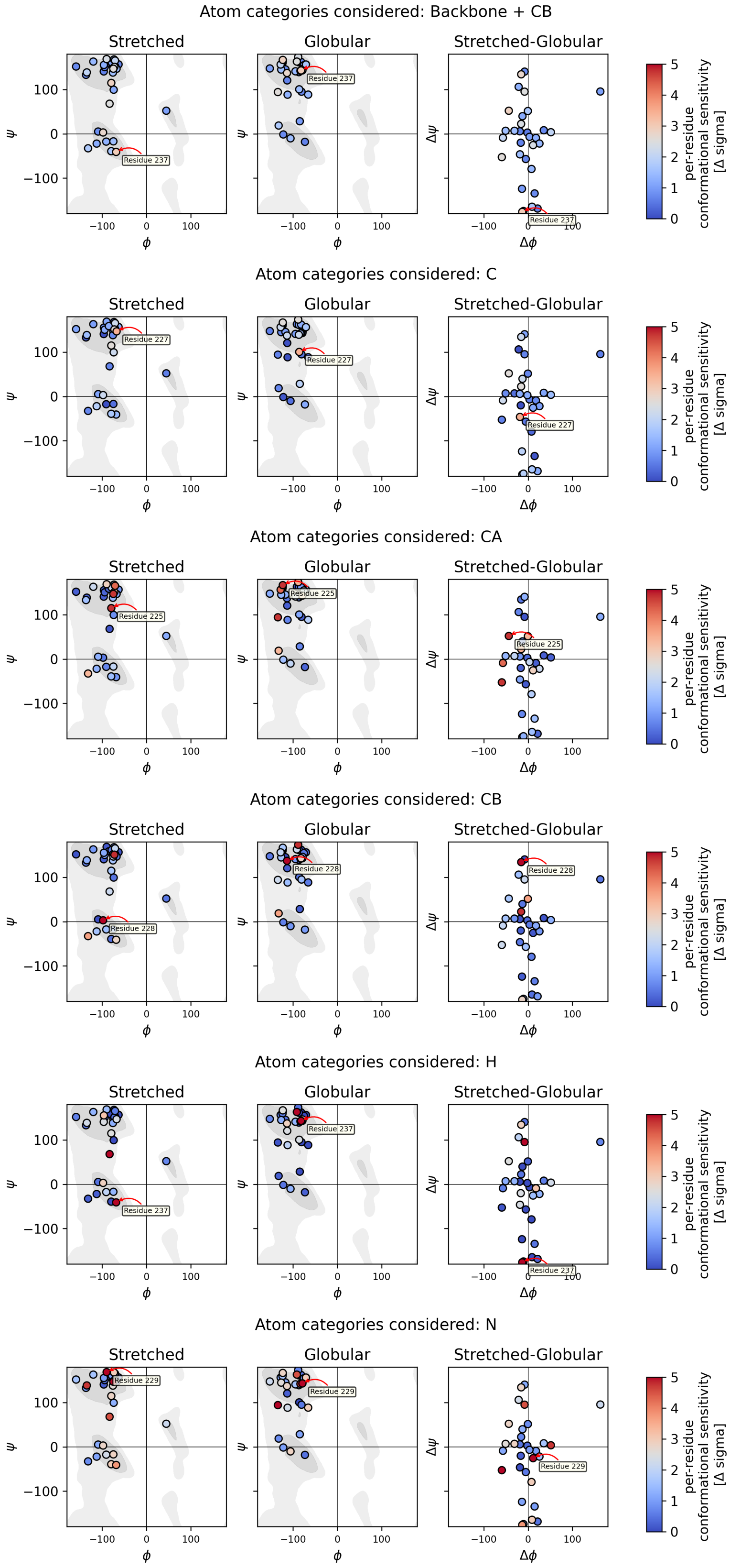

Figure S5.22: Influence of $\Delta\phi$ and $\Delta\psi$ on the conformational sensitivity calculated with the DFT-based QM/MM method using becke97-d/cc-pvdz theory with implicit solvent.

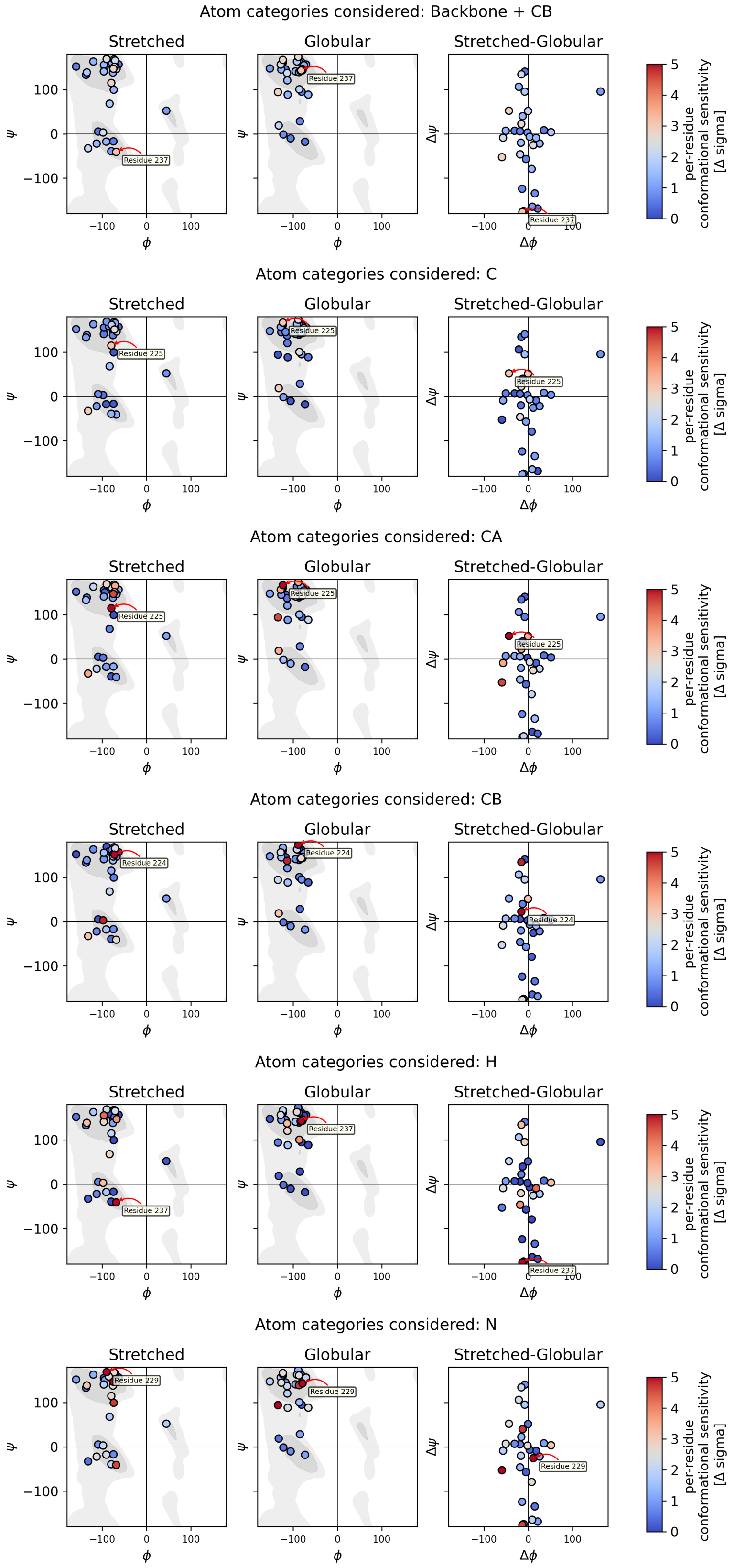

Figure S5.23: Influence of $\Delta\phi$ and $\Delta\psi$ on the conformational sensitivity calculated with the DFT-based QM method using becke97-d/cc-pvdz theory with implicit solvent.

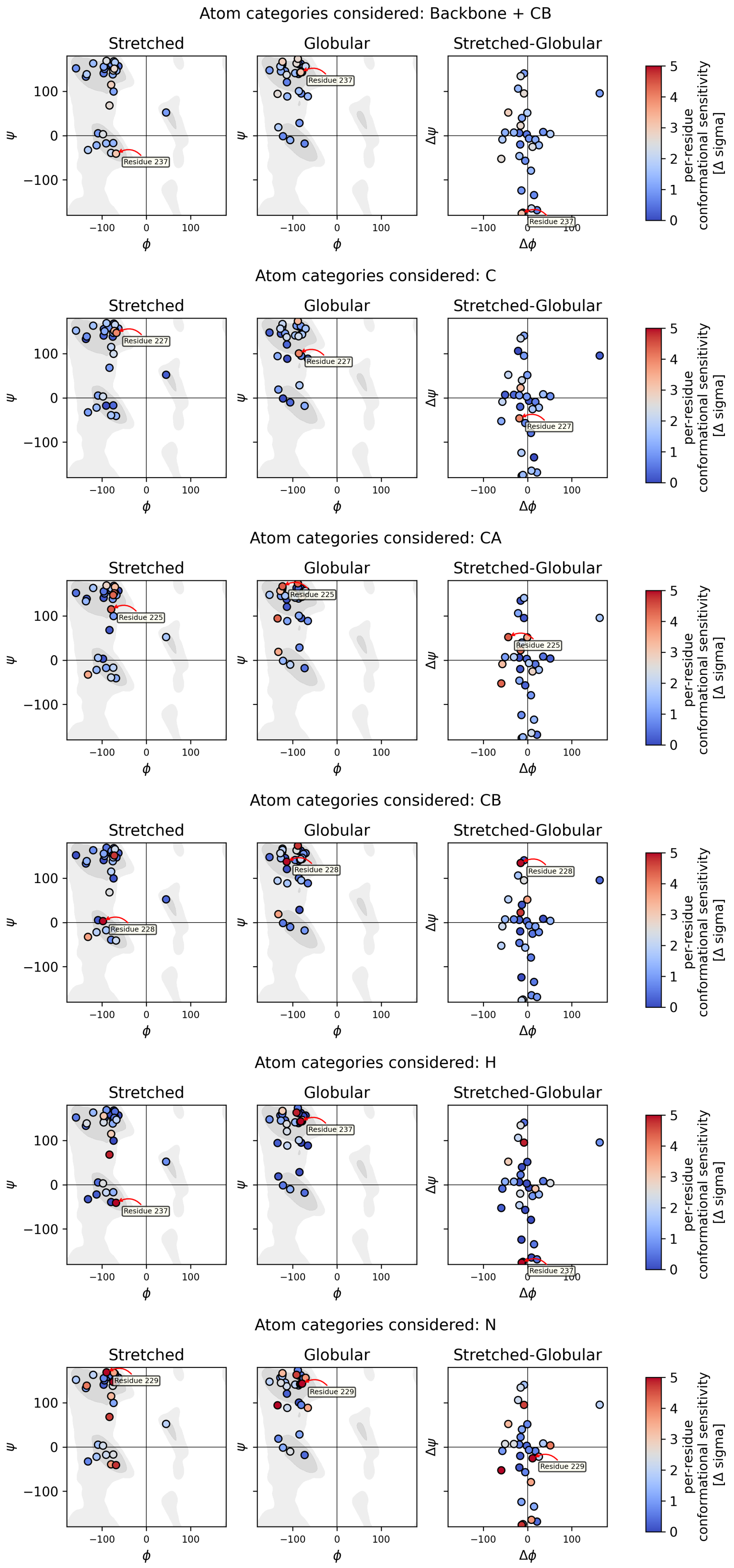

Figure S5.24: Influence of $\Delta\phi$ and $\Delta\psi$ on the conformational sensitivity calculated with the DFT-based QM/MM method using becke97-d/pcSseg-1 theory with implicit solvent.

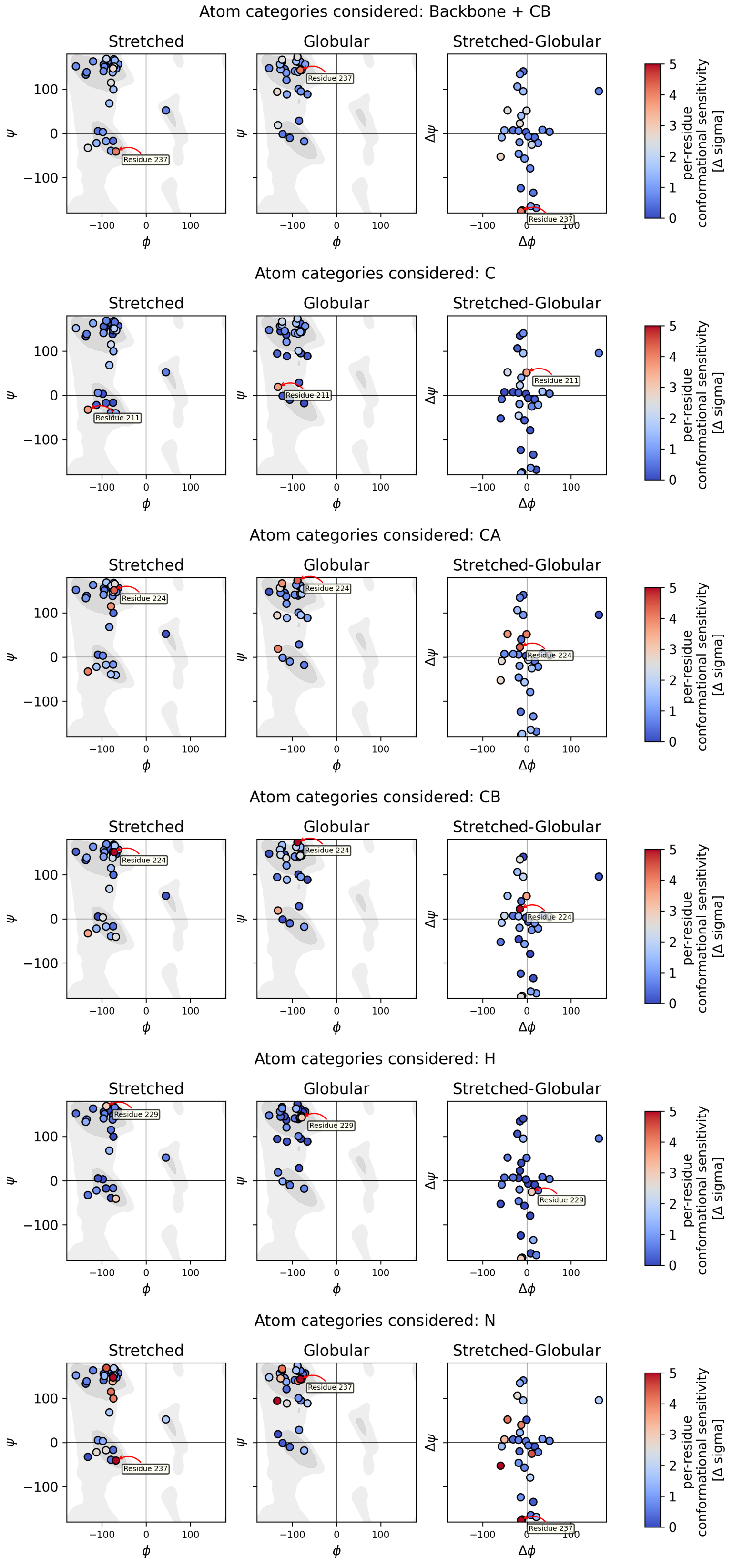

Figure S5.25: Influence of $\Delta\phi$ and $\Delta\psi$ on the conformational sensitivity calculated with the DFT-based QM method using becke97-d/pcSseg-1 theory with explicit solvent.

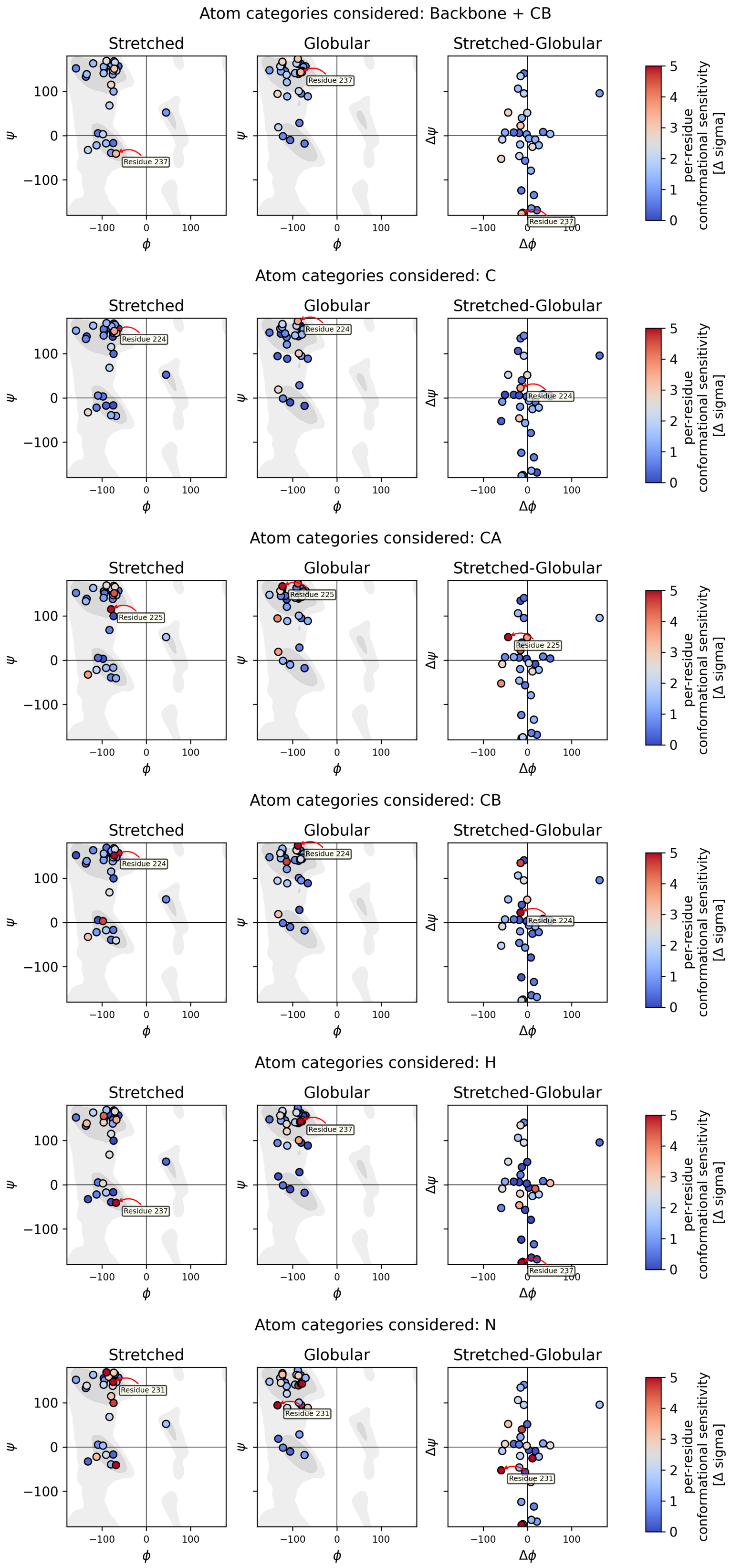

Figure S5.26: Influence of $\Delta\phi$ and $\Delta\psi$ on the conformational sensitivity calculated with the DFT-based QM method using becke97-d/pcSseg-1 theory with implicit solvent.

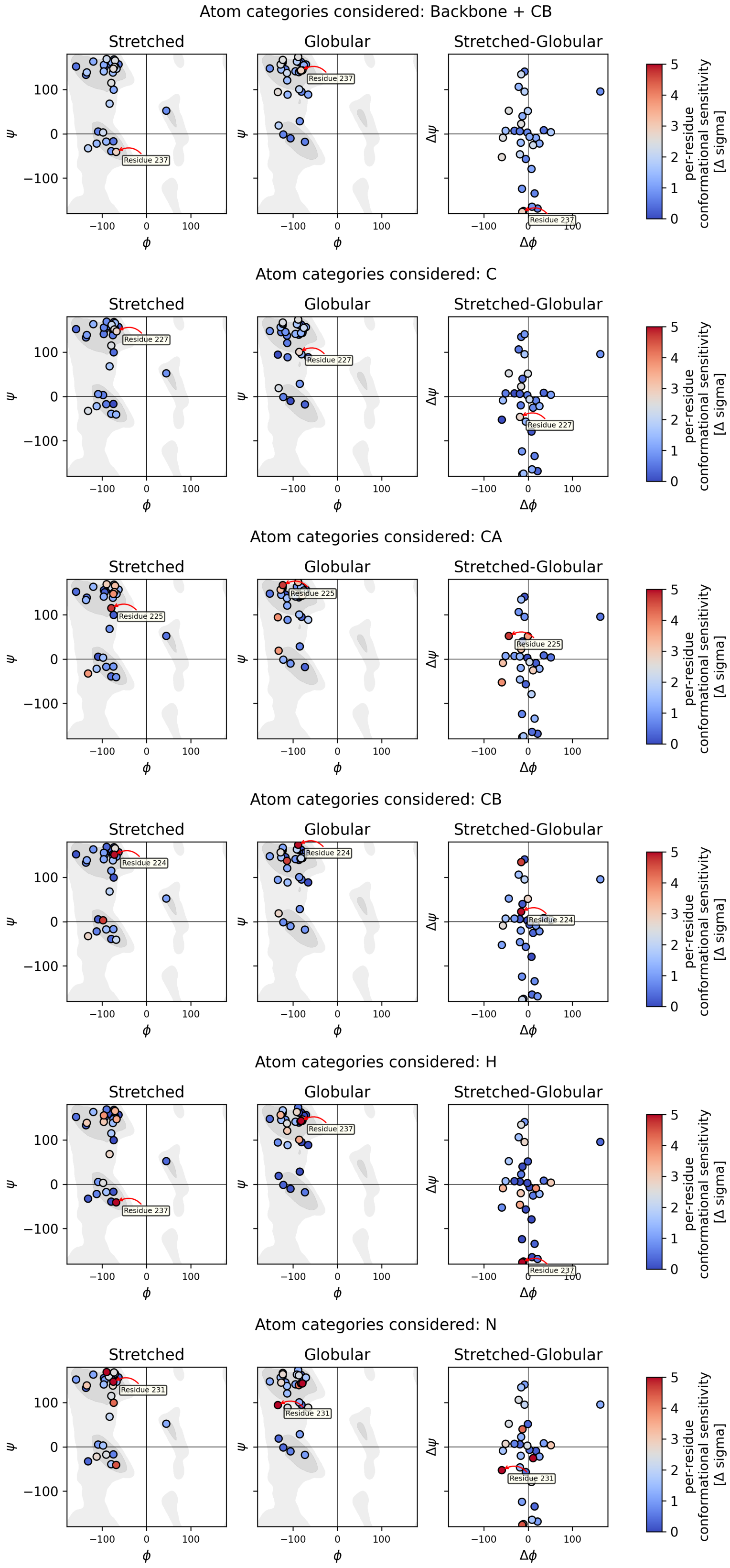

Figure S5.27: Influence of $\Delta\phi$ and $\Delta\psi$ on the conformational sensitivity calculated with the DFT-based QM method using wb97x-d3/6-31G* theory with implicit solvent.

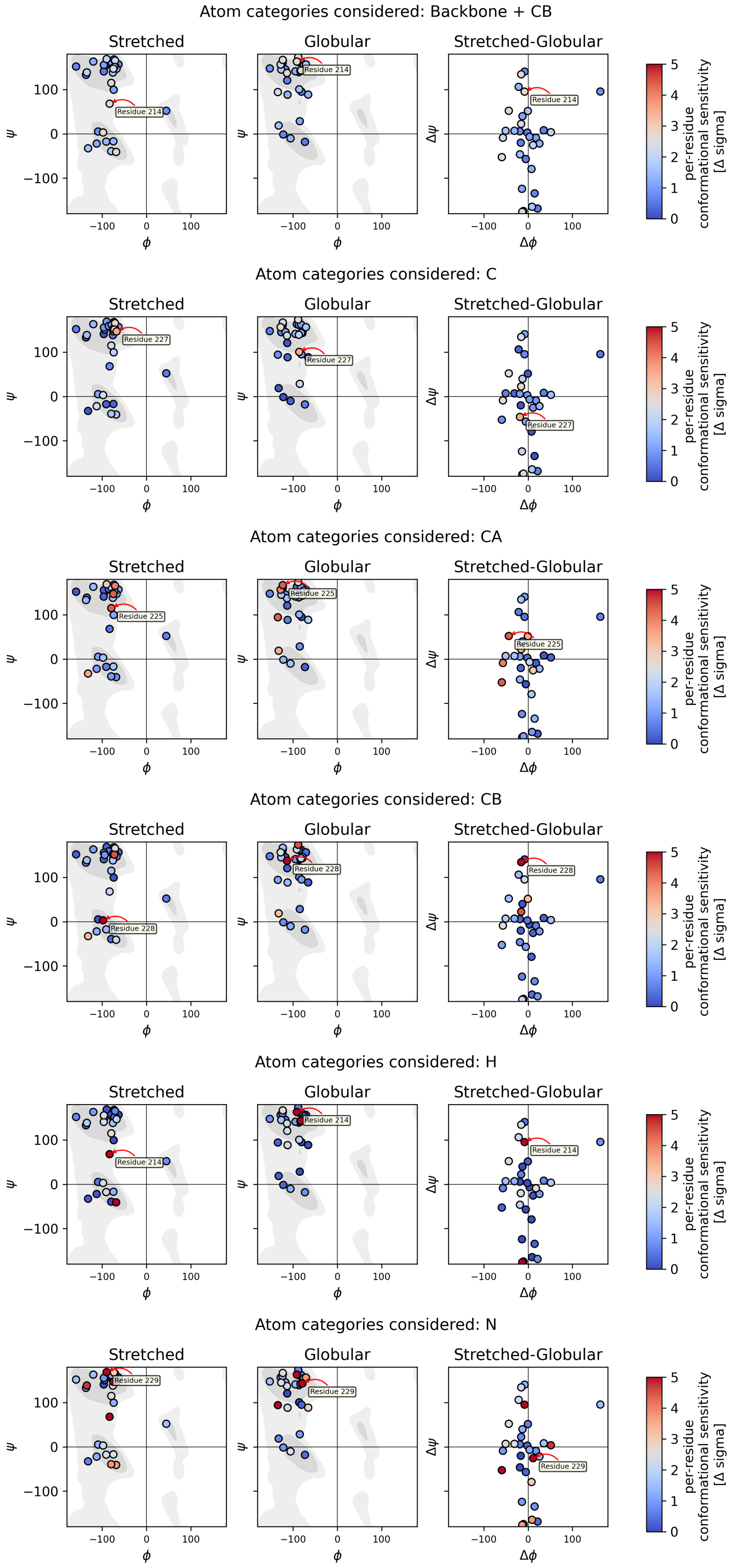

Figure S5.28: Influence of $\Delta\phi$ and $\Delta\psi$ on the conformational sensitivity calculated with the DFT-based QM/MM method using wb97x-d3/6-31G* theory with implicit solvent.

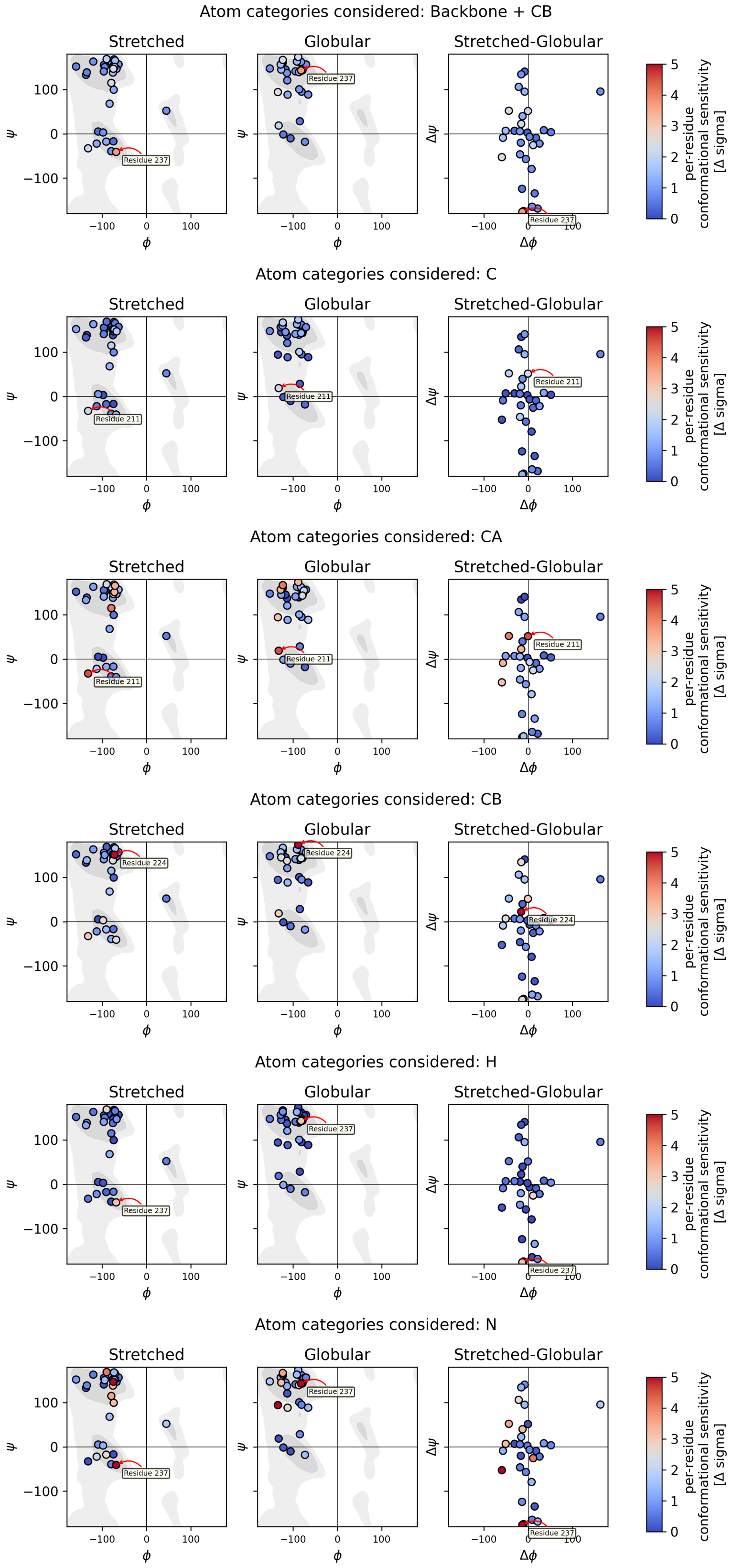

Figure S5.29: Influence of $\Delta\phi$ and $\Delta\psi$ on the conformational sensitivity calculated with the DFT-based QM method using wb97x-d3/cc-pvdz theory with explicit solvent.

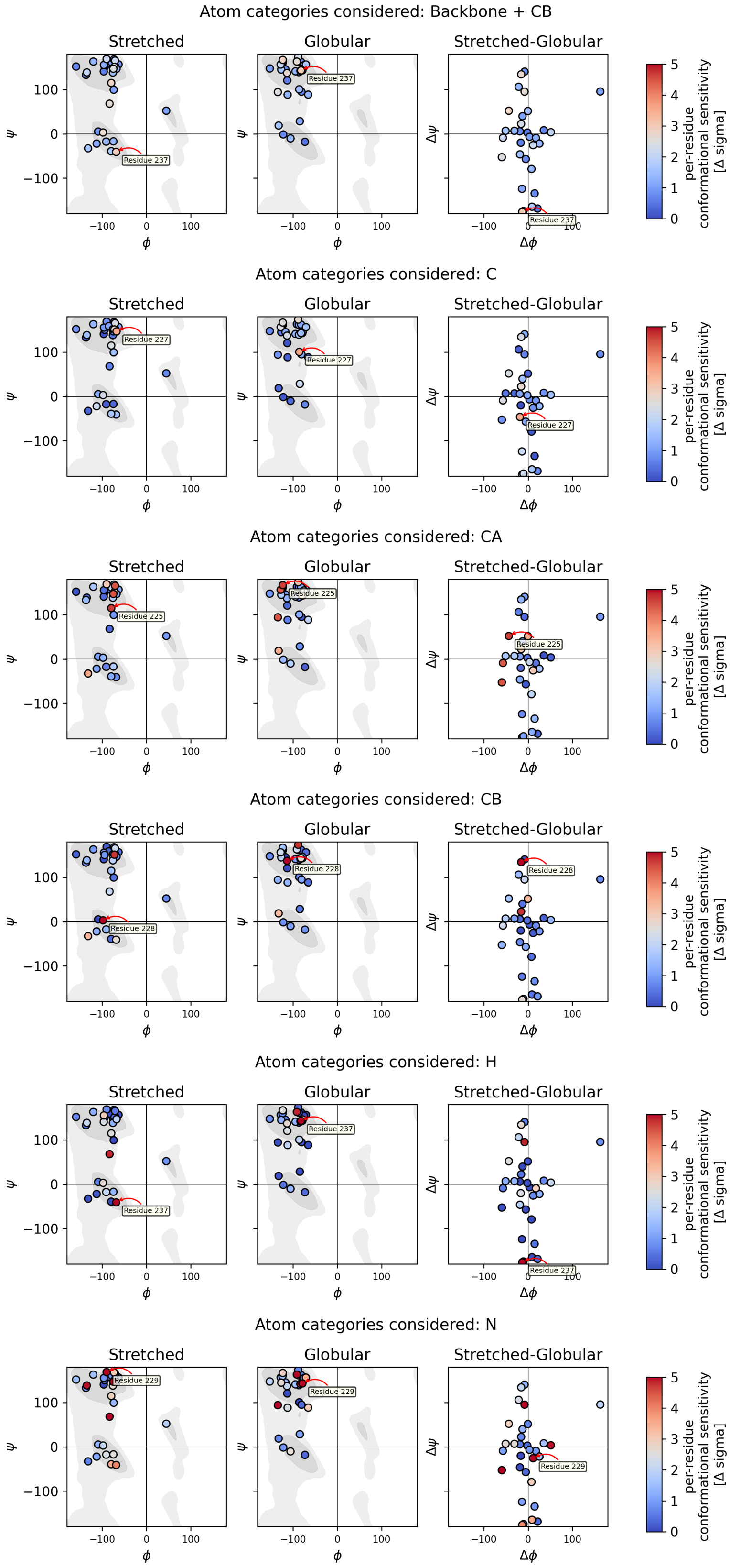

Figure S5.30: Influence of $\Delta\phi$ and $\Delta\psi$ on the conformational sensitivity calculated with the DFT-based QM/MM method using wb97x-d3/cc-pvdz theory with implicit solvent.

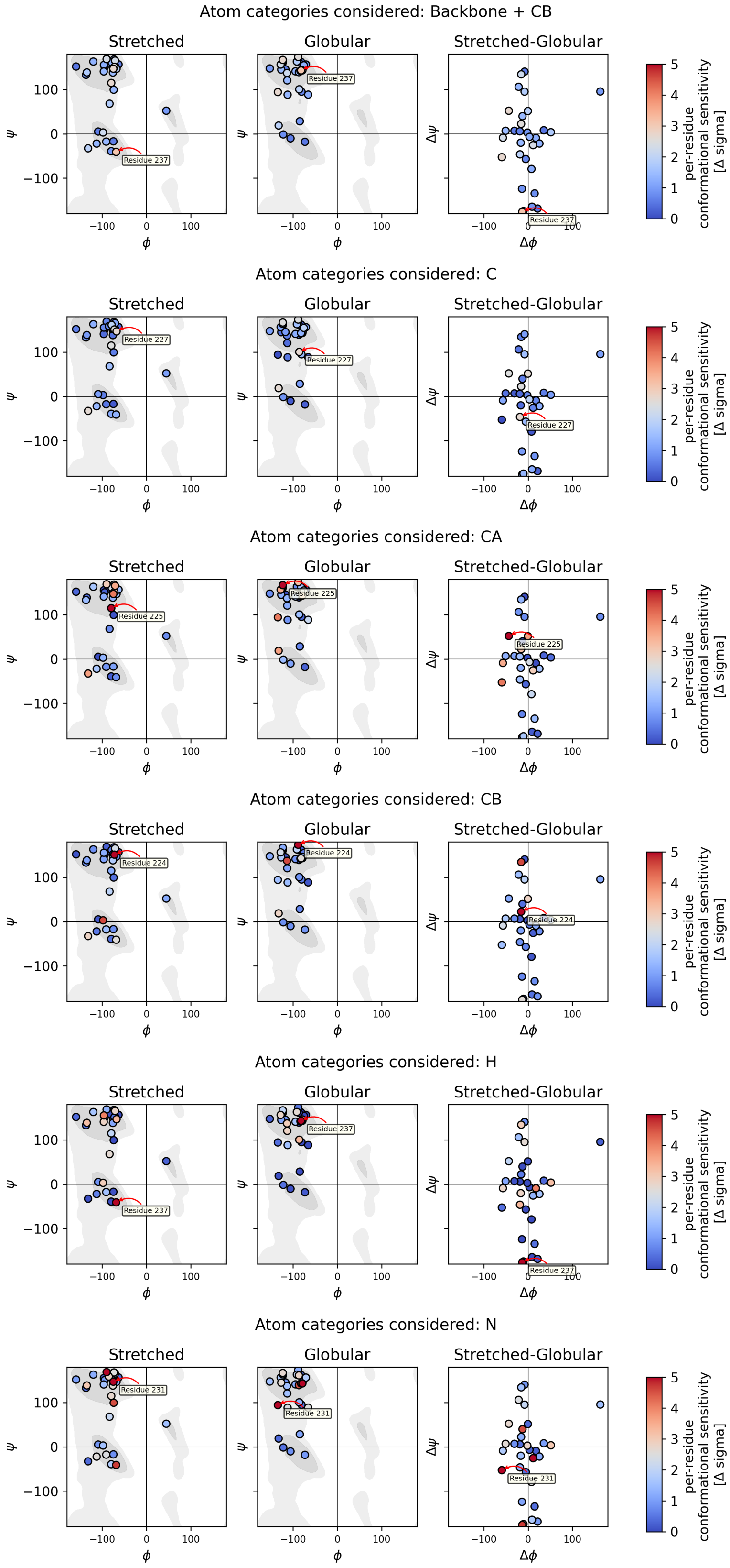

Figure S5.31: Influence of $\Delta\phi$ and $\Delta\psi$ on the conformational sensitivity calculated with the DFT-based QM method using wb97x-d3/cc-pvdz theory with implicit solvent.

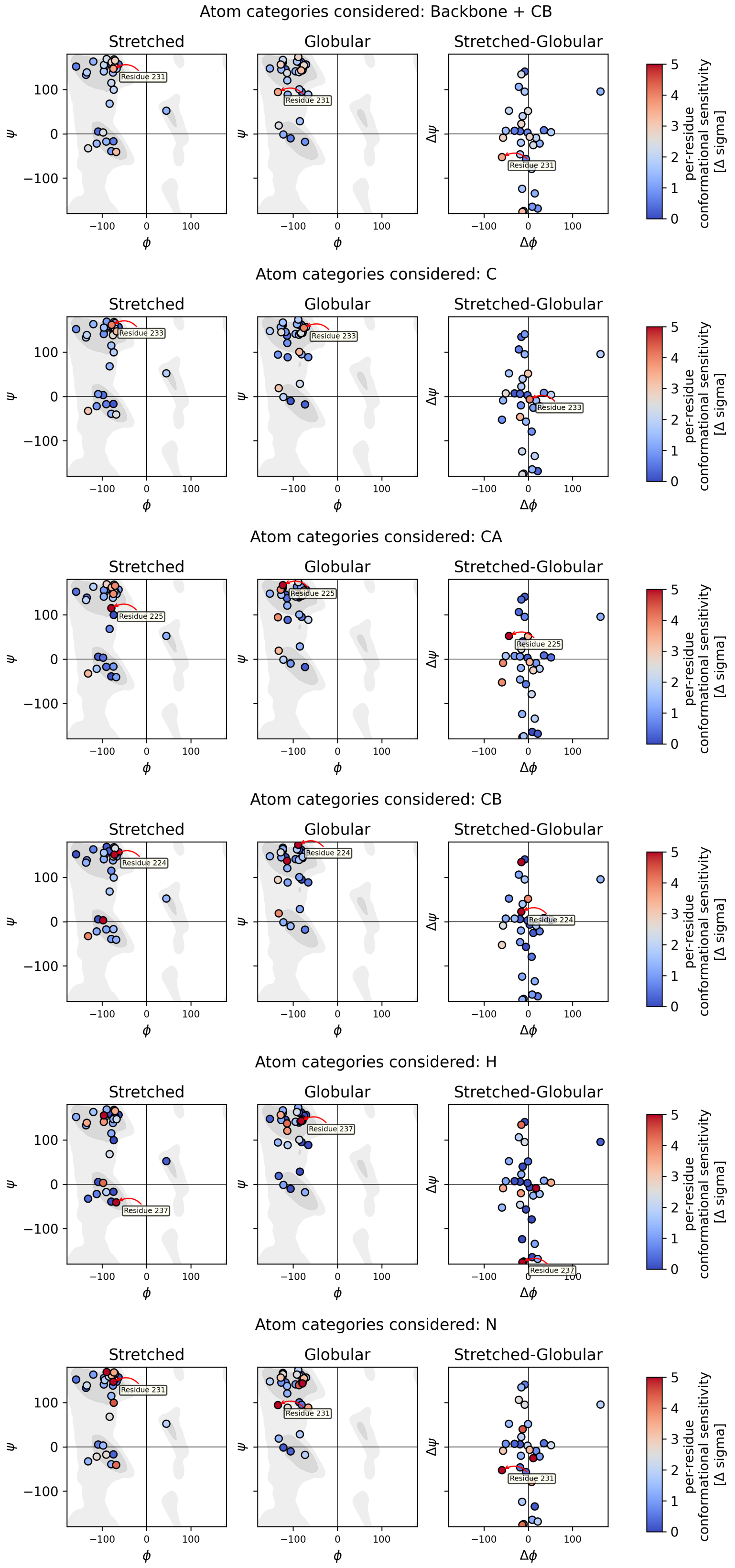

Figure S5.32: Influence of $\Delta\phi$ and $\Delta\psi$ on the conformational sensitivity calculated with the DFT-based QM method using wb97x-d3/cc-pvdz theory in vacuum.

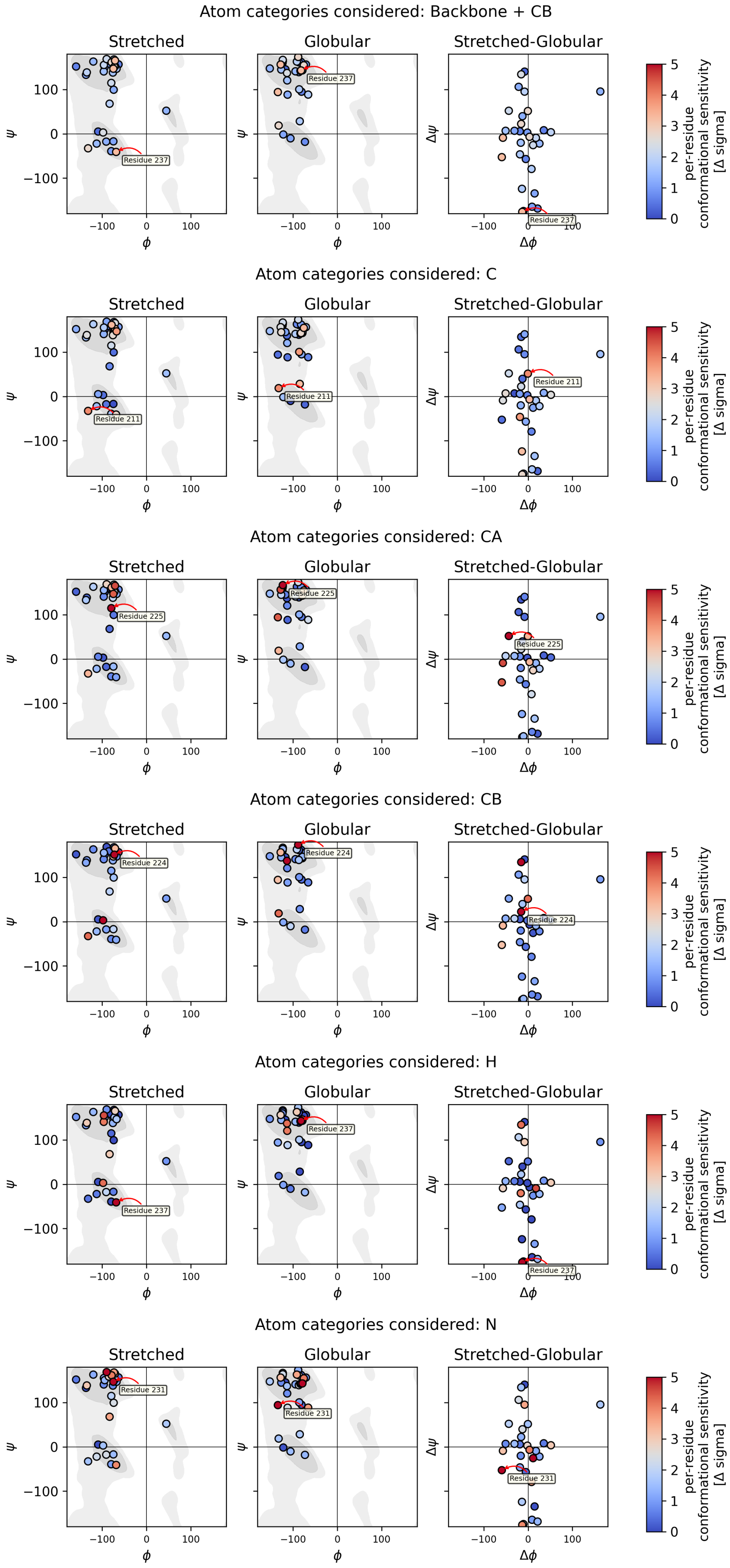

Figure S5.33: Influence of $\Delta\phi$ and $\Delta\psi$ on the conformational sensitivity calculated with the DFT-based QM/MM method using wb97x-d3/cc-pvdz theory in vacuum.

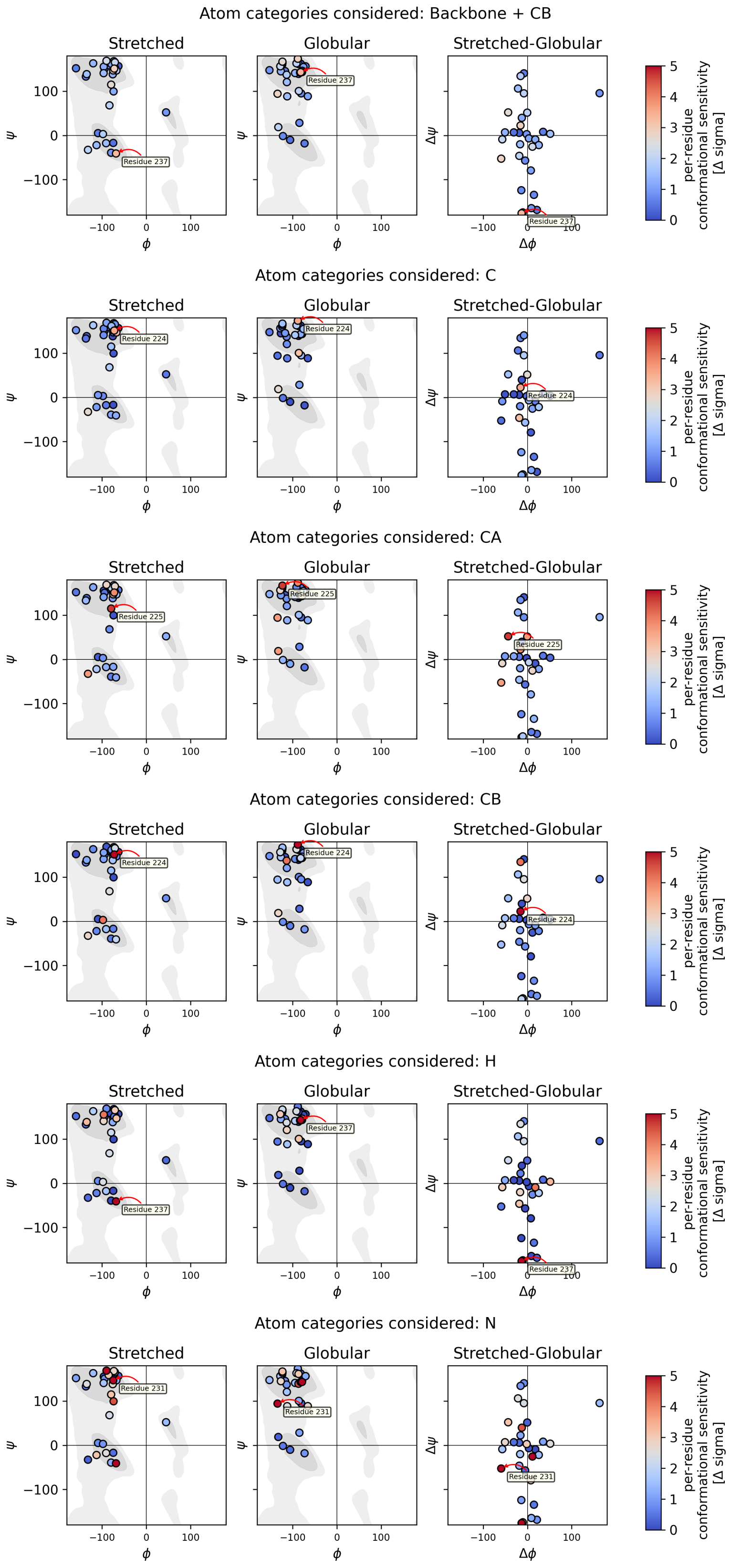

Figure S5.34: Influence of $\Delta\phi$ and $\Delta\psi$ on the conformational sensitivity calculated with the DFT-based QM method using wb97x-d3/pcSseg-1 theory with implicit solvent.

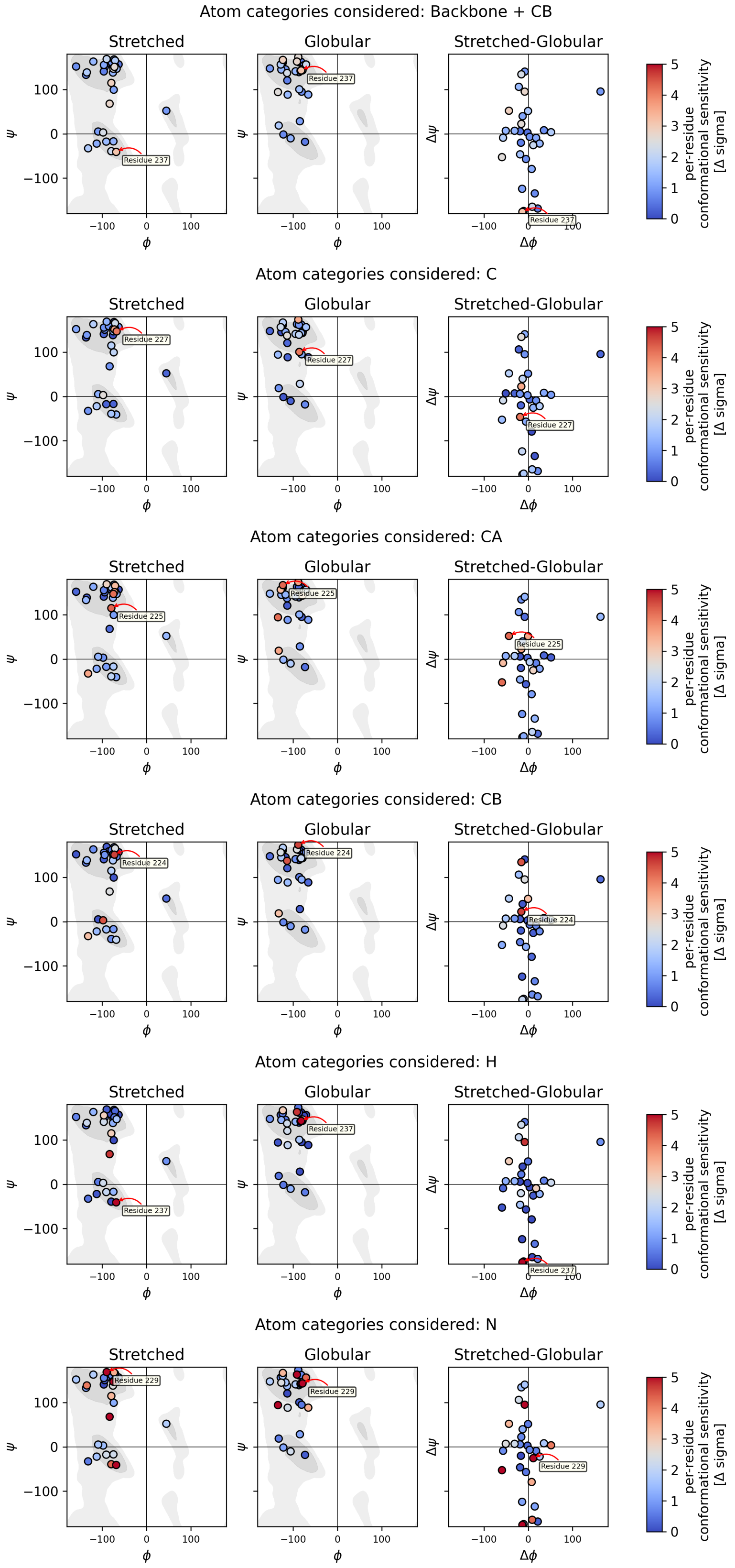

Figure S5.35: Influence of $\Delta\phi$ and $\Delta\psi$ on the conformational sensitivity calculated with the DFT-based QM/MM method using wb97x-d3/pcSseg-1 theory with implicit solvent.

1.6 Feature Importance using Ordinal Encoding

The following figures show the feature importances using ordinal encoding.

A table of content to find each graphic can be found in table 3.

Table 3: Table of content for the ordinal encoded feature importances of the methods.

Name	Type	Solvation	Figure	Page
PPM	empirical	vacuum	S6.1	80
UCBShiftX	empirical	vacuum	S6.2	80
shiftX2	empirical	vacuum	S6.3	81
sparta+	empirical	vacuum	S6.4	81
b3lyp/6-31G*	QM/MM	implicit	S6.6	82
b3lyp/6-31G*	QM	implicit	S6.6	82
b3lyp/cc-pvdz	QM/MM	vacuum	S6.10	84
b3lyp/cc-pvdz	QM	explicit	S6.8	83
b3lyp/cc-pvdz	QM/MM	implicit	S6.11	85
b3lyp/cc-pvdz	QM	vacuum	S6.10	84
b3lyp/cc-pvdz	QM	implicit	S6.11	85
b3lyp/pcSseg-1	QM	implicit	S6.13	86
b3lyp/pcSseg-1	QM/MM	implicit	S6.13	86
becke97-2/6-31G*	QM/MM	implicit	S6.15	87
becke97-2/6-31G*	QM	implicit	S6.15	87
becke97-2/cc-pvdz	QM	implicit	S6.17	88
becke97-2/cc-pvdz	QM/MM	implicit	S6.17	88
becke97-2/pcSseg-1	QM	implicit	S6.19	89
becke97-2/pcSseg-1	QM/MM	implicit	S6.19	89
becke97-d/6-31G*	QM	implicit	S6.21	90
becke97-d/6-31G*	QM/MM	implicit	S6.21	90
becke97-d/cc-pvdz	QM/MM	implicit	S6.23	91
becke97-d/cc-pvdz	QM	implicit	S6.23	91
becke97-d/pcSseg-1	QM/MM	implicit	S6.26	92
becke97-d/pcSseg-1	QM	explicit	S6.25	92
becke97-d/pcSseg-1	QM	implicit	S6.26	92
wb97x-d3/6-31G*	QM	implicit	S6.28	93
wb97x-d3/6-31G*	QM/MM	implicit	S6.28	93
wb97x-d3/cc-pvdz	QM	explicit	S6.29	94
wb97x-d3/cc-pvdz	QM/MM	implicit	S6.31	95
wb97x-d3/cc-pvdz	QM	implicit	S6.31	95
wb97x-d3/cc-pvdz	QM	vacuum	S6.33	96
wb97x-d3/cc-pvdz	QM/MM	vacuum	S6.33	96
wb97x-d3/pcSseg-1	QM	implicit	S6.35	97
wb97x-d3/pcSseg-1	QM/MM	implicit	S6.35	97

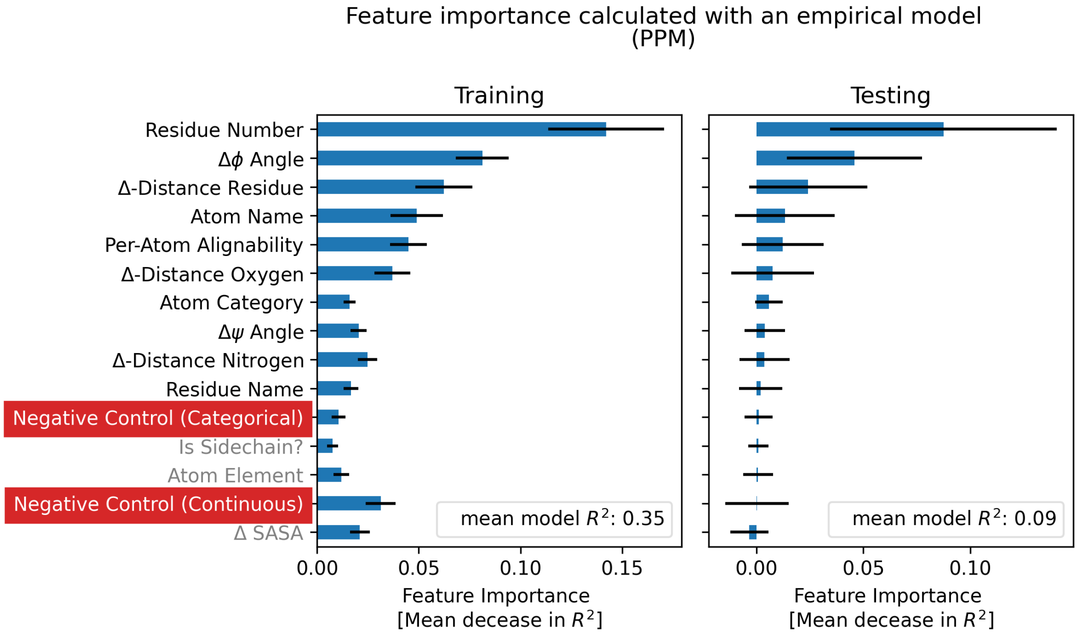

Figure S6.1: Feature importances (ordinal) calculated with the empirical method PPM.

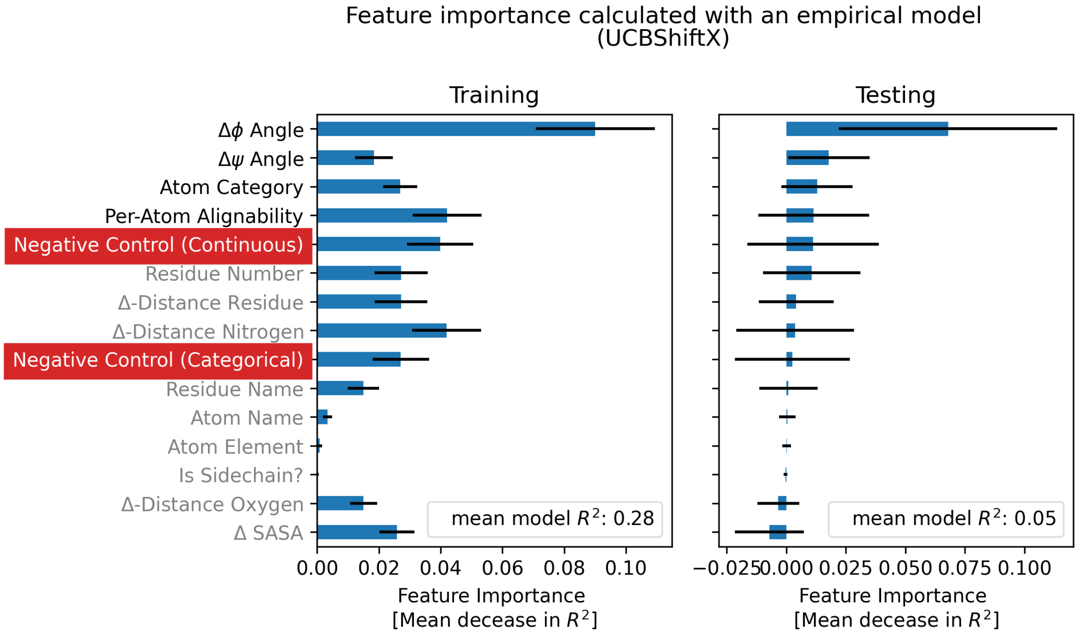

Figure S6.2: Feature importances (ordinal) calculated with the empirical method UCBShiftX.

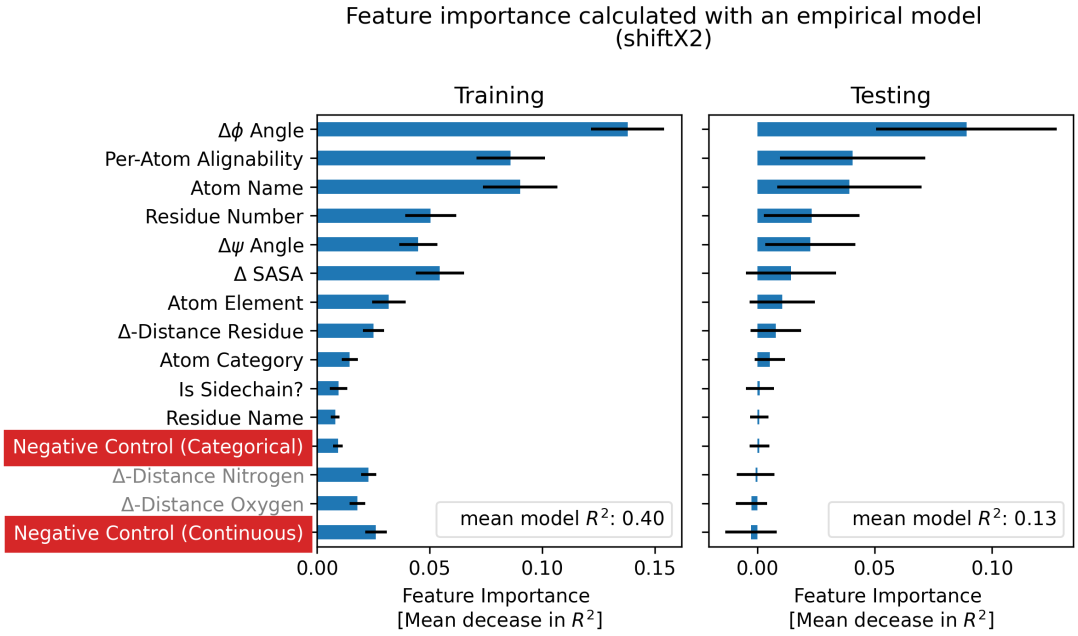

Figure S6.3: Feature importances (ordinal) calculated with the empirical method shiftX2.

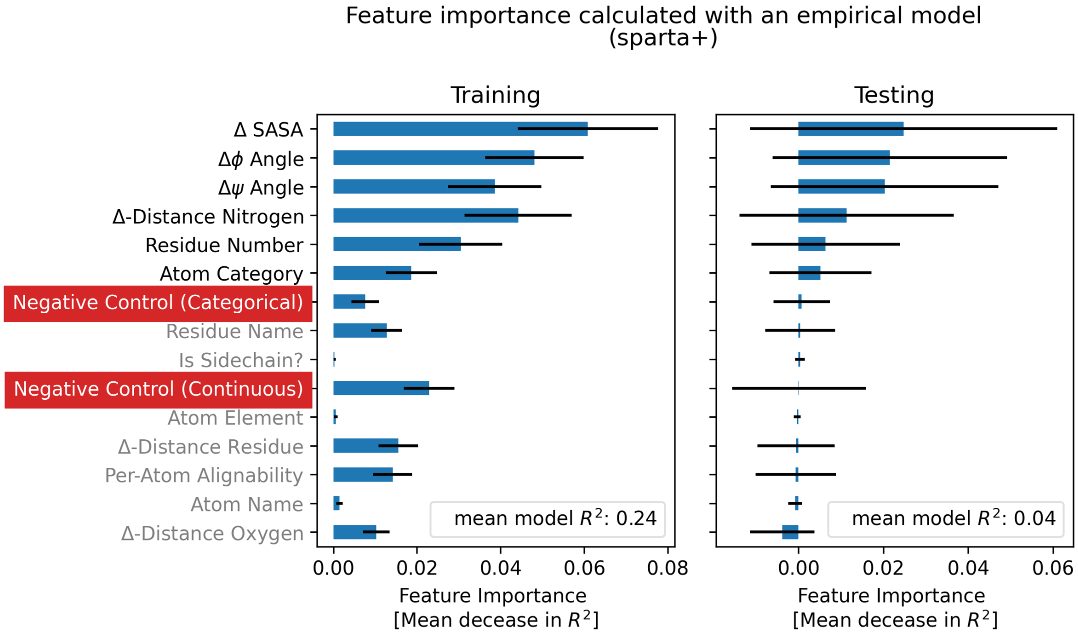

Figure S6.4: Feature importances (ordinal) calculated with the empirical method sparta+.

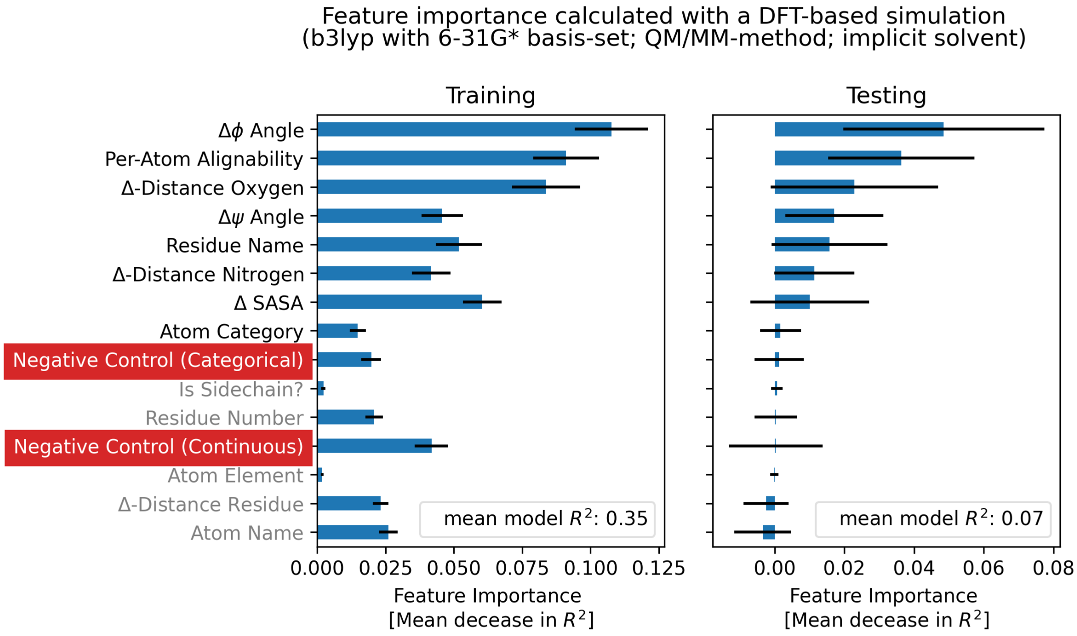

Figure S6.5: Feature importances (ordinal) calculated with the DFT-based QM/MM method using b3lyp/6-31G* theory with implicit solvent.

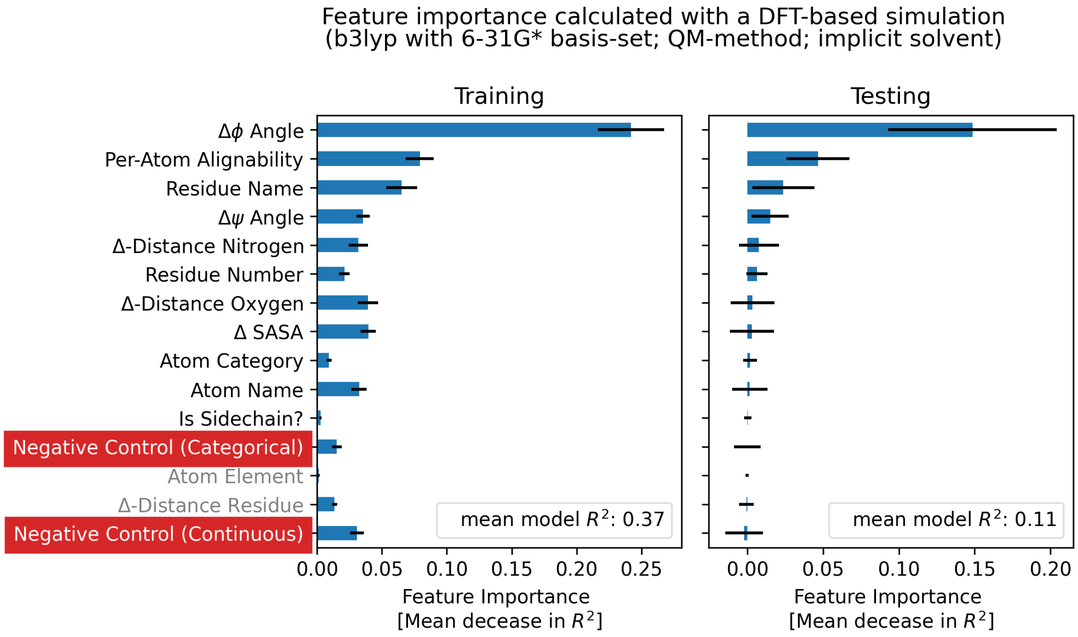

Figure S6.6: Feature importances (ordinal) calculated with the DFT-based QM method using b3lyp/6-31G* theory with implicit solvent.

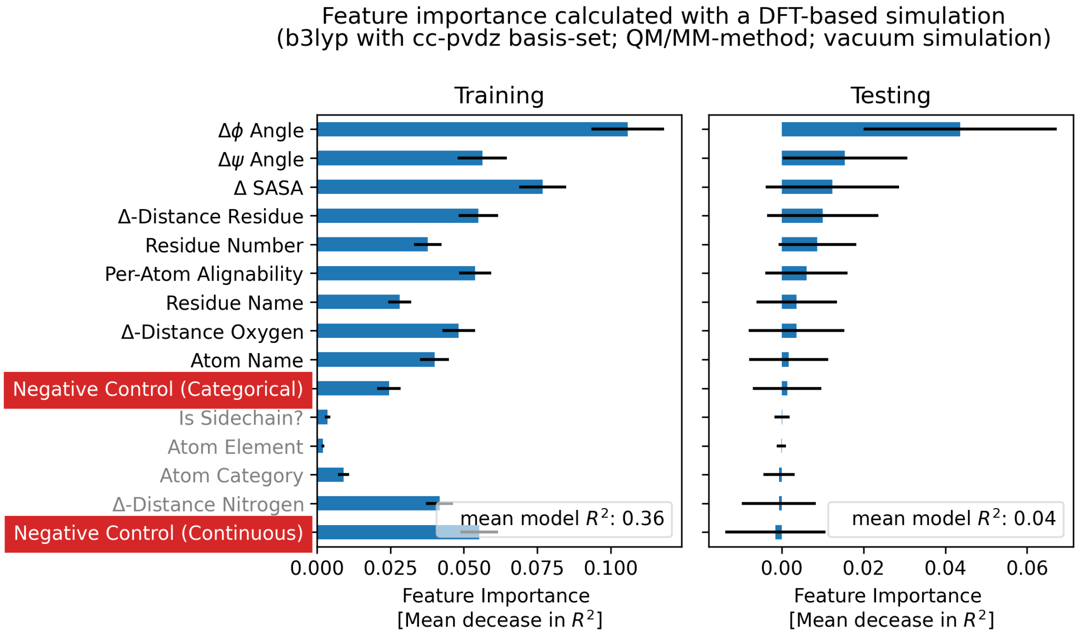

Figure S6.7: Feature importances (ordinal) calculated with the DFT-based QM/MM method using b3lyp/cc-pvdz theory in vacuum.

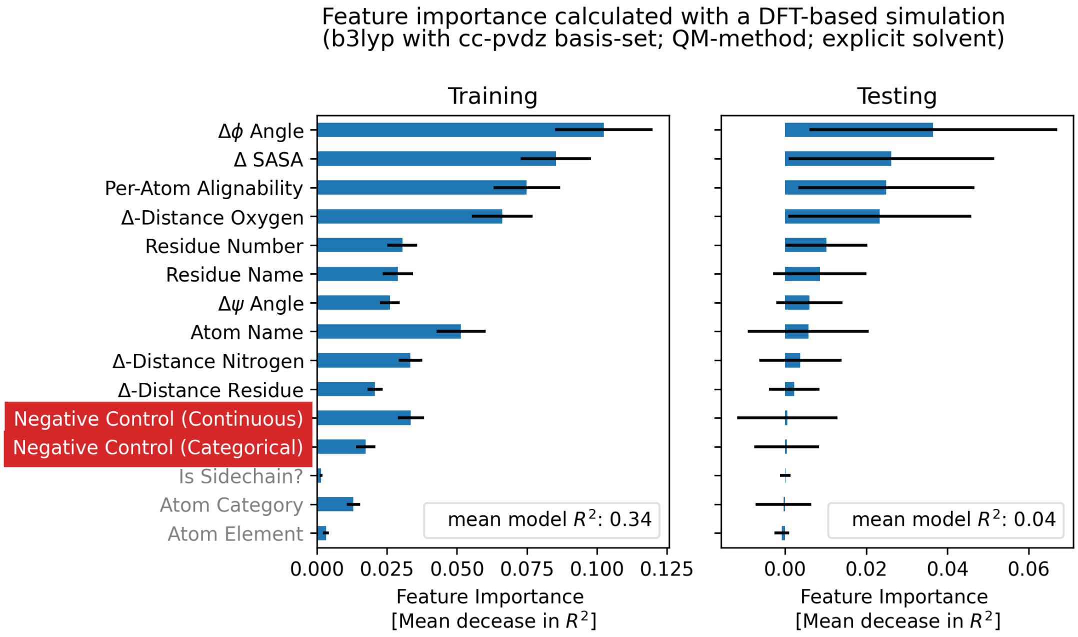

Figure S6.8: Feature importances (ordinal) calculated with the DFT-based QM method using b3lyp/cc-pvdz theory with explicit solvent.

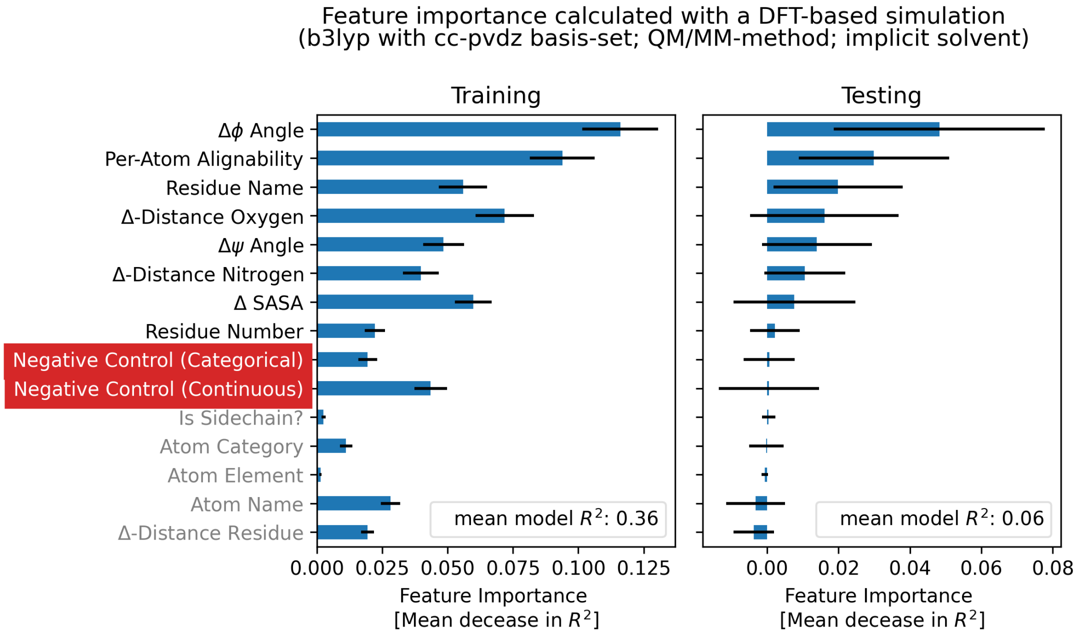

Figure S6.9: Feature importances (ordinal) calculated with the DFT-based QM/MM method using b3lyp/cc-pvdz theory with implicit solvent.

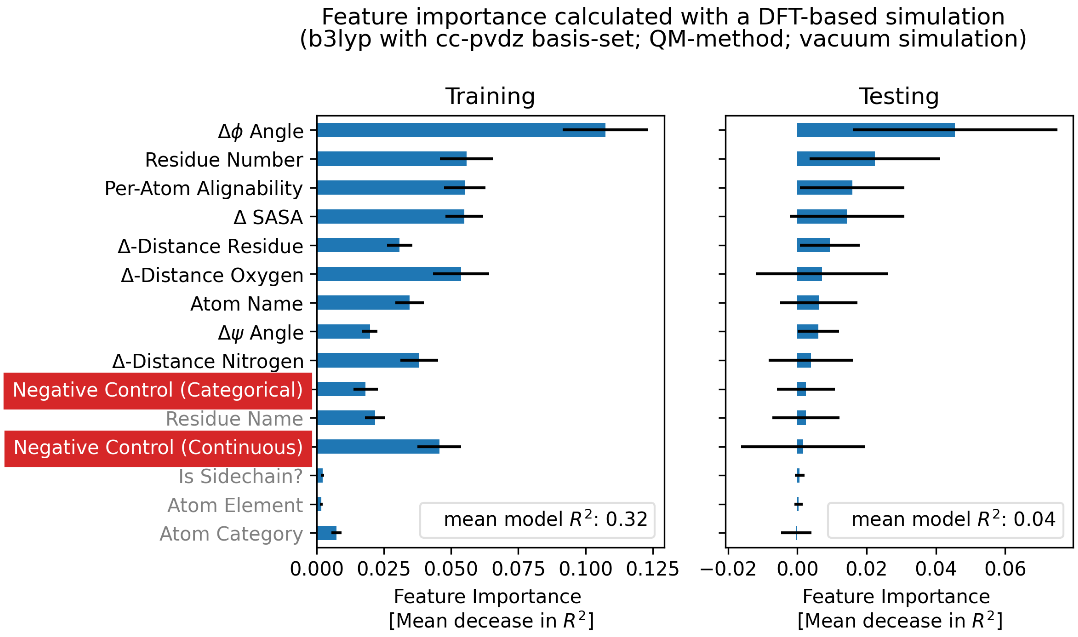

Figure S6.10: Feature importances (ordinal) calculated with the DFT-based QM method using b3lyp/cc-pvdz theory in vacuum.

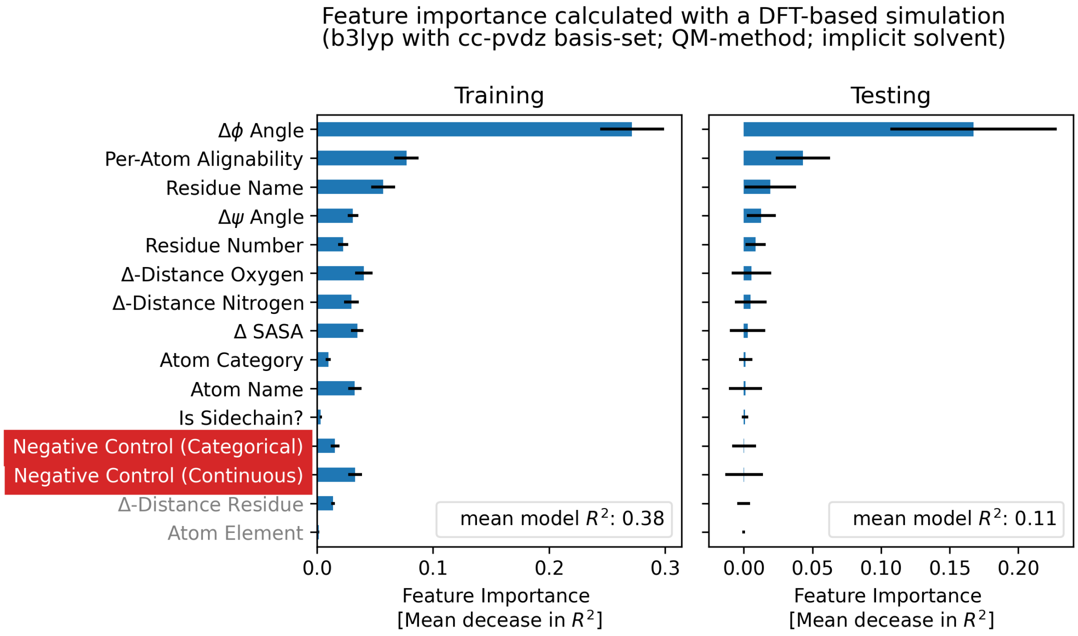

Figure S6.11: Feature importances (ordinal) calculated with the DFT-based QM method using b3lyp/cc-pvdz theory with implicit solvent.

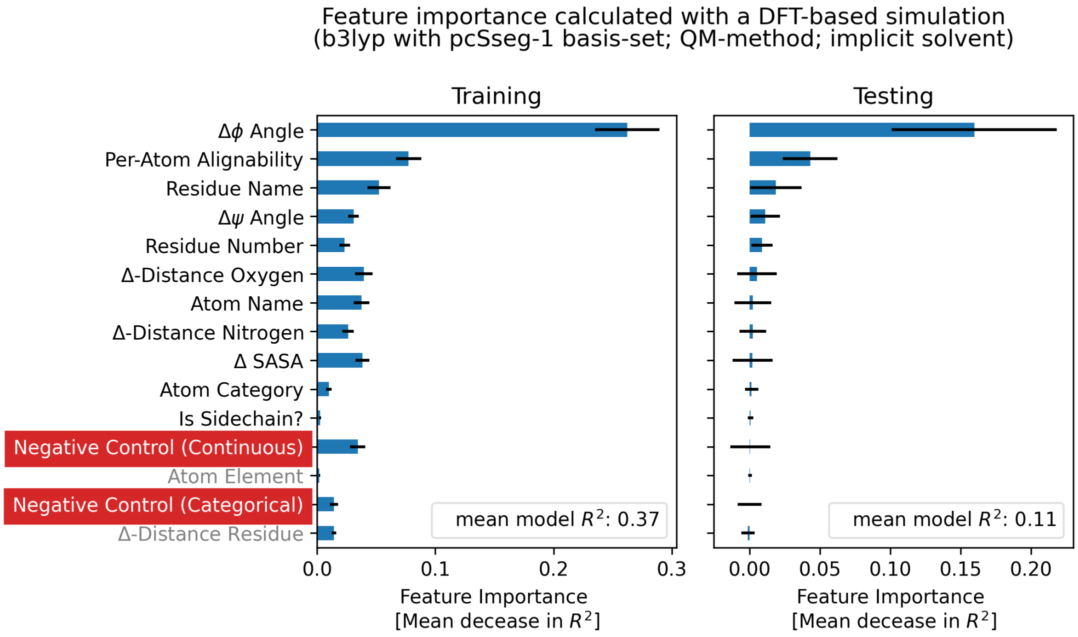

Figure S6.12: Feature importances (ordinal) calculated with the DFT-based QM method using b3lyp/pcSseg-1 theory with implicit solvent.

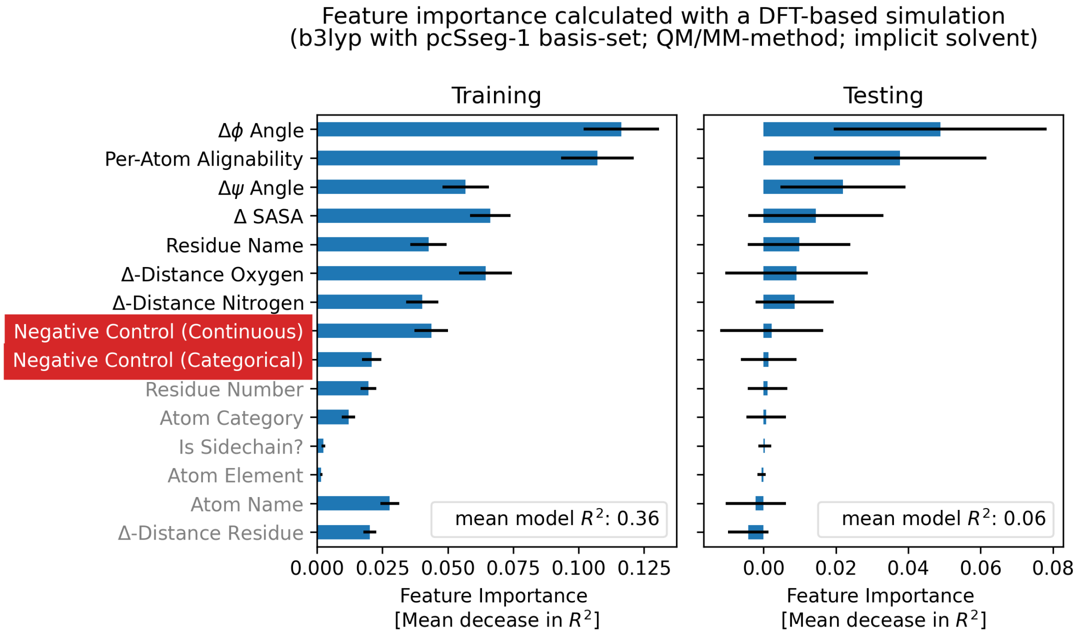

Figure S6.13: Feature importances (ordinal) calculated with the DFT-based QM/MM method using b3lyp/pcSseg-1 theory with implicit solvent.

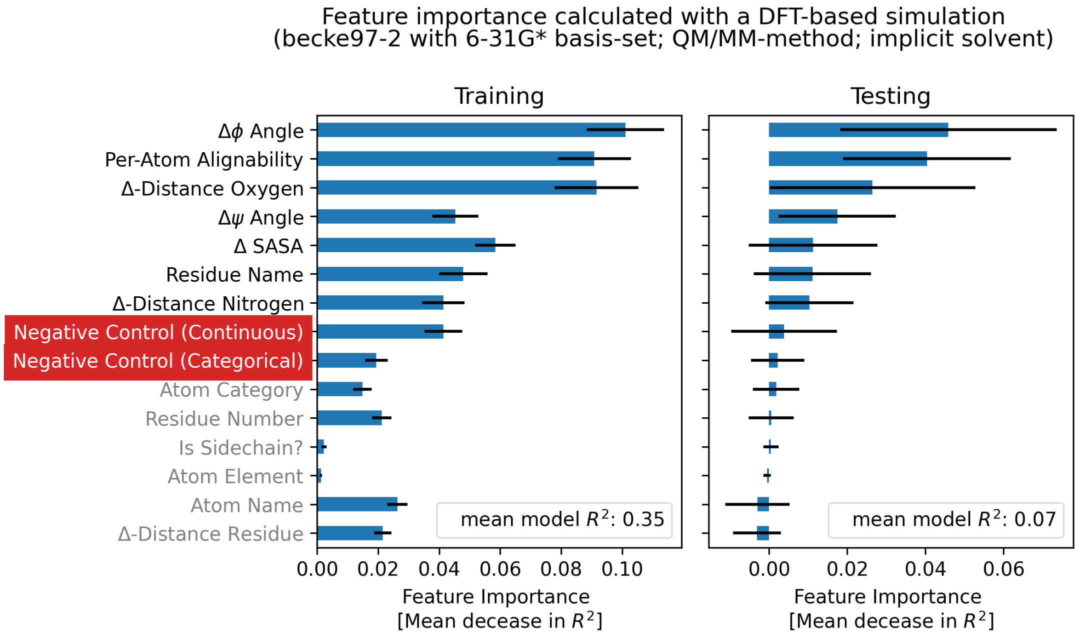

Figure S6.14: Feature importances (ordinal) calculated with the DFT-based QM/MM method using becke97-2/6-31G* theory with implicit solvent.

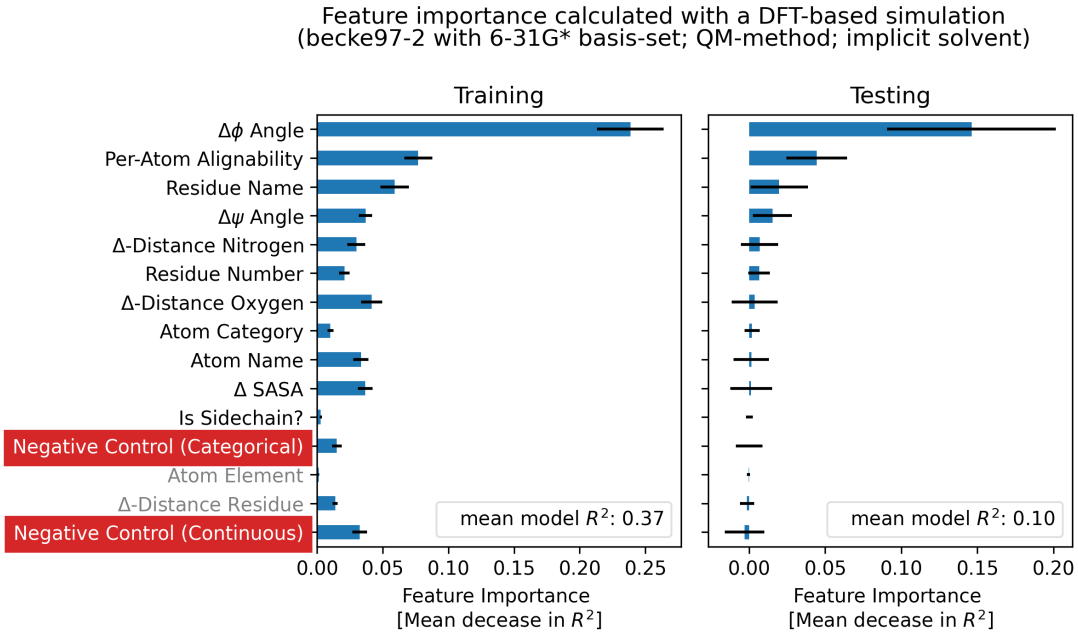

Figure S6.15: Feature importances (ordinal) calculated with the DFT-based QM method using becke97-2/6-31G* theory with implicit solvent.

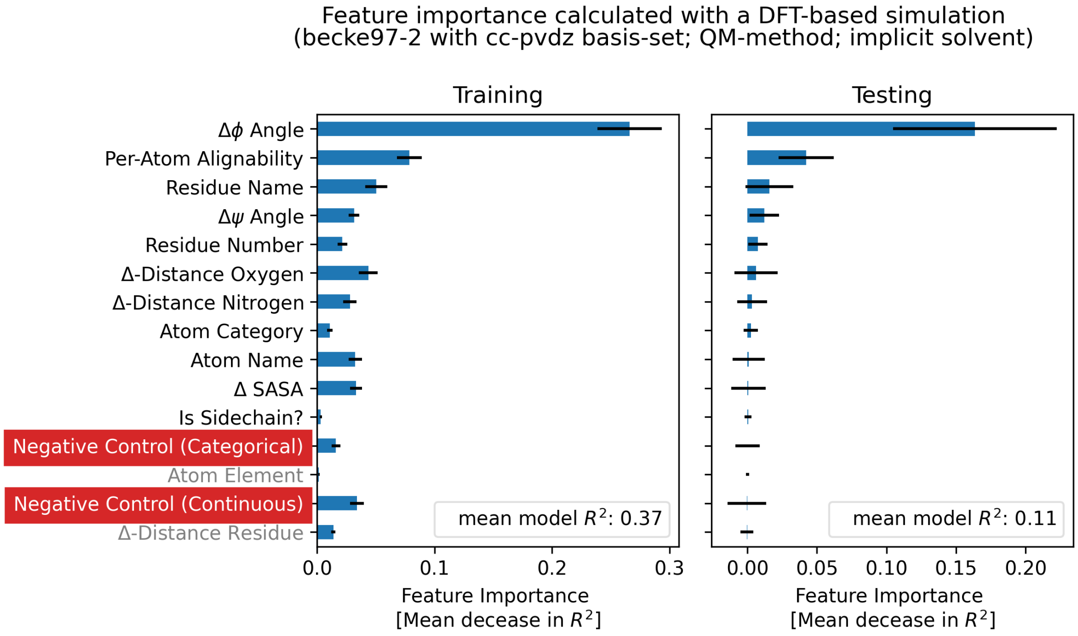

Figure S6.16: Feature importances (ordinal) calculated with the DFT-based QM method using becke97-2/cc-pvdz theory with implicit solvent.

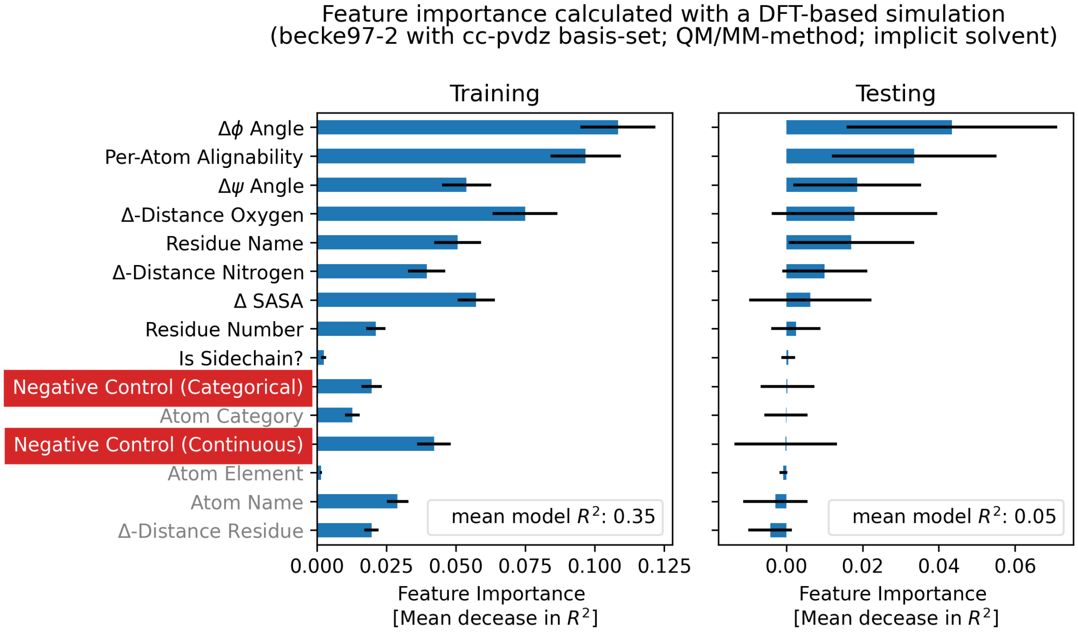

Figure S6.17: Feature importances (ordinal) calculated with the DFT-based QM/MM method using becke97-2/cc-pvdz theory with implicit solvent.

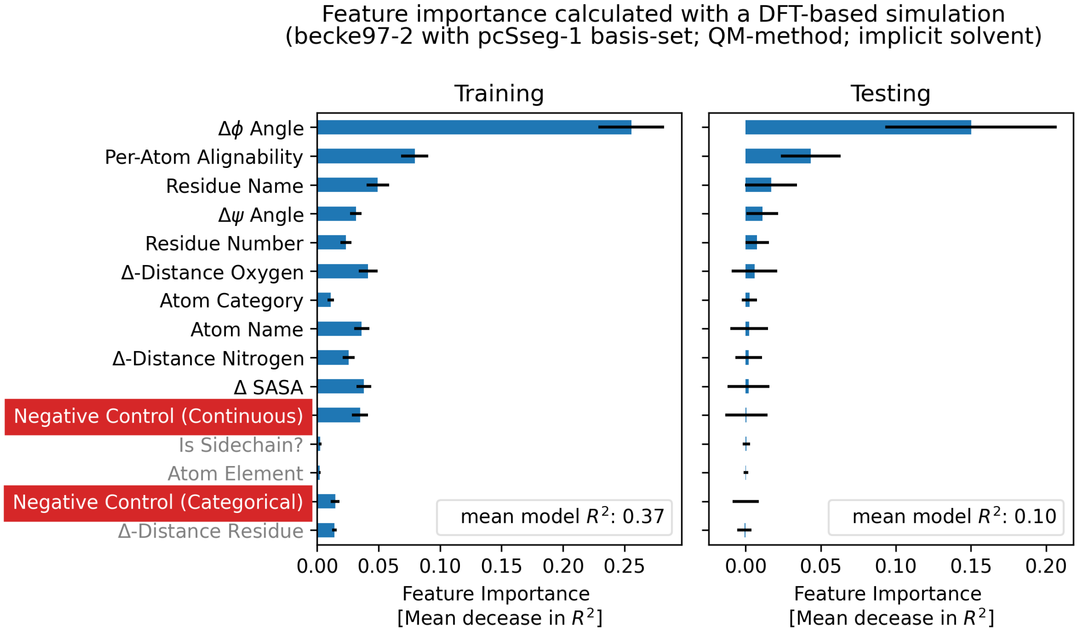

Figure S6.18: Feature importances (ordinal) calculated with the DFT-based QM method using becke97-2/pcSseg-1 theory with implicit solvent.

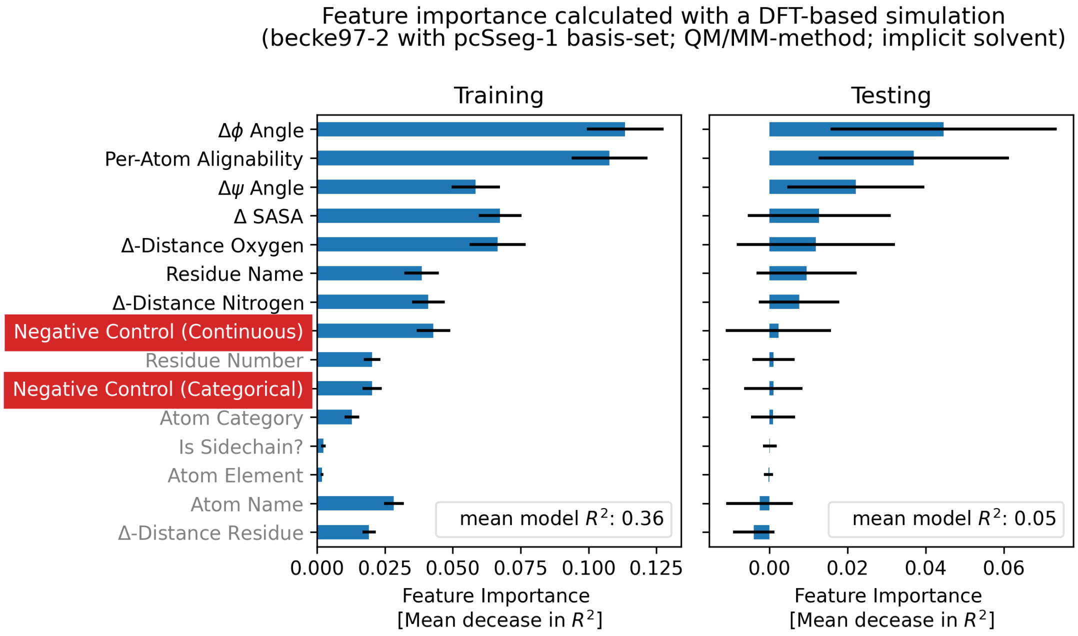

Figure S6.19: Feature importances (ordinal) calculated with the DFT-based QM/MM method using becke97-2/pcSseg-1 theory with implicit solvent.

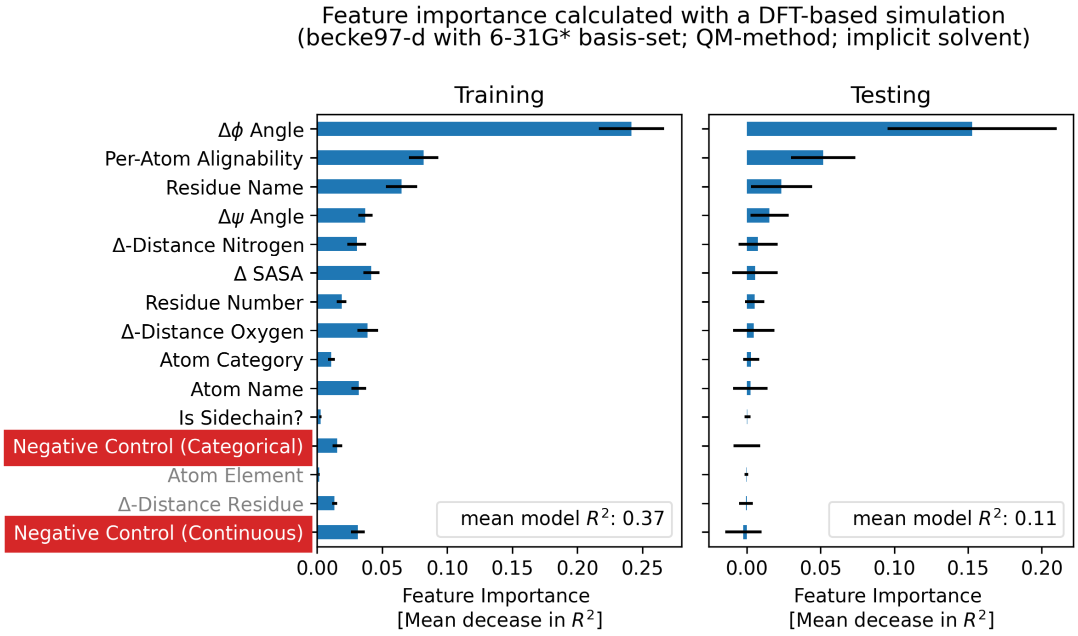

Figure S6.20: Feature importances (ordinal) calculated with the DFT-based QM method using becke97-d/6-31G* theory with implicit solvent.

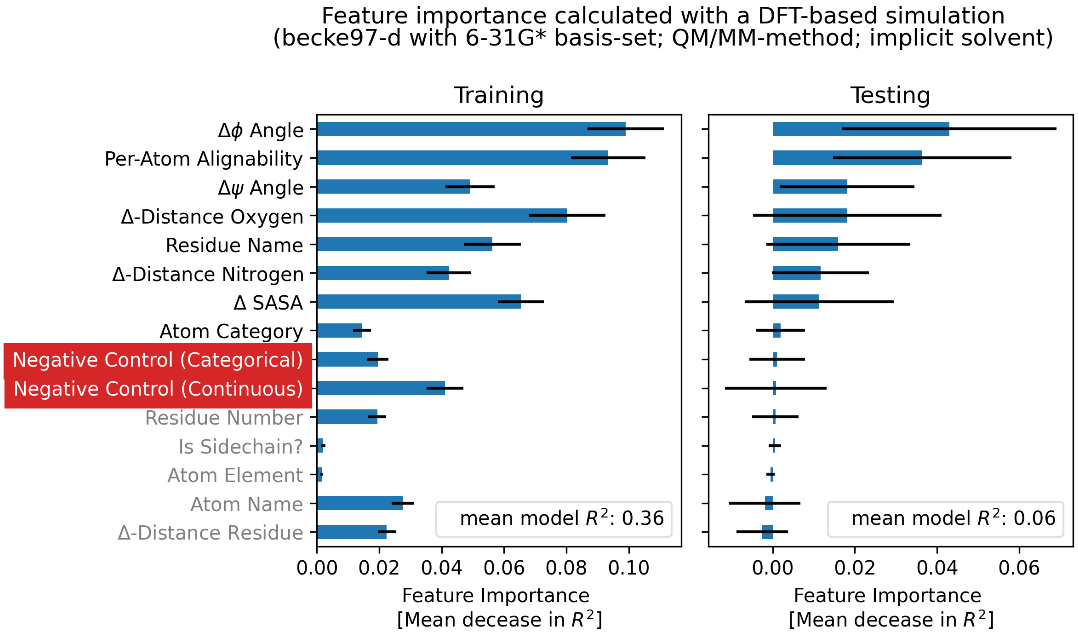

Figure S6.21: Feature importances (ordinal) calculated with the DFT-based QM/MM method using becke97-d/6-31G* theory with implicit solvent.

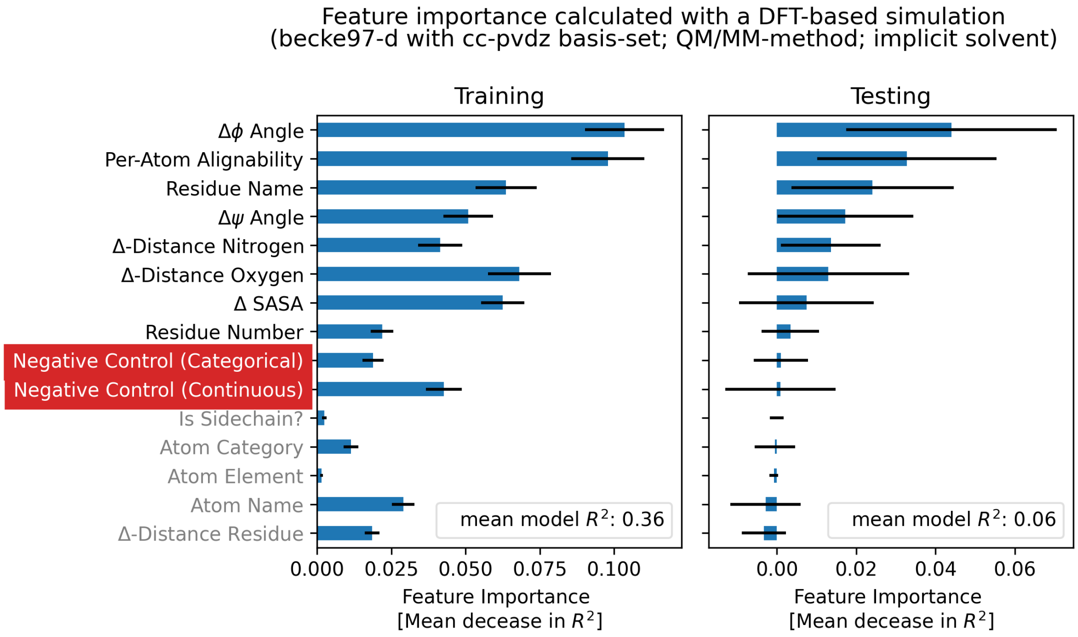

Figure S6.22: Feature importances (ordinal) calculated with the DFT-based QM/MM method using becke97-d/cc-pvdz theory with implicit solvent.

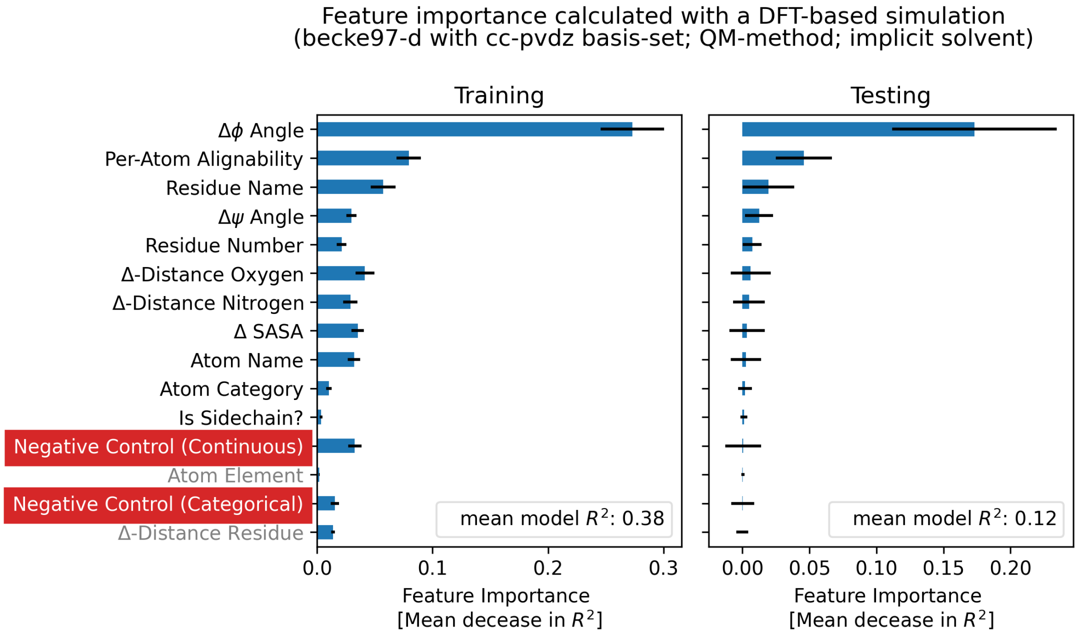

Figure S6.23: Feature importances (ordinal) calculated with the DFT-based QM method using becke97-d/cc-pvdz theory with implicit solvent.

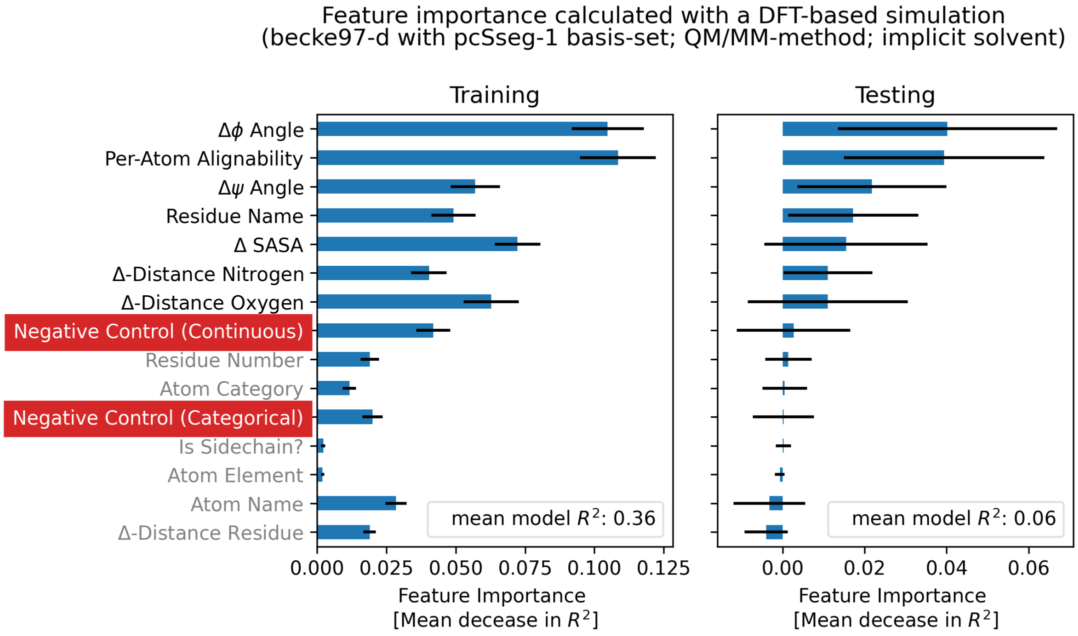

Figure S6.24: Feature importances (ordinal) calculated with the DFT-based QM/MM method using becke97-d/pcSseg-1 theory with implicit solvent.

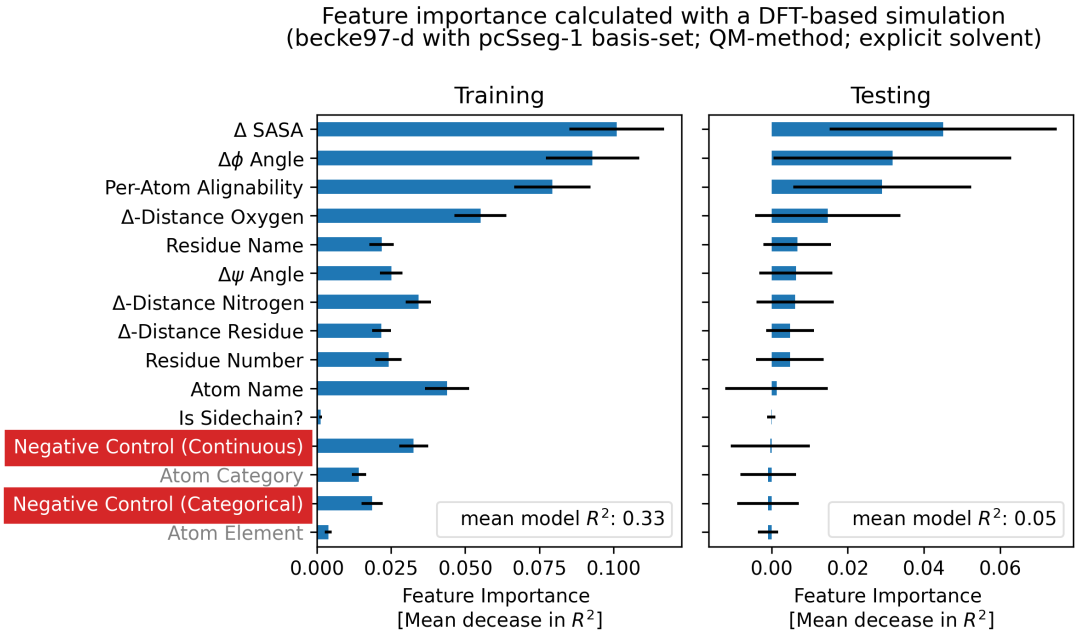

Figure S6.25: Feature importances (ordinal) calculated with the DFT-based QM method using becke97-d/pcSseg-1 theory with explicit solvent.

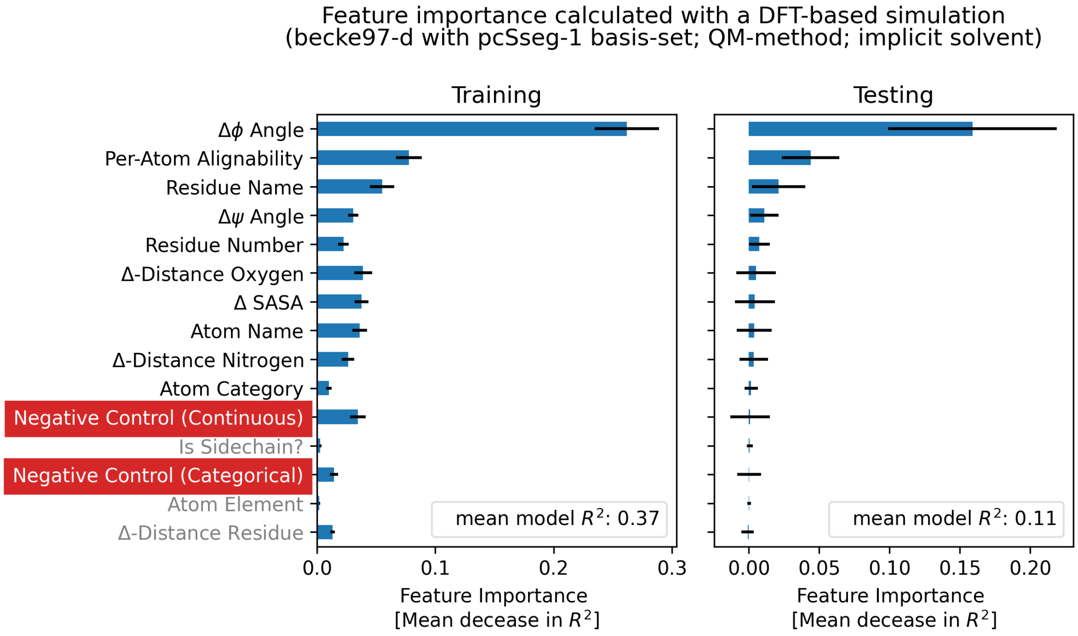

Figure S6.26: Feature importances (ordinal) calculated with the DFT-based QM method using becke97-d/pcSseg-1 theory with implicit solvent.

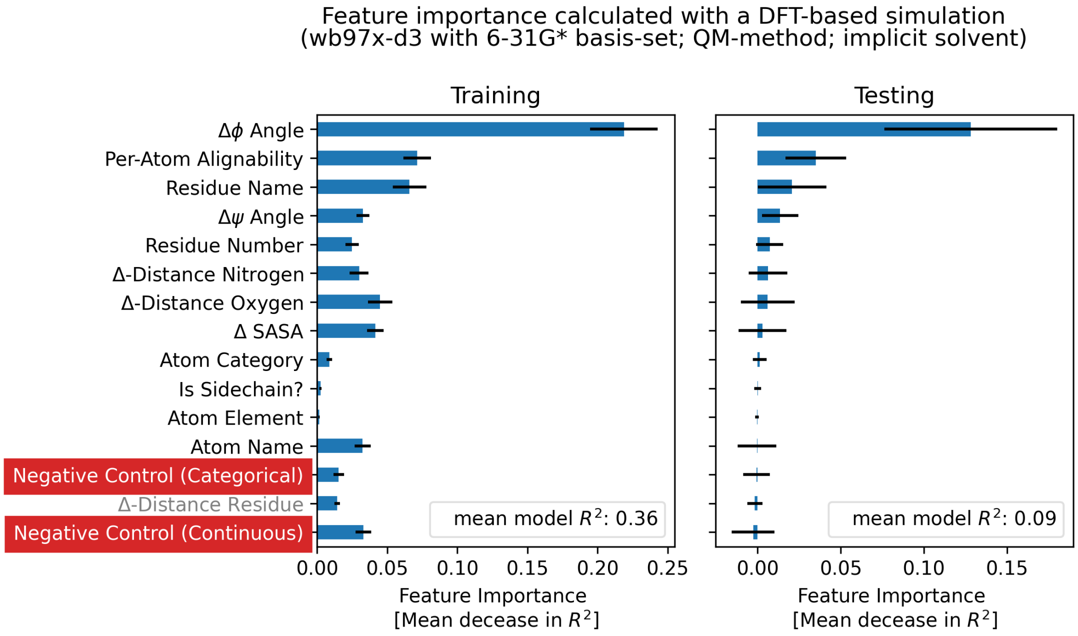

Figure S6.27: Feature importances (ordinal) calculated with the DFT-based QM method using wb97x-d3/6-31G* theory with implicit solvent.

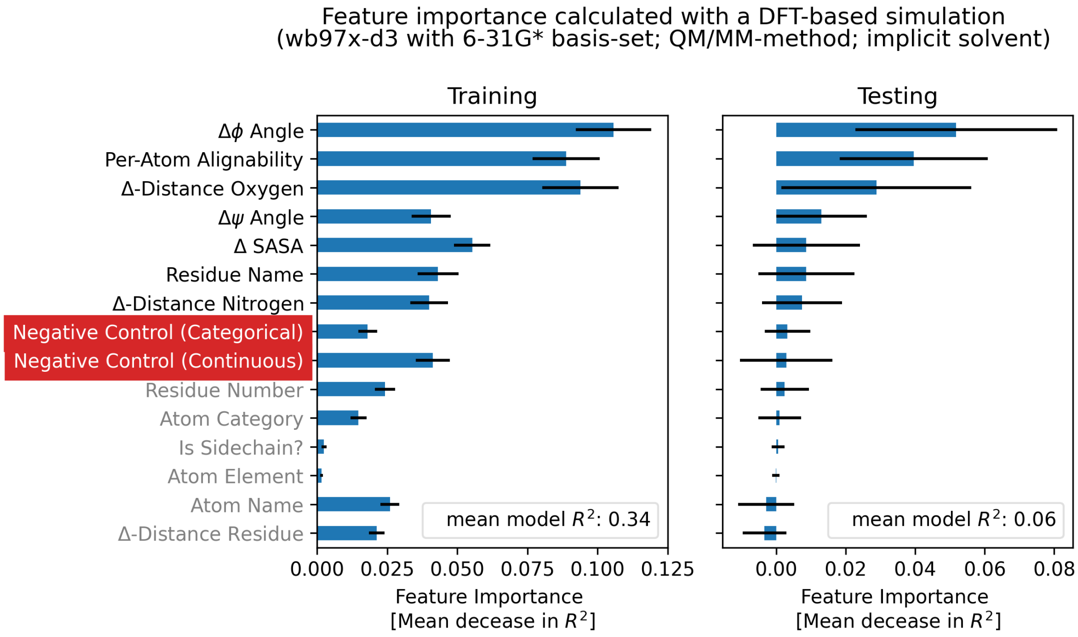

Figure S6.28: Feature importances (ordinal) calculated with the DFT-based QM/MM method using wb97x-d3/6-31G* theory with implicit solvent.

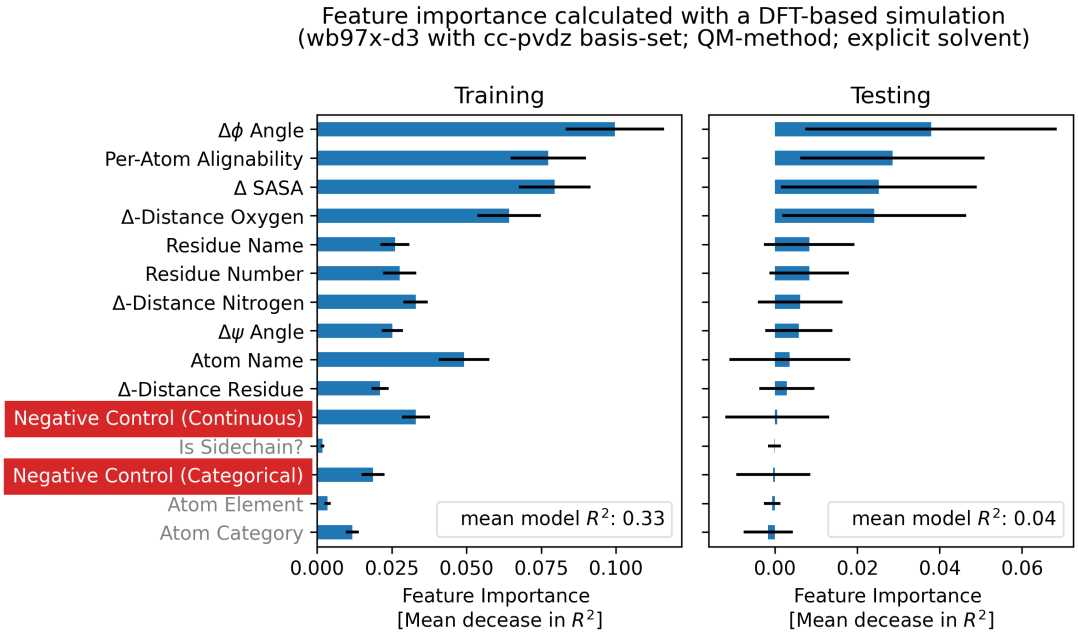

Figure S6.29: Feature importances (ordinal) calculated with the DFT-based QM method using wb97x-d3/cc-pvdz theory with explicit solvent.

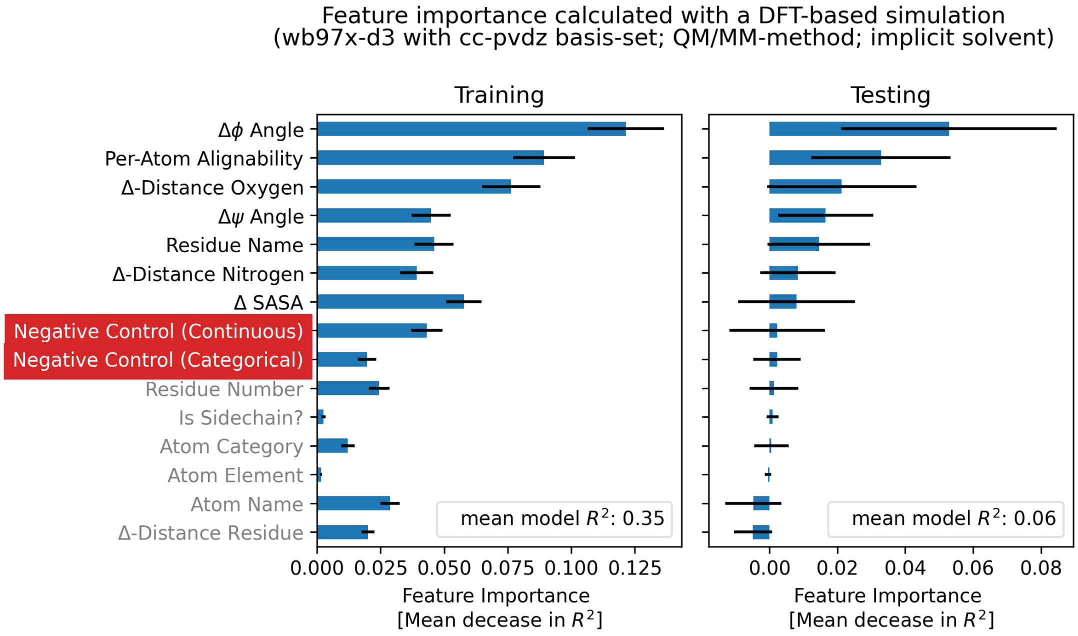

Figure S6.30: Feature importances (ordinal) calculated with the DFT-based QM/MM method using wb97x-d3/cc-pvdz theory with implicit solvent.

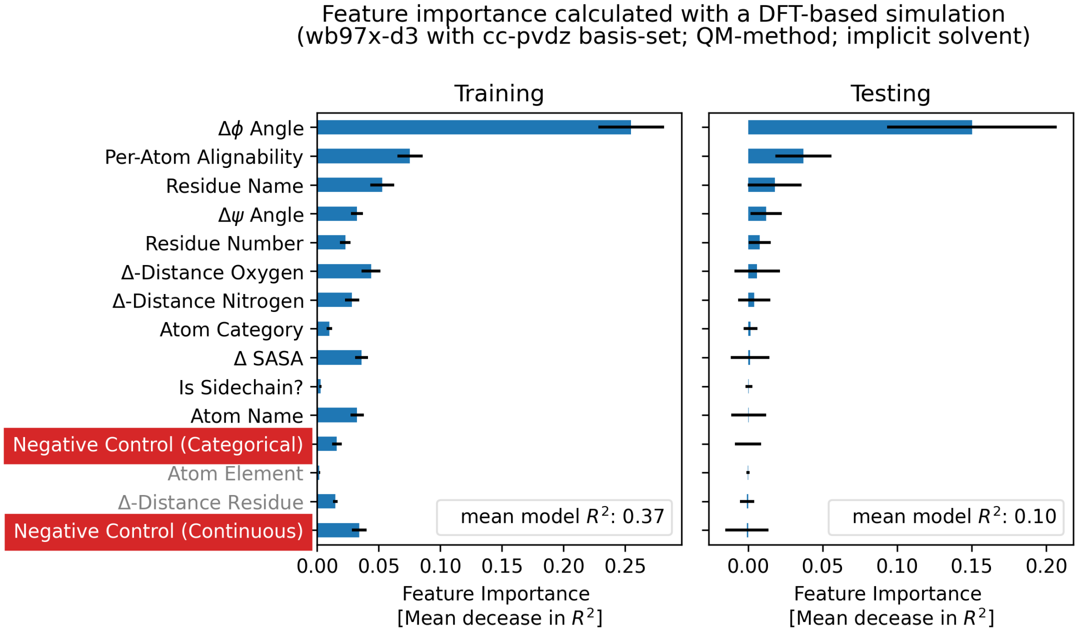

Figure S6.31: Feature importances (ordinal) calculated with the DFT-based QM method using wb97x-d3/cc-pvdz theory with implicit solvent.

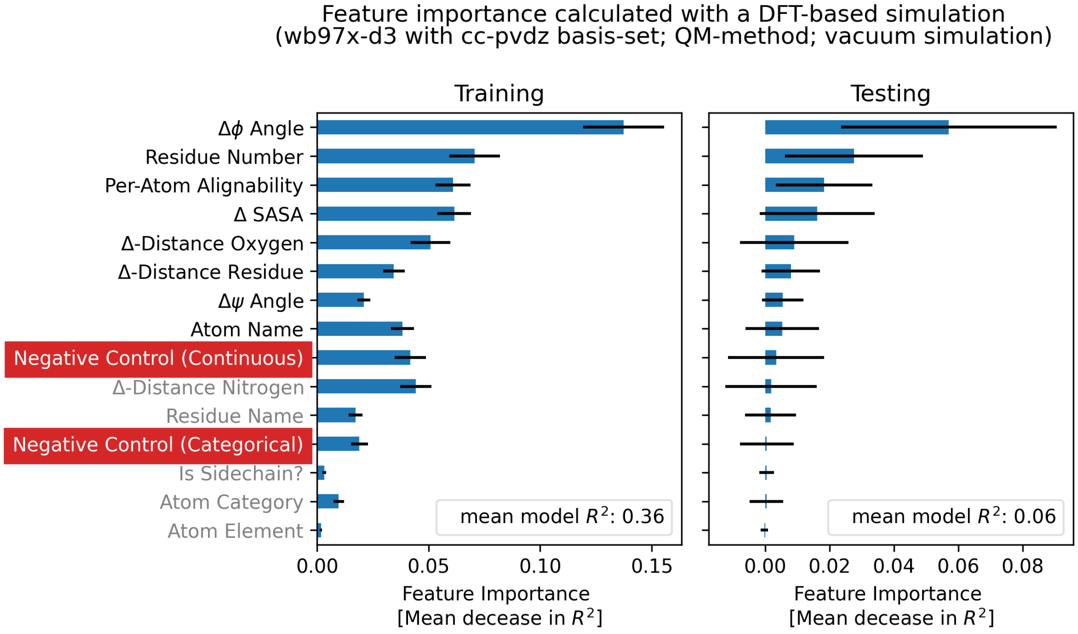

Figure S6.32: Feature importances (ordinal) calculated with the DFT-based QM method using wb97x-d3/cc-pvdz theory in vacuum.

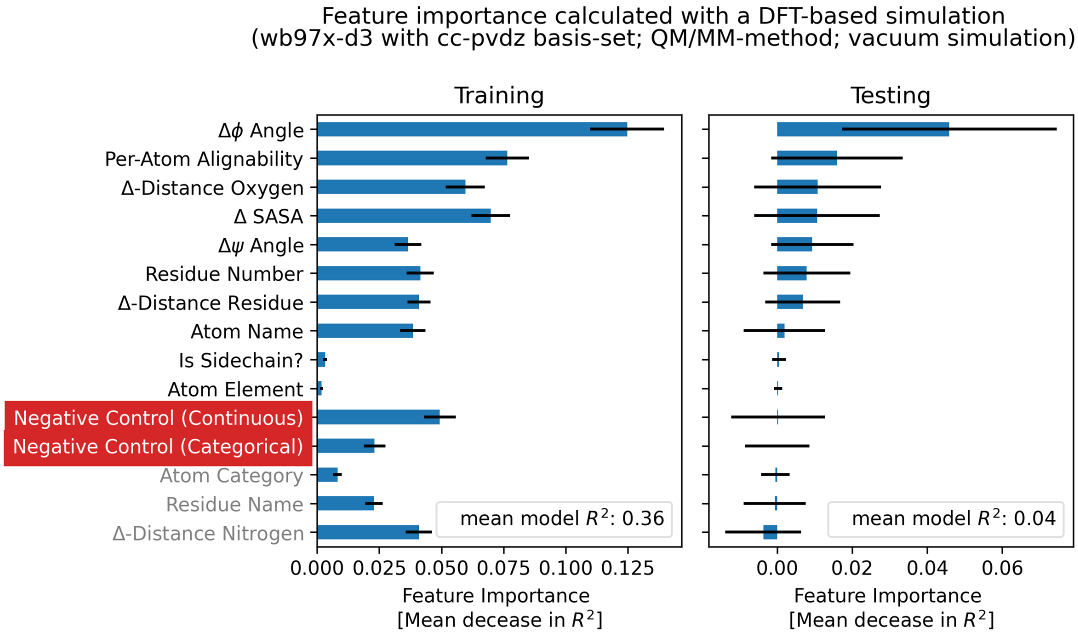

Figure S6.33: Feature importances (ordinal) calculated with the DFT-based QM/MM method using wb97x-d3/cc-pvdz theory in vacuum.

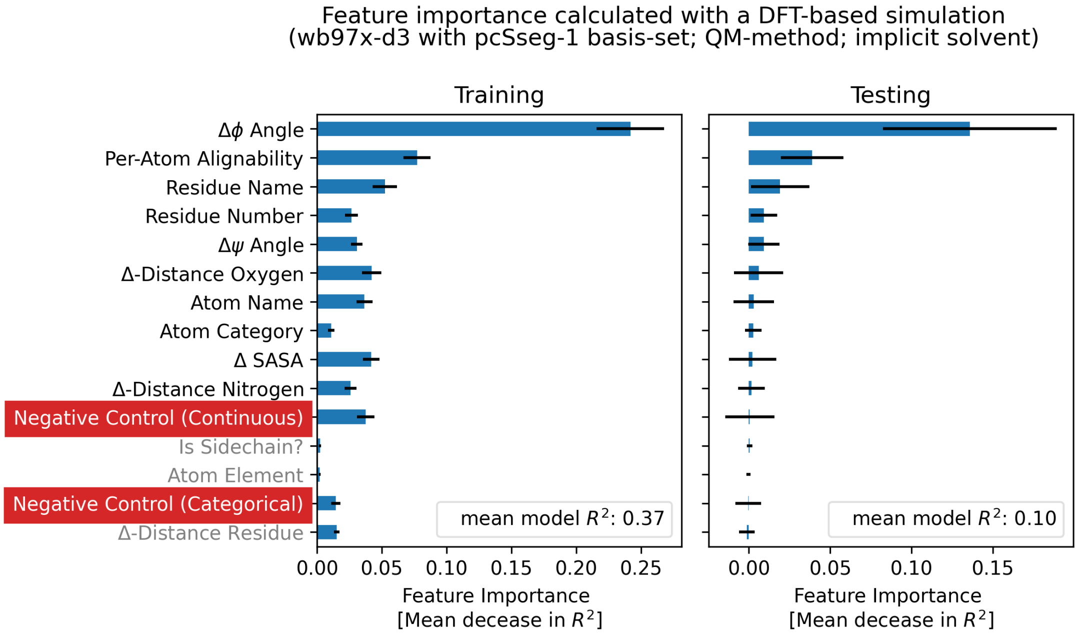

Figure S6.34: Feature importances (ordinal) calculated with the DFT-based QM method using wb97x-d3/pcSseg-1 theory with implicit solvent.

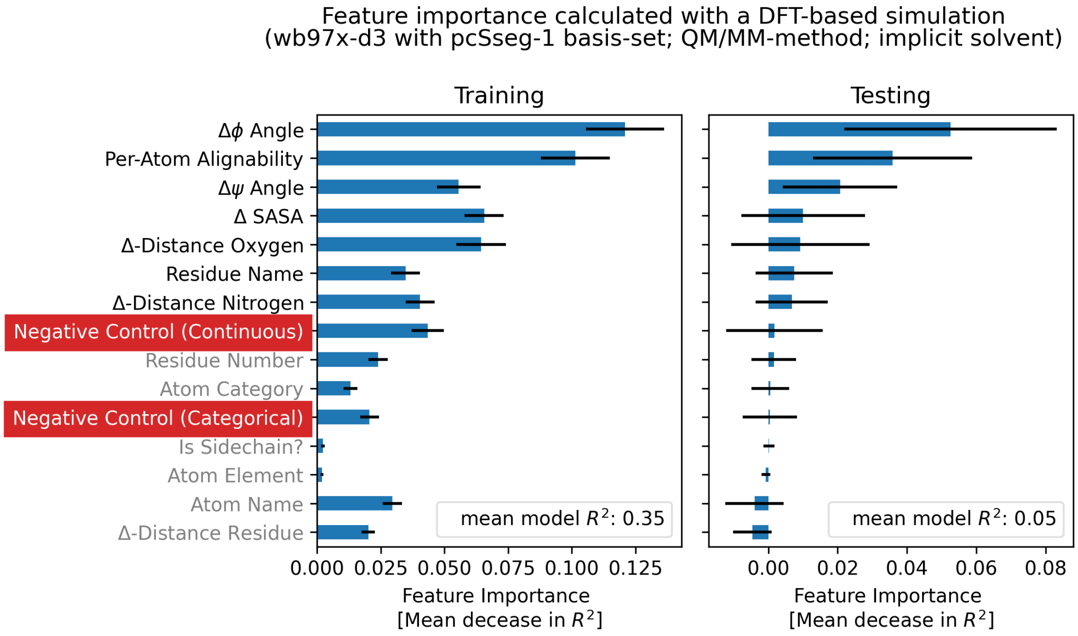

Figure S6.35: Feature importances (ordinal) calculated with the DFT-based QM/MM method using wb97x-d3/pcSseg-1 theory with implicit solvent.

1.7 Feature Importance using OneHot Encoding

The following figures show the feature importances using onehot encoding.

A table of content to find each graphic can be found in table 4.

Table 4: Table of content for the onehot encoded feature importances of the methods.

Name	Type	Solvation	Figure	Page
PPM	empirical	vacuum	S7.1	99
UCBShiftX	empirical	vacuum	S7.2	100
shiftX2	empirical	vacuum	S7.3	101
sparta+	empirical	vacuum	S7.4	102
b3lyp/6-31G*	QM/MM	implicit	S7.6	104
b3lyp/6-31G*	QM	implicit	S7.6	104
b3lyp/cc-pvdz	QM/MM	vacuum	S7.10	108
b3lyp/cc-pvdz	QM	explicit	S7.8	106
b3lyp/cc-pvdz	QM/MM	implicit	S7.11	109
b3lyp/cc-pvdz	QM	vacuum	S7.10	108
b3lyp/cc-pvdz	QM	implicit	S7.11	109
b3lyp/pcSseg-1	QM	implicit	S7.13	111
b3lyp/pcSseg-1	QM/MM	implicit	S7.13	111
becke97-2/6-31G*	QM/MM	implicit	S7.15	113
becke97-2/6-31G*	QM	implicit	S7.15	113
becke97-2/cc-pvdz	QM	implicit	S7.17	115
becke97-2/cc-pvdz	QM/MM	implicit	S7.17	115
becke97-2/pcSseg-1	QM	implicit	S7.19	117
becke97-2/pcSseg-1	QM/MM	implicit	S7.19	117
becke97-d/6-31G*	QM	implicit	S7.21	119
becke97-d/6-31G*	QM/MM	implicit	S7.21	119
becke97-d/cc-pvdz	QM/MM	implicit	S7.23	121
becke97-d/cc-pvdz	QM	implicit	S7.23	121
becke97-d/pcSseg-1	QM/MM	implicit	S7.26	124
becke97-d/pcSseg-1	QM	explicit	S7.25	123
becke97-d/pcSseg-1	QM	implicit	S7.26	124
wb97x-d3/6-31G*	QM	implicit	S7.28	126
wb97x-d3/6-31G*	QM/MM	implicit	S7.28	126
wb97x-d3/cc-pvdz	QM	explicit	S7.29	127
wb97x-d3/cc-pvdz	QM/MM	implicit	S7.31	129
wb97x-d3/cc-pvdz	QM	implicit	S7.31	129
wb97x-d3/cc-pvdz	QM	vacuum	S7.33	131
wb97x-d3/cc-pvdz	QM/MM	vacuum	S7.33	131
wb97x-d3/pcSseg-1	QM	implicit	S7.35	133
wb97x-d3/pcSseg-1	QM/MM	implicit	S7.35	133

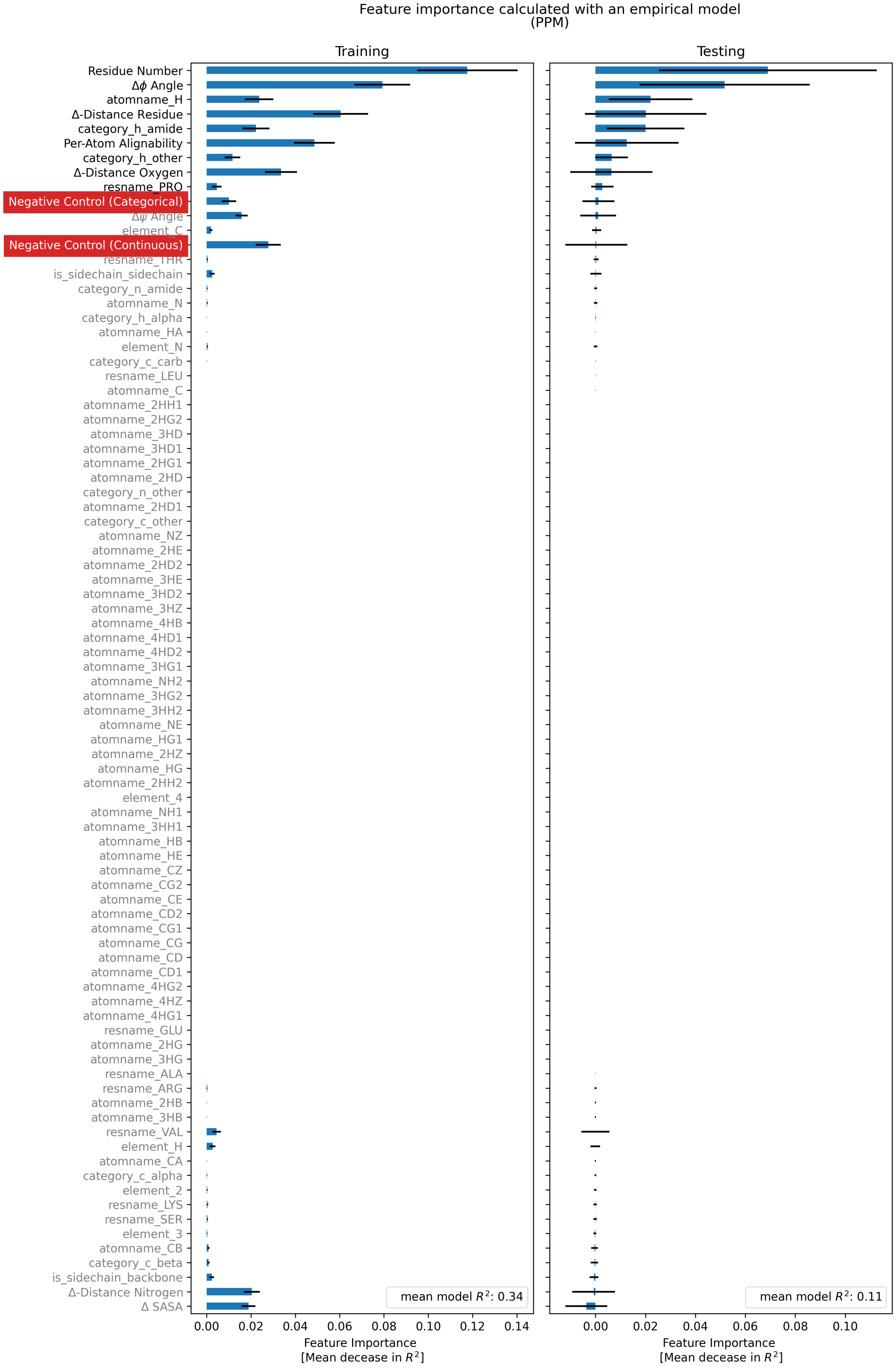

Figure S7.1: Feature importances (onehot) calculated with the empirical method PPM.

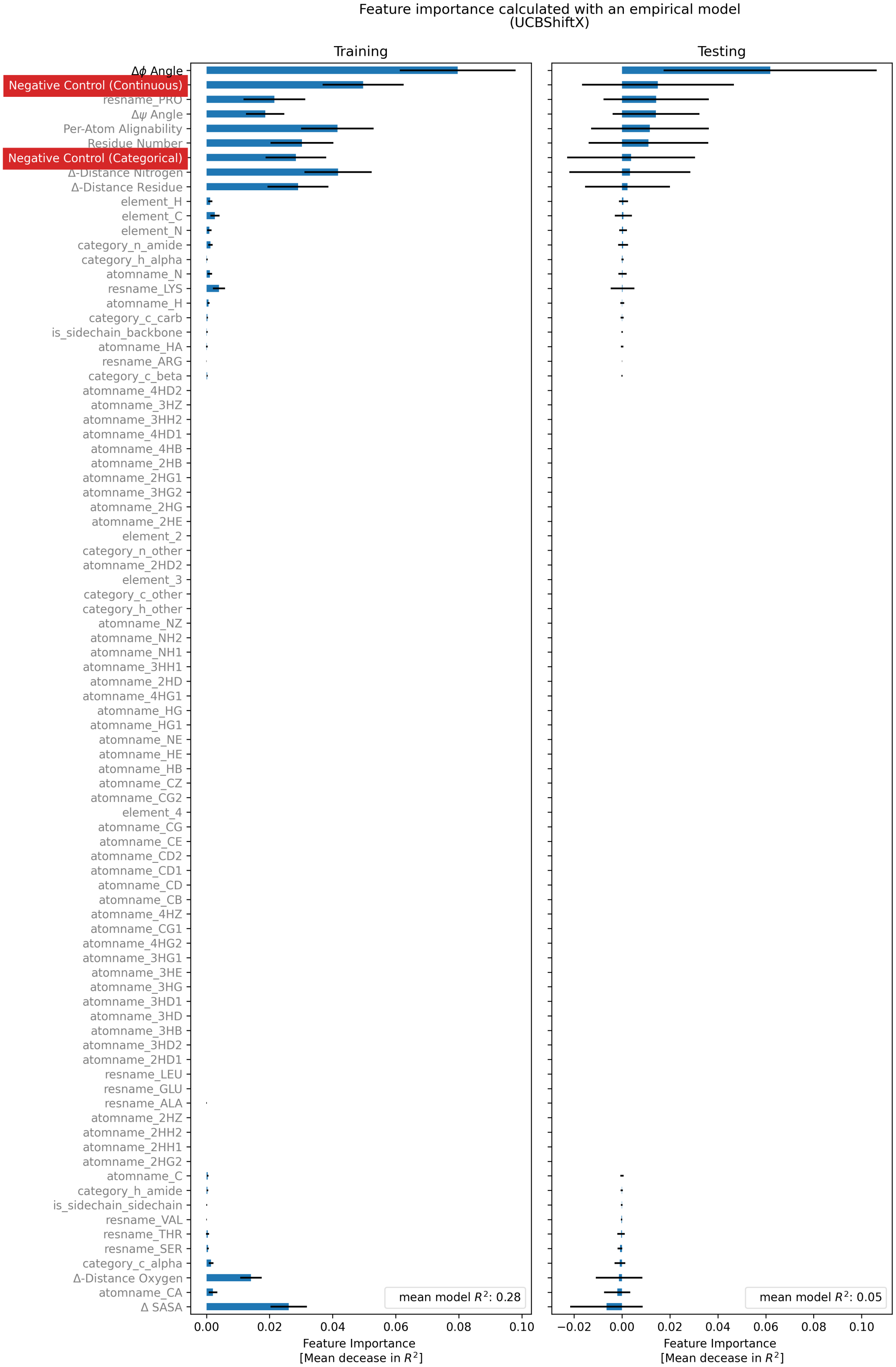

Figure S7.2: Feature importances (onehot) calculated with the empirical method UCBShiftX.

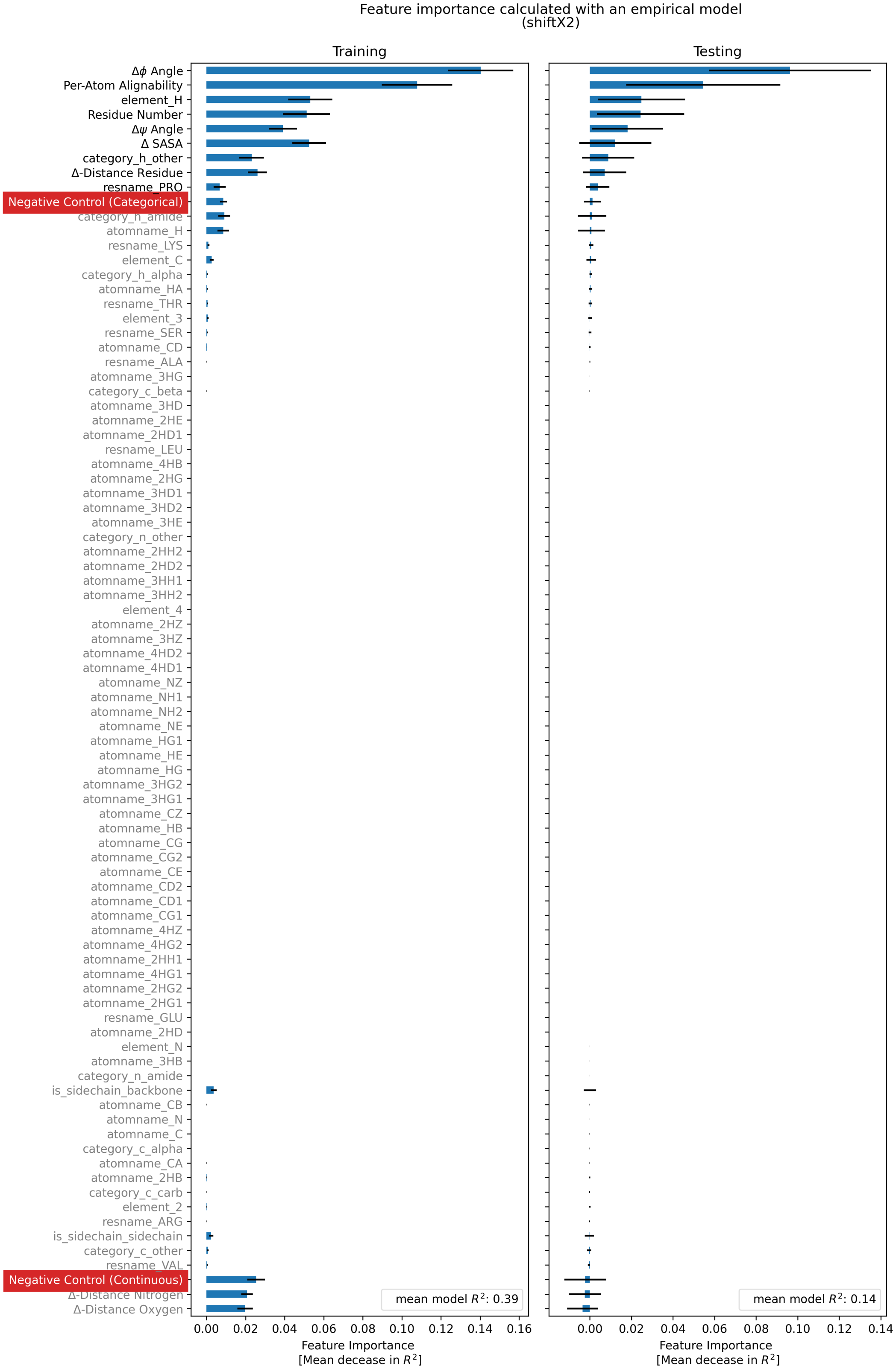

Figure S7.3: Feature importances (onehot) calculated with the empirical method shiftX2.

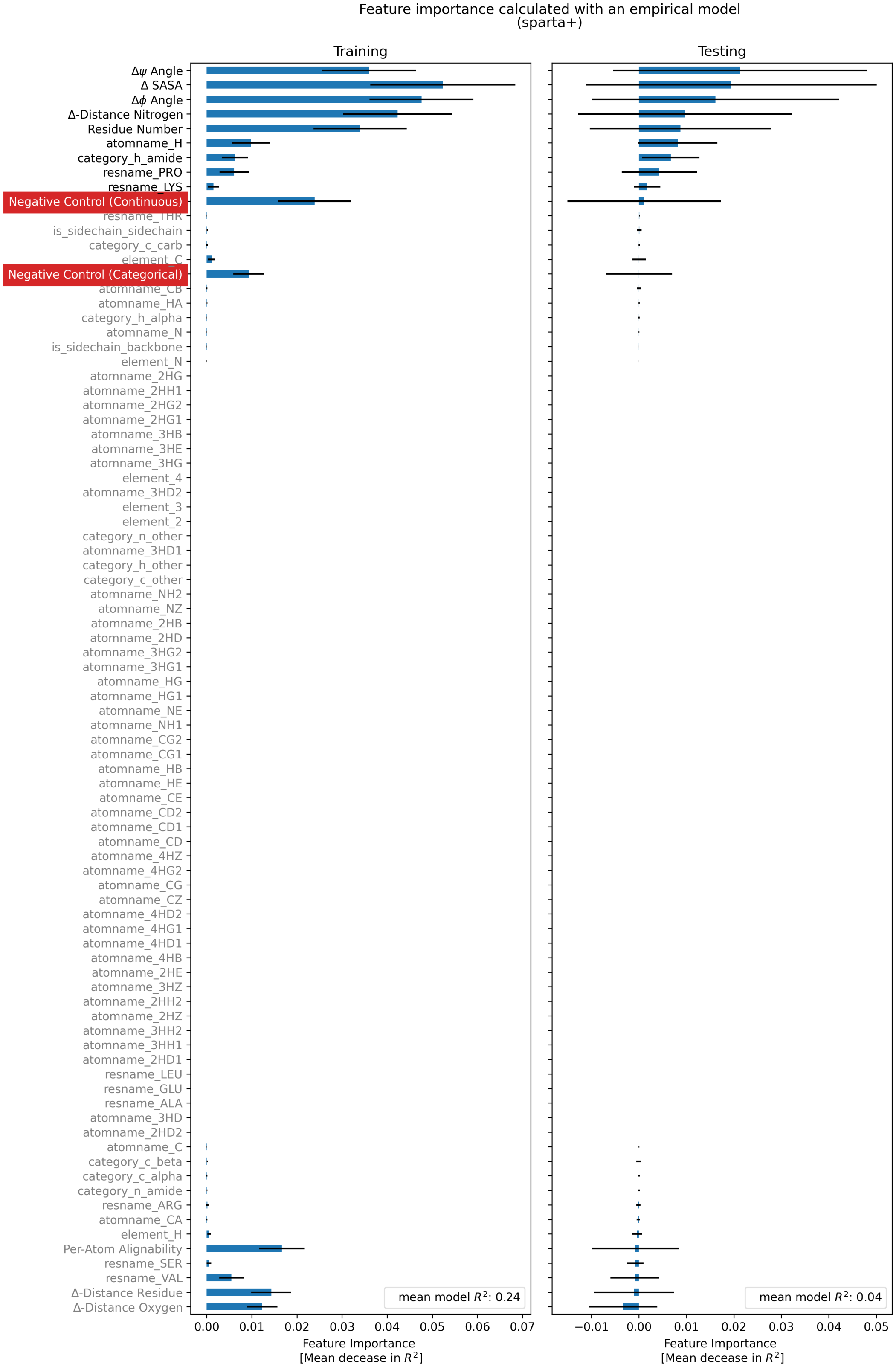

Figure S7.4: Feature importances (onehot) calculated with the empirical method sparta+.

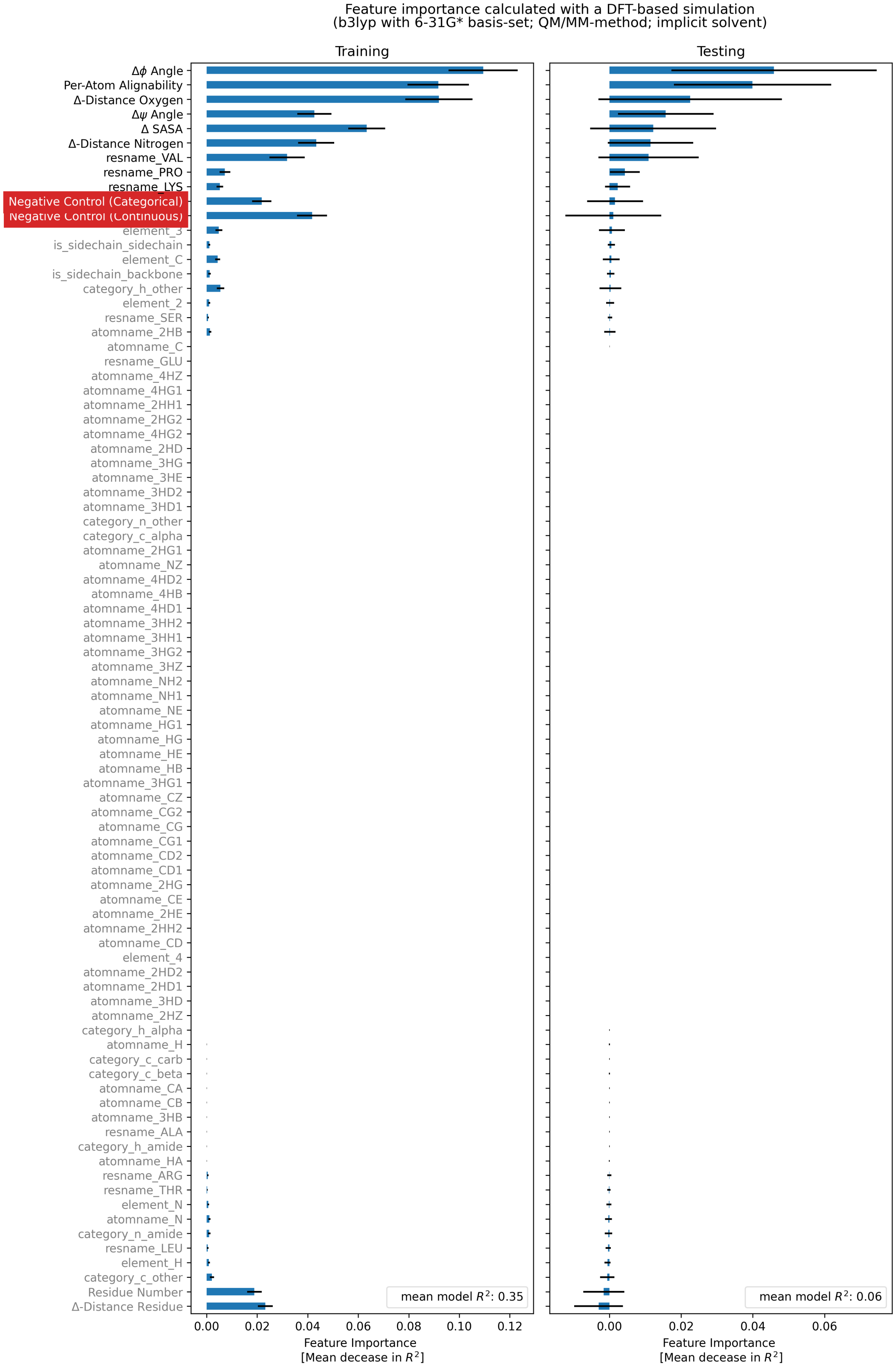

Figure S7.5: Feature importances (onehot) calculated with the DFT-based QM/MM method using b3lyp/6-31G* theory with implicit solvent.

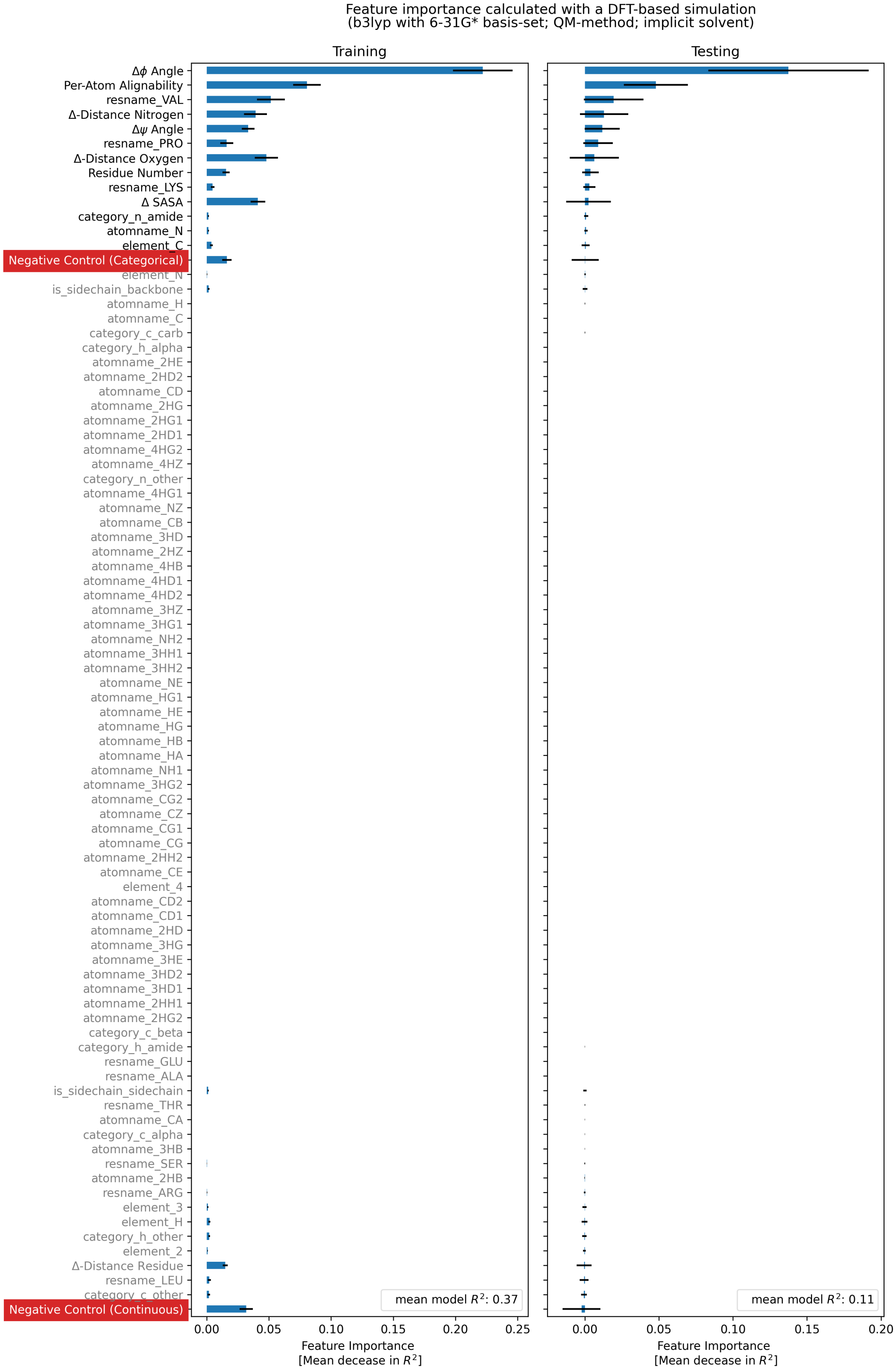

Figure S7.6: Feature importances (onehot) calculated with the DFT-based QM method using b3lyp/6-31G* theory with implicit solvent.

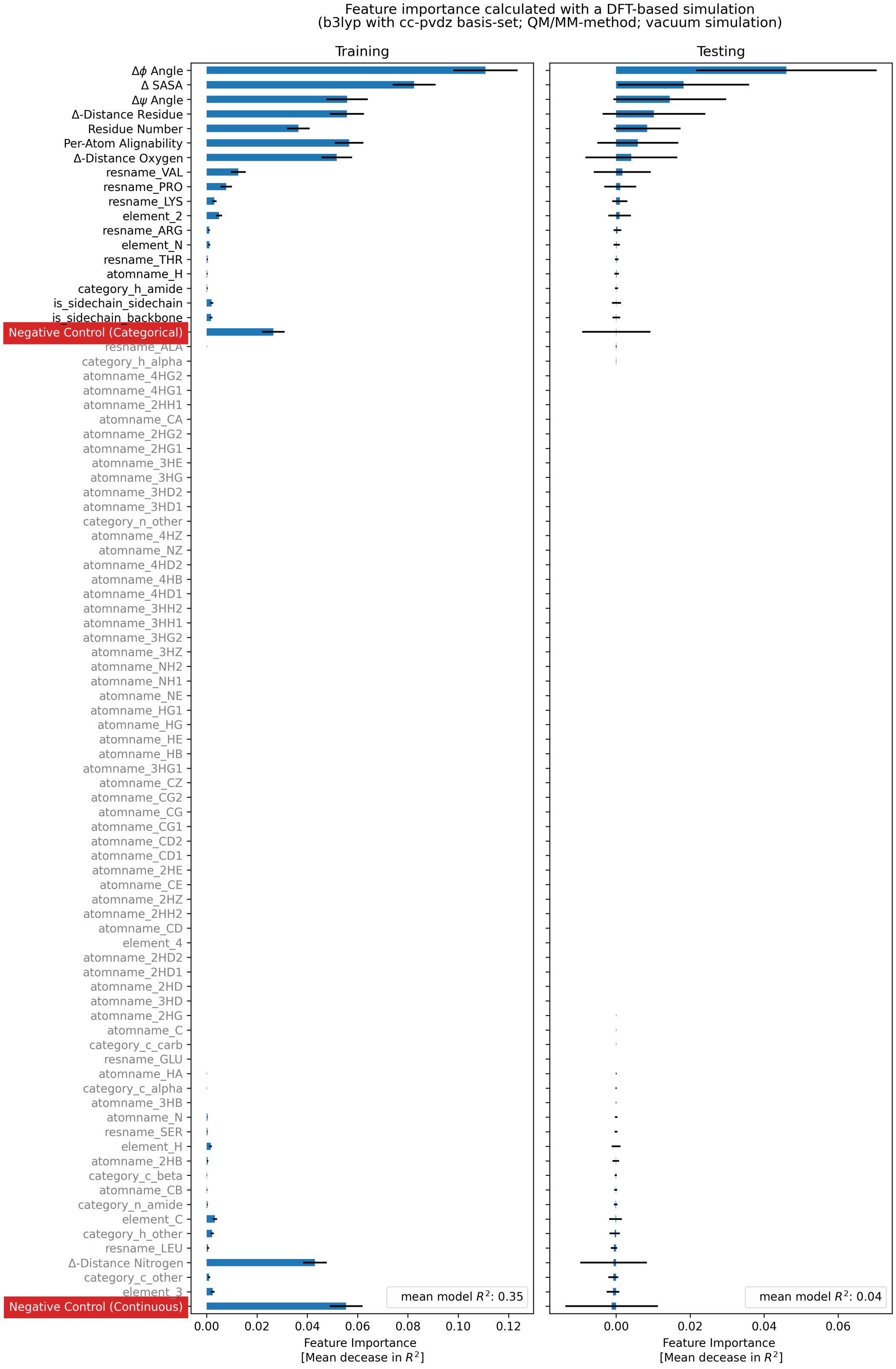

Figure S7.7: Feature importances (onehot) calculated with the DFT-based QM/MM method using b3lyp/cc-pvdz theory in vacuum.

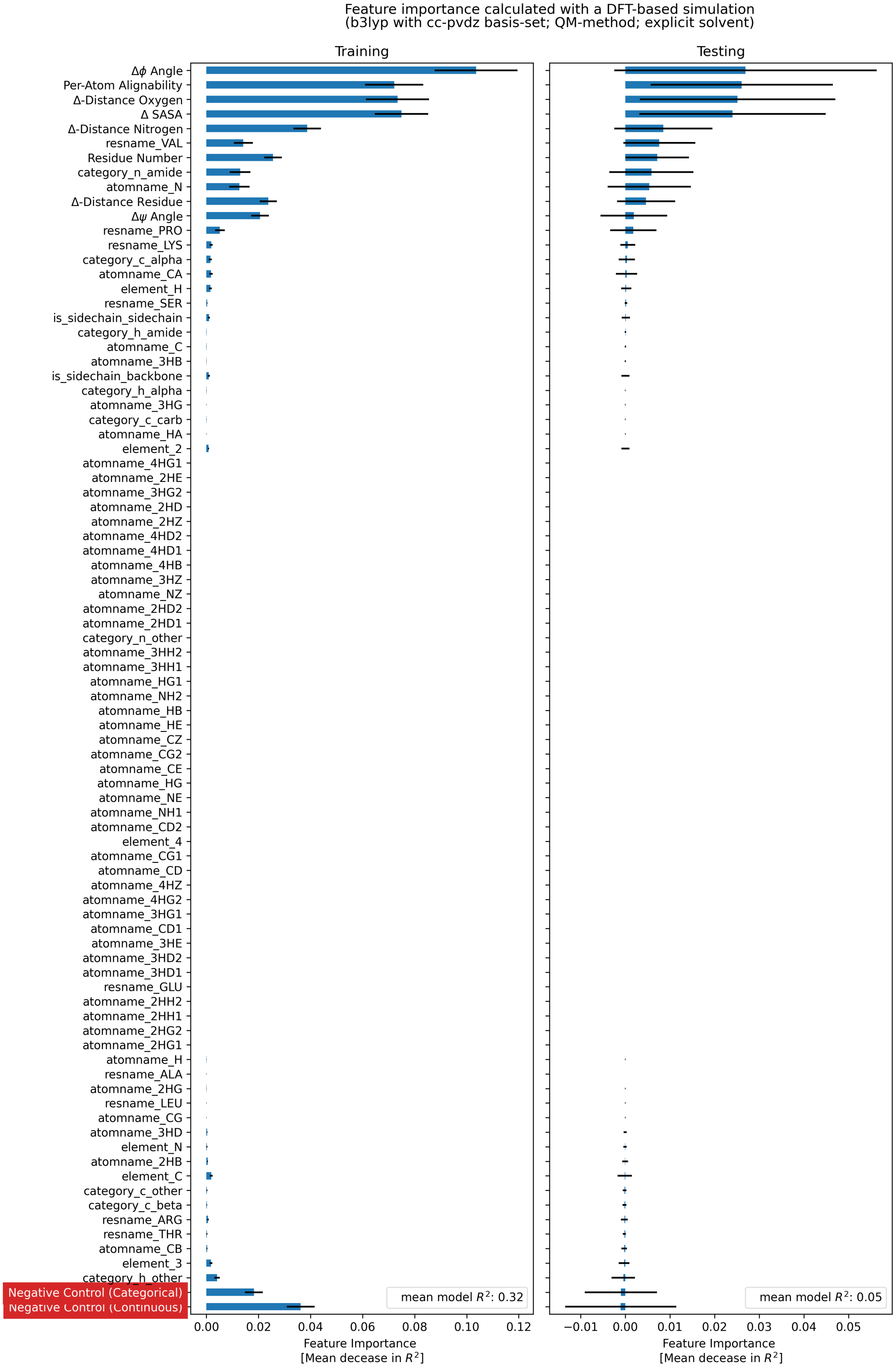

Figure S7.8: Feature importances (onehot) calculated with the DFT-based QM method using b3lyp/cc-pvdz theory with explicit solvent.

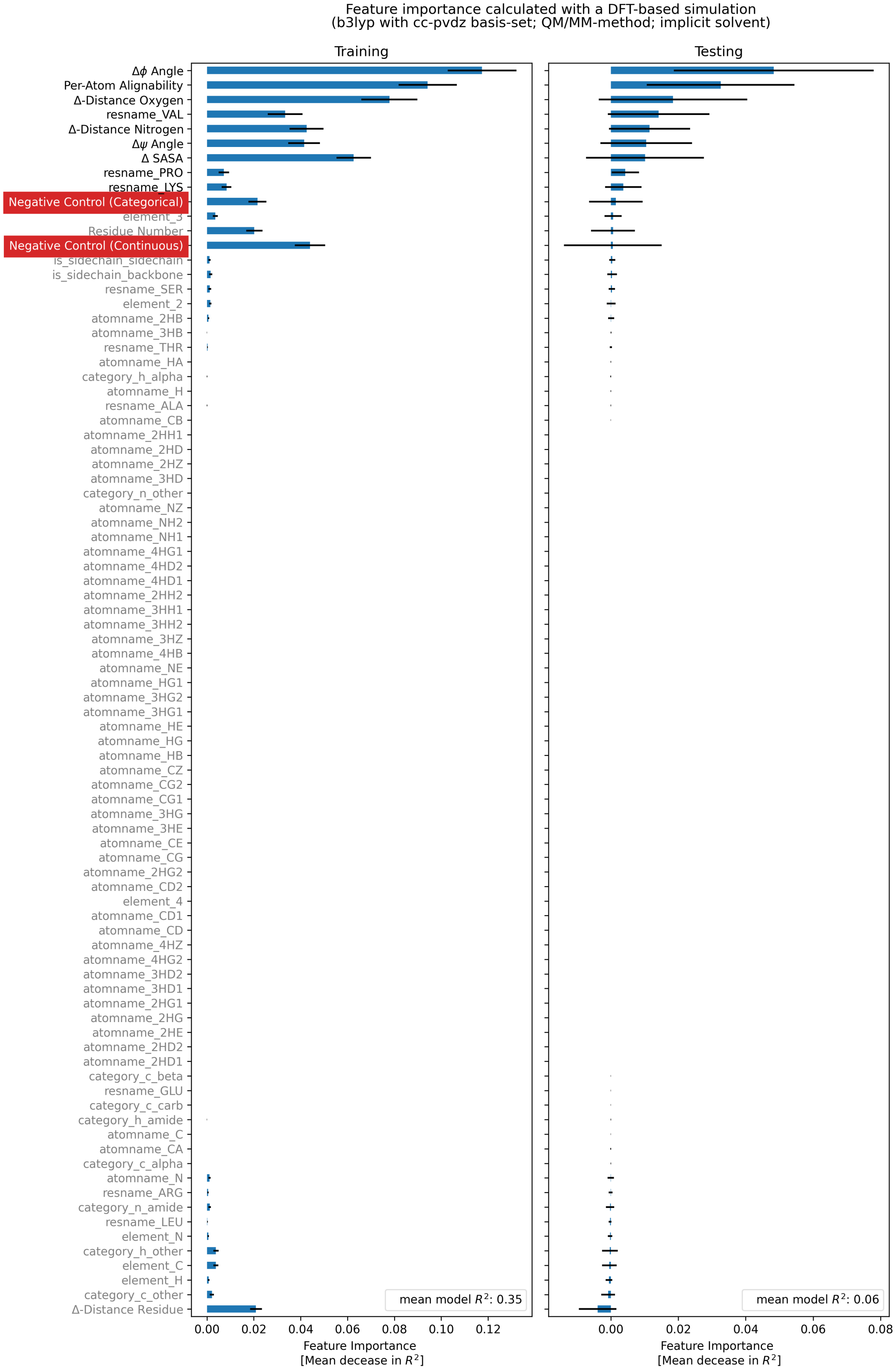

Figure S7.9: Feature importances (onehot) calculated with the DFT-based QM/MM method using b3lyp/cc-pvdz theory with implicit solvent.

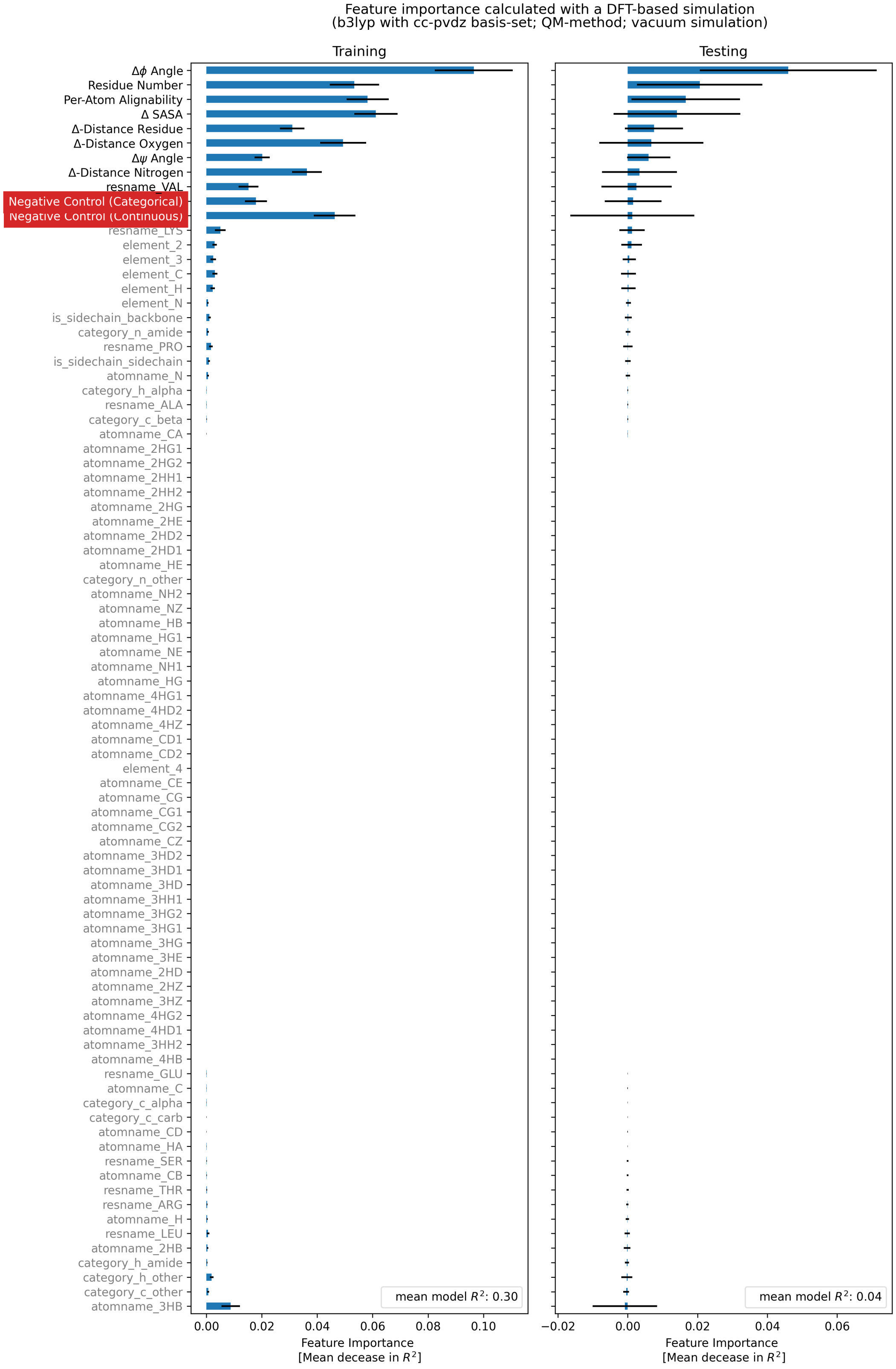

Figure S7.10: Feature importances (onehot) calculated with the DFT-based QM method using b3lyp/cc-pvdz theory in vacuum.

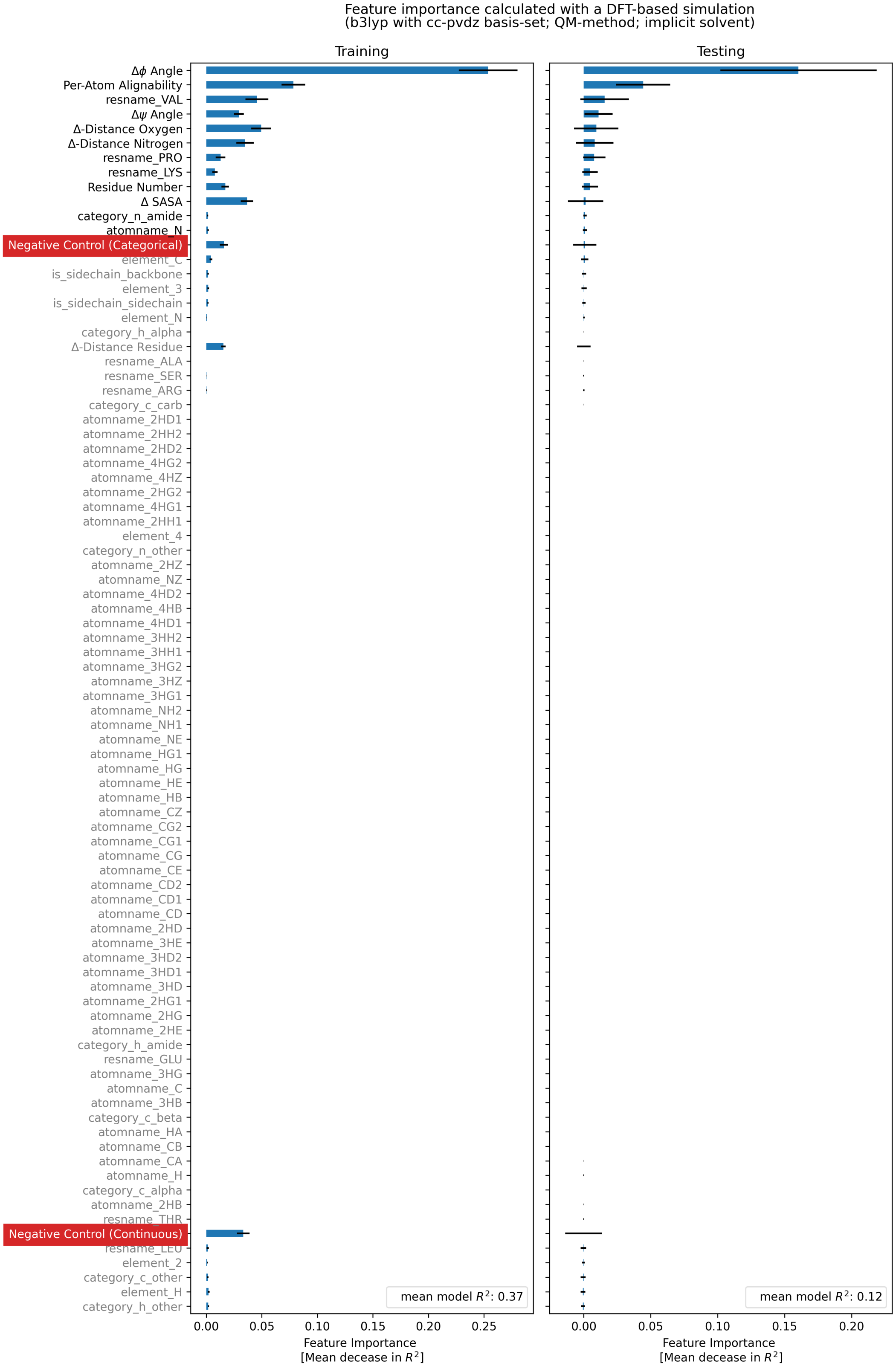

Figure S7.11: Feature importances (onehot) calculated with the DFT-based QM method using b3lyp/cc-pvdz theory with implicit solvent.

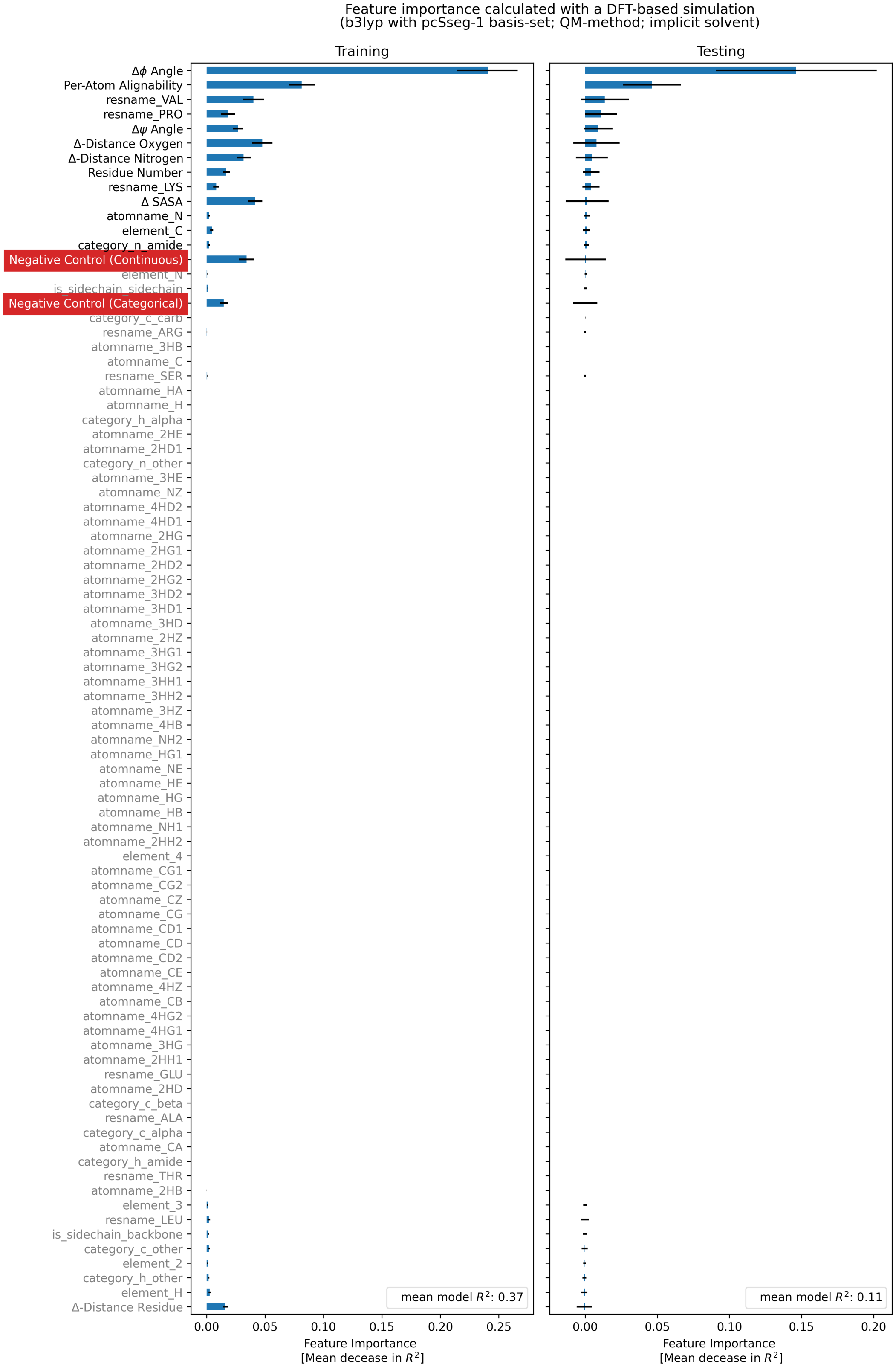

Figure S7.12: Feature importances (onehot) calculated with the DFT-based QM method using b3lyp/pcSseg-1 theory with implicit solvent.

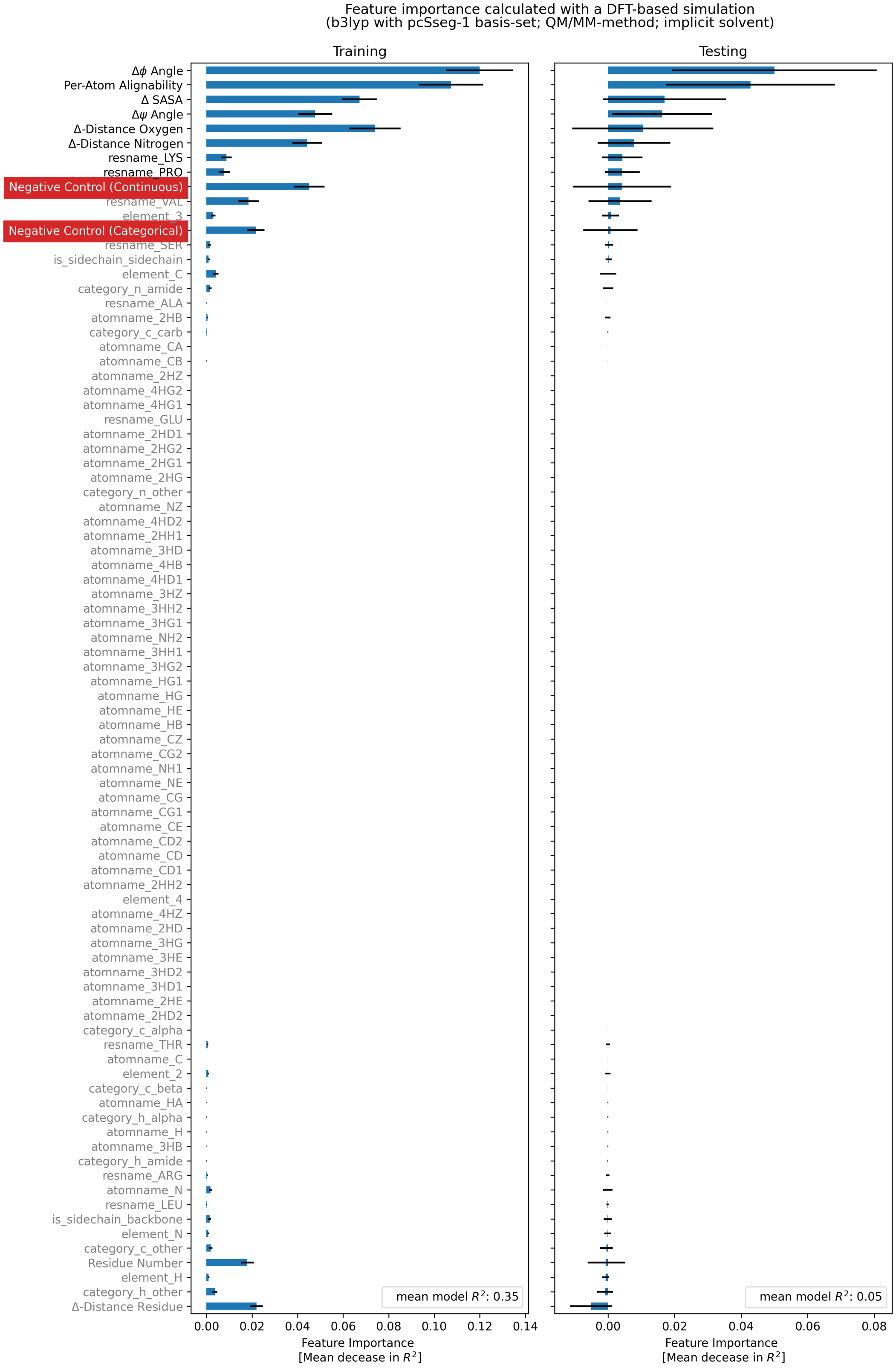

Figure S7.13: Feature importances (onehot) calculated with the DFT-based QM/MM method using b3lyp/pcSseg-1 theory with implicit solvent.

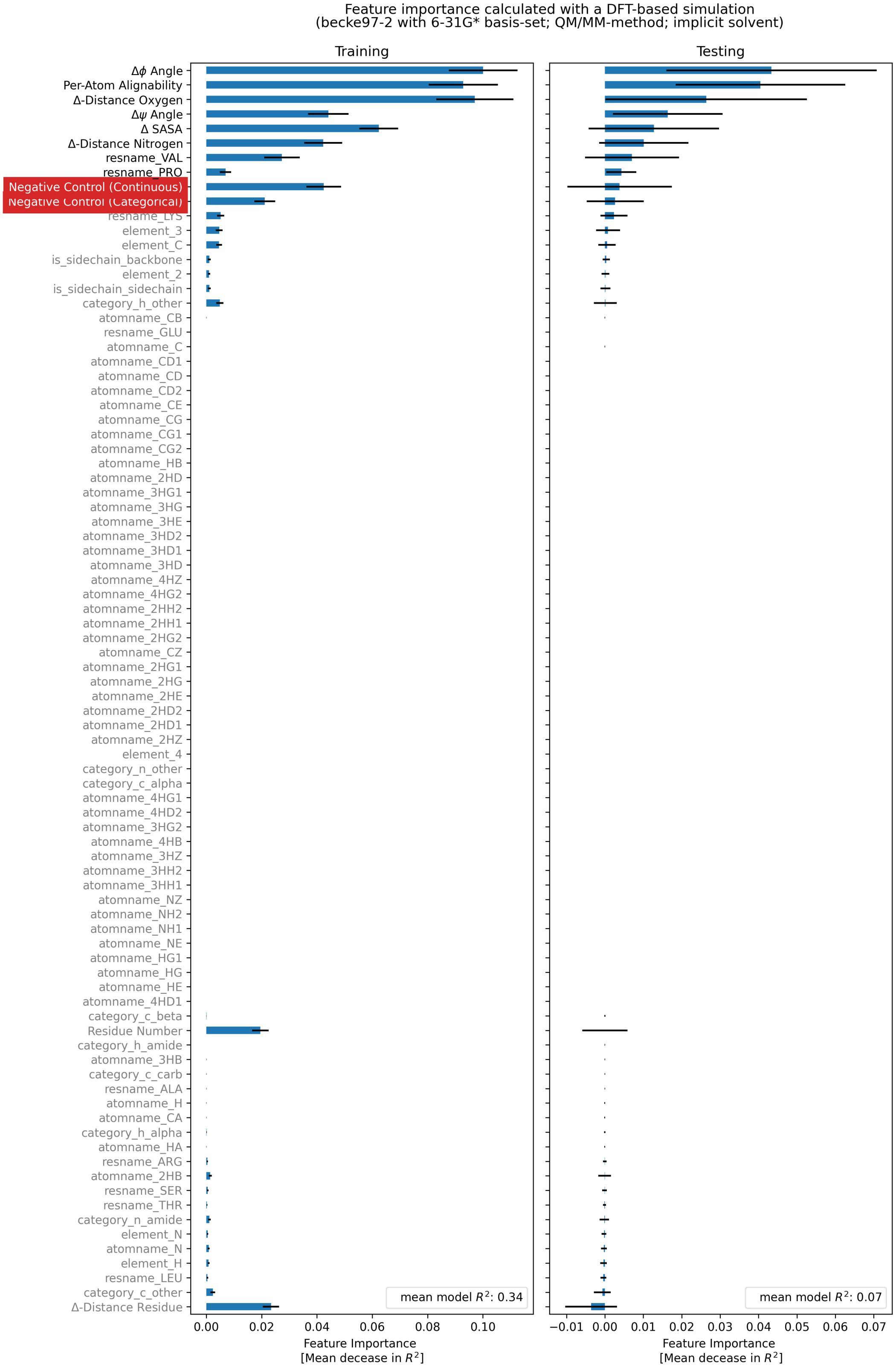

Figure S7.14: Feature importances (onehot) calculated with the DFT-based QM/MM method using becke97-2/6-31G* theory with implicit solvent.

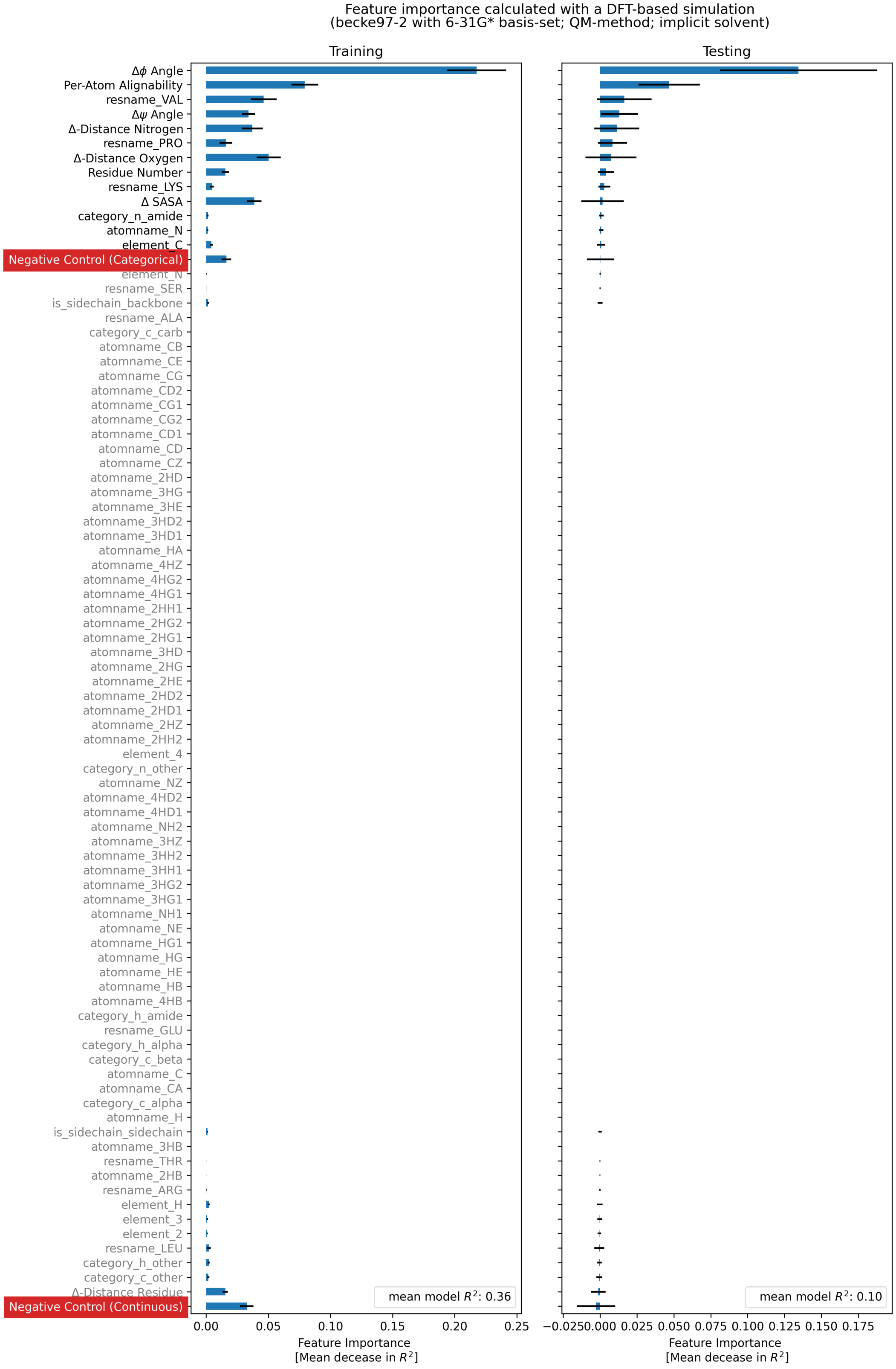

Figure S7.15: Feature importances (onehot) calculated with the DFT-based QM method using becke97-2/6-31G* theory with implicit solvent.

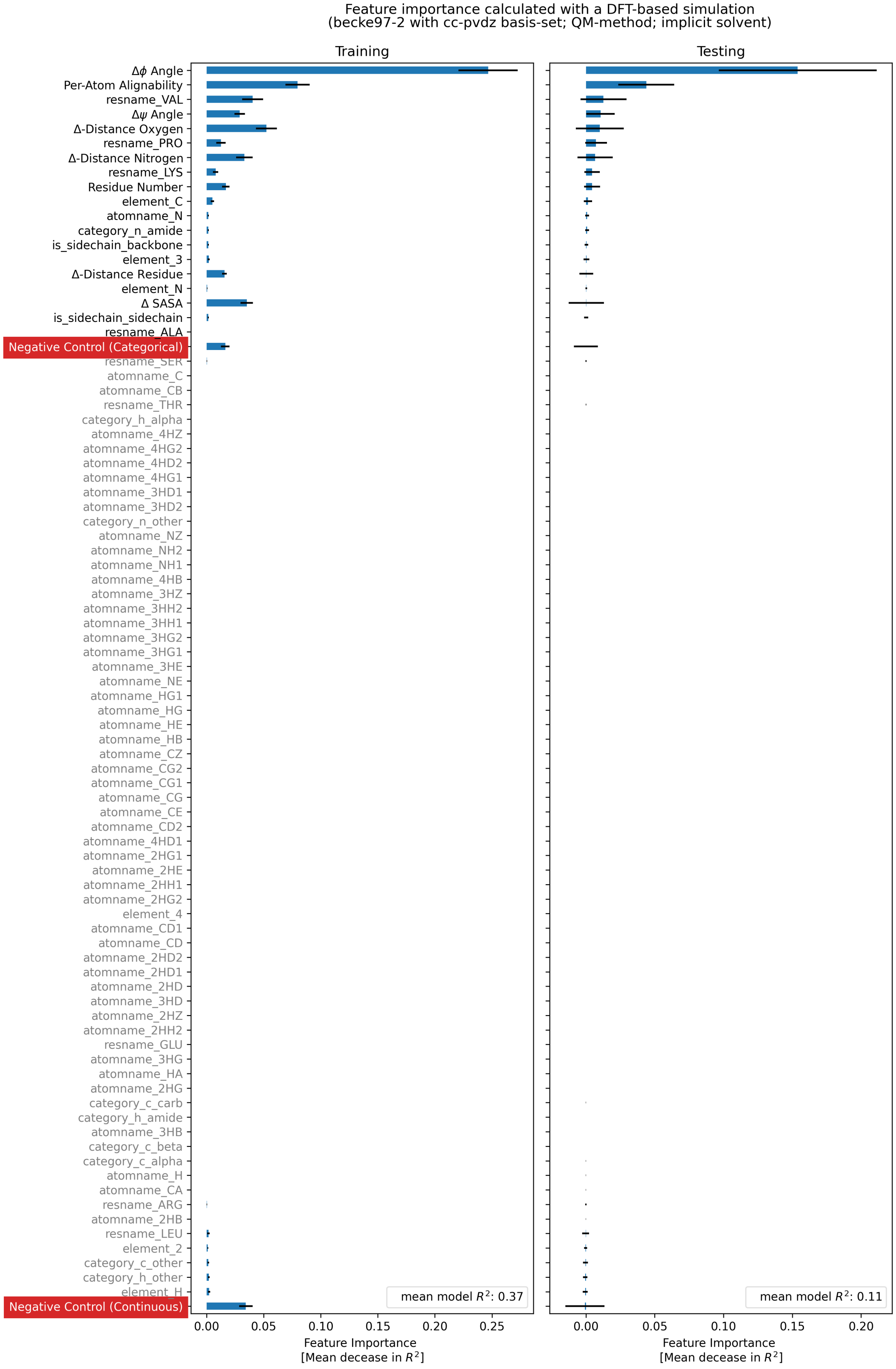

Figure S7.16: Feature importances (onehot) calculated with the DFT-based QM method using becke97-2/cc-pvdz theory with implicit solvent.

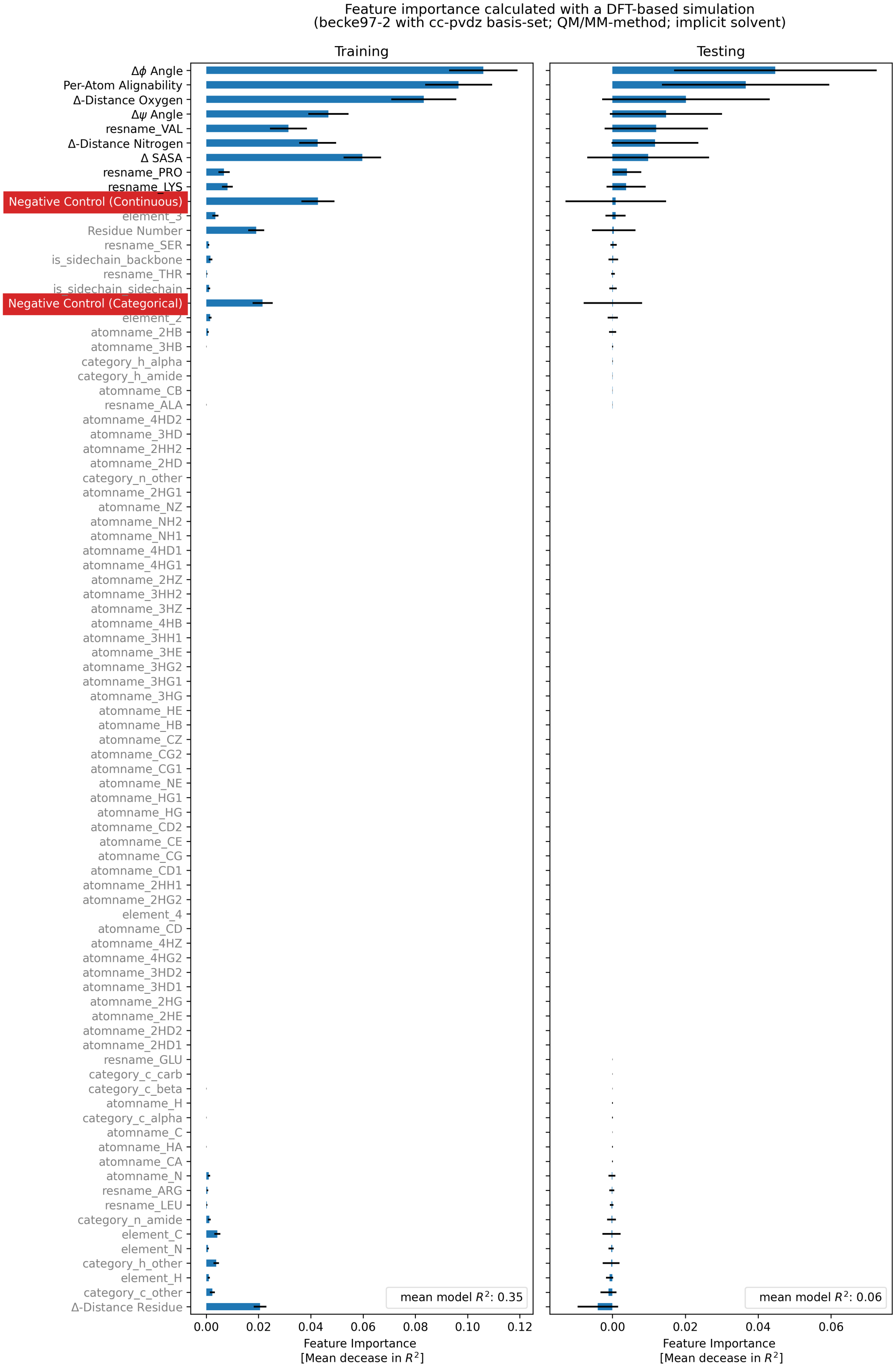

Figure S7.17: Feature importances (onehot) calculated with the DFT-based QM/MM method using becke97-2/cc-pvdz theory with implicit solvent.

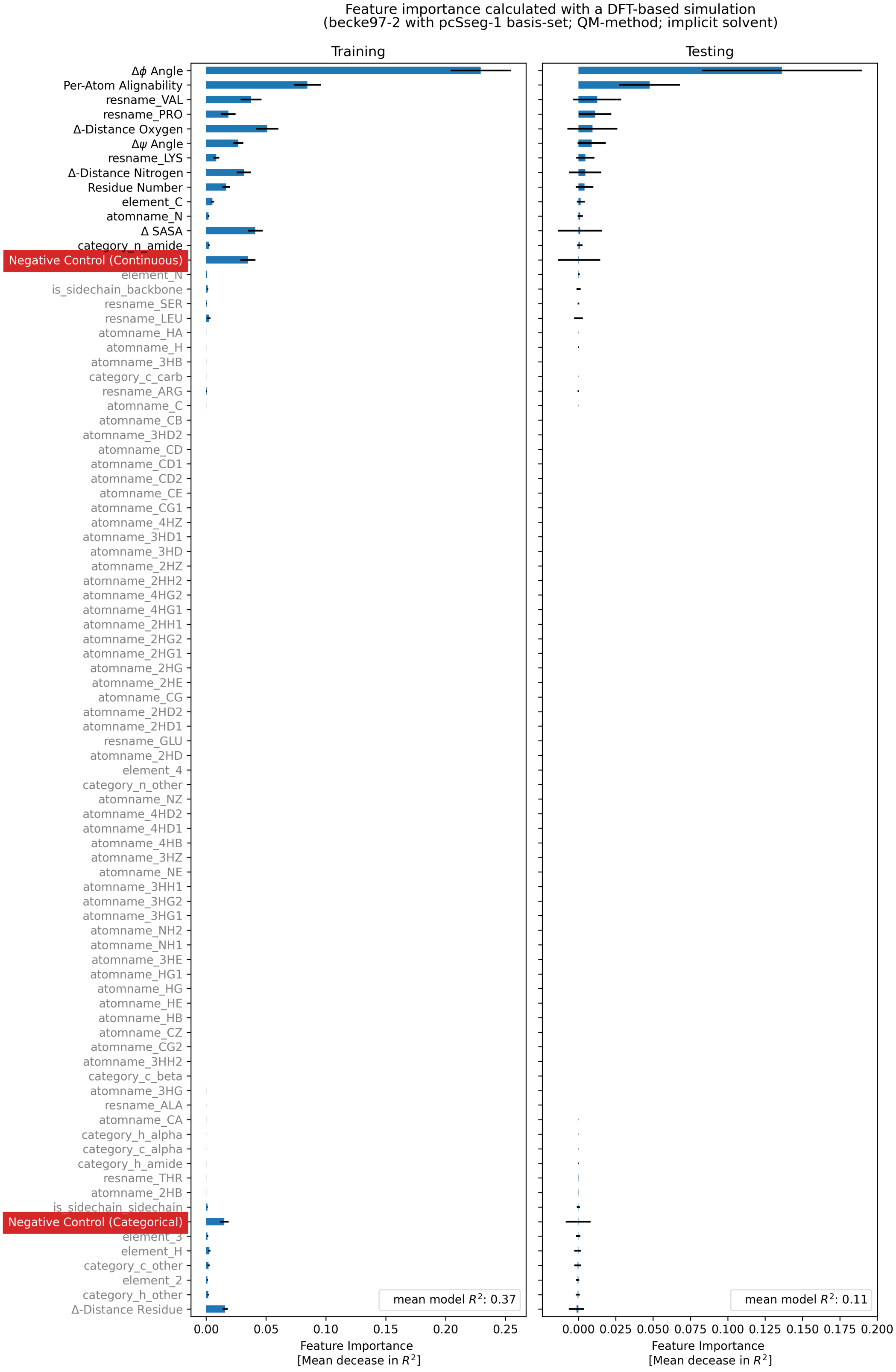

Figure S7.18: Feature importances (onehot) calculated with the DFT-based QM method using becke97-2/pcSseg-1 theory with implicit solvent.

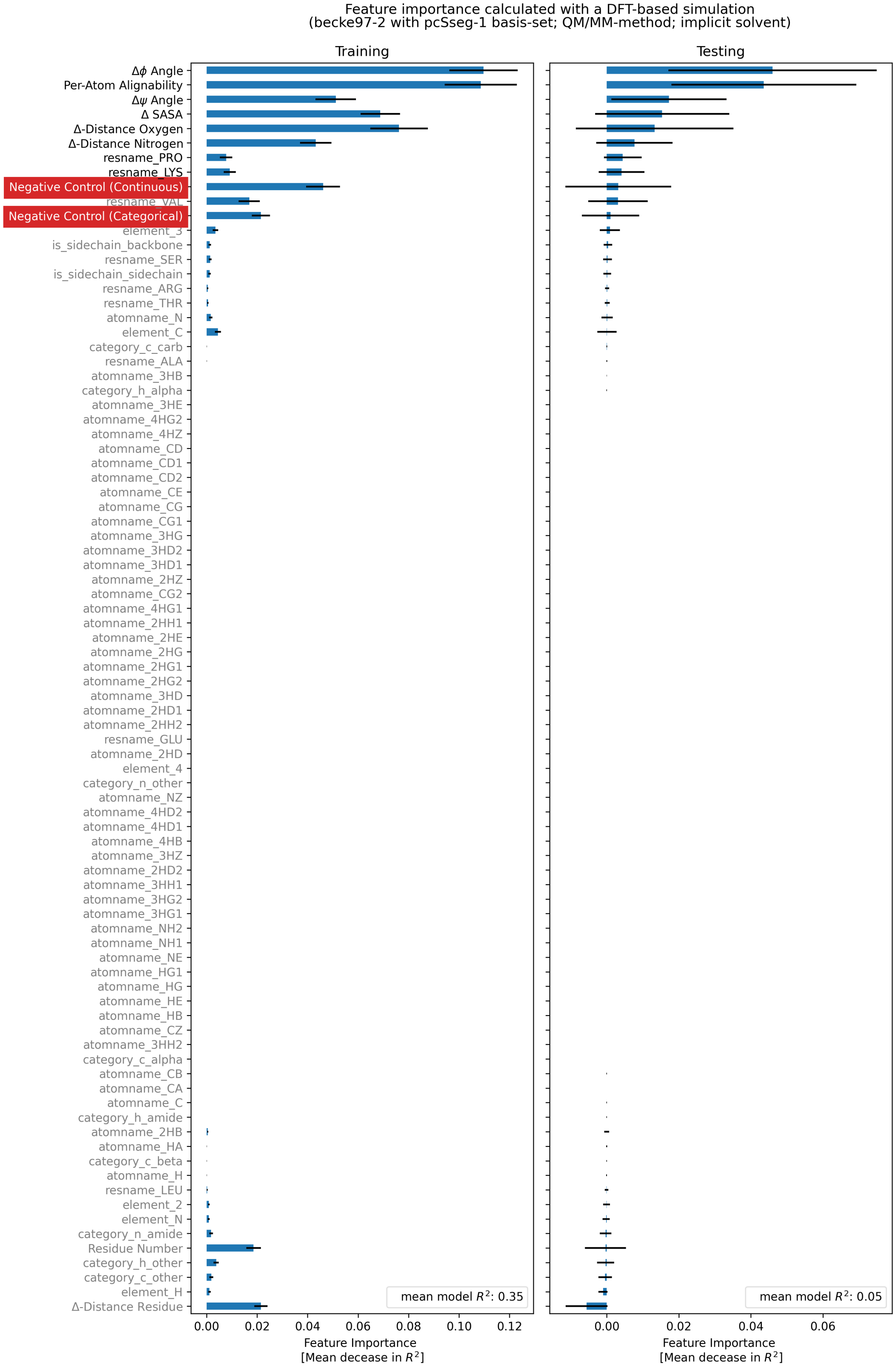

Figure S7.19: Feature importances (onehot) calculated with the DFT-based QM/MM method using becke97-2/pcSseg-1 theory with implicit solvent.

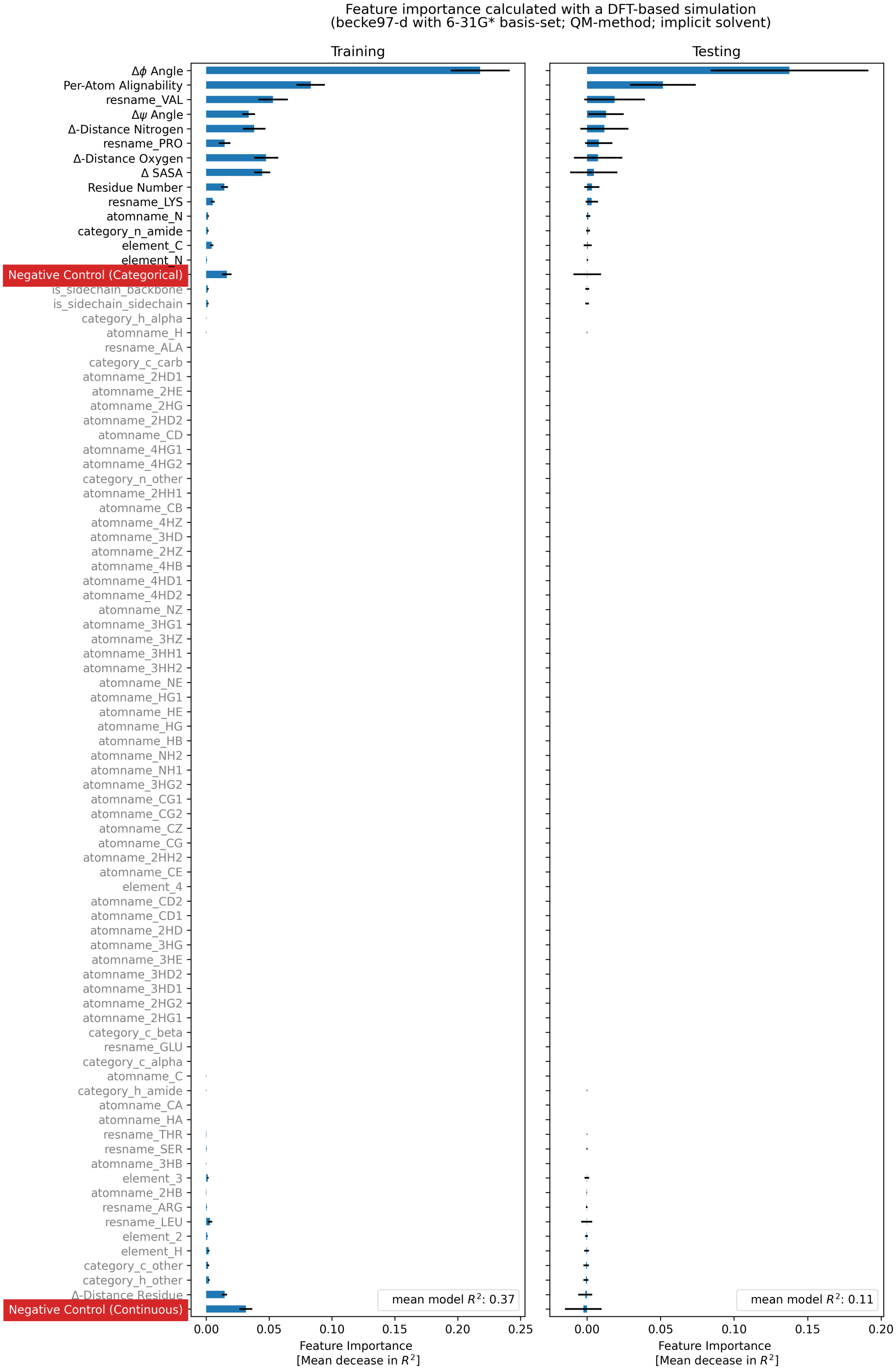

Figure S7.20: Feature importances (onehot) calculated with the DFT-based QM method using becke97-d/6-31G* theory with implicit solvent.

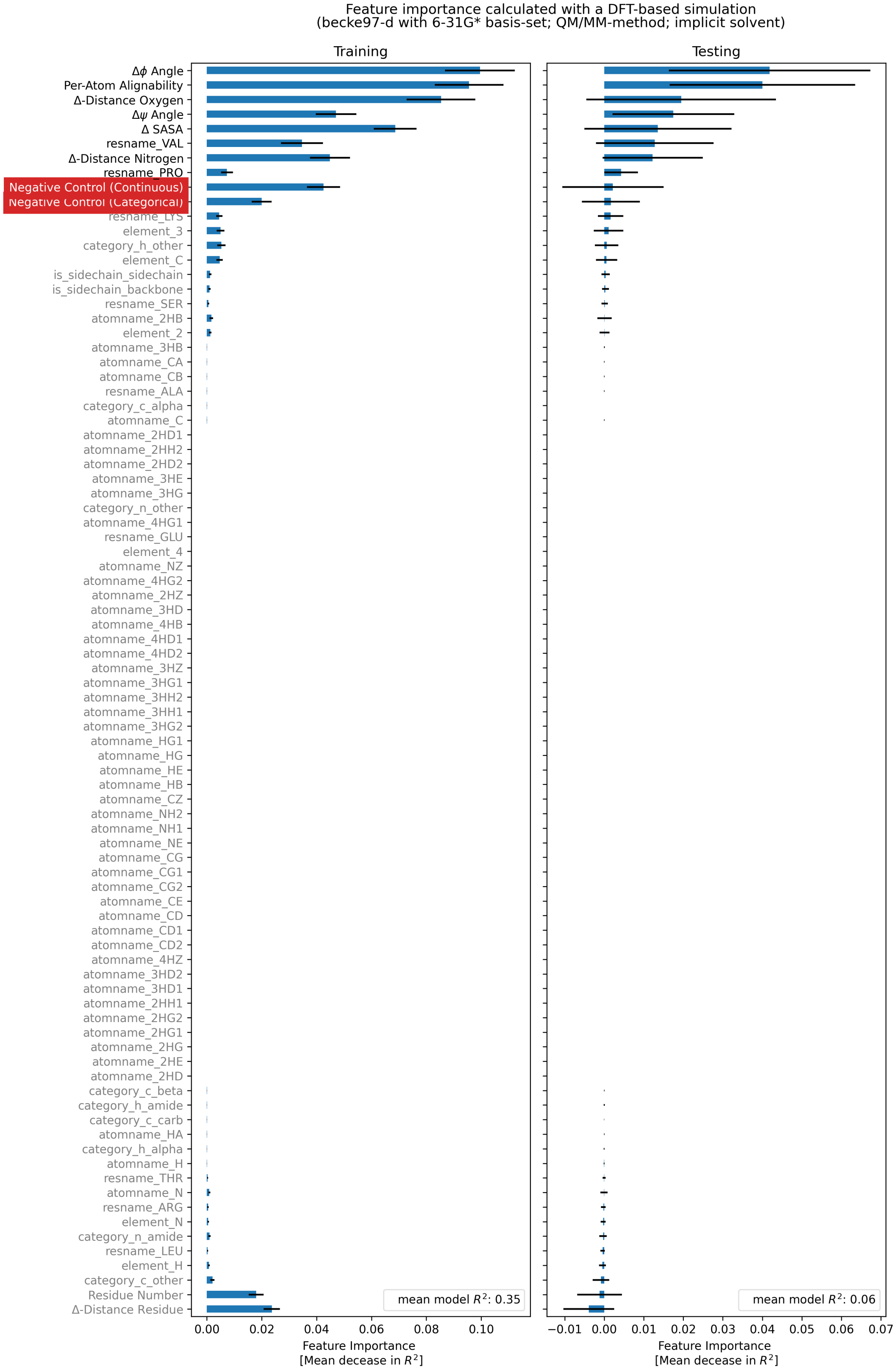

Figure S7.21: Feature importances (onehot) calculated with the DFT-based QM/MM method using becke97-d/6-31G* theory with implicit solvent.

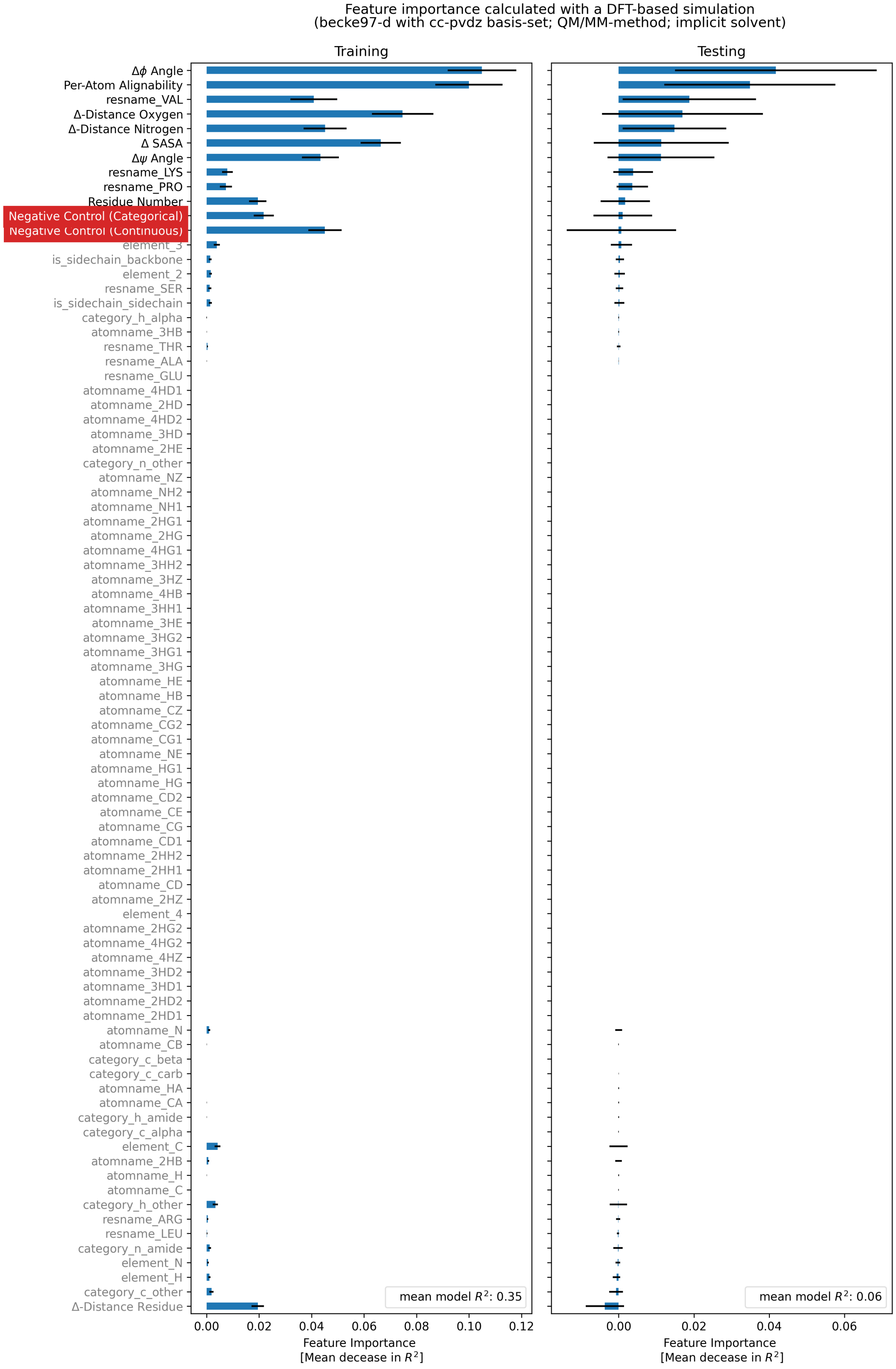

Figure S7.22: Feature importances (onehot) calculated with the DFT-based QM/MM method using becke97-d/cc-pvdz theory with implicit solvent.

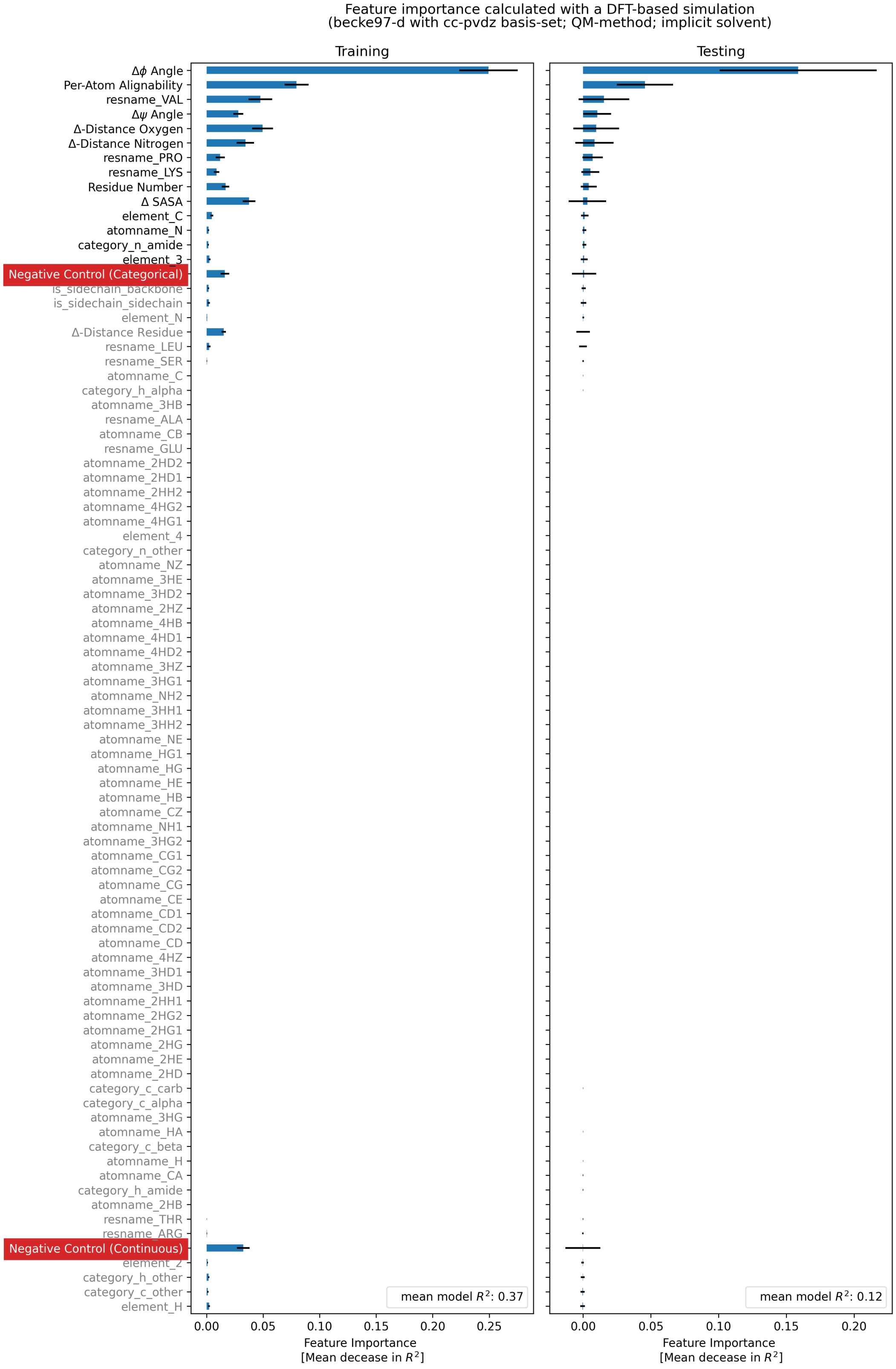

Figure S7.23: Feature importances (onehot) calculated with the DFT-based QM method using becke97-d/cc-pvdz theory with implicit solvent.

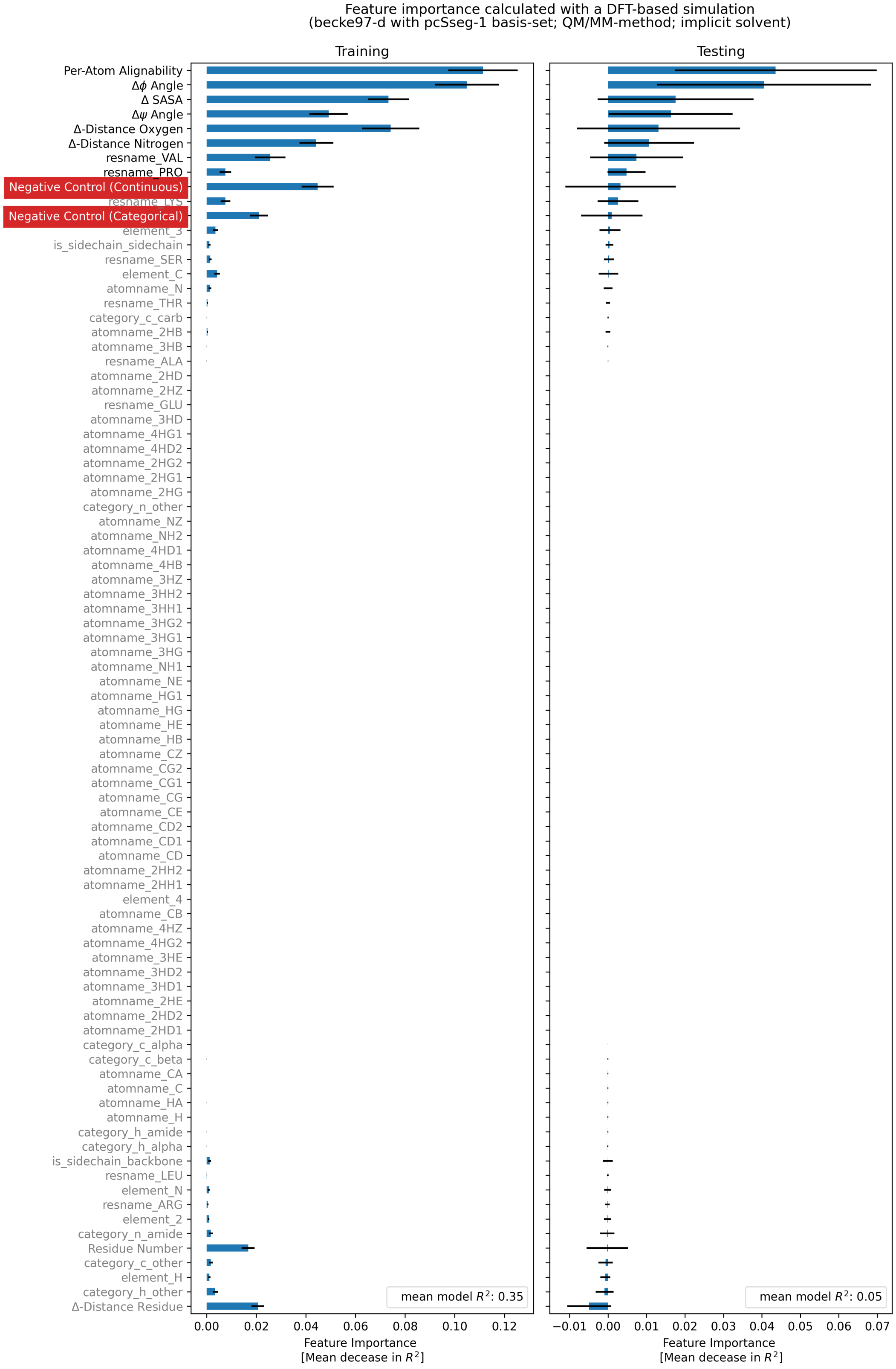

Figure S7.24: Feature importances (onehot) calculated with the DFT-based QM/MM method using becke97-d/pcSseg-1 theory with implicit solvent.

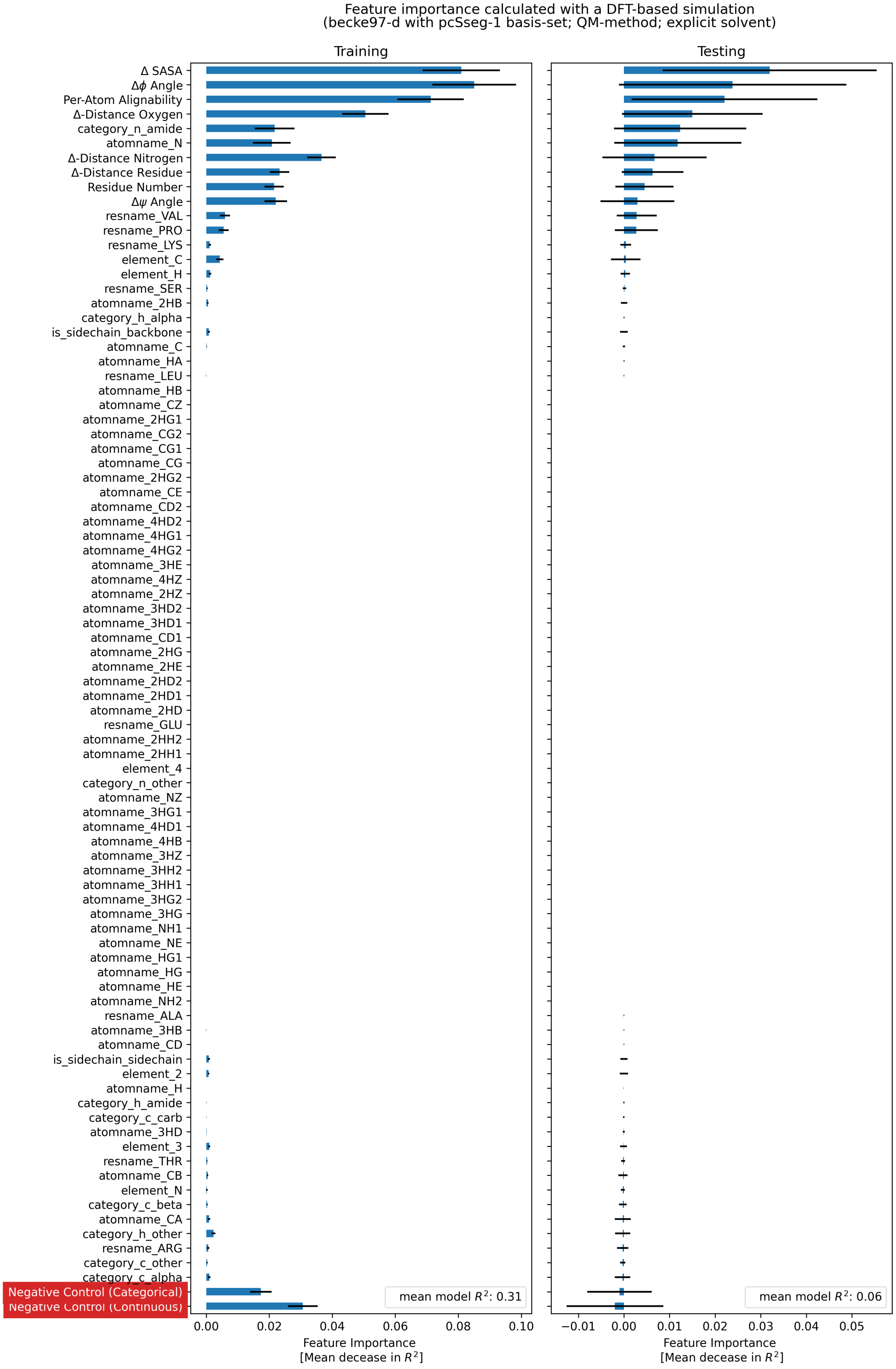

Figure S7.25: Feature importances (onehot) calculated with the DFT-based QM method using becke97-d/pcSseg-1 theory with explicit solvent.

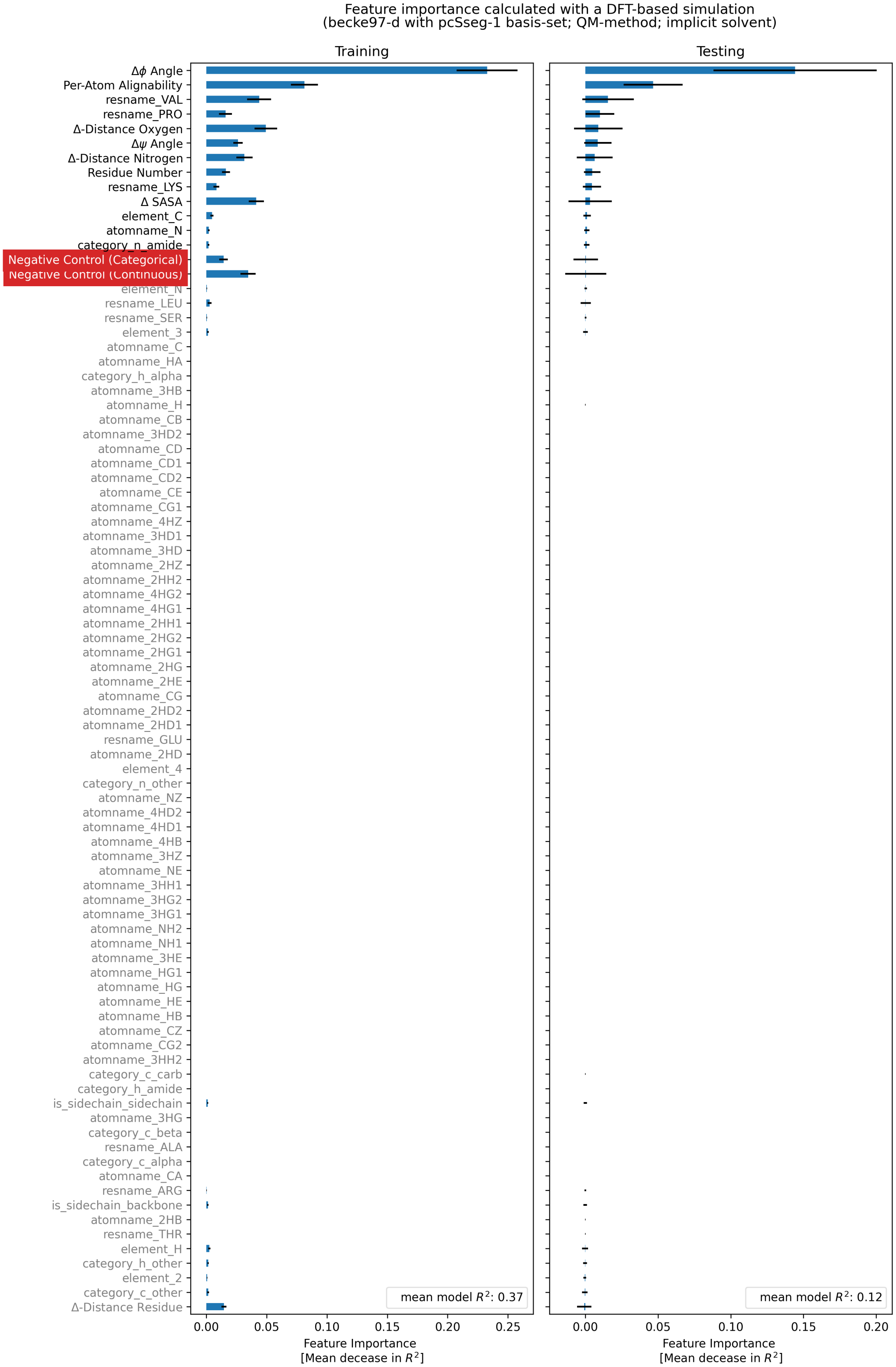

Figure S7.26: Feature importances (onehot) calculated with the DFT-based QM method using becke97-d/pcSseg-1 theory with implicit solvent.

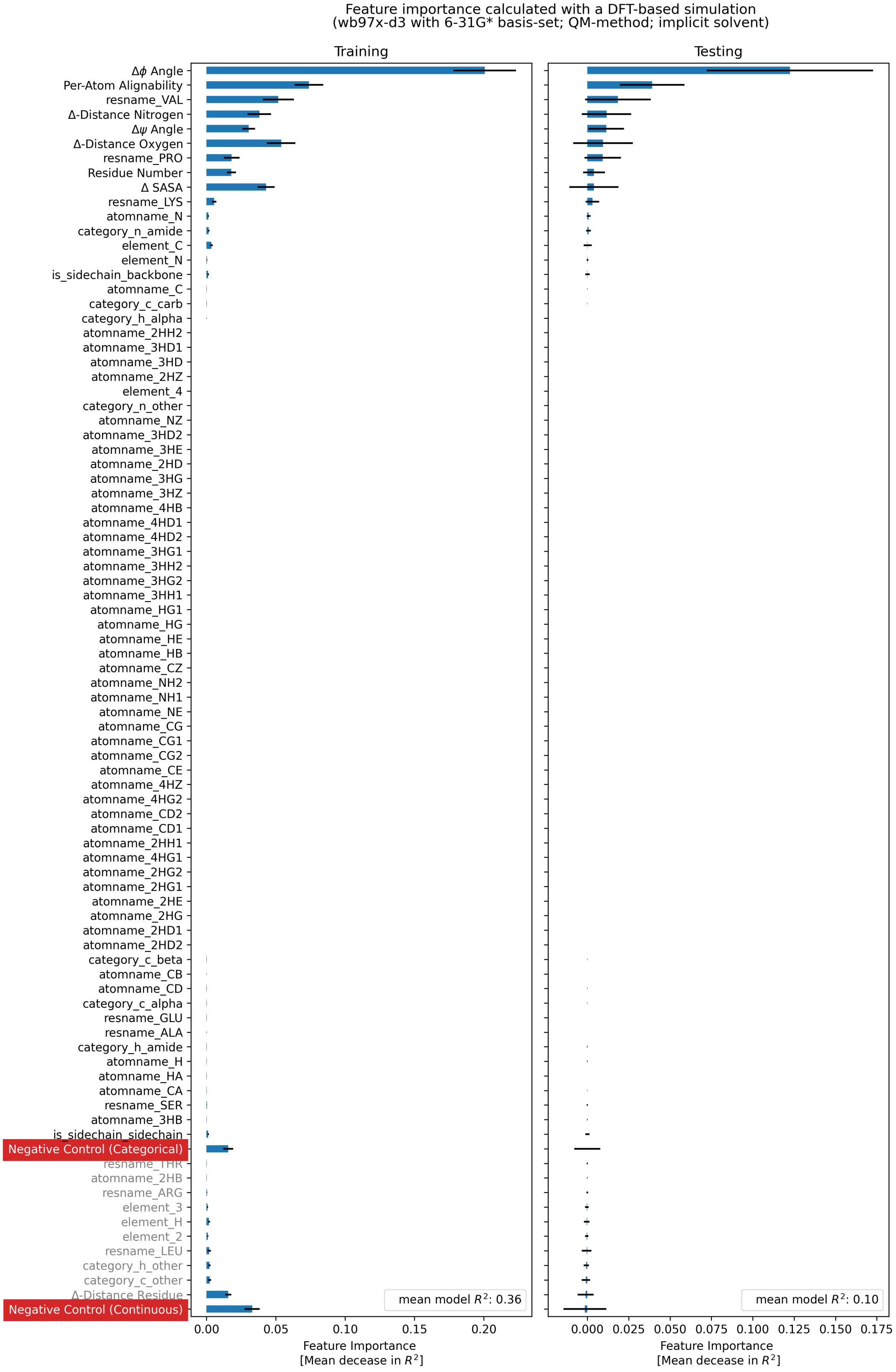

Figure S7.27: Feature importances (onehot) calculated with the DFT-based QM method using wb97x-d3/6-31G* theory with implicit solvent.

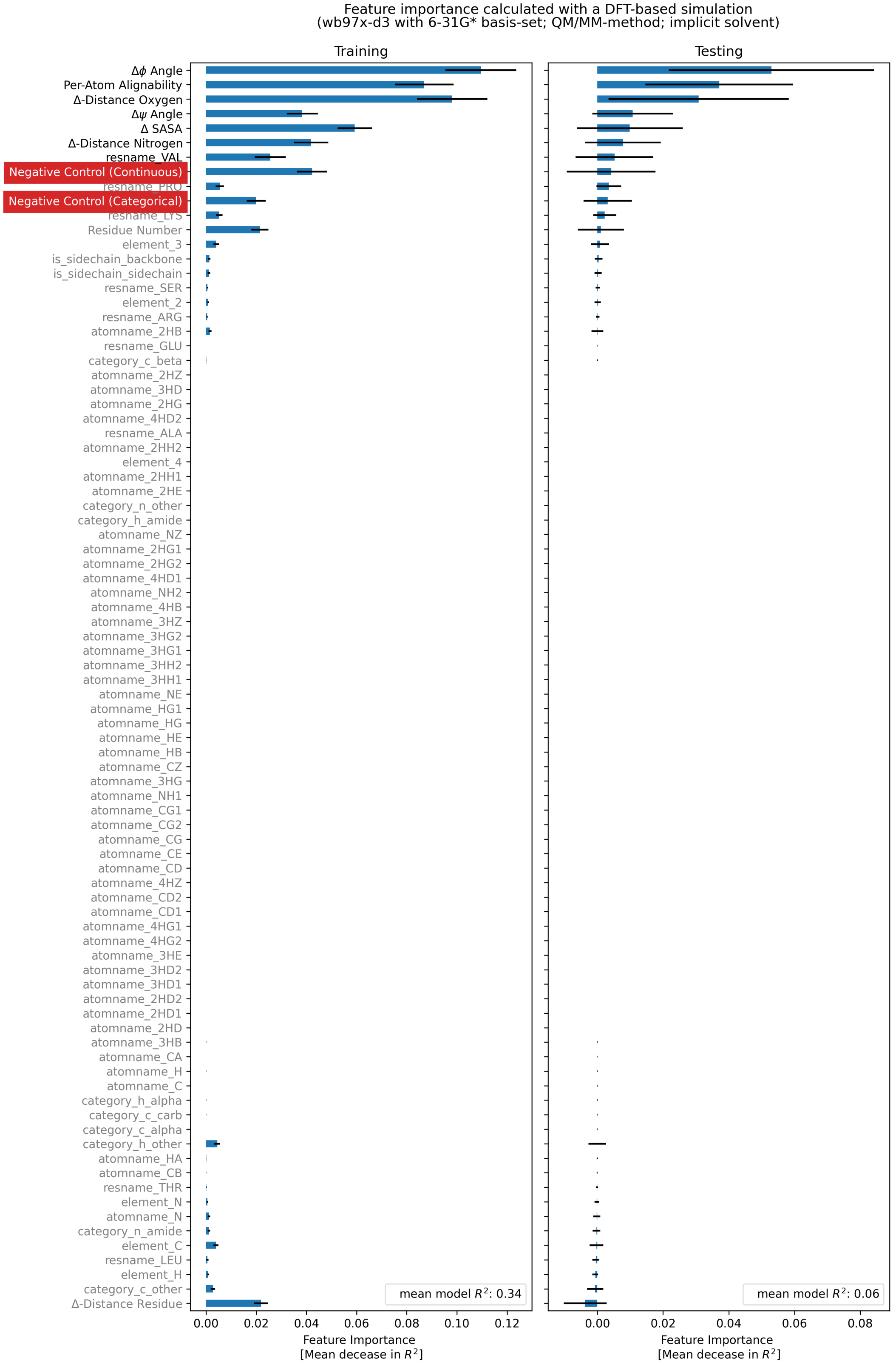

Figure S7.28: Feature importances (onehot) calculated with the DFT-based QM/MM method using wb97x-d3/6-31G* theory with implicit solvent.

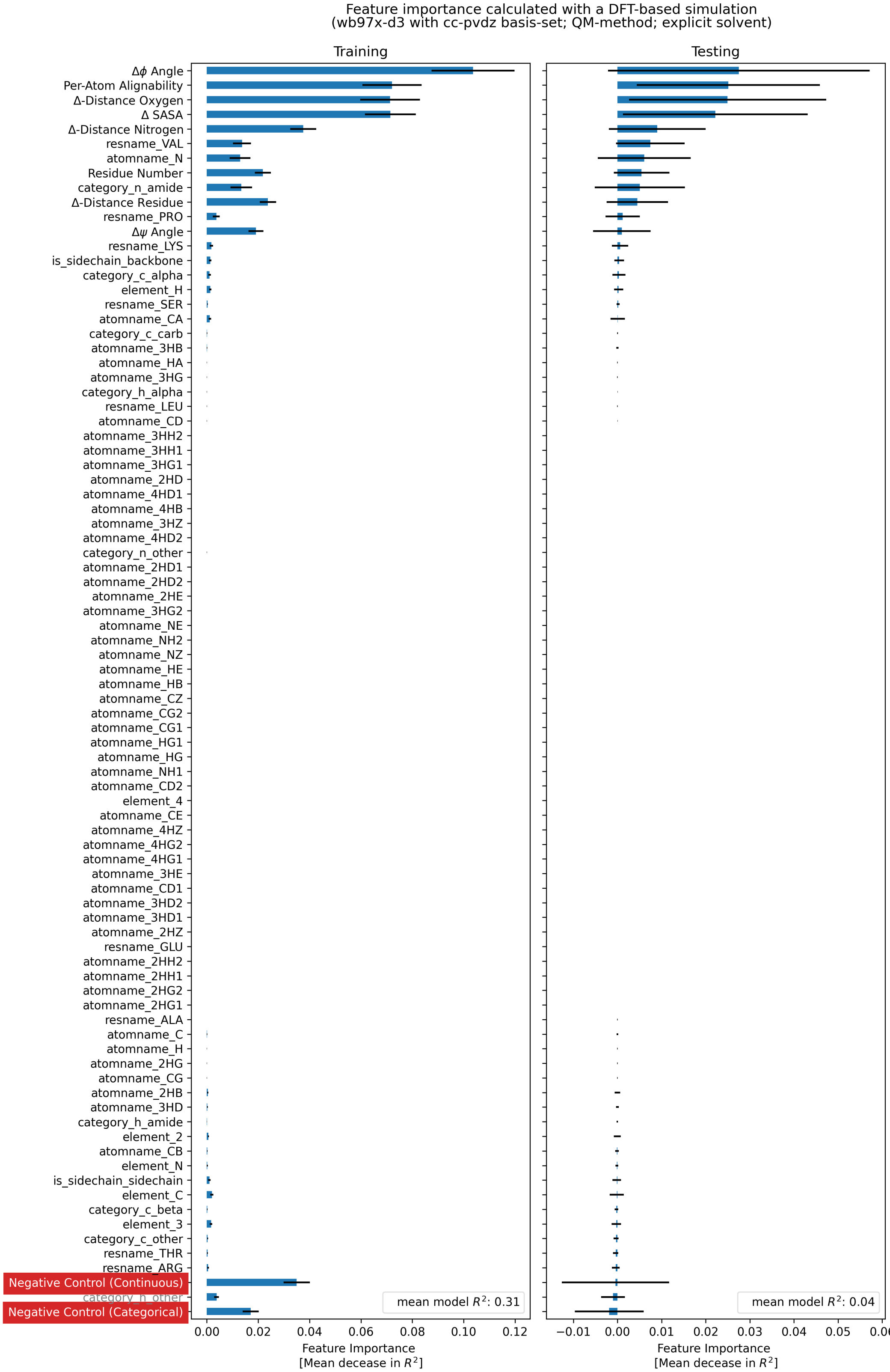

Figure S7.29: Feature importances (onehot) calculated with the DFT-based QM method using wb97x-d3/cc-pvdz theory with explicit solvent.

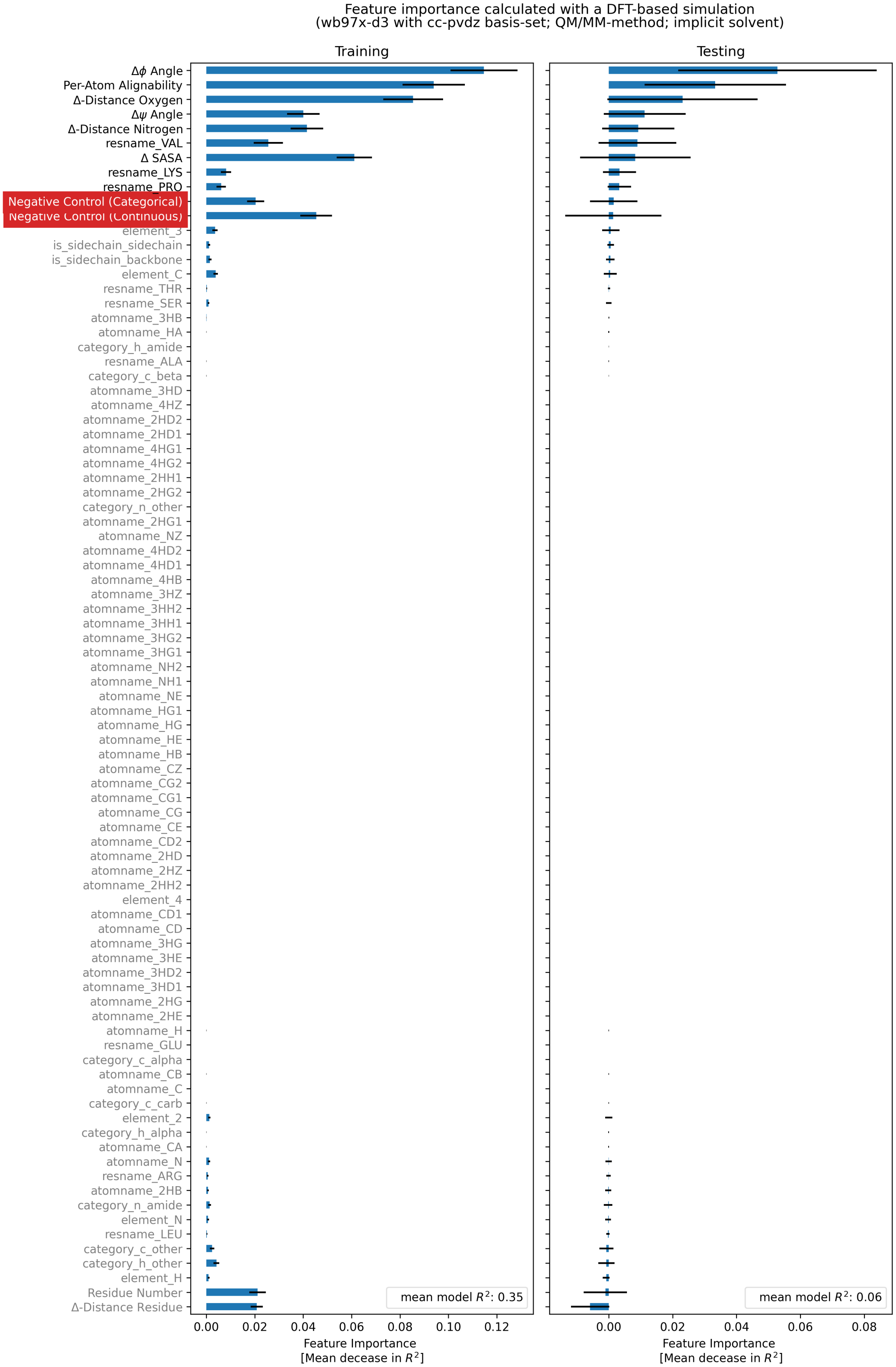

Figure S7.30: Feature importances (onehot) calculated with the DFT-based QM/MM method using wb97x-d3/cc-pvdz theory with implicit solvent.

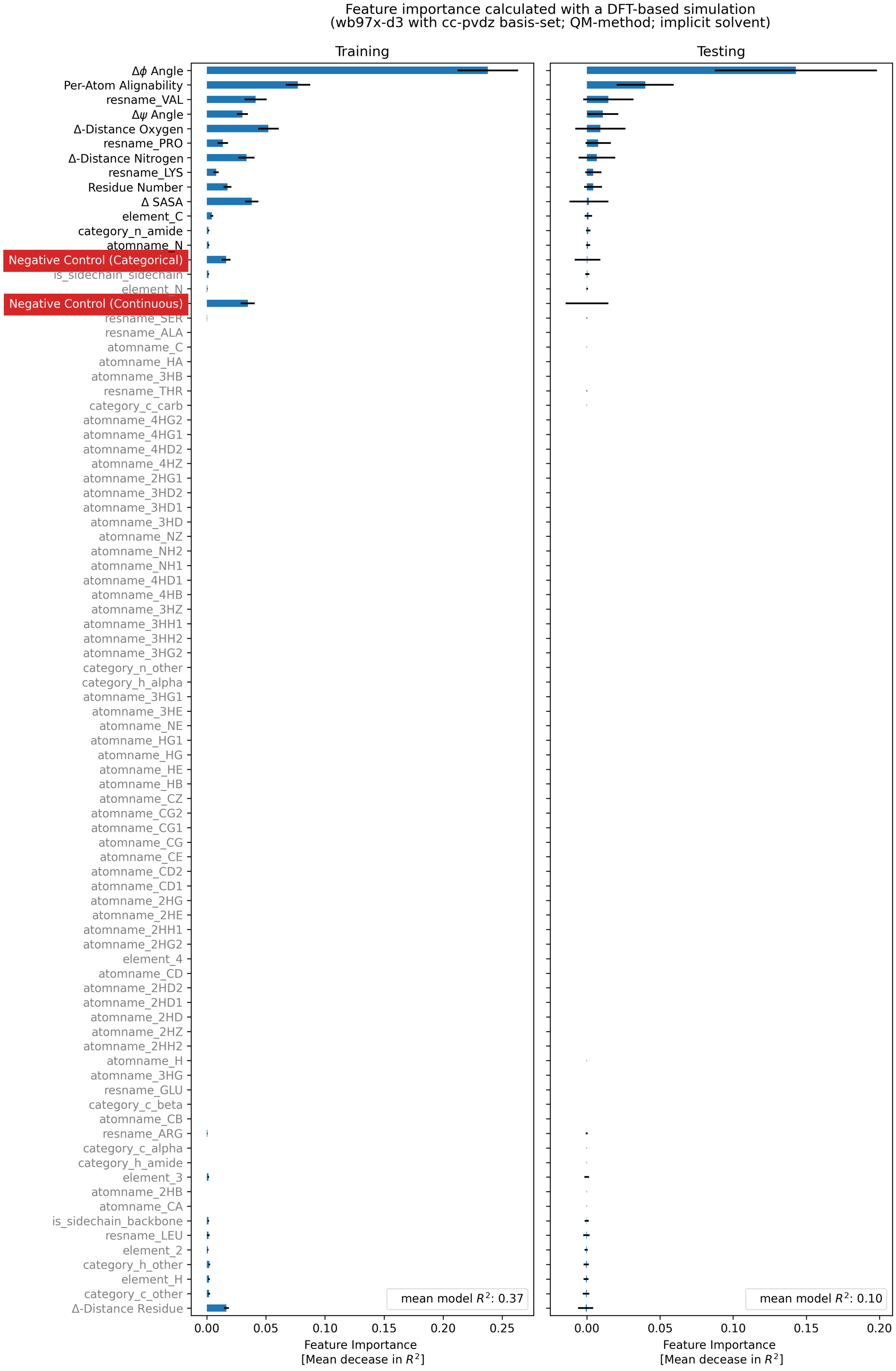

Figure S7.31: Feature importances (onehot) calculated with the DFT-based QM method using wb97x-d3/cc-pvdz theory with implicit solvent.

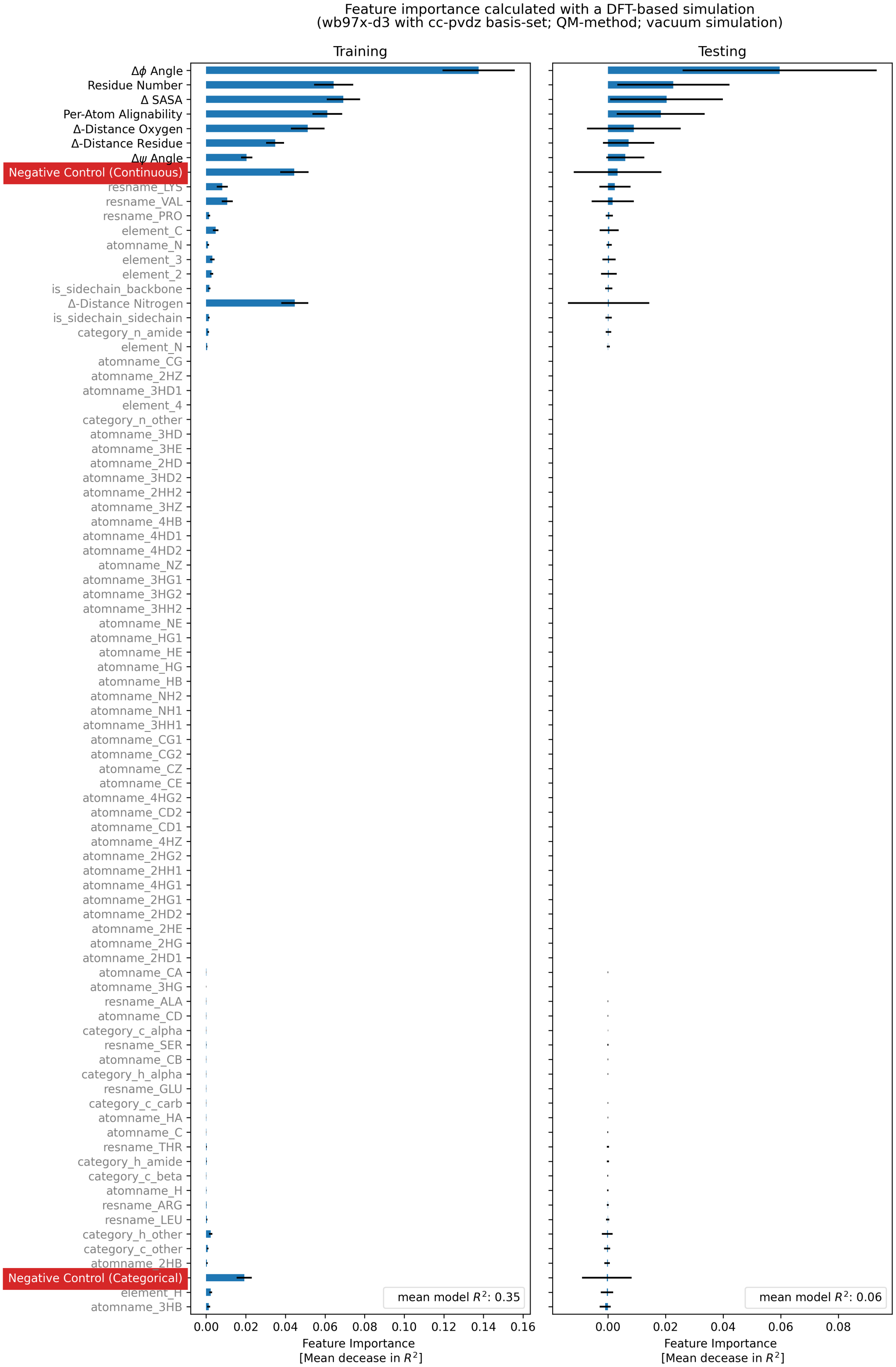

Figure S7.32: Feature importances (onehot) calculated with the DFT-based QM method using wb97x-d3/cc-pvdz theory in vacuum.

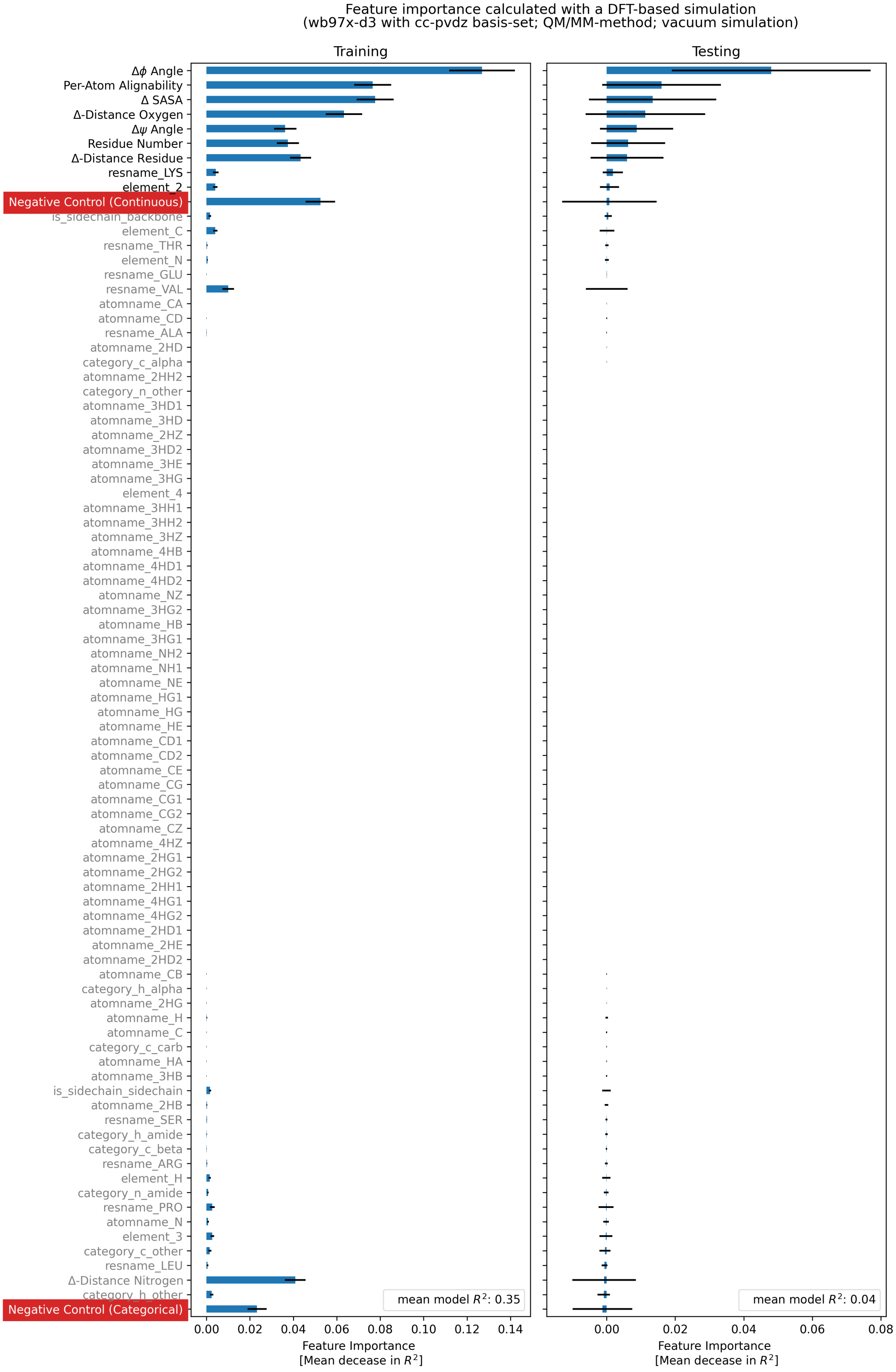

Figure S7.33: Feature importances (onehot) calculated with the DFT-based QM/MM method using wb97x-d3/cc-pvdz theory in vacuum.

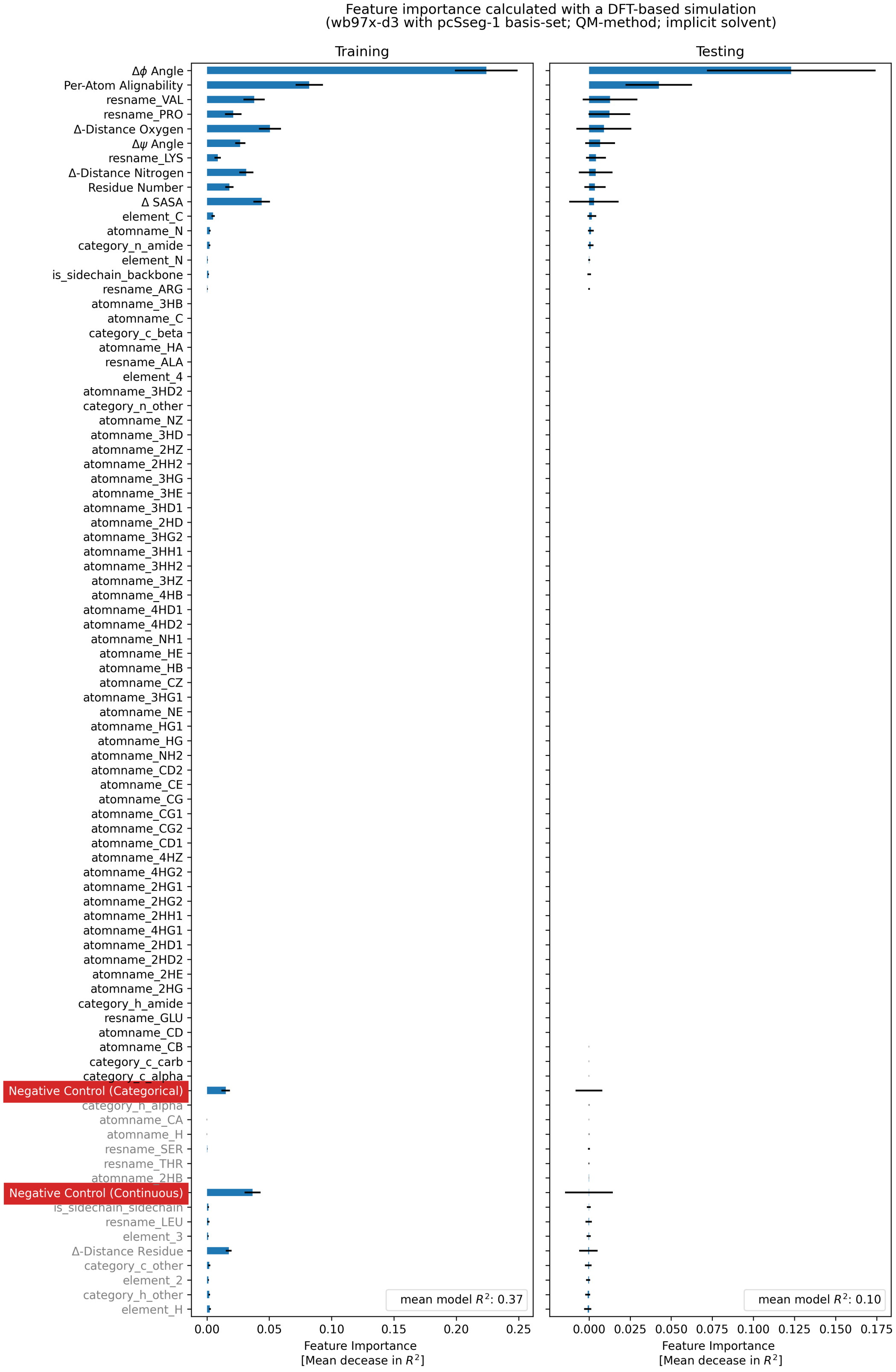

Figure S7.34: Feature importances (onehot) calculated with the DFT-based QM method using wb97x-d3/pcSseg-1 theory with implicit solvent.

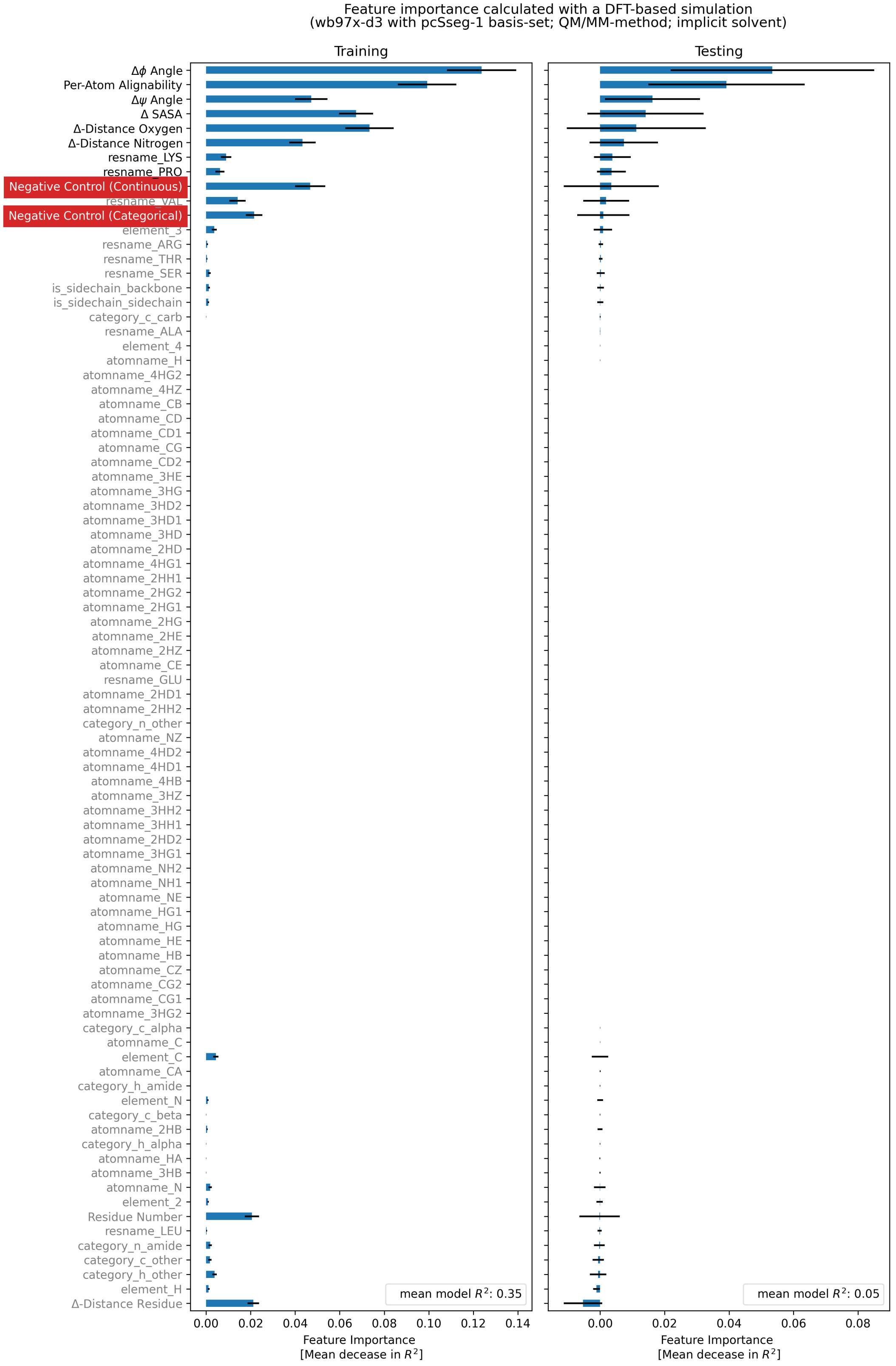

Figure S7.35: Feature importances (onehot) calculated with the DFT-based QM/MM method using wb97x-d3/pcSseg-1 theory with implicit solvent.

1.8 Comparison Simulation and Experiment

The following figures show the comparison of experimental and simulated chemical shifts. The experimental values were obtained from Lasorsa et al..[1,2] For each experimental value, there are two simulated values - one blue dot for the stretched conformation and one red dot for the globular one. Each dot represents the average chemical shifts of the five equal stretched or globular conformations.

A table of content to find each graphic can be found in table 5.

Table 5: Table of content for comparison of experiment and simulation.

Name	Type	Solvation	Figure	Page
PPM	empirical	vacuum	S8.1	136
UCBShiftX	empirical	vacuum	S8.2	137
shiftX2	empirical	vacuum	S8.3	138
sparta+	empirical	vacuum	S8.4	139
b3lyp/6-31G*	QM/MM	implicit	S8.6	141
b3lyp/6-31G*	QM	implicit	S8.6	141
b3lyp/cc-pvdz	QM/MM	vacuum	S8.10	145
b3lyp/cc-pvdz	QM	explicit	S8.8	143
b3lyp/cc-pvdz	QM/MM	implicit	S8.11	146
b3lyp/cc-pvdz	QM	vacuum	S8.10	145
b3lyp/cc-pvdz	QM	implicit	S8.11	146
b3lyp/pcSseg-1	QM	implicit	S8.13	148
b3lyp/pcSseg-1	QM/MM	implicit	S8.13	148
becke97-2/6-31G*	QM/MM	implicit	S8.15	150
becke97-2/6-31G*	QM	implicit	S8.15	150
becke97-2/cc-pvdz	QM	implicit	S8.17	152
becke97-2/cc-pvdz	QM/MM	implicit	S8.17	152
becke97-2/pcSseg-1	QM	implicit	S8.19	154
becke97-2/pcSseg-1	QM/MM	implicit	S8.19	154
becke97-d/6-31G*	QM	implicit	S8.21	156
becke97-d/6-31G*	QM/MM	implicit	S8.21	156
becke97-d/cc-pvdz	QM/MM	implicit	S8.23	158
becke97-d/cc-pvdz	QM	implicit	S8.23	158
becke97-d/pcSseg-1	QM/MM	implicit	S8.26	161
becke97-d/pcSseg-1	QM	explicit	S8.25	160
becke97-d/pcSseg-1	QM	implicit	S8.26	161
wb97x-d3/6-31G*	QM	implicit	S8.28	163
wb97x-d3/6-31G*	QM/MM	implicit	S8.28	163
wb97x-d3/cc-pvdz	QM	explicit	S8.29	164
wb97x-d3/cc-pvdz	QM/MM	implicit	S8.31	166
wb97x-d3/cc-pvdz	QM	implicit	S8.31	166
wb97x-d3/cc-pvdz	QM	vacuum	S8.33	168
wb97x-d3/cc-pvdz	QM/MM	vacuum	S8.33	168
wb97x-d3/pcSseg-1	QM	implicit	S8.35	170
wb97x-d3/pcSseg-1	QM/MM	implicit	S8.35	170

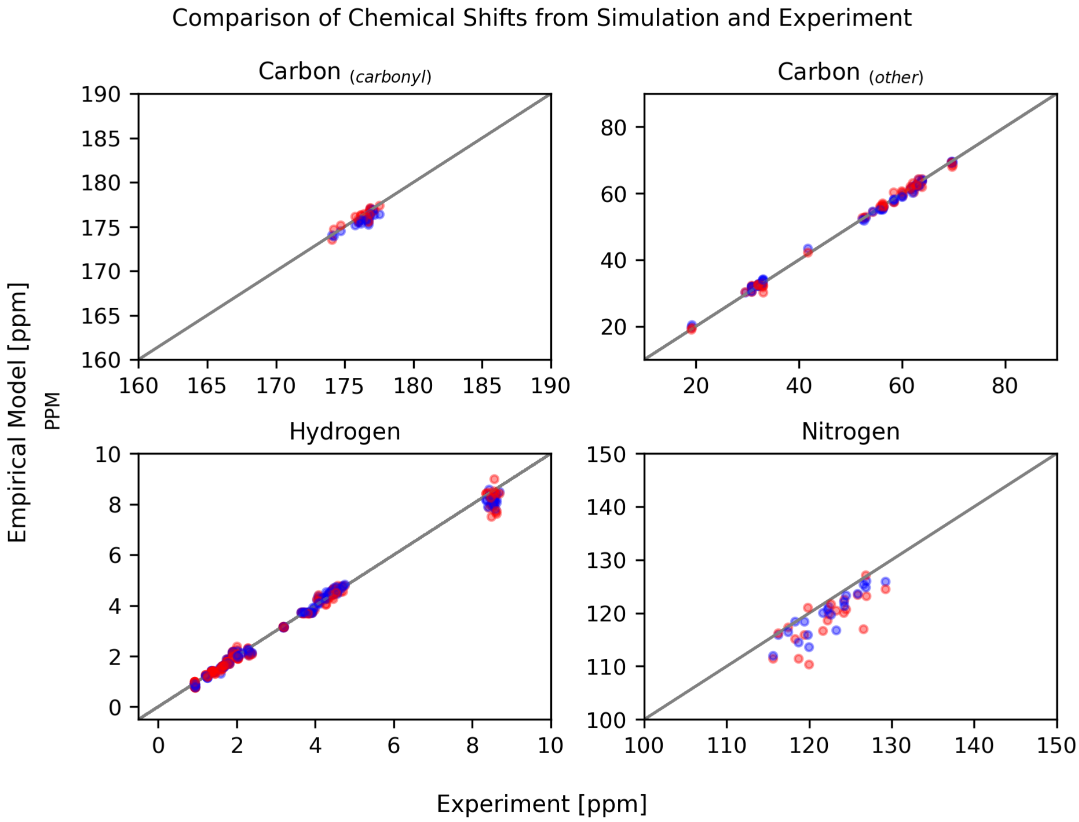

Figure S8.1: Comparison of experimental and simulated chemical shifts calculated with the empirical method PPM.

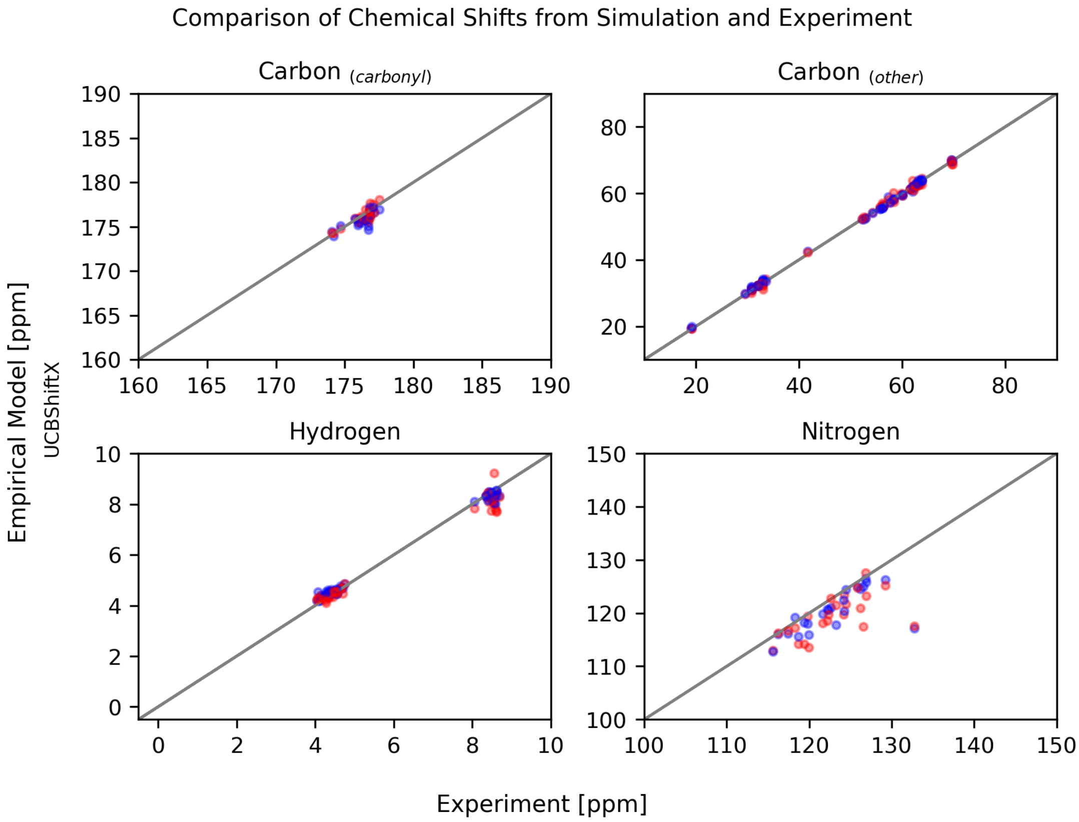

Figure S8.2: Comparison of experimental and simulated chemical shifts calculated with the empirical method UCBShiftX.

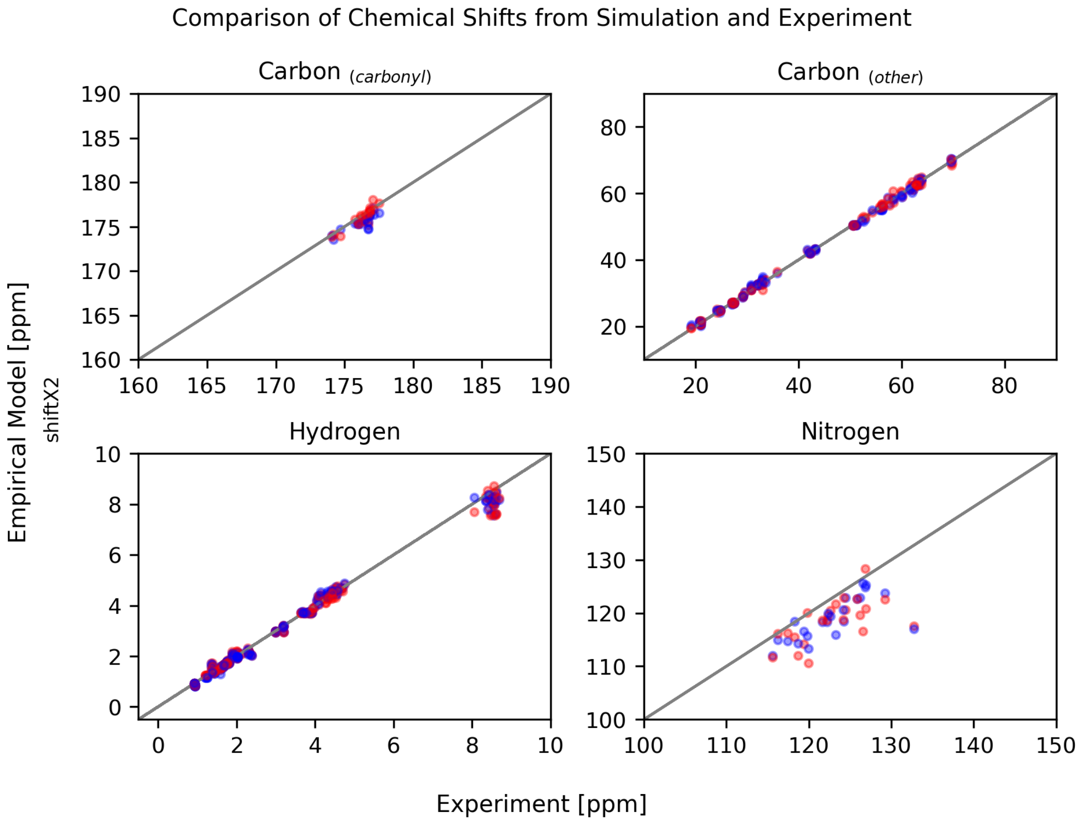

Figure S8.3: Comparison of experimental and simulated chemical shifts calculated with the empirical method shiftX2.

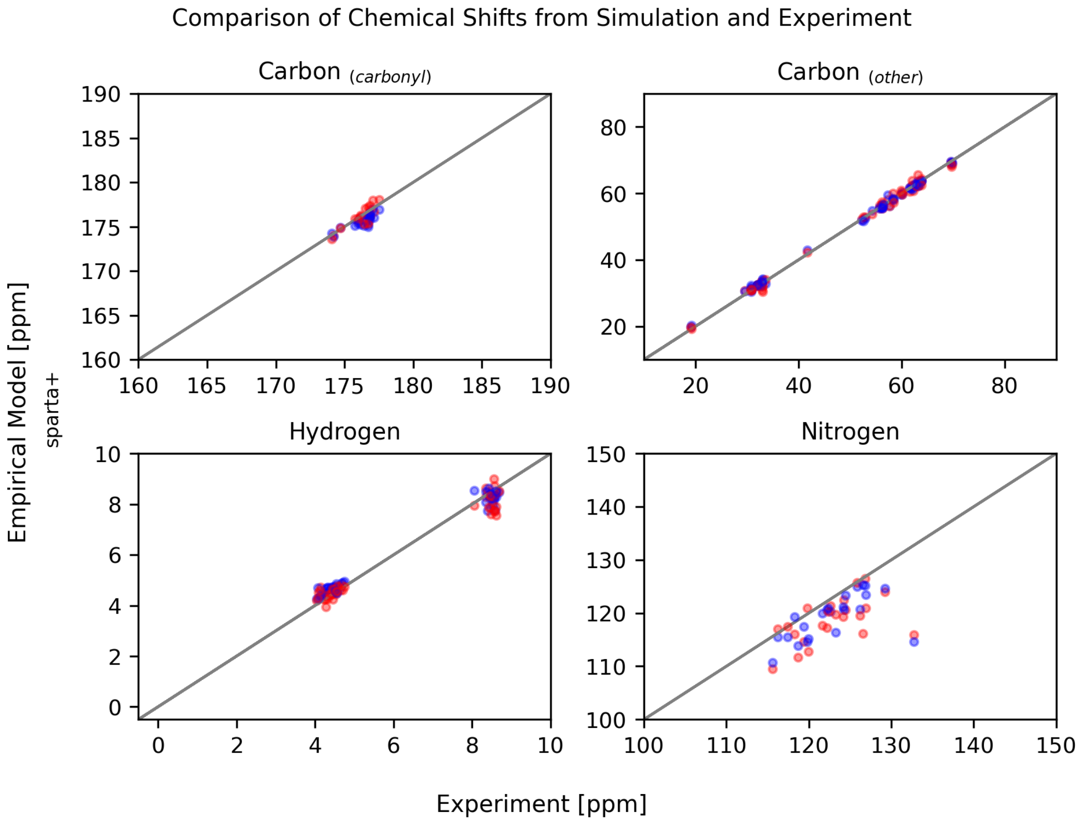

Figure S8.4: Comparison of experimental and simulated chemical shifts calculated with the empirical method sparta+.

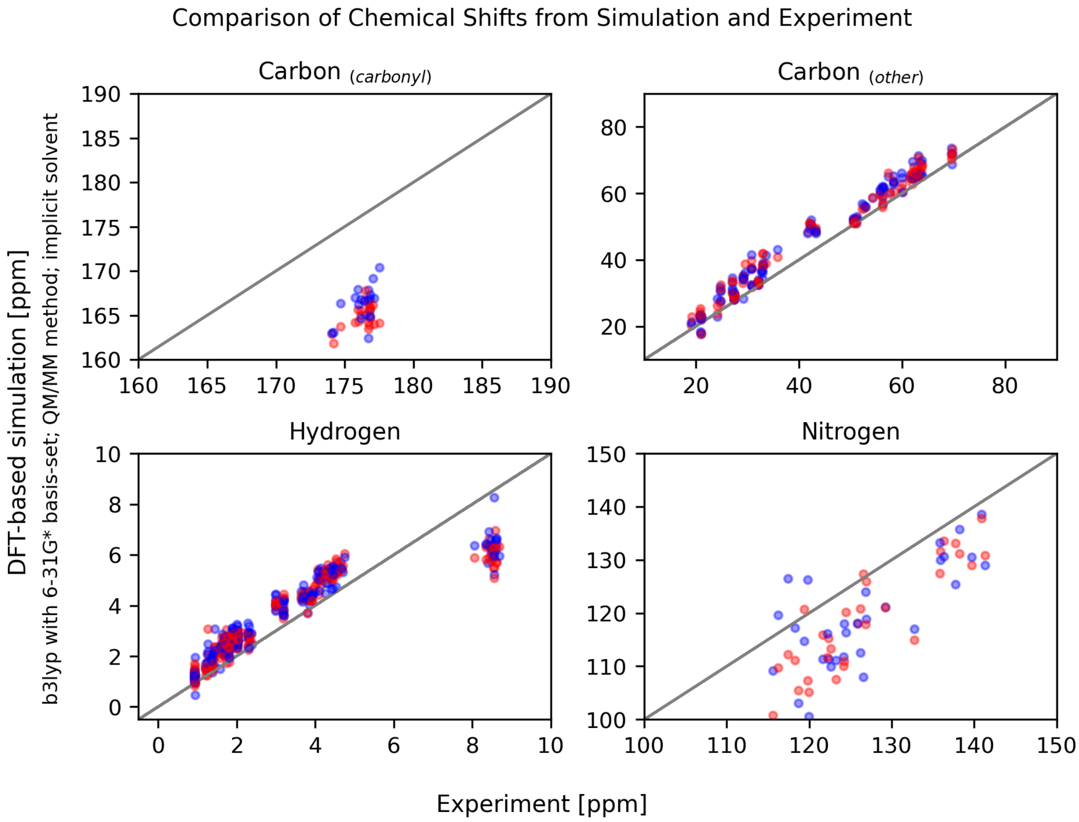

Figure S8.5: Comparison of experimental and simulated chemical shifts calculated with the DFT-based QM/MM method using b3lyp/6-31G* theory with implicit solvent.

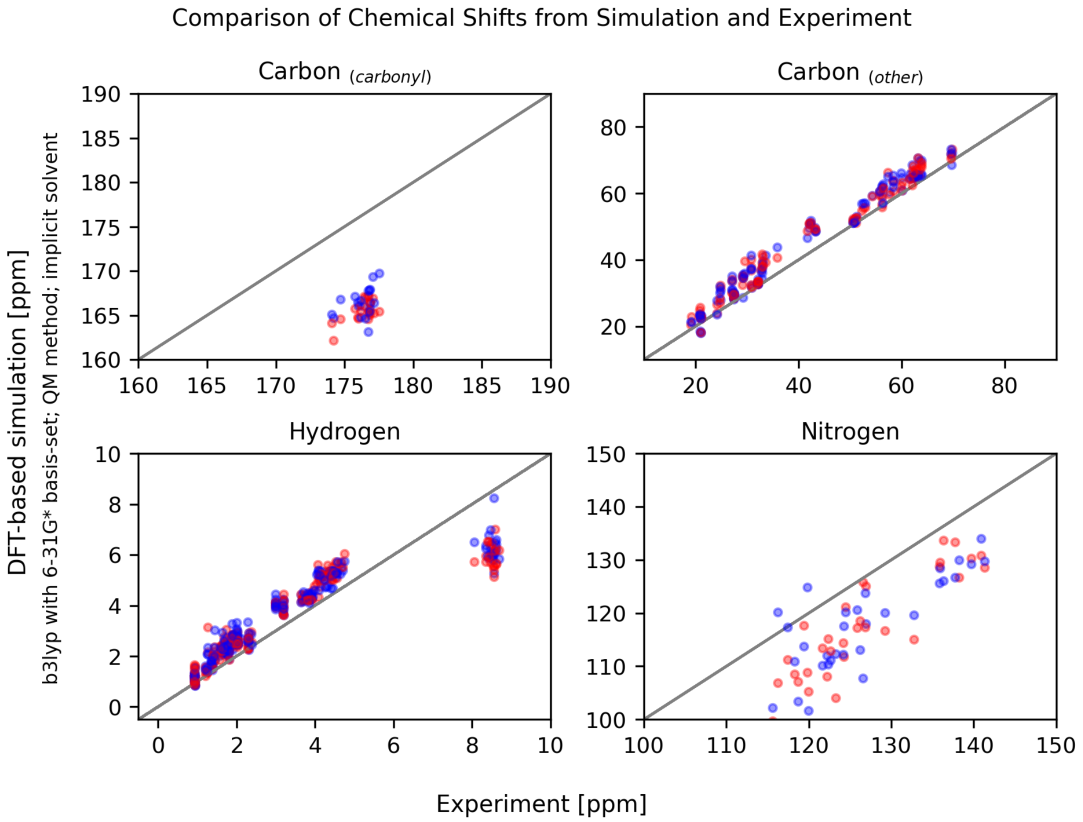

Figure S8.6: Comparison of experimental and simulated chemical shifts calculated with the DFT-based QM method using b3lyp/6-31G* theory with implicit solvent.

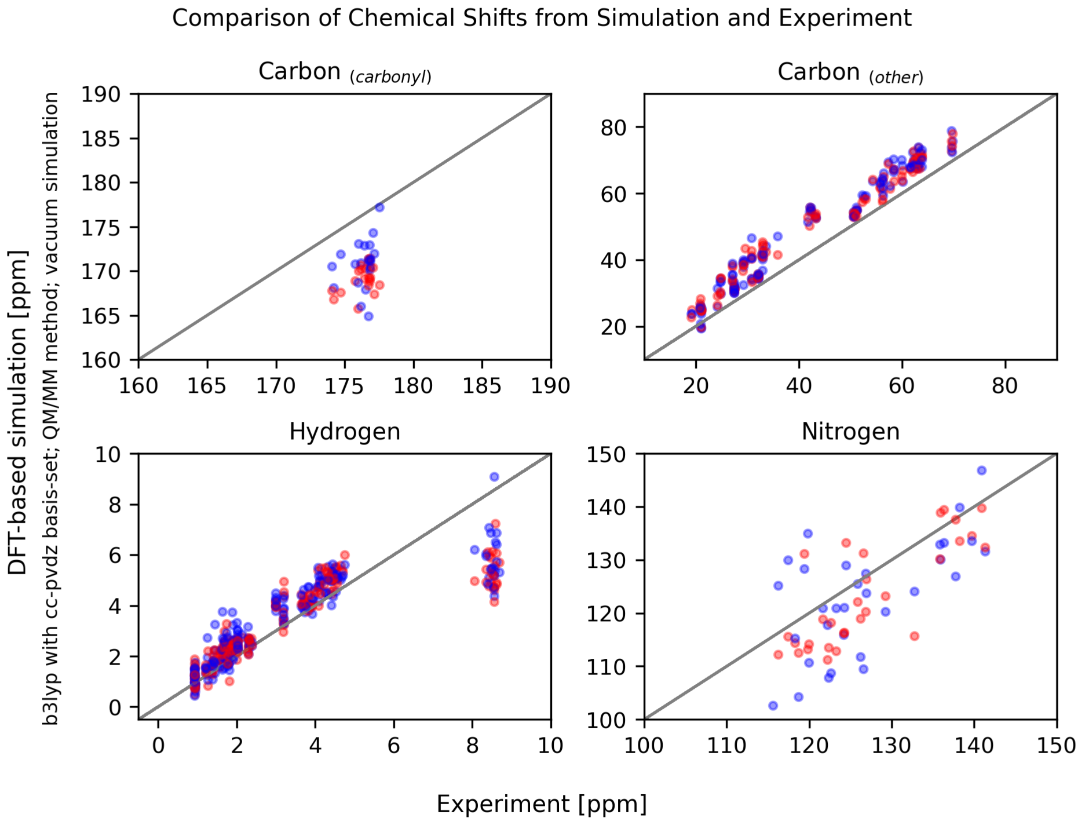

Figure S8.7: Comparison of experimental and simulated chemical shifts calculated with the DFT-based QM/MM method using b3lyp/cc-pvdz theory in vacuum.

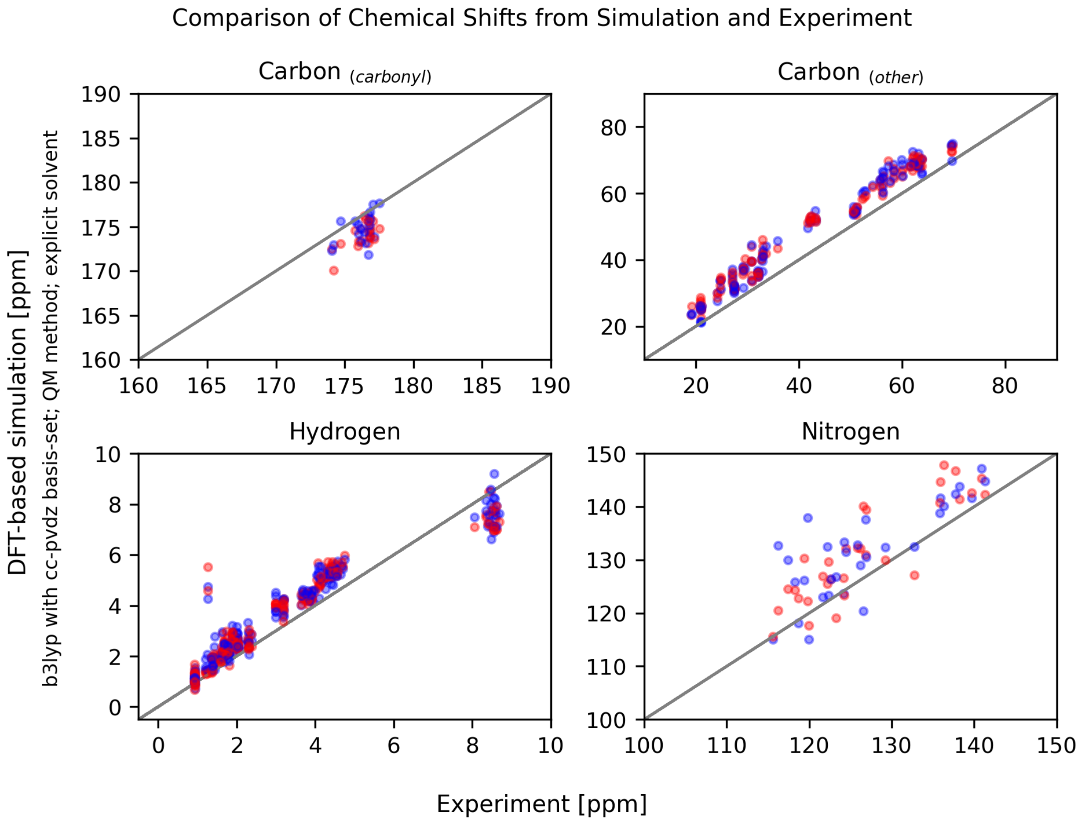

Figure S8.8: Comparison of experimental and simulated chemical shifts calculated with the DFT-based QM method using b3lyp/cc-pvdz theory with explicit solvent.

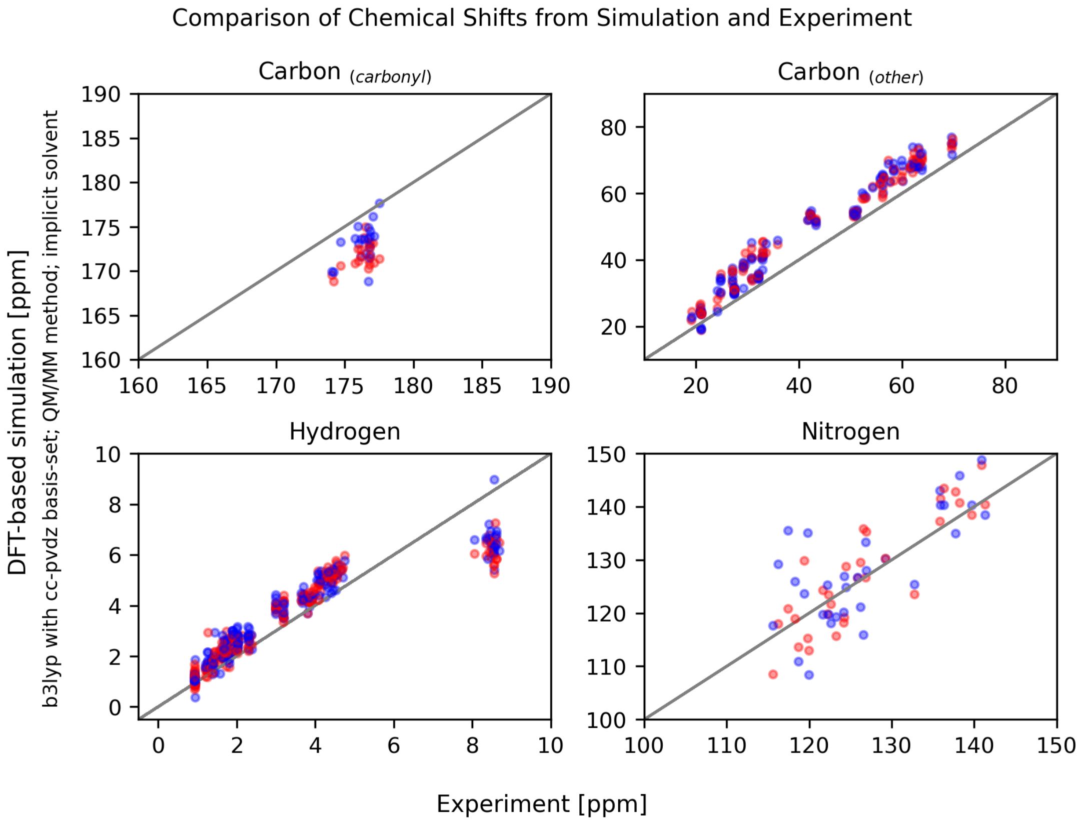

Figure S8.9: Comparison of experimental and simulated chemical shifts calculated with the DFT-based QM/MM method using b3lyp/cc-pvdz theory with implicit solvent.

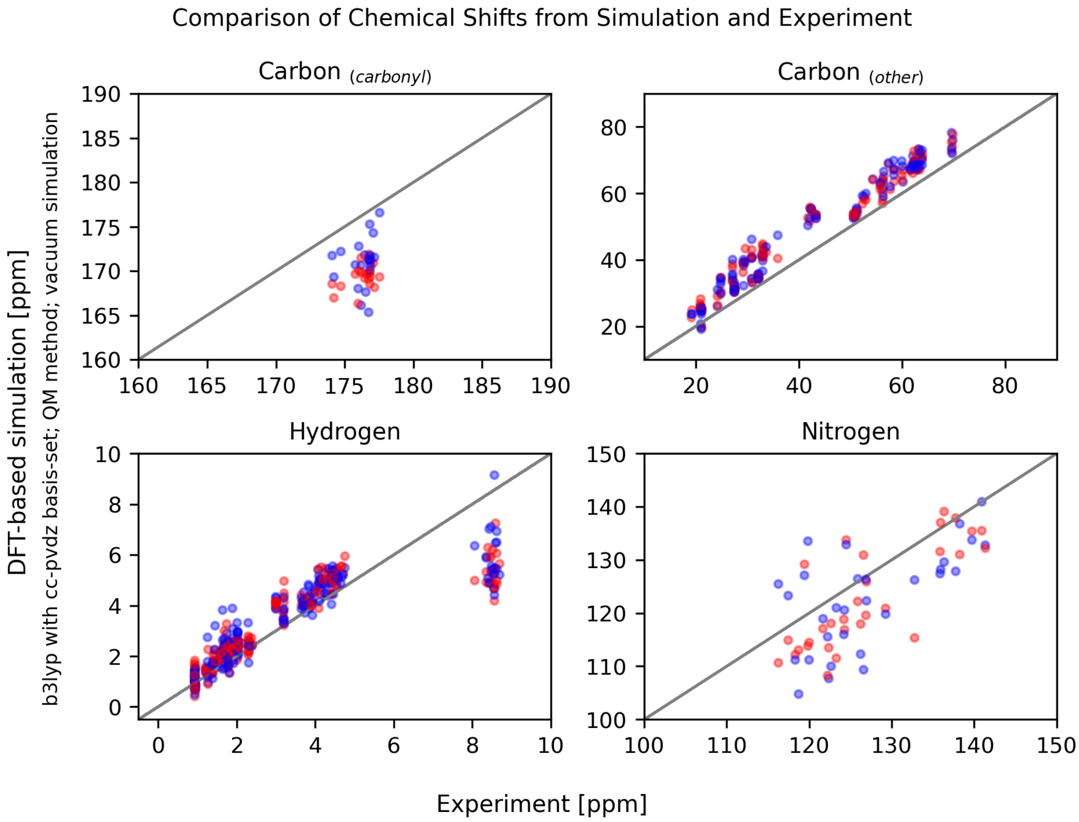

Figure S8.10: Comparison of experimental and simulated chemical shifts calculated with the DFT-based QM method using b3lyp/cc-pvdz theory in vacuum.

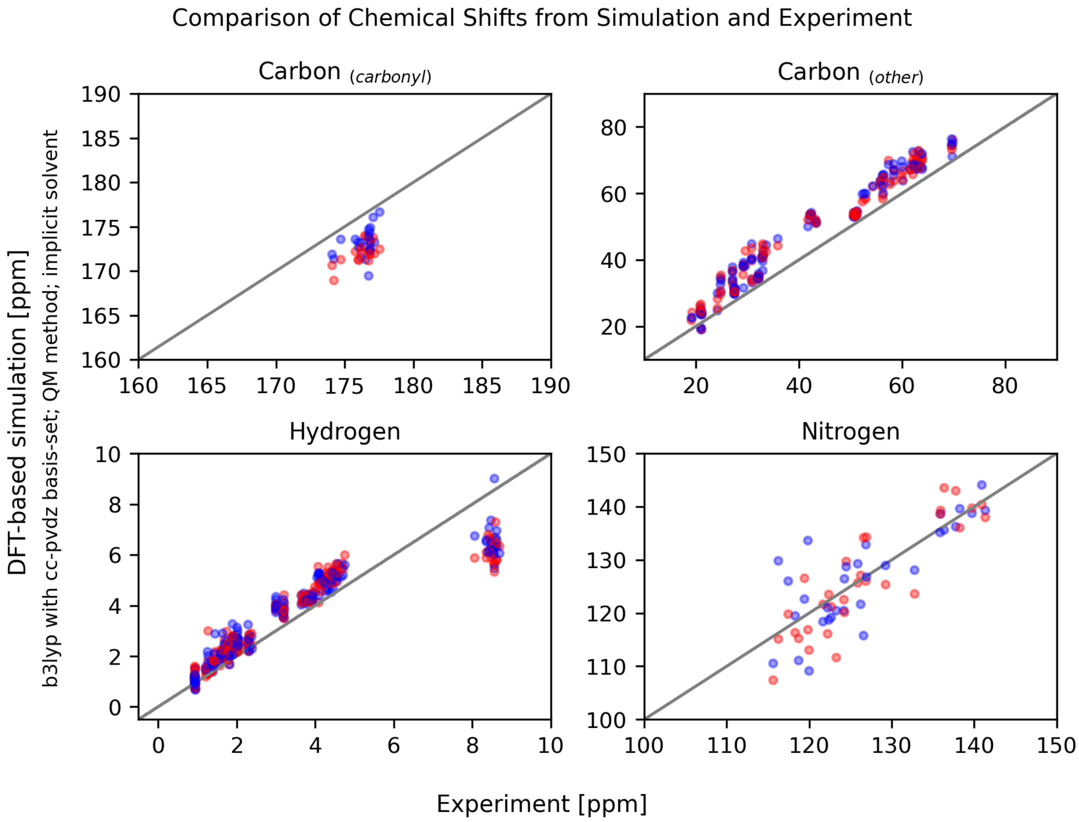

Figure S8.11: Comparison of experimental and simulated chemical shifts calculated with the DFT-based QM method using b3lyp/cc-pvdz theory with implicit solvent.

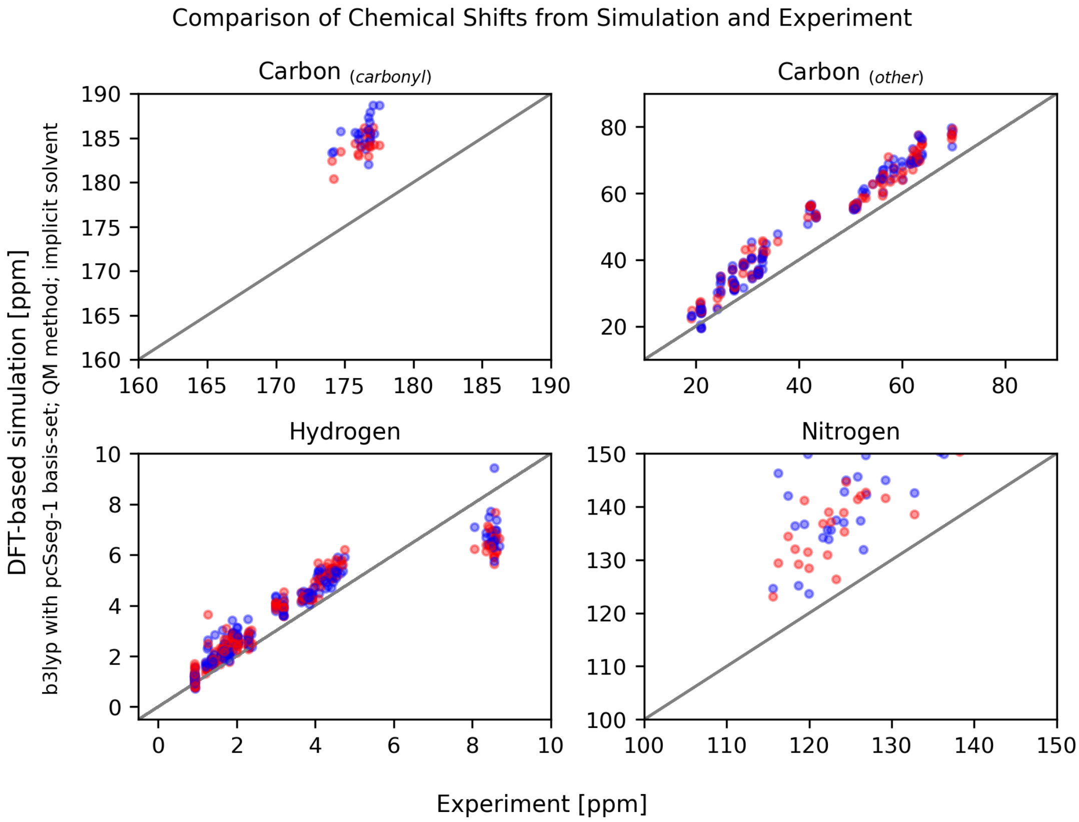

Figure S8.12: Comparison of experimental and simulated chemical shifts calculated with the DFT-based QM method using b3lyp/pcSseg-1 theory with implicit solvent.

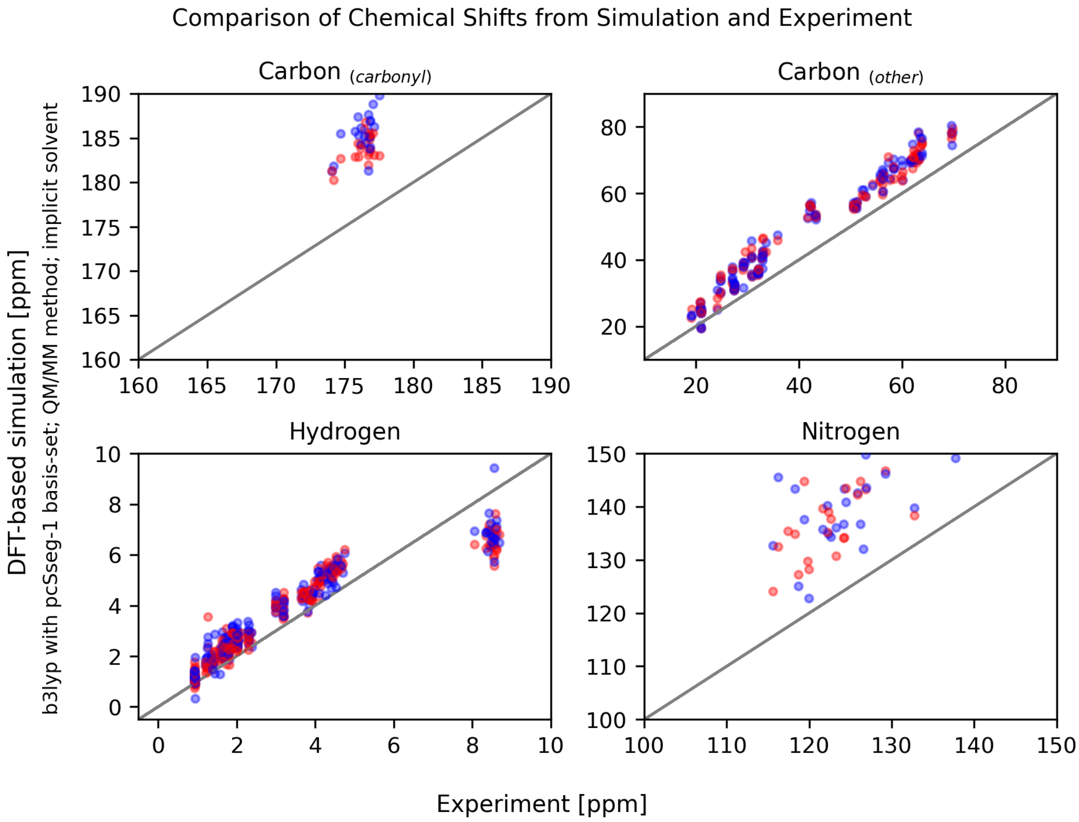

Figure S8.13: Comparison of experimental and simulated chemical shifts calculated with the DFT-based QM/MM method using b3lyp/pcSseg-1 theory with implicit solvent.

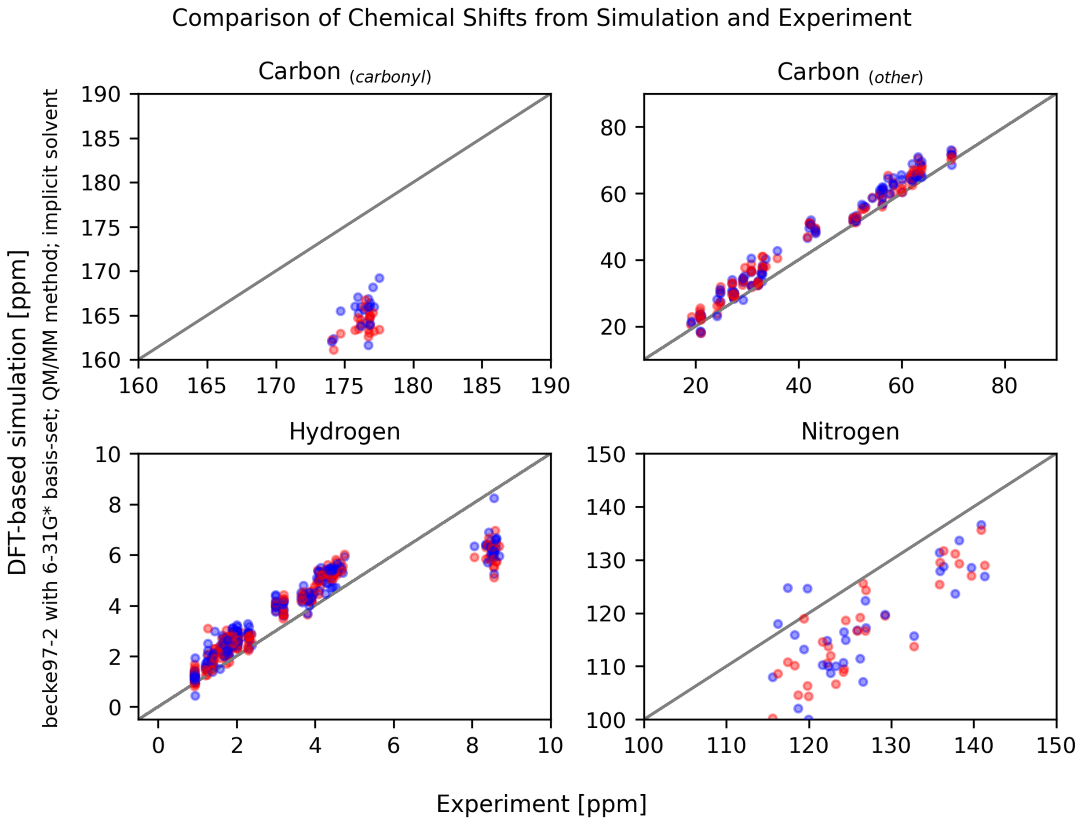

Figure S8.14: Comparison of experimental and simulated chemical shifts calculated with the DFT-based QM/MM method using becke97-2/6-31G* theory with implicit solvent.

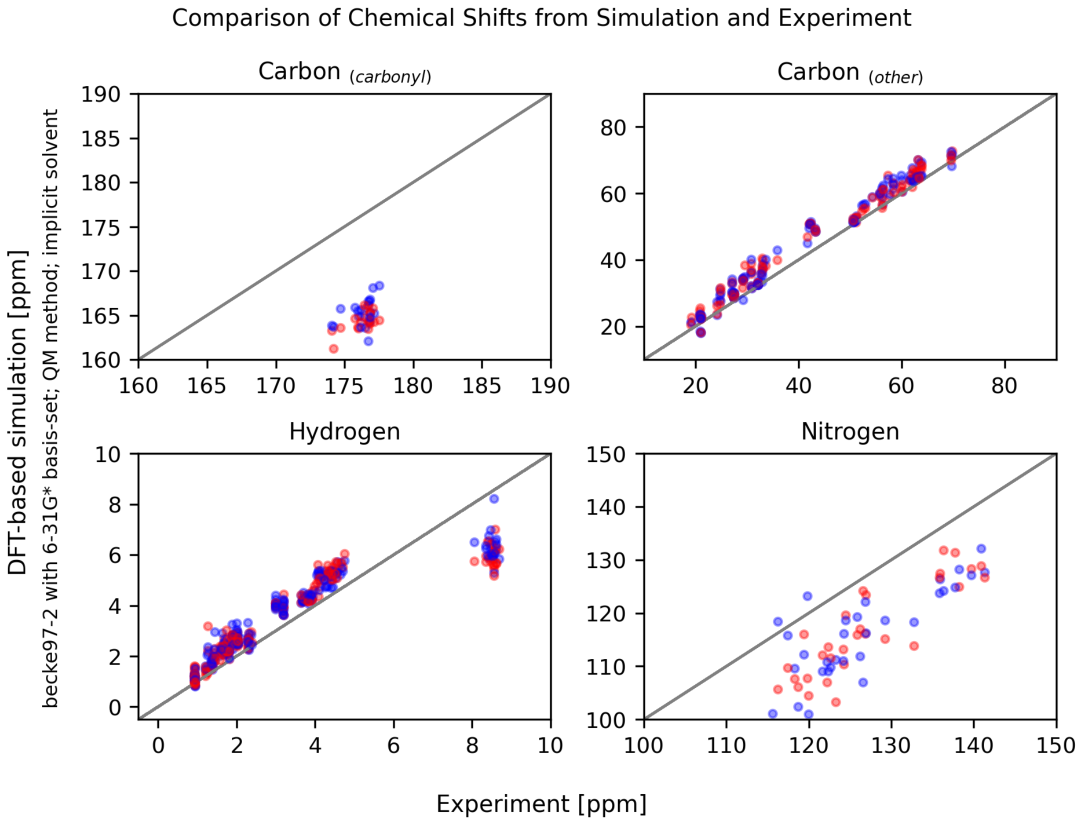

Figure S8.15: Comparison of experimental and simulated chemical shifts calculated with the DFT-based QM method using becke97-2/6-31G* theory with implicit solvent.

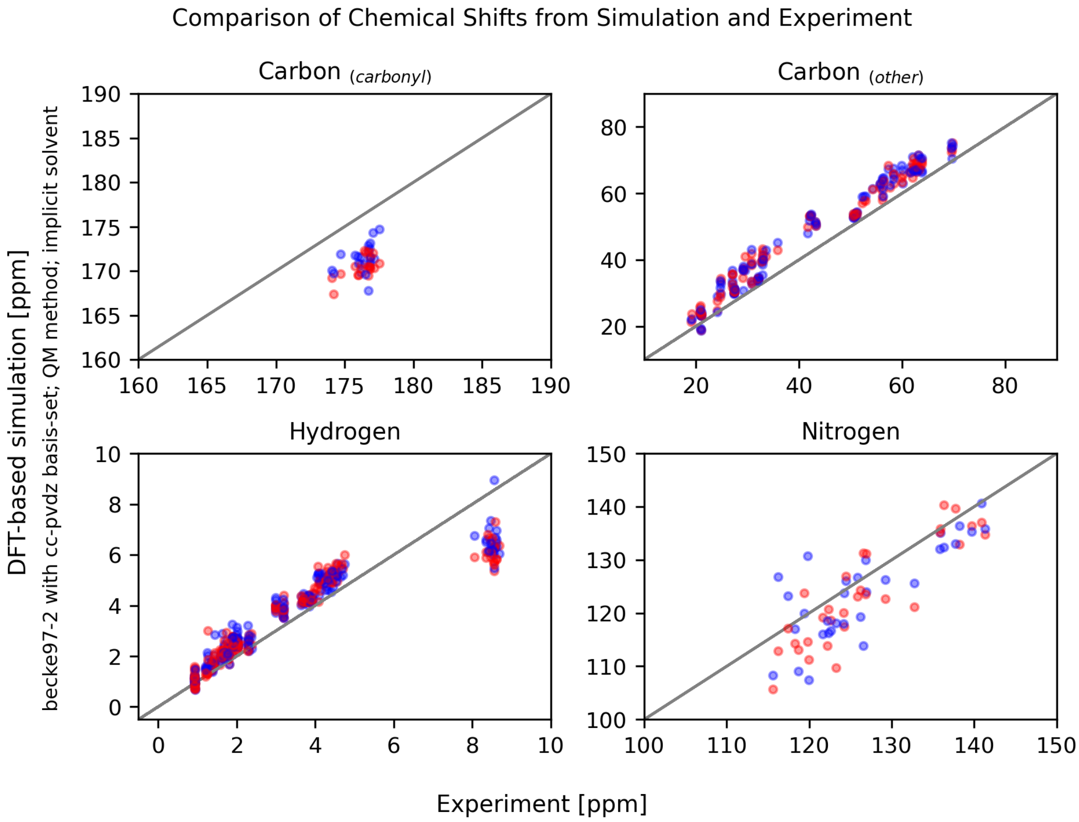

Figure S8.16: Comparison of experimental and simulated chemical shifts calculated with the DFT-based QM method using becke97-2/cc-pvdz theory with implicit solvent.

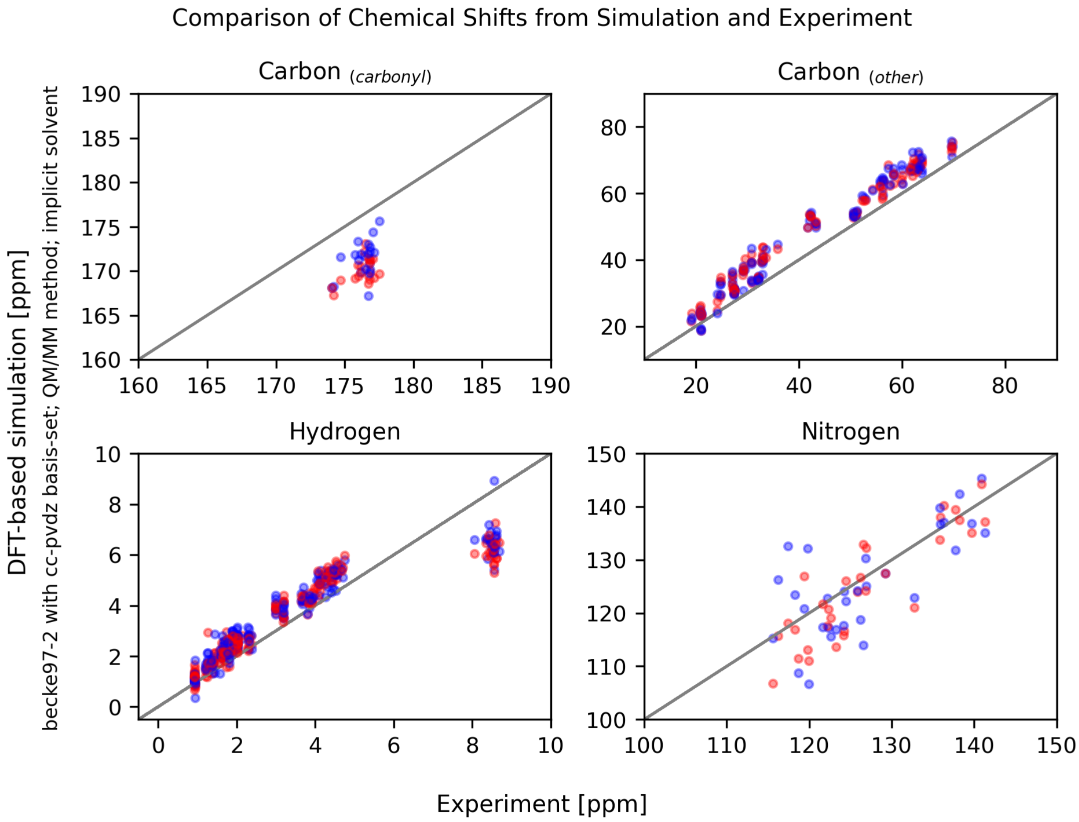

Figure S8.17: Comparison of experimental and simulated chemical shifts calculated with the DFT-based QM/MM method using becke97-2/cc-pvdz theory with implicit solvent.

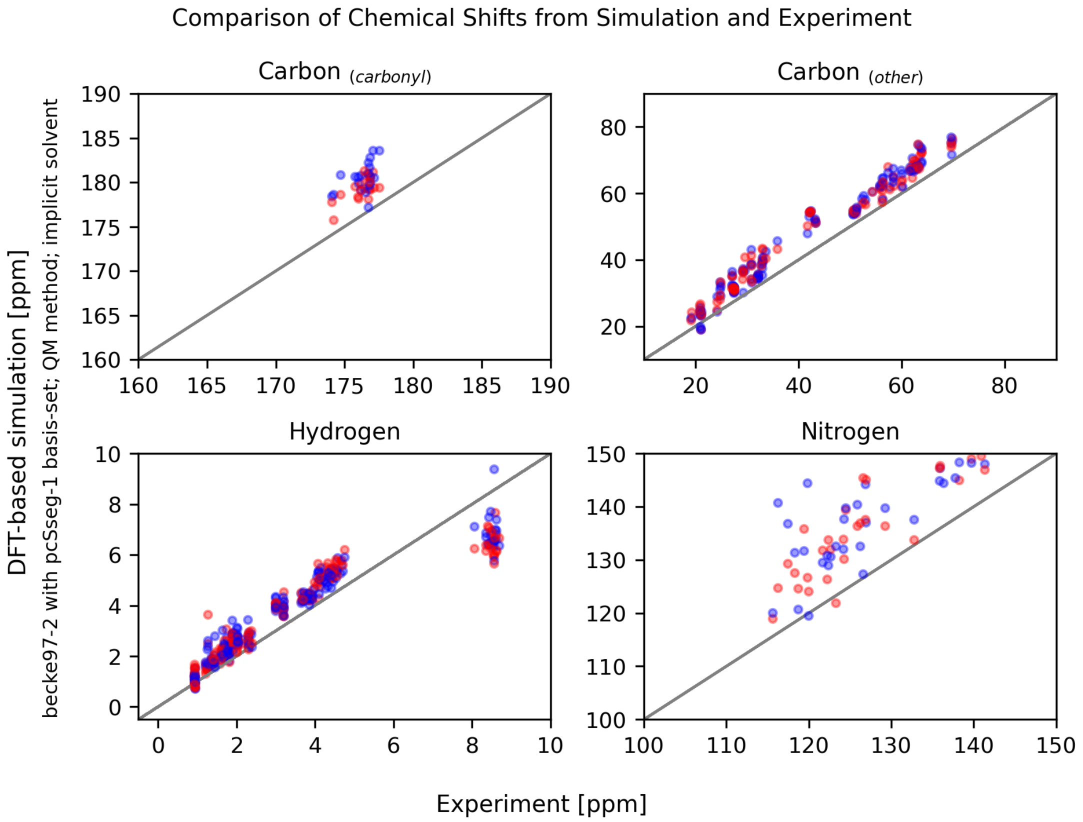

Figure S8.18: Comparison of experimental and simulated chemical shifts calculated with the DFT-based QM method using becke97-2/pcSseg-1 theory with implicit solvent.

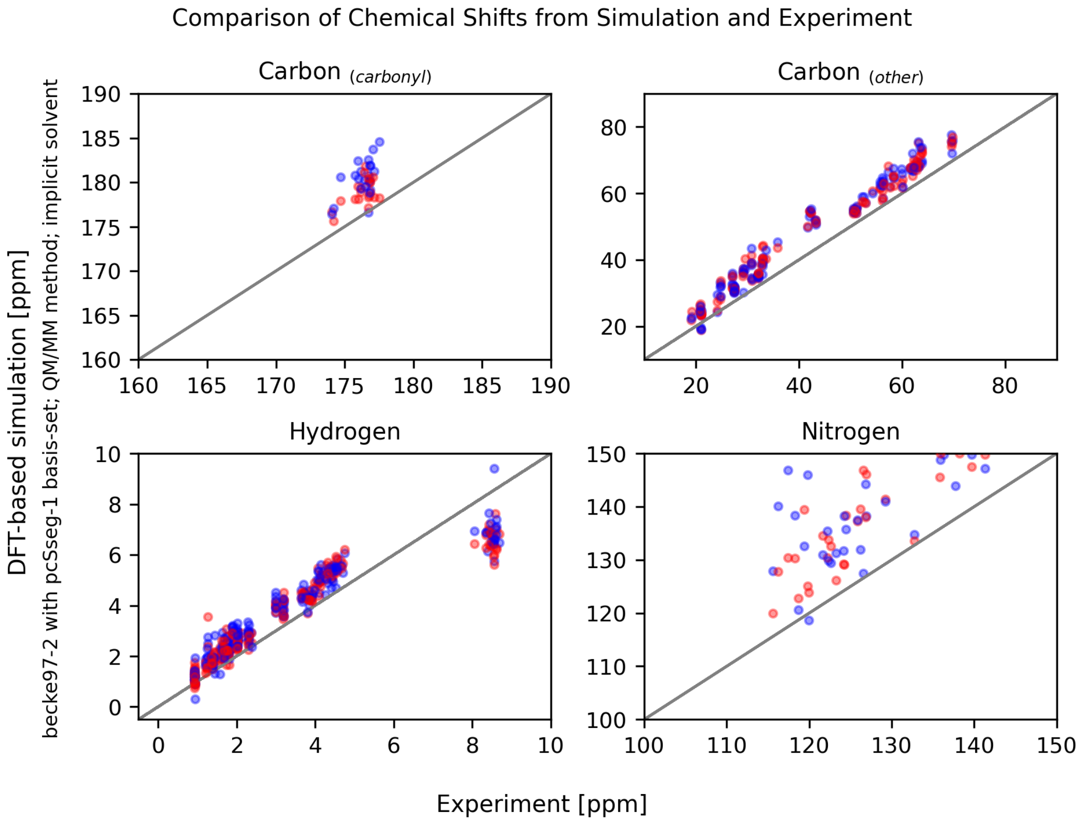

Figure S8.19: Comparison of experimental and simulated chemical shifts calculated with the DFT-based QM/MM method using becke97-2/pcSseg-1 theory with implicit solvent.

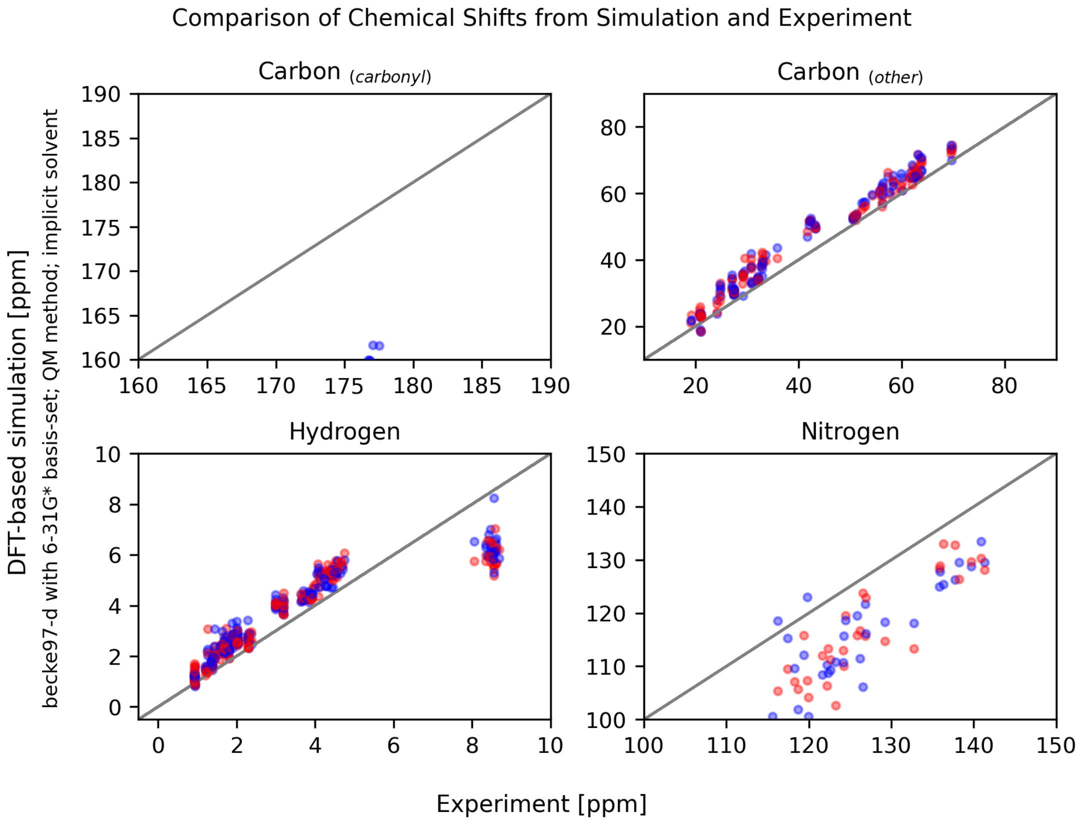

Figure S8.20: Comparison of experimental and simulated chemical shifts calculated with the DFT-based QM method using becke97-d/6-31G* theory with implicit solvent.

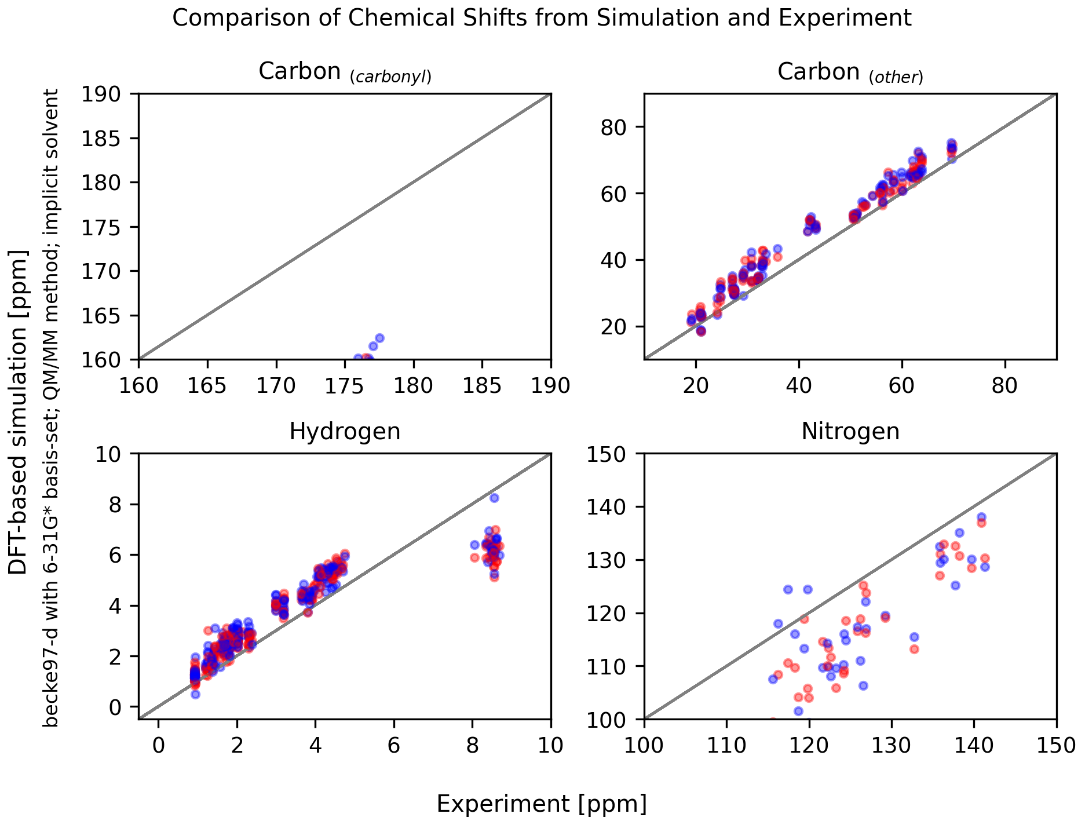

Figure S8.21: Comparison of experimental and simulated chemical shifts calculated with the DFT-based QM/MM method using becke97-d/6-31G* theory with implicit solvent.

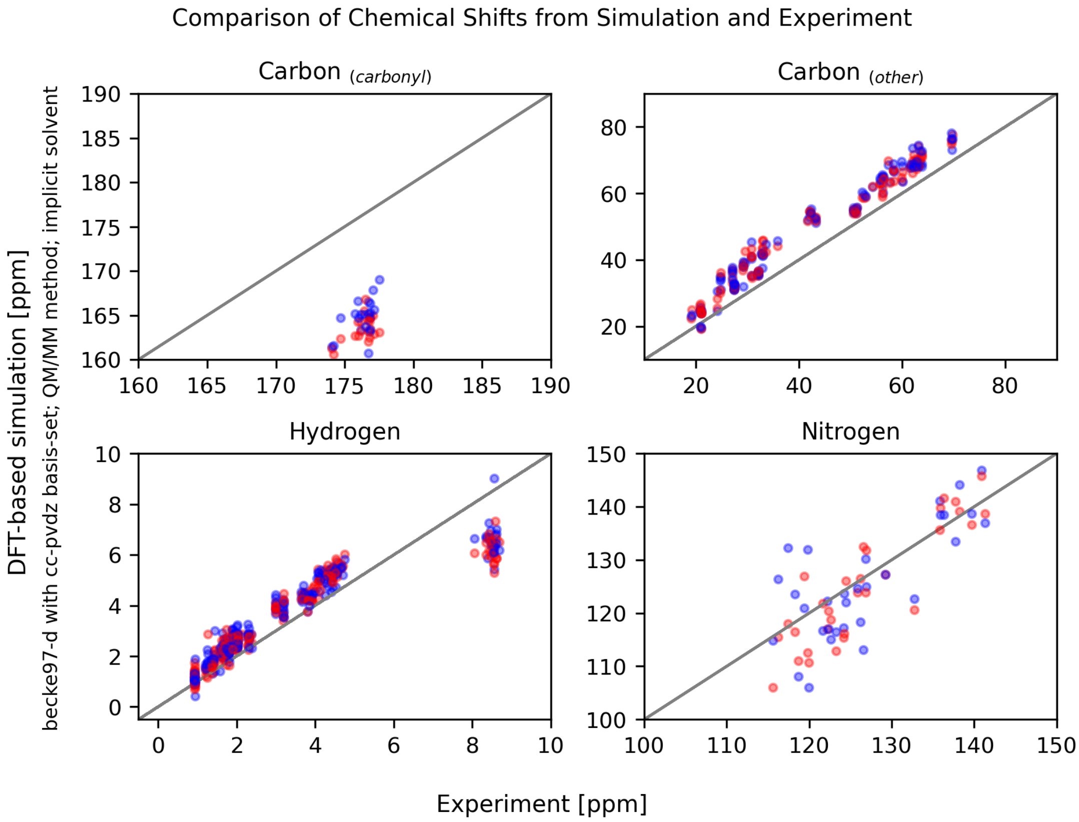

Figure S8.22: Comparison of experimental and simulated chemical shifts calculated with the DFT-based QM/MM method using becke97-d/cc-pvdz theory with implicit solvent.

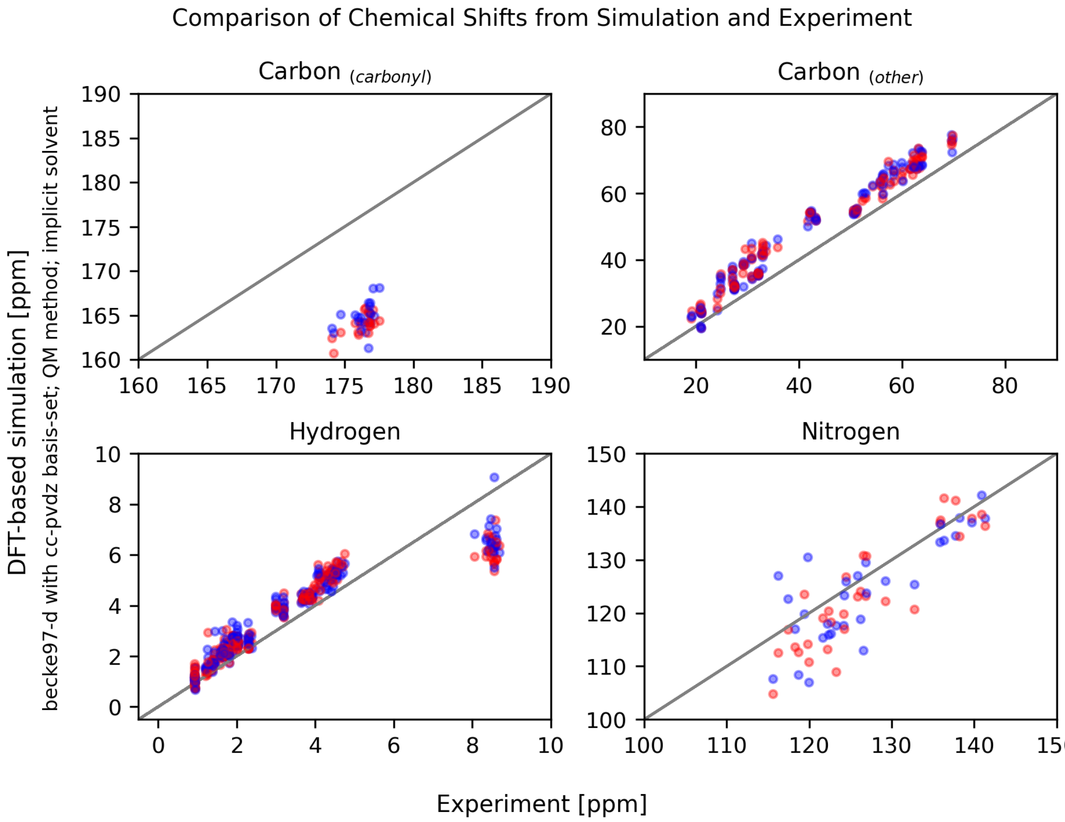

Figure S8.23: Comparison of experimental and simulated chemical shifts calculated with the DFT-based QM method using becke97-d/cc-pvdz theory with implicit solvent.

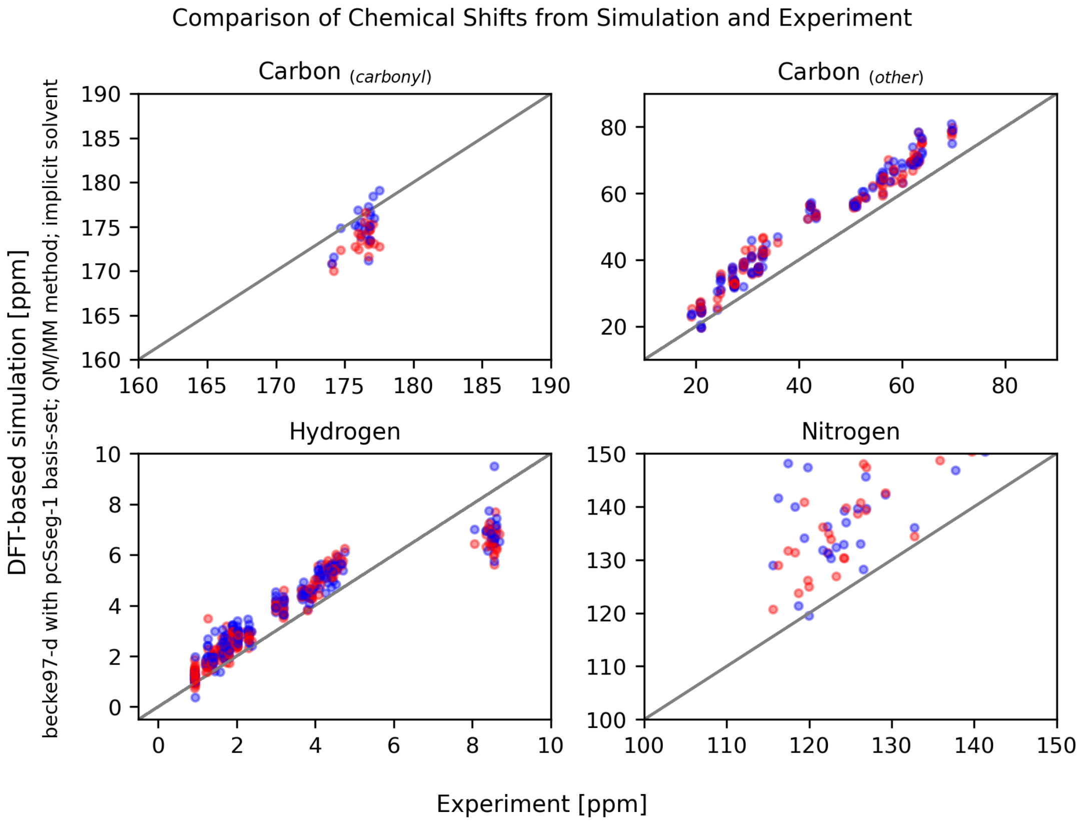

Figure S8.24: Comparison of experimental and simulated chemical shifts calculated with the DFT-based QM/MM method using becke97-d/pcSseg-1 theory with implicit solvent.

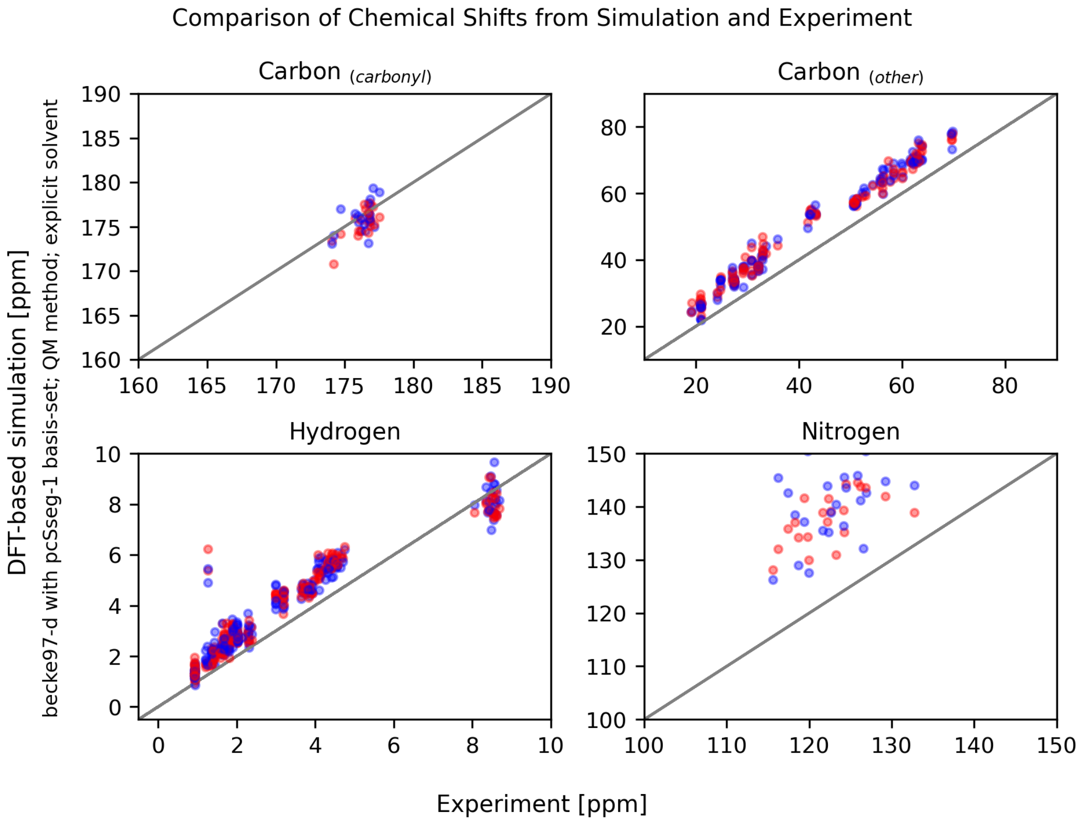

Figure S8.25: Comparison of experimental and simulated chemical shifts calculated with the DFT-based QM method using becke97-d/pcSseg-1 theory with explicit solvent.

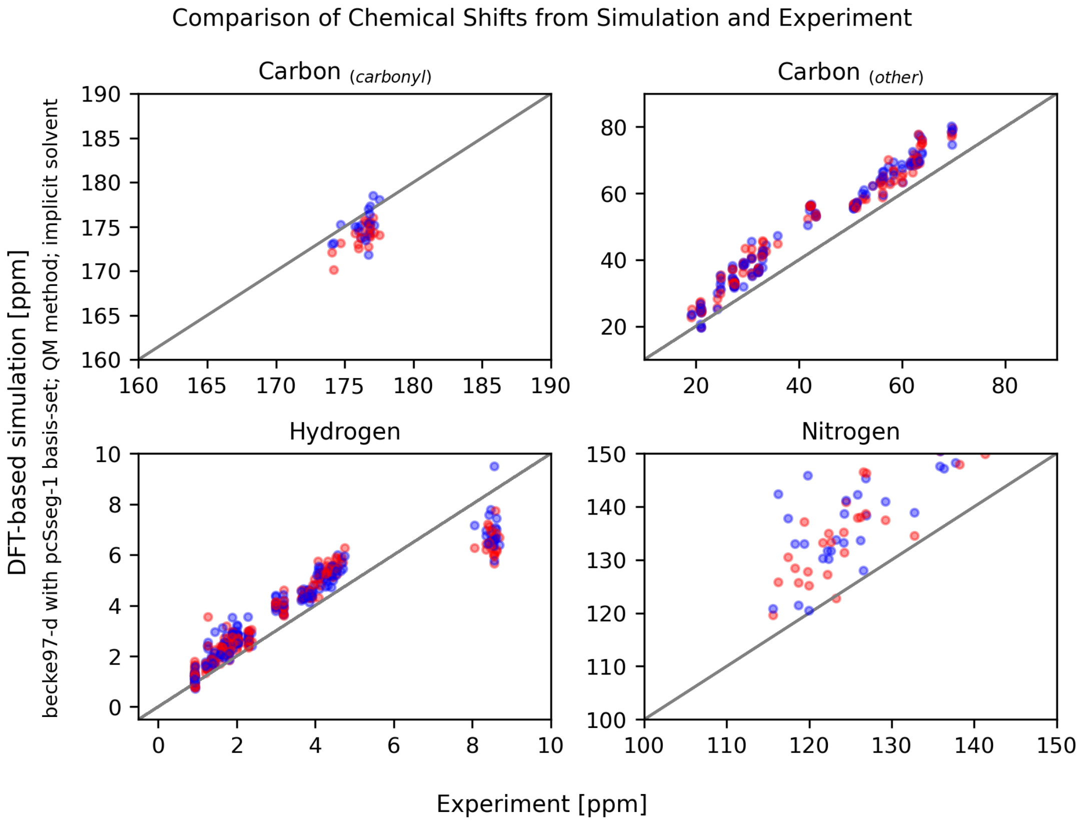

Figure S8.26: Comparison of experimental and simulated chemical shifts calculated with the DFT-based QM method using becke97-d/pcSseg-1 theory with implicit solvent.

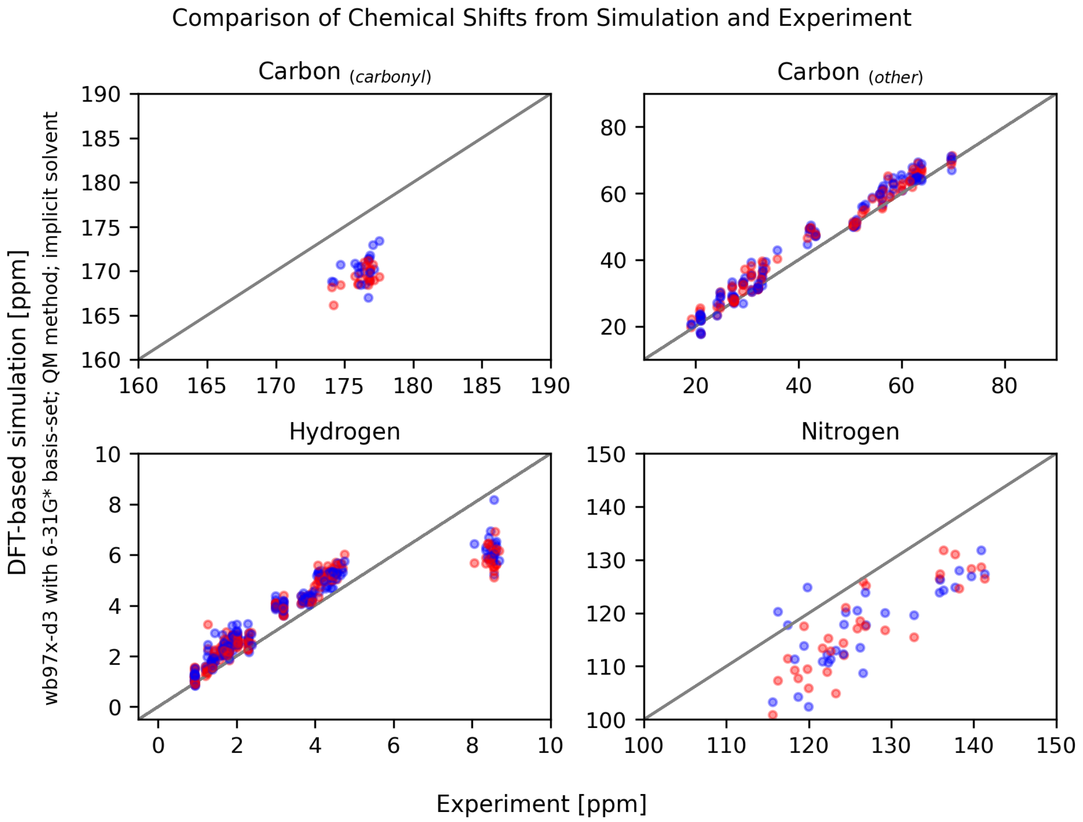

Figure S8.27: Comparison of experimental and simulated chemical shifts calculated with the DFT-based QM method using wb97x-d3/6-31G* theory with implicit solvent.

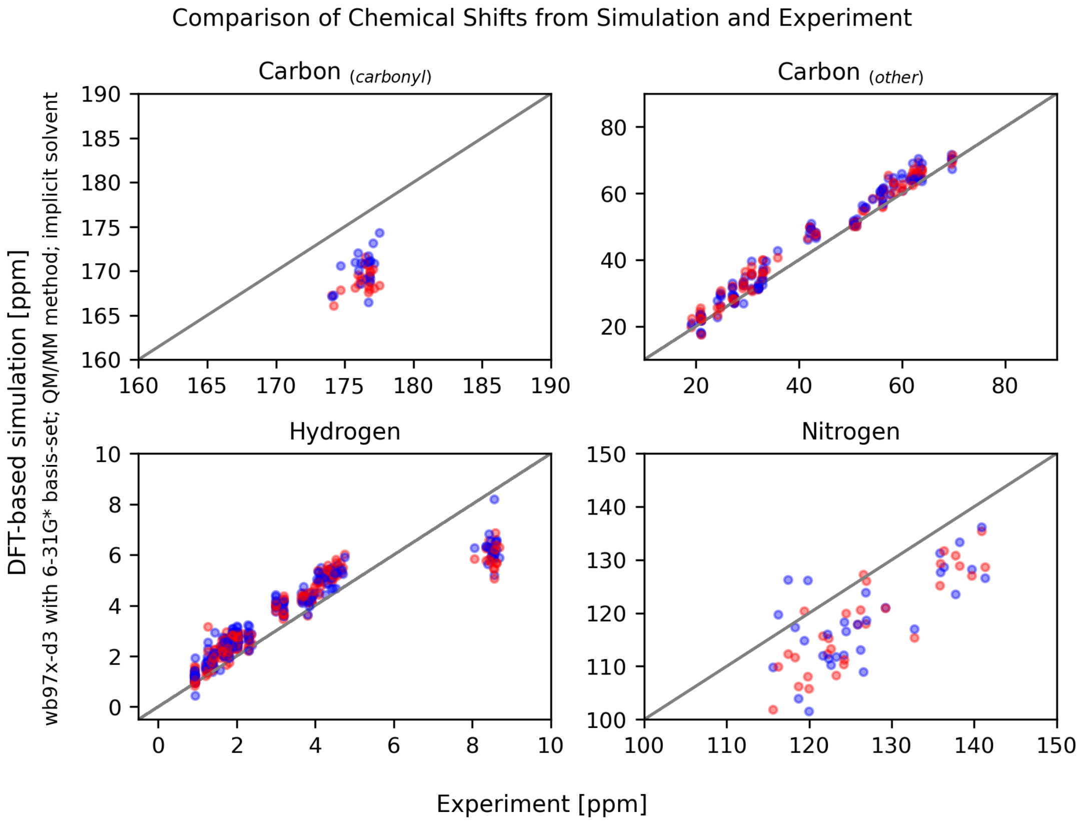

Figure S8.28: Comparison of experimental and simulated chemical shifts calculated with the DFT-based QM/MM method using wb97x-d3/6-31G* theory with implicit solvent.

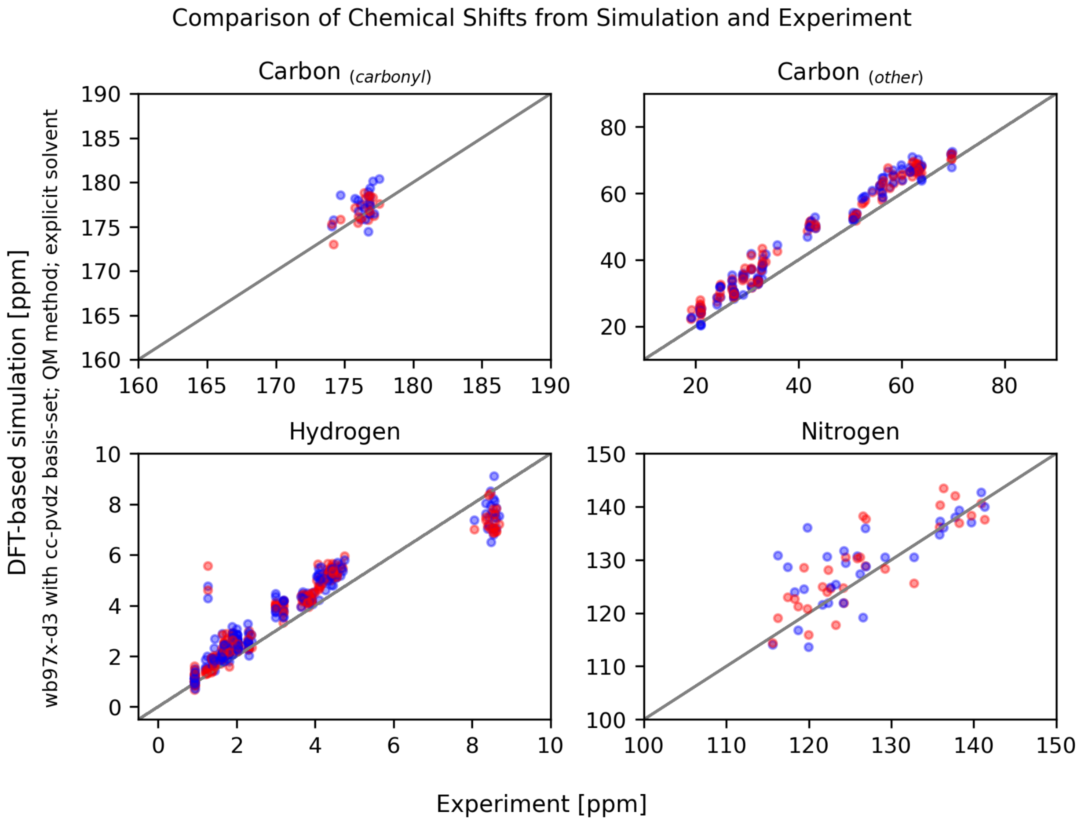

Figure S8.29: Comparison of experimental and simulated chemical shifts calculated with the DFT-based QM method using wb97x-d3/cc-pvdz theory with explicit solvent.

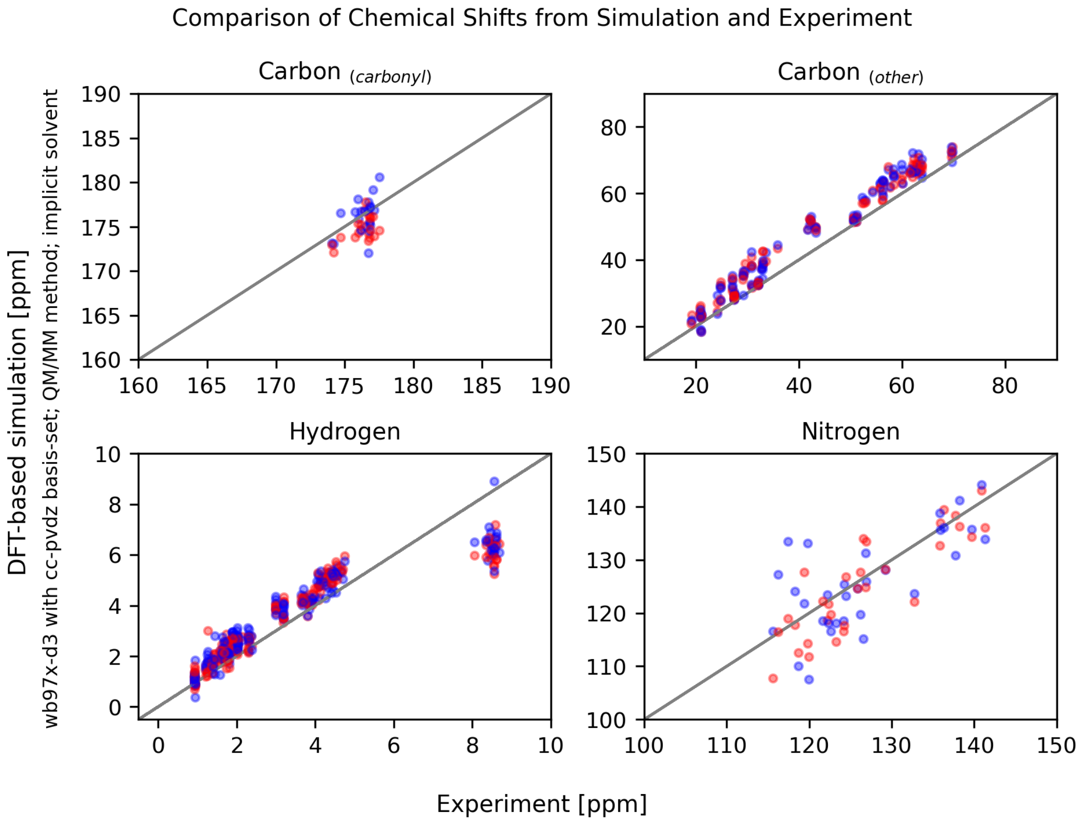

Figure S8.30: Comparison of experimental and simulated chemical shifts calculated with the DFT-based QM/MM method using wb97x-d3/cc-pvdz theory with implicit solvent.

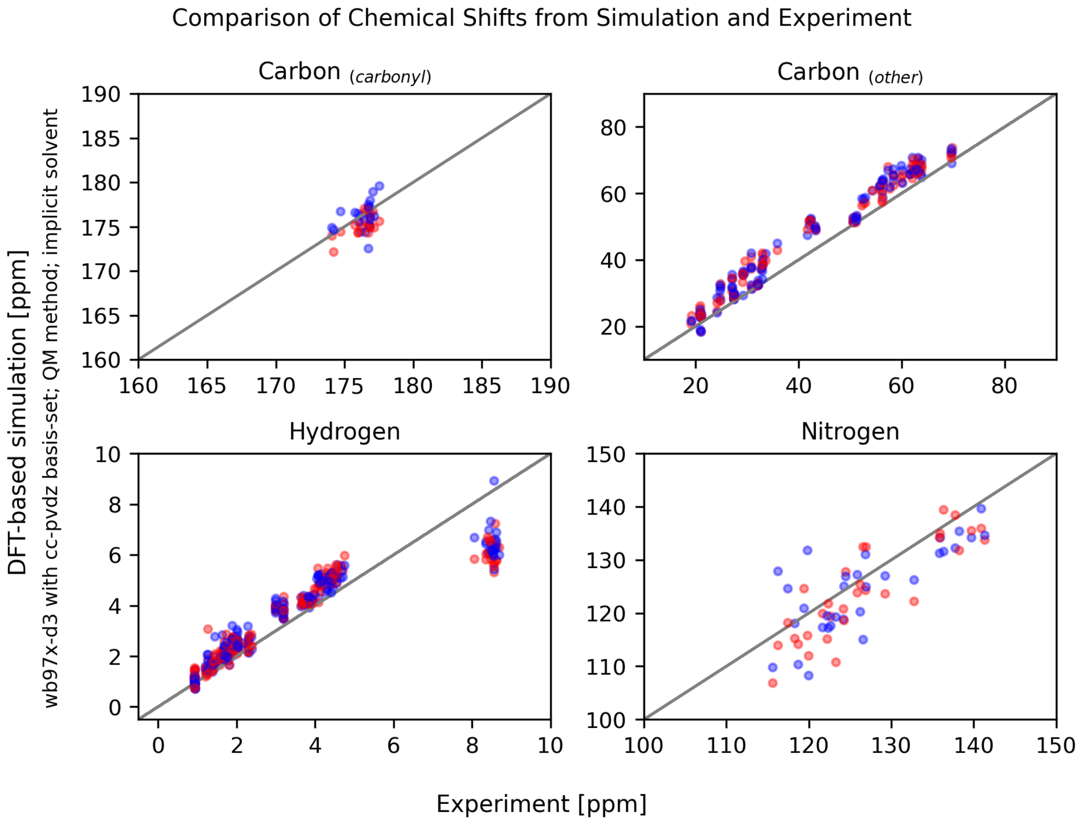

Figure S8.31: Comparison of experimental and simulated chemical shifts calculated with the DFT-based QM method using wb97x-d3/cc-pvdz theory with implicit solvent.

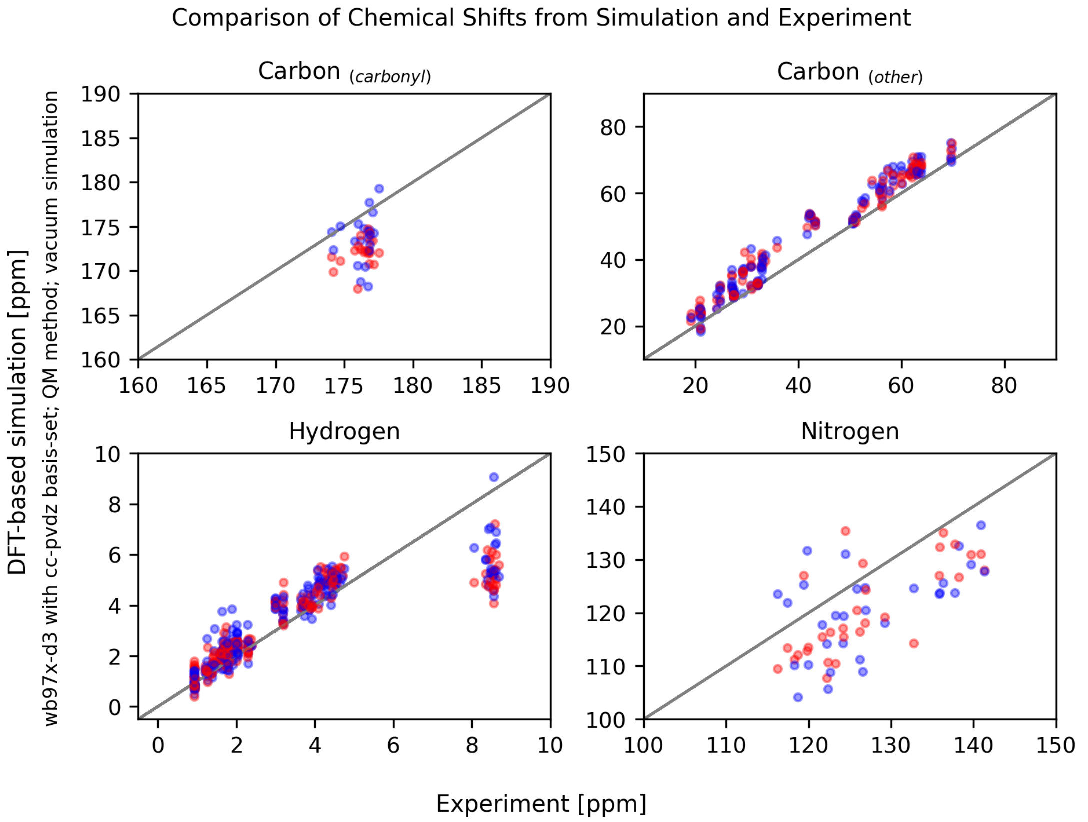

Figure S8.32: Comparison of experimental and simulated chemical shifts calculated with the DFT-based QM method using wb97x-d3/cc-pvdz theory in vacuum.

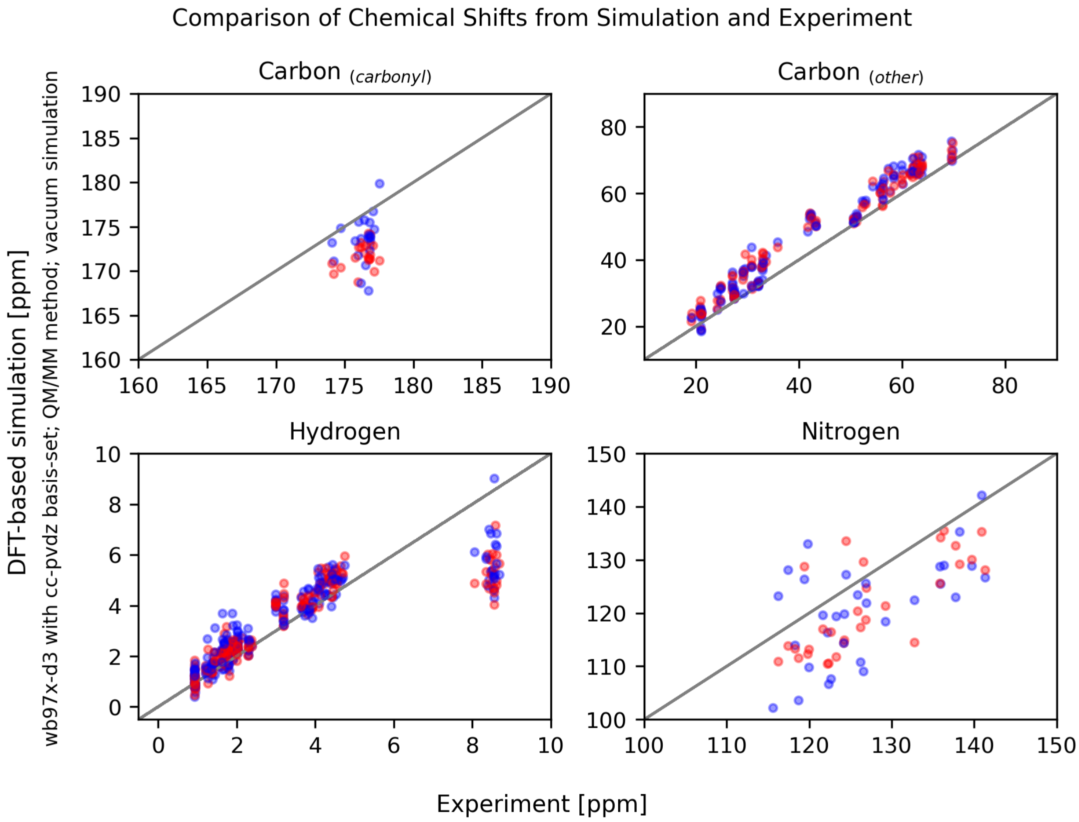

Figure S8.33: Comparison of experimental and simulated chemical shifts calculated with the DFT-based QM/MM method using wb97x-d3/cc-pvdz theory in vacuum.

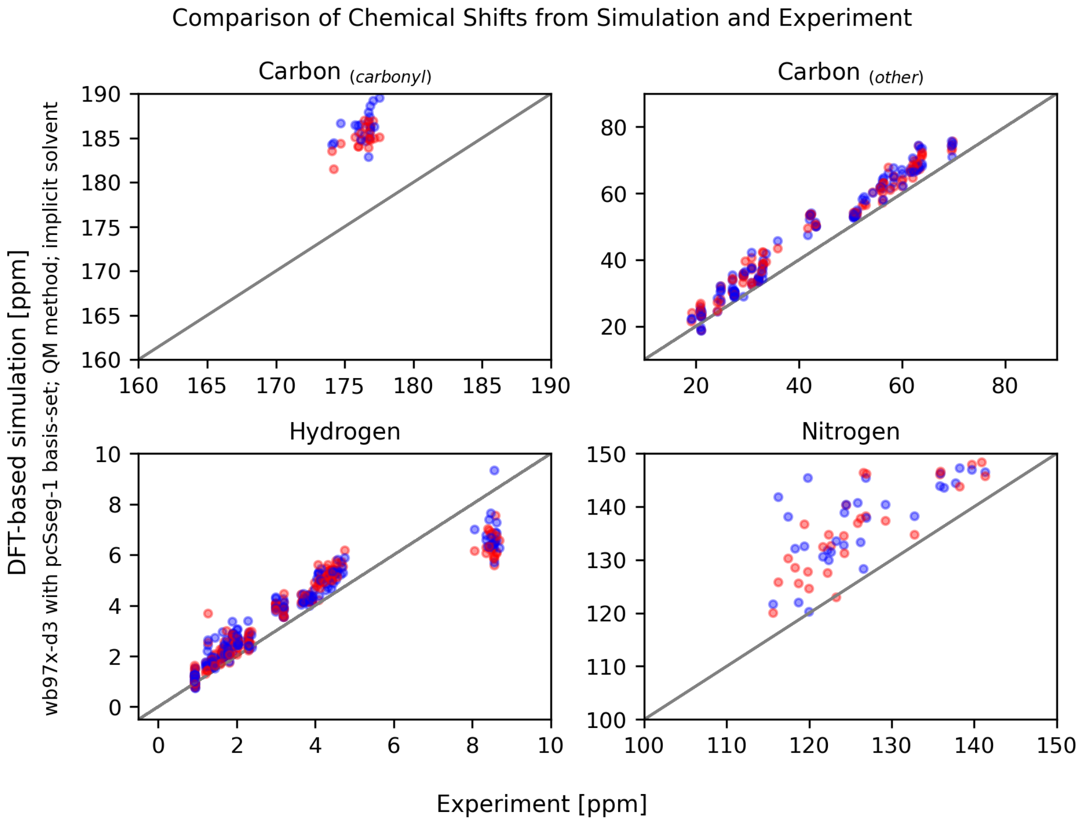

Figure S8.34: Comparison of experimental and simulated chemical shifts calculated with the DFT-based QM method using wb97x-d3/pcSseg-1 theory with implicit solvent.

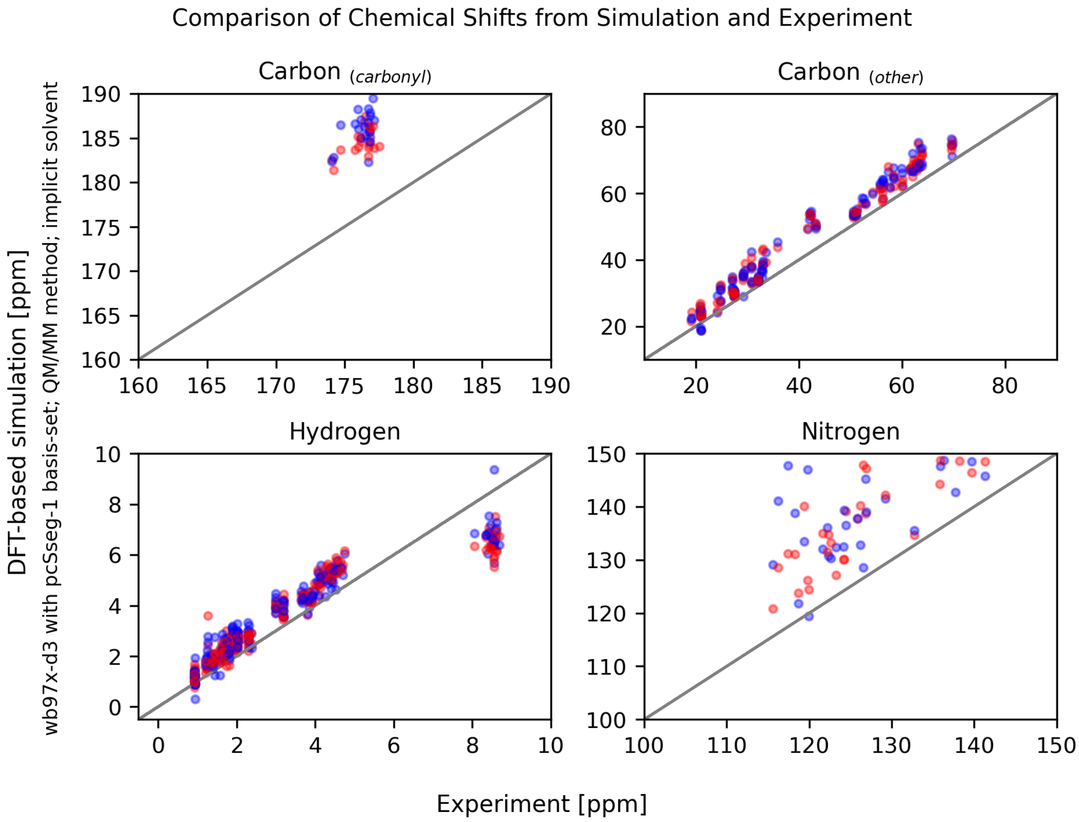

Figure S8.35: Comparison of experimental and simulated chemical shifts calculated with the DFT-based QM/MM method using wb97x-d3/pcSseg-1 theory with implicit solvent.

2 References

(1) A. Lasorsa, K. Bera, I. Malki, E. Dupré, F.-X. Cantrelle, H. Merzougui, D. Sinnaeve, X. Hanoulle, J. Hritz and I. Landrieu, "Conformation and Affinity Modulations by Multiple Phosphorylation Occurring in the BIN1 SH3 Domain Binding Site of the Tau Protein Proline-Rich Region," *Biochemistry*, 2023, **62**, 1631–1642.

(2) A. Lasorsa, I. Malki, F.-X. Cantrelle, H. Merzougui, E. Boll, J.-C. Lambert and I. Landrieu, "Structural Basis of Tau Interaction With BIN1 and Regulation by Tau Phosphorylation," *Frontiers in Molecular Neuroscience*, 2018, **11**, DOI: 10.3389/fnmol.2018.00421.
